# Supplementary material for: Research on quantitative evaluation of China’s Intelligent Construction Policy (CICP) based on the integration of PMC index model and multi-dimensional analytical framework
Source: PLoS One. 2025 Jul 15;20(7):e0326505. doi: 10.1371/journal.pone.0326505 (PMC12262895; doi:10.1371/journal.pone.0326505)
Supplement: S1 File — S1 Appendix. the thirty CICP texts. S2 Appendix. datasets for tables 1–14. S3 Appendix. datasets for figures 3–15. S4 Appendix. academic literature data of intelligent construction (CNKI). S5 Appendix. High-Frequency Keywords of intelligent construction. (ZIP) [file pone.0326505.s001.zip › data/data/S1 Appendix. the thirty CICP texts.docx]

**30份中国智能建造政策文本**

**The original documents of the thirty CICP texts**

**P1**

**Decision on Accelerating the Cultivation and Development of Strategic Emerging Industries**

**《国务院关于加快培育和发展战略性新兴产业的决定》**

国发〔2010〕32号

各省、自治区、直辖市人民政府，国务院各部委、各直属机构：
　　战略性新兴产业是引导未来经济社会发展的重要力量。发展战略性新兴产业已成为世界主要国家抢占新一轮经济和科技发展制高点的重大战略。我国正处在全面建设小康社会的关键时期，必须按照科学发展观的要求，抓住机遇，明确方向，突出重点，加快培育和发展战略性新兴产业。现作出如下决定：
　　一、抓住机遇，加快培育和发展战略性新兴产业
　　战略性新兴产业是以重大技术突破和重大发展需求为基础，对经济社会全局和长远发展具有重大引领带动作用，知识技术密集、物质资源消耗少、成长潜力大、综合效益好的产业。加快培育和发展战略性新兴产业对推进我国现代化建设具有重要战略意义。
　　（一）加快培育和发展战略性新兴产业是全面建设小康社会、实现可持续发展的必然选择。我国人口众多、人均资源少、生态环境脆弱，又处在工业化、城镇化快速发展时期，面临改善民生的艰巨任务和资源环境的巨大压力。要全面建设小康社会、实现可持续发展，必须大力发展战略性新兴产业，加快形成新的经济增长点，创造更多的就业岗位，更好地满足人民群众日益增长的物质文化需求，促进资源节约型和环境友好型社会建设。
　　（二）加快培育和发展战略性新兴产业是推进产业结构升级、加快经济发展方式转变的重大举措。战略性新兴产业以创新为主要驱动力，辐射带动力强，加快培育和发展战略性新兴产业，有利于加快经济发展方式转变，有利于提升产业层次、推动传统产业升级、高起点建设现代产业体系，体现了调整优化产业结构的根本要求。
　　（三）加快培育和发展战略性新兴产业是构建国际竞争新优势、掌握发展主动权的迫切需要。当前，全球经济竞争格局正在发生深刻变革，科技发展正孕育着新的革命性突破，世界主要国家纷纷加快部署，推动节能环保、新能源、信息、生物等新兴产业快速发展。我国要在未来国际竞争中占据有利地位，必须加快培育和发展战略性新兴产业，掌握关键核心技术及相关知识产权，增强自主发展能力。
　　加快培育和发展战略性新兴产业具备诸多有利条件，也面临严峻挑战。经过改革开放30多年的快速发展，我国综合国力明显增强，科技水平不断提高，建立了较为完备的产业体系，特别是高技术产业快速发展，规模跻身世界前列，为战略性新兴产业加快发展奠定了较好的基础。同时，也面临着企业技术创新能力不强，掌握的关键核心技术少，有利于新技术新产品进入市场的政策法规体系不健全，支持创新创业的投融资和财税政策、体制机制不完善等突出问题。必须充分认识加快培育和发展战略性新兴产业的重大意义，进一步增强紧迫感和责任感，抓住历史机遇，加大工作力度，加快培育和发展战略性新兴产业。
　　二、坚持创新发展，将战略性新兴产业加快培育成为先导产业和支柱产业
　　根据战略性新兴产业的特征，立足我国国情和科技、产业基础，现阶段重点培育和发展节能环保、新一代信息技术、生物、高端装备制造、新能源、新材料、新能源汽车等产业。
　　（一）指导思想。
　　以邓小平理论和“三个代表”重要思想为指导，深入贯彻落实科学发展观，把握世界新科技革命和产业革命的历史机遇，面向经济社会发展的重大需求，把加快培育和发展战略性新兴产业放在推进产业结构升级和经济发展方式转变的突出位置。积极探索战略性新兴产业发展规律，发挥企业主体作用，加大政策扶持力度，深化体制机制改革，着力营造良好环境，强化科技创新成果产业化，抢占经济和科技竞争制高点，推动战略性新兴产业快速健康发展，为促进经济社会可持续发展作出贡献。
　　（二）基本原则。
　　坚持充分发挥市场的基础性作用与政府引导推动相结合。要充分发挥我国市场需求巨大的优势，创新和转变消费模式，营造良好的市场环境，调动企业主体的积极性，推进产学研用结合。同时，对关系经济社会发展全局的重要领域和关键环节，要发挥政府的规划引导、政策激励和组织协调作用。
　　坚持科技创新与实现产业化相结合。要切实完善体制机制，大幅度提升自主创新能力，着力推进原始创新，大力增强集成创新和联合攻关，积极参与国际分工合作，加强引进消化吸收再创新，充分利用全球创新资源，突破一批关键核心技术，掌握相关知识产权。同时，要加大政策支持和协调指导力度，造就并充分发挥高素质人才队伍的作用，加速创新成果转化，促进产业化进程。
　　坚持整体推进与重点领域跨越发展相结合。要对发展战略性新兴产业进行统筹规划、系统布局，明确发展时序，促进协调发展。同时，要选择最有基础和条件的领域作为突破口，重点推进。大力培育产业集群，促进优势区域率先发展。
　　坚持提升国民经济长远竞争力与支撑当前发展相结合。要着眼长远，把握科技和产业发展新方向，对重大前沿性领域及早部署，积极培育先导产业。同时，要立足当前，推进对缓解经济社会发展瓶颈制约具有重大作用的相关产业较快发展，推动高技术产业健康发展，带动传统产业转型升级，加快形成支柱产业。
　　（三）发展目标。
　　到2015年，战略性新兴产业形成健康发展、协调推进的基本格局，对产业结构升级的推动作用显著增强，增加值占国内生产总值的比重力争达到8%左右。
　　到2020年，战略性新兴产业增加值占国内生产总值的比重力争达到15%左右，吸纳、带动就业能力显著提高。节能环保、新一代信息技术、生物、高端装备制造产业成为国民经济的支柱产业，新能源、新材料、新能源汽车产业成为国民经济的先导产业；创新能力大幅提升，掌握一批关键核心技术，在局部领域达到世界领先水平；形成一批具有国际影响力的大企业和一批创新活力旺盛的中小企业；建成一批产业链完善、创新能力强、特色鲜明的战略性新兴产业集聚区。
　　再经过十年左右的努力，战略性新兴产业的整体创新能力和产业发展水平达到世界先进水平，为经济社会可持续发展提供强有力的支撑。
　　三、立足国情，努力实现重点领域快速健康发展
　　根据战略性新兴产业的发展阶段和特点，要进一步明确发展的重点方向和主要任务，统筹部署，集中力量，加快推进。
　　（一）节能环保产业。重点开发推广高效节能技术装备及产品，实现重点领域关键技术突破，带动能效整体水平的提高。加快资源循环利用关键共性技术研发和产业化示范，提高资源综合利用水平和再制造产业化水平。示范推广先进环保技术装备及产品，提升污染防治水平。推进市场化节能环保服务体系建设。加快建立以先进技术为支撑的废旧商品回收利用体系，积极推进煤炭清洁利用、海水综合利用。
　　（二）新一代信息技术产业。加快建设宽带、泛在、融合、安全的信息网络基础设施，推动新一代移动通信、下一代互联网核心设备和智能终端的研发及产业化，加快推进三网融合，促进物联网、云计算的研发和示范应用。着力发展集成电路、新型显示、高端软件、高端服务器等核心基础产业。提升软件服务、网络增值服务等信息服务能力，加快重要基础设施智能化改造。大力发展数字虚拟等技术，促进文化创意产业发展。
　　（三）生物产业。大力发展用于重大疾病防治的生物技术药物、新型疫苗和诊断试剂、化学药物、现代中药等创新药物大品种，提升生物医药产业水平。加快先进医疗设备、医用材料等生物医学工程产品的研发和产业化，促进规模化发展。着力培育生物育种产业，积极推广绿色农用生物产品，促进生物农业加快发展。推进生物制造关键技术开发、示范与应用。加快海洋生物技术及产品的研发和产业化。
　　（四）高端装备制造产业。重点发展以干支线飞机和通用飞机为主的航空装备，做大做强航空产业。积极推进空间基础设施建设，促进卫星及其应用产业发展。依托客运专线和城市轨道交通等重点工程建设，大力发展轨道交通装备。面向海洋资源开发，大力发展海洋工程装备。强化基础配套能力，积极发展以数字化、柔性化及系统集成技术为核心的智能制造装备。
　　（五）新能源产业。积极研发新一代核能技术和先进反应堆，发展核能产业。加快太阳能热利用技术推广应用，开拓多元化的太阳能光伏光热发电市场。提高风电技术装备水平，有序推进风电规模化发展，加快适应新能源发展的智能电网及运行体系建设。因地制宜开发利用生物质能。
　　（六）新材料产业。大力发展稀土功能材料、高性能膜材料、特种玻璃、功能陶瓷、半导体照明材料等新型功能材料。积极发展高品质特殊钢、新型合金材料、工程塑料等先进结构材料。提升碳纤维、芳纶、超高分子量聚乙烯纤维等高性能纤维及其复合材料发展水平。开展纳米、超导、智能等共性基础材料研究。
　　（七）新能源汽车产业。着力突破动力电池、驱动电机和电子控制领域关键核心技术，推进插电式混合动力汽车、纯电动汽车推广应用和产业化。同时，开展燃料电池汽车相关前沿技术研发，大力推进高能效、低排放节能汽车发展。
　　四、强化科技创新，提升产业核心竞争力
　　增强自主创新能力是培育和发展战略性新兴产业的中心环节，必须完善以企业为主体、市场为导向、产学研相结合的技术创新体系，发挥国家科技重大专项的核心引领作用，结合实施产业发展规划，突破关键核心技术，加强创新成果产业化，提升产业核心竞争力。
　　（一）加强产业关键核心技术和前沿技术研究。围绕经济社会发展重大需求，结合国家科技计划、知识创新工程和自然科学基金项目等的实施，集中力量突破一批支撑战略性新兴产业发展的关键共性技术。在生物、信息、空天、海洋、地球深部等基础性、前沿性技术领域超前部署，加强交叉领域的技术和产品研发，提高基础技术研究水平。
　　（二）强化企业技术创新能力建设。加大企业研究开发的投入力度，对面向应用、具有明确市场前景的政府科技计划项目，建立由骨干企业牵头组织、科研机构和高校共同参与实施的有效机制。依托骨干企业，围绕关键核心技术的研发和系统集成，支持建设若干具有世界先进水平的工程化平台，结合技术创新工程的实施，发展一批由企业主导，科研机构、高校积极参与的产业技术创新联盟。加强财税政策引导，激励企业增加研发投入。加强产业集聚区公共技术服务平台建设，促进中小企业创新发展。
　　（三）加快落实人才强国战略和知识产权战略。建立科研机构、高校创新人才向企业流动的机制，加大高技能人才队伍建设力度。加快完善期权、技术入股、股权、分红权等多种形式的激励机制，鼓励科研机构和高校科技人员积极从事职务发明创造。加大工作力度，吸引全球优秀人才来华创新创业。发挥研究型大学的支撑和引领作用，加强战略性新兴产业相关专业学科建设，增加急需的专业学位类别。改革人才培养模式，制定鼓励企业参与人才培养的政策，建立企校联合培养人才的新机制，促进创新型、应用型、复合型和技能型人才的培养。支持知识产权的创造和运用，强化知识产权的保护和管理，鼓励企业建立专利联盟。完善高校和科研机构知识产权转移转化的利益保障和实现机制，建立高效的知识产权评估交易机制。加大对具有重大社会效益创新成果的奖励力度。
　　（四）实施重大产业创新发展工程。以加速产业规模化发展为目标，选择具有引领带动作用，并能够实现突破的重点方向，依托优势企业，统筹技术开发、工程化、标准制定、市场应用等环节，组织实施若干重大产业创新发展工程，推动要素整合和技术集成，努力实现重大突破。
　　（五）建设产业创新支撑体系。发挥知识密集型服务业支撑作用，大力发展研发服务、信息服务、创业服务、技术交易、知识产权和科技成果转化等高技术服务业，着力培育新业态。积极发展人力资源服务、投资和管理咨询等商务服务业，加快发展现代物流和环境服务业。
　　（六）推进重大科技成果产业化和产业集聚发展。完善科技成果产业化机制，加大实施产业化示范工程力度，积极推进重大装备应用，建立健全科研机构、高校的创新成果发布制度和技术转移机构，促进技术转移和扩散，加速科技成果转化为现实生产力。依托具有优势的产业集聚区，培育一批创新能力强、创业环境好、特色突出、集聚发展的战略性新兴产业示范基地，形成增长极，辐射带动区域经济发展。
　　五、积极培育市场，营造良好市场环境
　　要充分发挥市场的基础性作用，充分调动企业积极性，加强基础设施建设，积极培育市场，规范市场秩序，为各类企业健康发展创造公平、良好的环境。
　　（一）组织实施重大应用示范工程。坚持以应用促发展，围绕提高人民群众健康水平、缓解环境资源制约等紧迫需求，选择处于产业化初期、社会效益显著、市场机制难以有效发挥作用的重大技术和产品，统筹衔接现有试验示范工程，组织实施全民健康、绿色发展、智能制造、材料换代、信息惠民等重大应用示范工程，引导消费模式转变，培育市场，拉动产业发展。
　　（二）支持市场拓展和商业模式创新。鼓励绿色消费、循环消费、信息消费，创新消费模式，促进消费结构升级。扩大终端用能产品能效标识实施范围。加强新能源并网及储能、支线航空与通用航空、新能源汽车等领域的市场配套基础设施建设。在物联网、节能环保服务、新能源应用、信息服务、新能源汽车推广等领域，支持企业大力发展有利于扩大市场需求的专业服务、增值服务等新业态。积极推行合同能源管理、现代废旧商品回收利用等新型商业模式。
　　（三）完善标准体系和市场准入制度。加快建立有利于战略性新兴产业发展的行业标准和重要产品技术标准体系，优化市场准入的审批管理程序。进一步健全药品注册管理的体制机制，完善药品集中采购制度，支持临床必需、疗效确切、安全性高、价格合理的创新药物优先进入医保目录。完善新能源汽车的项目和产品准入标准。改善转基因农产品的管理。完善并严格执行节能环保法规标准。
　　六、深化国际合作，提高国际化发展水平
　　要通过深化国际合作，尽快掌握关键核心技术，提升我国自主发展能力与核心竞争力。把握经济全球化的新特点，深度开展国际合作与交流，积极探索合作新模式，在更高层次上参与国际合作。
　　（一）大力推进国际科技合作与交流。发挥各种合作机制的作用，多层次、多渠道、多方式推进国际科技合作与交流。鼓励境外企业和科研机构在我国设立研发机构，支持符合条件的外商投资企业与内资企业、研究机构合作申请国家科研项目。支持我国企业和研发机构积极开展全球研发服务外包，在境外开展联合研发和设立研发机构，在国外申请专利。鼓励我国企业和研发机构参与国际标准的制定，鼓励外商投资企业参与我国技术示范应用项目，共同形成国际标准。
　　（二）切实提高国际投融资合作的质量和水平。完善外商投资产业指导目录，鼓励外商设立创业投资企业，引导外资投向战略性新兴产业。支持有条件的企业开展境外投资，在境外以发行股票和债券等多种方式融资。扩大企业境外投资自主权，改进审批程序，进一步加大对企业境外投资的外汇支持。积极探索在海外建设科技和产业园区。制定国别产业导向目录，为企业开展跨国投资提供指导。
　　（三）大力支持企业跨国经营。完善出口信贷、保险等政策，结合对外援助等积极支持战略性新兴产业领域的重点产品、技术和服务开拓国际市场，以及自主知识产权技术标准在海外推广应用。支持企业通过境外注册商标、境外收购等方式，培育国际化品牌。加强企业和产品国际认证合作。
　　七、加大财税金融政策扶持力度，引导和鼓励社会投入
　　加快培育和发展战略性新兴产业，必须健全财税金融政策支持体系，加大扶持力度，引导和鼓励社会资金投入。
　　（一）加大财政支持力度。在整合现有政策资源和资金渠道的基础上，设立战略性新兴产业发展专项资金，建立稳定的财政投入增长机制，增加中央财政投入，创新支持方式，着力支持重大关键技术研发、重大产业创新发展工程、重大创新成果产业化、重大应用示范工程、创新能力建设等。加大政府引导和支持力度，加快高效节能产品、环境标志产品和资源循环利用产品等推广应用。加强财政政策绩效考评，创新财政资金管理机制，提高资金使用效率。
　　（二）完善税收激励政策。在全面落实现行各项促进科技投入和科技成果转化、支持高技术产业发展等方面的税收政策的基础上，结合税制改革方向和税种特征，针对战略性新兴产业的特点，研究完善鼓励创新、引导投资和消费的税收支持政策。
　　（三）鼓励金融机构加大信贷支持。引导金融机构建立适应战略性新兴产业特点的信贷管理和贷款评审制度。积极推进知识产权质押融资、产业链融资等金融产品创新。加快建立包括财政出资和社会资金投入在内的多层次担保体系。积极发展中小金融机构和新型金融服务。综合运用风险补偿等财政优惠政策，促进金融机构加大支持战略性新兴产业发展的力度。
　　（四）积极发挥多层次资本市场的融资功能。进一步完善创业板市场制度，支持符合条件的企业上市融资。推进场外证券交易市场的建设，满足处于不同发展阶段创业企业的需求。完善不同层次市场之间的转板机制，逐步实现各层次市场间有机衔接。大力发展债券市场，扩大中小企业集合债券和集合票据发行规模，积极探索开发低信用等级高收益债券和私募可转债等金融产品，稳步推进企业债券、公司债券、短期融资券和中期票据发展，拓宽企业债务融资渠道。
　　（五）大力发展创业投资和股权投资基金。建立和完善促进创业投资和股权投资行业健康发展的配套政策体系与监管体系。在风险可控的范围内为保险公司、社保基金、企业年金管理机构和其他机构投资者参与新兴产业创业投资和股权投资基金创造条件。发挥政府新兴产业创业投资资金的引导作用，扩大政府新兴产业创业投资规模，充分运用市场机制，带动社会资金投向战略性新兴产业中处于创业早中期阶段的创新型企业。鼓励民间资本投资战略性新兴产业。
　　八、推进体制机制创新，加强组织领导
　　加快培育和发展战略性新兴产业是我国新时期经济社会发展的重大战略任务，必须大力推进改革创新，加强组织领导和统筹协调，为战略性新兴产业发展提供动力和条件。
　　（一）深化重点领域改革。建立健全创新药物、新能源、资源性产品价格形成机制和税费调节机制。实施新能源配额制，落实新能源发电全额保障性收购制度。加快建立生产者责任延伸制度，建立和完善主要污染物和碳排放交易制度。建立促进三网融合高效有序开展的政策和机制，深化电力体制改革，加快推进空域管理体制改革。
　　（二）加强宏观规划引导。组织编制国家战略性新兴产业发展规划和相关专项规划，制定战略性新兴产业发展指导目录，开展战略性新兴产业统计监测调查，加强与相关规划和政策的衔接。加强对各地发展战略性新兴产业的引导，优化区域布局、发挥比较优势，形成各具特色、优势互补、结构合理的战略性新兴产业协调发展格局。各地区要根据国家总体部署，从当地实际出发，突出发展重点，避免盲目发展和重复建设。
　　（三）加强组织协调。成立由发展改革委牵头的战略性新兴产业发展部际协调机制，形成合力，统筹推进。
　　国务院各有关部门、各省（区、市）人民政府要根据本决定的要求，抓紧制定实施方案和具体落实措施，加大支持力度，加快将战略性新兴产业培育成为先导产业和支柱产业，为我国现代化建设作出新的贡献。
　　　　　　　　　　　　　　　　　　　　　　　　　　　　　　国务院

二○一○年十月十日

**P2**

**Guidelines on the Priority Development of High-tech Industrialization Key Areas (2011)**

**当前优先发展的高技术产业化重点领域指南（2011 年度）**

2011年第10号

修订说明

2007 年，国家发展改革委、科技部、商务部、知识产权局联合发布 了《当前优先发展的高技术产业化重点领域指南（2007 年度）》（国家发 展改革委2007 年第6 号公告，以下简称《指南（2007 年度）》），对指导 各部门、各地方开展高技术产业化工作，促进产业结构调整、加快经济 发展方式转变，引导社会资源投向等发挥了重要作用。为贯彻党的十七 届五中全会精神，落实《国民经济和社会发展第十二个五年规划纲要》、 《国家中长期科学和技术发展规划纲要（2006-2020）》，进一步发挥“指 南”的指导作用，国家发展改革委、科技部、工业和信息化部、商务部、 知识产权局，在充分分析国内外高技术发展现状及趋势，广泛征求意见 的基础上，研究提出了《当前优先发展的高技术产业化重点领域指南（2011

年度）》（以下简称《指南（2011 年度）》）。

《指南（2011 年度）》确定了当前优先发展的信息、生物、航空航天、 新材料、先进能源、现代农业、先进制造、节能环保和资源综合利用、 海洋、高技术服务十大产业中的 137 项高技术产业化重点领域，其中， 信息15 项，生物17 项，航空航天 6 项，新材料24 项，先进能源 13 项， 现代农业 18 项，先进制造21 项，节能环保和资源综合利用 9 项，海洋6 项，高技术服务 8 项。重点内容体现了发展高技术产业、大力培育发展 战略性新兴产业，推进产业结构优化升级、促进经济发展方式转变，应 对全球气候变化的新需求。与《指南（2007 年度）》相比，《指南（2011 年度）》新增了高技术服务产业和 15 项重点领域，删除了 8 项已基本实现产业化的重点领域，并对各领域下的具体内容进行了调整。

一、信息

1、 网络设备

适用于下一代高速宽带信息网和三网融合应用的网络产品，物联网关键设 备，基于 IPv4/IPv6 的高性能路由器/交换机，能够提供端到端服务质量(QoS) 、 支持多功能多业务、安全的网络技术及设备， Tbit 以上大容量汇聚交换设备，软 交换设备、网关，IP 多媒体子系统（IMS）设备，流媒体系统设备，相应的网络 测试设备，与物联网有关的分布式感知、拓扑控制、信息资源调度、协同计算等

关键设备和产品。

2、 光传输设备

40Gbit/s、100Gbit/s 超大容量密集波分复用（DWDM）设备，可重构光分差 复用设备（ROADM）及波分复用系统用光交叉互连（OXC）设备，智能光网络

传输设备（ASON），多业务传输设备，高速光器件（有源和无源）。

3、 接入网系统设备

宽带、有线、无线和卫星等多种接入技术、专用芯片及系统设备，包括 10G 无源光纤网（xPON）接入、宽带光纤接入（FTTH）、同轴电缆接入、宽带无线 城域网、近距离超高频无线通信等多种宽带接入技术及设备；适用于三网融合的 统一身份认证与访问控制网关，以及接入网统一网管系统、专用芯片及设备；物

联网感知技术及无线射频（RFID）产品。

4、 数字移动通信产品

3G 增强/长期演进型技术产品，新一代移动通信系统（含移动互联网）的网 络设备、智能终端、专用芯片、操作系统、业务平台及应用软件，与新一代移动

通信有关的设备关键配套件及测试仪器，宽带集群通信系统及设备。

5、 数字音视频产品

面向三网融合的数字音视频编解码（AVS 、DRA 等）技术与数字电视音视 频信号处理相关的关键设备、专用芯片、关键部件（数字高清成像器件和智能监 控产品），机卡分离的数字有线电视前端、条件接收、中间件、 一体化机产品， 地面数字电视、数字和移动多媒体广播电视发射、接收产品及设备，卫星直播电 视专用芯片及终端产品， 3D 显示技术与设备，数字电影产品及设备，高密度数

字激光视盘机及关键部件，数字摄录一体机及数码相机，与数字电视内容有关数字版权管理、内容分发、安全保障等关键技术和设备，信息设备资源共享关联 应用技术与产品，高档数字音响系统等家庭信息终端， 4C（计算机、通信、消

费电子、内容）融合产品等新型消费类电子产品。

6、 计算机及外部设备

高端服务器及配套软件，云计算模式下的新型终端、新型云计算结点、网络 存储及海量存储设备，大容量高速率的移动存储器，固态硬盘（SSD）以及 PB 级海量存储管理集群文件系统，面向超大规模复杂数据的分级和虚拟化存储管理 系统，光、磁盘驱动器，无线网卡和模式识别输入设备，生物特征识别及智能卡 系统，激光打印机，新型专用打印机，自助服务终端产品，各类计算机外部设备

关键部件。

7、 软件及应用系统

服务器操作系统、桌面操作系统和网络（云计算）操作系统，数据库管理系 统和支撑软件，多媒体数据库以及检索系统；嵌入式操作系统、嵌入式软件开发 平台等核心支撑软件；基础中间件，云计算资源自动调度管理中间件，面向应用 的中间件；基于 Web 服务的核心软件，面向 Web 服务计算环境的网络系统软件，

基于各种相关软件技术研究和软件开发平台研制的网构化软件生产平台。

高性能计算机应用软件和跨平台共性应用软件（CAD ，图像处理等），虚拟 现实与平台；网络搜索引擎，中文的全文检索，中文信息处理（含少数民族语言 信息处理、中文和外文间的机器翻译），文字识别、语音合成与识别。分布式无 线射频编码解析服务系统软件、编码解析安全管理系统软件， RFID 技术公共服 务平台；物联网应用平台，信息组织、控制、处理技术和软件系统， RFID 与无 线通信、传感技术、生物识别等技术融合系统。电子政务信息系统，包括政务办 公和决策支持系统，政府综合监管系统、预警预报与应急响应系统、执法系统， 公共服务系统，舆情监测分析系统，经济运行分析系统与监测预警系统，政务信 息公开目录及交换体系，绩效评估分析体系，政府信息资源数据中心，安全认证 及保障平台；电子商务信息系统，包括交易与服务、供应链管理、加密与电子认 证、在线支付、信用管理、多式联运技术与系统及相关应用产品，物流信息服务 技术与平台；社会信息化系统，包括社会保障、医疗保障系统、社区服务管理系 统、教育培训系统、城市应急联动信息系统等；企业信息化系统，包括企业信息

基础设施、协同设计与仿真、产品数据管理系统、企业资源管理系统等；工业软

件，重要行业的管理和应用软件。

8、 信息安全产品与系统

适用于特殊领域的安全服务器，安全路由器，安全网络存储设备，计算机安 全操作系统，安全数据库，操作系统与数据库安全管理系统防病毒和防攻击系统， 入侵检测、防信息泄漏、后门发现和漏洞分析，加解密设备和芯片，安全协议， 公钥基础结构（PKI）系统，组合公钥（CPK）系统，安全支付系统，电子防伪 系统以及网络安全监控系统，虚拟专用网和无线网络领域的安全监管系统，等级 保护管理，可信计算技术与产品，网络安全预警系统，内容安全和网络容灾类产 品，信息系统安全测评系统、软件测评技术与系统，保障云计算、物联网、新一

代信息网络以及面向三网融合的安全产品，多媒体内容监管系统。

9、 集成电路

高性能传感器及关键芯片、高速集成电路技术及芯片、线宽 65 纳米以下的 纳米级集成电路芯片制造、封装和测试，纳米级芯片设计平台（EDA 工具）及 配套 IP 库，设计、开发智能存储卡控制器芯片以及整机所需的各种专用集成电 路芯片和系统级芯片，低功耗、高性能数字信号处理器（DSP），低功耗、高性

能嵌入式中央处理器（CPU）及其系统级芯片，高性能多核 32 位／64 位 CPU。

10、 信息功能材料与器件

以氮化镓、碳化硅、氮化铝为代表的第三代（高温宽带隙）半导体材料与器 件，蓝宝石晶片、石墨烯和碳纳米管混合材料，高 k 栅介质和金属栅极材料，新 型微电子和光电子材料与器件，大尺寸光纤预制棒及配套材料，光子晶体材料与 器件，硅基光电子材料与器件，半导体纳米结构材料与器件，光传感用光电子材 料与高端核心器件，轧制印刷电路板及锂电池用高性能、低轮廓电子铜箔、 IC 引线框架铜带、封装基板材料、高频、高耐热性覆铜板、无铅焊料，高性能永磁 软磁铁氧体材料与器件、低损耗电容器纸、8－12 吋硅片生产设备的配套材料（超

高纯石英材料）。

11、 电子专用设备、仪器和工模具

8-12 英吋集成电路生产设备、封装测试设备，无线射频（RFID）封装设备， 化合物半导体生产设备，碳化硅单晶材料生长设备，片式元件生产设备，半导体 照明设备、光伏太阳能设备、新型显示专用设备、敏感元器件/传感器件生产设

备，高频率器件生产设备，电力电子器件生产设备，超净设备，环境试验设备，

高精度电子专用模具，终测仪、路测仪等电子专用测试仪器。

12、 新型显示器件

大屏幕高端 LED 显示、TFT－LCD、PDP、OLED 显示、场致发光显示（FED）、 激光显示、3.5－13.5 英吋电容式触摸屏、电子纸、3D 显示等新型显示技术及器 件，新型显示面板生产、整机模组一体化设计、玻璃基板制造等关键技术，以及 相关的驱动电路、光学引擎、彩色滤光片、偏光片、光学薄膜等配套材料，LED 背光源、大屏幕液晶显示器（TFT－LCD）光掩膜用大尺寸掩膜板、 TFT－LCD 用靶材，等离子显示器（PDP）和有机发光二极管（OLED）用材料，高亮度 LED

外延片及芯片及封装技术。

13、 新型元器件

高档片式元器件，新型机电元件，微机电系统（MEMS），光集成和光电集 成器件，半导体激光器件，光纤激光器件，高性能全固态激光器件，高性能敏感 元器件及传感器，高端混合集成电路和高频器件，高密度多层印刷电路板和柔性 电路板，小型精密无刷电动机，微型通讯电声器件，新型晶体器件，高精密电阻 器件，超导滤波器，中大功率高压绝缘栅双极晶体管（IGBT）、快恢复二极管 （FRD）芯片和模块，中小功率智能模块；高电压的金属氧化物半导体场效应管

（MOSFET）；大功率集成门极换流晶闸管（IGCT）；6 吋大功率晶闸管。

14、 汽车电子

汽油机和柴油机动力总成控制系统，电机控制系统，动力电池管理系统，自 动变速控制系统，电控动力转向系统，主/被动安全控制系统，电子控制制动系 统，关键元器件和车用集成电路芯片，关键车用传感器，车用总线网络系统、车 辆维修诊断系统，车载雷达及相关图象处理软件、零事故智能交通系统，基于车 载自动诊断系统（ODBIII）的远程车辆信息采集监控系统，车载综合信息系统，

数字化仪表。

15、 民用雷达

半高层大气探测雷达（MST 甚高频雷达和激光雷达），新一代天气雷达(双 极化、双多基地和相控阵雷达)，机载测风雷达，毫米波（3 毫米）云雷达，海洋 状态监测雷达，探地雷达，船用导航雷达，空管全固态一次雷达和 S 模式二次雷 达、合成孔径雷达，通用航空机场雷达，机动多功能航管雷达，机场场面监视雷

达，雷达综合应用平台，组网雷达数据分析与共享平台。

二、生物

16、 生物反应及分离技术

高效生物反应器，高密度培养技术，佐剂、悬浮培养、发酵培养等生物制品 产业化关键技术及动植物生物反应器技术，大规模高效分离技术、介质和设备， 大型分离系统及在线检测控制装置，基因工程、细胞工程和蛋白质工程产品专用

分离设备，生物过程参数传感器和自控系统。

17、 生物制造关键技术及重大产品

新型高效工业、食品、医药和环保等专用酶制剂，酶制剂质量评价技术及标 准，以动植物为原料深加工药物中间体，功能性淀粉糖（醇），小品种高附加值 的氨基酸和有机酸、生物防腐剂、生物絮凝剂等新型微生物制造的食品和大宗发

酵制品，生物反应废液生物酶分解技术，抗生素和维生素的绿色生产技术。

18、 新型疫苗

预防流行性呼吸系统疾病、艾滋病、肝炎、结核病、布氏菌病、出血热、疟 疾、钩虫病、血吸虫病、手足口病、肠道疾病、自然疫源性疾病等传染病和治疗 肿瘤等慢病的联合疫苗、治疗性疫苗、口服疫苗、新型佐剂等，疫苗生产用清洁

动物、细胞基质。

19、 重大疾病创新药物及关键技术

新型抗恶性肿瘤疾病、抗心脑血管疾病、糖尿病等内分泌疾病,抗肝炎、艾 滋、结核等抗感染类疾病，抗老年性痴呆、帕金森氏症等神经退行性疾病及神经 精神类疾病、非成瘾性镇痛、戒毒类等的创新药物、通用名大品种药物、特色药 物。药物生产的绿色合成、手性拆分、晶型制备技术，药物生产在线质量控制技

术，药物信息技术。

20、 生物技术药物及关键技术

基因工程药物、抗体药物、多肽药物、核酸药物等的规模化制备技术，蛋白 质工程技术，聚乙二醇化学修饰技术，干细胞治疗相关技术，多肽药物大规模合

成技术，治疗性抗体生产技术，科研用试剂关键技术及产品,医学实验动物。

21、 单克隆抗体系列产品与检测试剂

传染病早期检测诊断试剂及试剂盒，病毒细菌感染鉴别诊断试剂及试剂盒， 新型系列肿瘤标记物检测试剂及试剂盒，出生缺陷早期筛查试剂及试剂盒，食品

安全检测试剂及试剂盒，动植物疫病检测试剂及试剂盒。

22、 新型给药技术及药物新剂型

新型释药系统，包括缓释、控释、靶向给药技术，蛋白或多肽类药物的口服 给药技术及制剂，药物控释纳米材料和药物新晶型制备技术，新型给药技术、装

备和辅料，中药新剂型及其新型辅料。

23、 计划生育药具

生育调节药具，具有避孕和预防生殖道感染双重功能的避孕节育新技术，米 非司酮新剂型，第三代甾体激素避孕药的缓释、控释新材料、新剂型，中药口服 避孕药，妊娠和生殖检测技术，新型终止妊娠技术，新剂型避孕疫苗，新型医用

高分子材料避孕套具和避孕制剂。

24、 中药材及饮片

道地和紧缺中药材优质种源繁育及规范化种植、养殖，重要、濒危野生中药 材人工栽培，中药种质及活性成分资源库，中药饮片炮制技术和新型中药饮片生

产技术和装备。

25、 中药制品

防治呼吸系统疾病、肿瘤、肝病、心脑血管疾病、免疫功能性疾病、胃肠道

疾病、病毒性疾病、糖尿病、老年性疾病和妇科疾病等中药新药及预防保健产品。

26、 中药制药工艺及设备

中药有效成分提取制备技术及组装式生产自动化生产线，中药制药工艺参数 在线检测和自动化控制系统，中药制药过程质量监控技术，中药材加工、制药技

术和工艺装备。

27、 生物医学材料

骨、牙及关节系统用生物活性修复替换材料，牙用人工材料和体内植入物， 组织工程血管、人工心瓣膜等心血管系统替换材料和制品，心血管支架，软骨、 骨、肌腱、周围神经、皮肤、眼角膜等组织工程支架及干细胞或其他体细胞结构 和功能性再生组织，用于微创手术的材料和结构，介入导管和器件，介入性治疗

材料，血浆代用品，血液净化材料和体外循环装置，医学材料表面处理设备。

28、 新型医用精密诊断及治疗设备

肿瘤等重大疾病的新型诊疗设备，新型便携式诊疗设备，新型多功能激光治 疗设备，微创手术及介入治疗设备， CT、彩超、磁共振、 X 射线等大型设备及

成像材料和关键零部件，新型血液净化处理设备，新型急救、诊断、康复设备。

29、 医学信息技术及远程医疗

适用于个人、家庭、社区、农村基层诊所及医院的信息服务系统及带有相应 信息化功能的便携式分析、监护、诊断及预防治疗仪器，病人信息数据库、专家 系统，医学信息数据库、数字医学影像存储与传输系统，远程医疗诊断、监护和

教育系统，社区卫生服务网络系统，数字医学信息处理专用软件。

30、 生物芯片

重大疾病、传染病、遗传病、地方病等诊断用芯片，食品安全、生物安全检

测用芯片，研究用芯片，生物芯片数据获取、处理和分析设备及软件。

31、 生物材料及产品

利用生物质生产聚乳酸、聚羟基烷酸、聚氨基酸和聚有机酸等可降解材料， 生物可降解聚酯，可降解高分子材料与淀粉共混的环境友好材料，新型炭质吸附 材料，新型绿色生态可降解聚乳酸纤维、多元醇纤维，生物乙烯、 1,3-丙二醇、 丁醇系列产品，乳酸、丁二酸、琥珀酸以及各种具有特定性能的有机酸产品和医

药中间体。

32、 功能性食品

辅助降血脂、降血压、降血糖功能食品，抗氧化与抗缺氧功能食品，减肥功 能食品，特殊人群功能食品等，功能因子的绿色高效制备技术及生物活性稳态化

加工技术；功能性食品有效成分检测和安全评价技术。

三、航空航天

33、 民用飞机

先进大型客机；新型先进支线客机，现有支线客机的改进改型；民用直升机 和通用飞机（含无人驾驶飞行器），公务机、多用途通用飞机和专业用小型、超 小型飞机（含无人驾驶飞机）；民用特种飞行器（含系留气球、飞艇）；综合航空 电子、环境控制、安全及救生三大机载系统，民用航空发动机及重要部件；民用

飞机及发动机标准件、工艺装备；训练用、工程用飞行模拟机系统，机场及地面

保障设备。

34、 空中交通管理系统

民用航空卫星通信、导航、监视及航空交通管理系统（CNS/ATM）管制工 作站系统、 CNS/ATM 网关系统、飞行流量管理系统和自动化管制系统等在内的 成套空中交通管理设备，空域设计与评估系统，航空电信网（ATN）处理系统， 自动终端信息服务（D-ATIS）系统，空中交通进离港排序辅助决策系统，空管 监视数据融合处理系统，飞行计划集成系统，卫星导航地面增强系统，场面监视 系统，自动相关监视系统和多点相关定位系统，空域预警光电搜索跟踪系统，远

程大范围视频智能监控系统。

35、 新一代民用航空运输系统

行业综合性公共信息网络平台，安全管理系统，航空气象立体探测系统与客 观预报系统，航空气象四维资料共享平台与决策支持系统，适航审定系统，机场

安全检查系统，机场运行保障系统，大流量行李分拣及传送系统。

36、 卫星通信应用系统

通信卫星地面用户终端、便携式多媒体终端、卫星地面上行系统、卫星地面 差放站以及采用卫星通信新技术（新协议）的高性价比地面通信系统，低轨数据 采集卫星应用终端、应急减灾卫星通信系统，宽带/高频/激光卫星通信系统；C、 Ku 、Ka 及 L 波段的转发器，与卫星固定通信业务、卫星移动通信业务、电视卫 星直播业务（卫星数字音频广播）和互联网宽带接入等四大业务相关的地面终端 设备及其关键配套件；移动中卫星通信设备；基于卫星直播技术的数字内容投递

服务、天地一体化信息网络服务。

37、 卫星导航应用服务系统

卫星导航多模增强应用服务系统（含连续观测网络、实时通信网络、数据处 理中心和公共服务平台），基于北斗兼容型多模卫星导航芯片、个人移动信息终 端 SOC 芯片、 RNSS 授时接收机，基于位置信息的综合服务系统及其应用服务 终端（与无线通信网络结合的全球导航卫星系统技术和室内定位技术），具有导 航、通信、视听等多种功能车辆、船舶信息系统、个人导航信息终端，兼容型卫 星导航接收机，卫星导航用芯片和嵌入式软件，基于 BD-2 的气象测风终端、高 现势性导航电子地图，卫星导航时间频率原子钟；卫星导航高精度测地应用平台，

导航、位置（三维地理信息）与授时综合信息服务；卫星导航应用产业化标准体

系。

38、 卫星遥感应用系统

卫星遥感系统综合应用平台，形成基于自主数据源的高速全交换式地面接收 系统、基于网格架构的卫星遥感数据处理像素工厂、面向服务的分发系统、模式 类应用系统等共享平台，在国土测绘与监测、气象观测与服务、资源考察、城市 规划管理与监测、交通运输、农林监测、地质勘探、环境监测及防灾减灾等领域

的应用；城市空间信息服务；有效载荷国产化。

四、新材料

39、 纳米材料

纳米钨粉及纳米硬质合金材料、纳米膜材料、纳米催化材料和纳米晶金属材 料，材料表面纳米化技术，纳米能源材料与技术，纳米生物医用材料与技术，包 括重大疾病早期诊断与治疗用纳米材料与器件，纳米环境材料与技术，纳米多孔 气凝胶材料，纳米电子、光子、传感材料及器件，纳米材料与器件的制备、加工、

计量、评价技术与装备。

40、 高性能、低成本钢铁材料

超细组织钢铁材料的轧制工艺、先进微合金化、高均质连铸坯及高洁净的冶 炼工艺，高强度耐热合金钢及铸锻工艺和焊接技术，高强度轿车用钢、超超临界 机组用钢、高性能工模具钢、960MPa 以上高韧性工程机械用钢、耐腐蚀及耐高 温、高压高强钢，经济型奥氏体及铁素体不锈钢，高质量大型轴承钢，高速铁路

用钢，特殊品质高级无缝管。

41、 高性能镁、铝、钛合金材料

高性能铝合金、镁合金、钛合金、钨合金及其复合材料，钛合金及铝合金大 型宽厚板，镁及镁合金的液态铸轧技术，镁、铝、钛、钨合金的线、棒、板、带、 薄板、铸件、锻件、异型材等系列化产品的加工与焊接技术，大型复杂构件成形

技术，着色、防腐技术及相关配套设备。

42、 特种功能材料

特种功能焊接材料、特种功能喷涂材料、特种功能密封材料、超导材料，智 能材料，功能陶瓷、功能薄膜，气敏、湿敏、磁性液体、光敏材料、巨磁阻抗等

传感材料，氢的制备及分离、储氢合金和储氢容器、太阳能电池、高性能二次锂

电池和新型电容器等能量转换和储能材料，烯烃等聚合物及清洁生产所需催化材 料，稀贵金属高纯材料，非晶材料，特种阳极材料，稀有金属粉末及制品，多孔 材料及元器件，特种功能金属纤维及其制品，新型超硬材料及设备，贵金属催化

剂。

43、 稀土材料

高纯度稀土氧化物和稀土单质的分离、提取技术，高性能稀土（永）磁性材 料及其制品，稀土催化材料，稀土贮氢材料，稀土发光材料，稀土转换膜，超磁 致伸缩材料，稀土光导纤维，稀土激光晶体和玻璃,稀土精密陶瓷材料，高性能 稀土抛光材料，稀土磁光存储材料，稀土磁致冷材料，稀土生物功能材料，高性

能稀土合金材料。

44、 高温结构材料

陶瓷-金属复合材料，高温过滤及净化用多孔陶瓷材料，连续陶瓷纤维及其 复合材料，高性能、细晶氧化铝产品，低温烧结复相陶瓷、碳化硅陶瓷产品，单 晶高温合金低成本制备技术，TiAl 基和高熔点金属间化合物材料，粉末高温合金

成型产品、复杂高温合金铸件。

45、 新型建筑节能材料

高性能外墙自保温墙体材料、功能墙体材料、热反射涂料、相变储能材料、 外墙隔火防热材料，高效屋面保温材料，楼地面隔热保温材料，高性能节能玻璃

和门窗，低辐射玻璃。

46、 重交通道路沥青

利用环烷基原油资源生产重交通道路沥青，用重油和含硫原油生产高质量的 AH-70、AH-90 等牌号的重交通道路沥青，抗紫外线、防冻道路改性沥青，路面

再生及有机大分子废弃物在改性沥青中的应用。

47、 高分子材料及新型催化剂

新型工程塑料与塑料合金，新型特种工程塑料，阻燃改性塑料，通用塑料改 性技术，汽车轻量化热塑性复合材料，农林等纤维素原料提取高分子材料-酶解 木质素技术，氟塑料成形加工技术，聚烯烃催化剂、高效硝基苯加氢催化剂及原 位聚合聚烯烃纳米复合材料催化剂，交联聚乙烯材料和电器用合成树脂材料，高 性能聚芳醚酮类树脂材料，硅树脂、异戊橡胶、乙丙橡胶、硅橡胶材料及改性技

术，邻甲酚环氧树脂，万吨级聚碳酸酯塑料、千吨级尼龙 11 塑料、万吨级通信

和电力电缆用及油气输送用聚烯烃管材生产技术及设备，超低密度材料。

48、 复合材料

双金属材料及多金属复合材料，高性能铜合金复合材料，金属基复合材料， 碳-碳复合材料，陶瓷基复合材料，先进树脂基复合材料及其低成本制备技术， 新型特殊结构复合材料制备技术。绿色玻璃钢-热塑性复合材料制品，输气管道、

轴承、渔船、汽车覆盖件用玻璃钢。高强高导铜基纳米陶瓷弥散增强复合材料。

49、 特种纤维材料

高性能碳纤维、无碱玻璃纤维、氨纶纤维、芳纶纤维、芳砜纶纤维、超高分 子量聚乙烯纤维、聚苯硫醚纤维、聚四氟乙烯纤维、聚酞亚胺纤维，陶瓷纤维， 高性能、高感性、高功能和环保型纤维，晶须材料，低成本、高性能、特种用途

玻璃纤维及其制品。

50、 环境友好材料

生态环境材料，环境友好及特殊用途光学玻璃材料，环保型可降解塑料，建 筑与海洋防护用工程环保涂料，无机高分子絮凝剂，电子电器产品限用物质替代 材料，可降解汽车内饰材料技术，材料的可循环回收技术，高分子材料环境友好 技术，低碳型和环境友好型包装材料，建筑材料环境友好技术，环境友好材料的

分析检测技术和方法及标准物质。

51、 膜材料及组件

功能高分子膜材料及成套装置，均相系列荷电膜及装备，聚烯烃类微滤膜及 应用，纳米结构敏感膜、液体脱气膜、汽液相分离膜材料，模内转印（IMD）用 膜材料，氯碱用膜材料，高性能复合纳米滤膜材料，无机分离催化膜材料，生物 功能和仿生分离膜材料，海水、苦咸水及中水处理用反渗透膜材料及组件，陶瓷

分离膜材料与技术，渗透气化和蒸汽渗透分离膜材料与技术。

52、 金属粉体材料及粉末冶金技术

超高温、高压惰性气体雾化制粉技术，超声振动雾化制粉技术，注射成形、 温压成形、喷射成形等先进粉末冶金技术，系列化高性能粉末冶金产品，纳米粉

末冶金材料，低成本触点材料，复合粉体材料，高性能镍基高温合金粉体材料。

53、 表面涂、镀层材料

环保型防腐涂料，环保型高性能工业涂料，高温陶瓷涂敷材料，高档汽车用

金属颜料，水性重防腐涂料，耐高温抗强碱涂料，防火阻燃涂料，磁性热敏涂层

材料，先进高能束表面改性技术，复合表面技术，锡系无铅可焊性电沉积环保工

艺材料，超低表面能含氟表面保护材料与技术。

54、 盐湖及海水提锂、提镁技术

万吨级碳酸锂和高纯氯化锂技术，千吨级高纯度碳酸锂和单水氢氧化锂、万 吨级氧化镁和高纯金属锂，电解镁、高纯镁砂、高纯度无水氯化镁和氢氧化镁技 术，锂电池电解质、空调用溴化锂等相关产品，锂、钾盐精细加工工业过程二次

资源的综合回收利用，锂、镁盐产品的绿色过程优化集成系统和技术。

55、 新型纺织材料及印染后整理技术

新型合成纤维与纯棉、丝绸、麻、竹等天然纤维复合面料，天然纤维素的绿 色制浆技术和溶剂法纤维素纤维技术，新型纺丝技术，少水、少污染的清洁生产 技术，微悬浮体染色技术，可降解上浆剂，数字喷射印花技术和自动制网技术， 四分色印花技术，激光处理技术，等离子体处理技术，高附着力、高牢度的高档 染料，高效短流程染色技术及配套的活性染料和助剂，生物酶加工技术，多功能

染后整理技术，天然纤维织物的防皱整理技术以及环保型、功能性助染剂。

56、 高性能密封材料

轿车及中高档轻型车覆盖件、结构件及动力传动、减振、制动系统用密封材 料，大型成套设备高压、液压、气动系统用密封件，电力设备高温、高压机械用 密封件，石油化学工业用高速透平压缩机的非接触气膜密封件，金属磁流体密封 件，高性能无石棉密封材料，高性能碳石墨密封材料，高性能无压烧结碳化硅材

料，航空航天用聚硫密封剂材料。

57、 子午线轮胎生产技术和关键原材料

低碳、节能、安全、高性能子午线轮胎制造技术，异戊橡胶、杜仲橡胶生产 技术及装备，新型环保、节能、高性能纤维（金属）骨架材料， 5 万吨/年节能、 低耗、环保、高性能软质新工艺炭黑，高性能、低能耗特种炭黑，低耗、低排、

绿色、高性能橡胶助剂。

58、 金属、无机非金属多孔复合催化材料

能源工业净化燃煤烟气用金属催化过滤材料，多孔过滤催化材料，金属多孔 材料表面预处理技术，载体复合、催化剂活性组分附着等表面技术，金属复合催

化材料的制备技术，催化过滤材料的制备技术，催化反应膜技术。

59、 油田用助剂

万吨级耐高温、耐盐聚合物驱油剂，驱油表面活性剂，万吨级钻井液用化学 品，万吨级高效清防蜡剂和降凝降粘剂，千吨级高温原油破乳剂，千吨级石油压 裂液增稠剂、采油和炼油缓蚀剂，千吨级采油用稠油降粘剂，千吨级高效杀菌剂，

石油开采中的环境友好型高分子驱油材料，原油脱硫化氢剂。

60、 造纸用助剂

2 万吨/年造纸专用增强剂，万吨级涂布纸用专用化学品，万吨级造纸用树脂 障碍控制剂， 2 万吨/年高留着型淀粉表面施胶剂， 5 千吨级印刷适应性改进剂，

万吨级造纸增强填料石膏晶须产品，新型功能表面活性剂。

61、 新型选矿设备及药剂

铜矿、铁矿等大型金属矿山和铝土矿、钨矿、锡矿、钛矿及低品位的氧化锌 矿、锶矿等难处理矿成套选矿设备，大型选矿、冶炼自动控制技术与装备，千米 深井采矿技术与装备，大深度精细勘查技术与装备，数字矿山关键技术，高效低

毒的捕收剂、调整剂、起泡剂等选矿药剂。

62、 核工程用特种材料

高纯海绵锆及核级锆与锆合金、锆合金的表面改性，核级不锈钢，耐晶间腐 蚀和应力腐蚀的镍基合金，抗液体钠腐蚀材料，抗氢脆材料，抗高温热腐蚀低合 金钢，高纯、抗辐照各向同性石墨，中子屏蔽用石墨，耐腐蚀、抗辐照脆化、具 有良好焊接性能的高强度压力壳体钢，核二、核三级设备超厚超宽钢板和锻件，

安全运行监测控制用低熔点材料。

五、先进能源

63、 动力电池及储能电池

高性能锂离子电池正极材料、隔膜材料、电解质材料制备技术，大容量锂动 力电池成组技术与设备、电池管理系统设计与生产，大容量钠硫电池模块制备、 储能系统、电网接入系统与控制技术，全钒液流储能电池制备、电池系统设计、 集成与运行控制技术，质子交换膜燃料电池及关键材料制备技术，直接醇类燃料

电池，中低温固体氧化物燃料电池及微型燃料电池。

64、 氢开发与利用

高效天然气制氢、化工、冶金副产煤气制氢，低能耗电解水制氢，生物质制

氢、微生物制氢技术，高压容器贮氢、金属贮氢、化合物贮氢技术，氢加注设备

和加氢站技术，超高纯度氢的制备技术，氢燃料发动机与发电系统技术。

65、 风能

兆瓦级以上风电机组关键零、部件技术，风电逆变系统的数字化实时控制技 术，保护检测技术，风能监测与应用技术及装备，风电储能及电网稳定技术与设 备，海上风电机组基础及安装技术和风电场运维管理运行技术，海上风电机组及

核心零部件设计、制造技术。

66、 太阳能

高效率、低成本、新型太阳能光伏电池材料，太阳能晶硅冶炼用长寿命石墨 材料，太阳能电池制造技术及装备，太阳能电池非玻璃封装技术，中、高温太阳 能发电技术与设备，太阳能储热材料，光伏逆变并网系统技术，兆瓦级以上光伏 太阳能并网发电系统，兆瓦级以上大规模太阳能高温热发电系统，风/光及其他 能源互补发电系统，太阳能采暖与制冷系统与设备，太阳能与建筑一体化技术，

薄膜太阳电池关键技术及装备，聚光、柔性等新型太阳电池技术及装备。

67、 生物质能

非粮作物生物燃料乙醇及副产品联产技术，农林生物质能源原料新品种及其 配套生产技术，农业废弃物生产高值生物燃气技术，绿色生物柴油精制技术，生 物质热解、气化燃料技术，生物航煤生产技术，生物质直燃、混燃和气化供热/ 发电技术，生物质气化制氢技术，生物质成型燃料生产、应用及系统集成技术， 垃圾、垃圾填埋气和沼气发电技术，油料植物的高附加值利用技术，秸杆、芦苇、

麻类高效降解与转化技术和设备，二氧化碳（CO2 ）藻类转化技术。

68、 地热能与海洋能

水源、地源、空气源热泵与采暖、空调、热水联供系统技术，高温地热勘探、 开发与利用技术及装备，深层干热岩发电技术，潮汐发电、波浪发电、海流发电

并网技术及装备。

69、 石油勘探开发技术及设备

山地、沙漠、滩海、浅海和深海等复杂地区地球物理勘探技术，单点数字检 波器技术，高密度、多波、时移地震技术，高精度电磁波勘探技术，地球物理采 集、处理、解释软件系统，基于 GPU/CPU 协同并行地震处理系统，大型地震仪，

可控震源，高性能数控、成像测井技术及装备，井间电磁波和声波测井技术，油

藏平价随钻具或过钻具测井技术，特殊水平井、全过程欠平衡等钻井综合配套技 术，地质导向钻井技术及装备，旋转地质导向钻井技术及设备，复杂结构井开发 油气藏技术，复杂深井钻井技术及装备，气体钻井技术及装备，膨胀管技术及装 备，大型压裂装备，凝析油气田、稠油、超稠油开发技术、三次采油复合驱工业

化应用配套技术及装备， CO2 强化驱油技术及设备，高含硫气田开发技术。

70、 油品加工技术及设备

加氢裂化催化剂和相关技术，劣质原油和渣油加氢技术，催化裂化原料预加 氢技术，煤液化油加氢提质技术，费-托合成油加氢改质技术，特种油品的加氢 技术，电脱盐、常减压蒸馏等一次加工技术，催化裂化、焦化、重整、异构化、 烷基化、S-Zorb 等二次加工技术，油品精制技术，润滑油加氢技术，生产超清洁

汽柴油的油品加氢技术，油浆、石油焦的加工利用技术。

71、 长距离高压油气输送设备

输送压力 10MPa 以上的输气设备，钢材、管材及施工机具的制造，管道监 测与监测机器人，网络监测系统、控制系统和安全维护装备，天然气脱硫、 脱 CO2 、脱水技术及设备，富气密相管道输送技术，液化天然气（LNG）及再气化 技术与装备，稠油长距离管道输送技术，多种油品或原油顺序输送工艺及配套技

术，油气管道破损检测及堵漏技术。

72、 煤炭高效安全生产、开发与转化利用

煤矿地质与资源条件适用型成套生产装备，大型矿井支护、采掘设备及自动 化控制，短壁采煤技术，高效分选、配煤装备，水煤浆专用设备及高性能添加剂， 大型微泡浮选柱、煤泥水高效澄清及控制技术，型煤加工及利用设备，煤矿瓦斯 高效抽采技术及设备，煤矿用高性能抢险救灾装备，煤矿全矿井安全监控与预警 系统，高精度煤田地质地球物理探测设备和煤矿地质灾害勘探技术装备，高效益、 低成本的煤层气勘探技术，煤层气规模开发与采煤一体化技术，煤层气井上、井 下联合抽采技术与装备，大型煤炭气化及煤基多联产系统装备技术，煤整体汽化 联合循环技术（IGCC），高参数超超临界发电技术，煤炭（直接、间接）液化技

术，煤制天然气技术，合成气制甲醇、制乙二醇技术，甲醇制低碳烯烃技术。

73、 高效低污染燃煤发电及水电技术系统

300MW、600MW 级超临界循环流化床锅炉及辅助设备，低氮氧化物燃烧技

术，高效超超临界燃煤发电技术，100MW～200MW 级燃用中低热值煤气和高氢

燃料燃气轮机及相关关键技术，复杂条件水电站开发关键技术。

74、 核电及核燃料循环

百万千瓦级先进压水堆核电站关键技术与成套设备，铀纯化转化、铀矿勘查 和采冶、铀浓缩技术及关键设备，高性能燃料元件，铀钚混合氧化物燃料，先进 乏燃料后处理技术，核辐射安全与监测技术，核设施退役与放射性废物处理和处 置技术，快中子堆和高温气冷堆核电站技术及设备，模块化小型核能装置，核应

急技术。

75、 电网输送及安全保障技术

复杂环境地区电网电气安全运行新技术，大型变压器，直流换流变压器，开 关设备和电抗器，无功补偿设备，柔性输电系统及设备，变电站及电气设备的智 能化，电子式互感器及核心元器件，状态评估及诊断装置，500 千伏以上直流输 电技术及设备， 1000 千伏交流长距离输电技术及设备，环保绝缘材料输变电设 备，超大规模电网安全保障和防御体系及智能调度技术，可再生能源规模化及高 密度多接入点分布式电源并网及控制技术，智能配电、用电技术，电动汽车充电 设施与电网协调运行技术，电网与用户互动技术，安全高效施工技术及设备，电

网环保与节能技术及设备，大规模储能系统。

六、现代农业

76、 农作物新品种

应用基因工程、分子育种、细胞工程、染色体工程、航天辐射工程和杂种优 势利用工程等技术选育的高产、优质、高效、抗除草剂、抗病虫、抗逆的新品种， 特色、名优、专用和功能性新品种，大宗粮棉油糖烟作物优良品种繁育和检测技

术，水肥资源高效利用型新品种，草地、牧草新品种及其繁育技术。

77、 蔬菜、水果等园艺作物新品种

优质、耐贮运蔬菜专用品种，耐低温弱光、抗多种病害瓜菜、水果专用品种， 优质、高产高山冷凉蔬菜新品种，适合旱区栽培的水肥资源高效利用型蔬菜优良 品种，含有特殊功能成分的高营养性蔬菜、瓜果品种，果树矮化砧木及良种苗木

繁育技术，名优水果绿色安全生产与经营技术（IFP 、GAP 以及有机果园）。

78、 林木、花卉新品种

用材林、能源林、经济林树种及新品种，特有或珍稀种质资源、速生丰产优

质用材林、生态保护与城市绿化种/苗，名贵花卉新品种及组织快繁技术，球根

花卉种球繁育技术。

79、 畜禽水产新品种

高效、优质、抗逆、资源高效利用的畜禽、水产、特种经济动物新品种及生 物工程快速育种与繁殖技术；转基因动物与生物反应器生产技术，地方品种、珍 稀畜禽、重要经济鱼类、特种动物、资源昆虫等珍惜资源的挖掘、保存、创新与

综合利用。

80、 新型设施农业技术

节能日光温室及连栋温室标准化建造技术，设施配套装备和环境测控技术， 设施专用农林作物新品种及其高效节能配套栽培技术（包括高效设施无土栽培技 术、病虫害综合防治技术、节水灌溉技术与设备等），高效节能植物工厂及其配

套装备。

81、 安全高效、规模化畜禽清洁养殖技术

新型节耗成套畜禽标准化清洁生产养殖工艺和设备，畜禽舍与环境净化成套 设备，新型节耗、减排添加剂及综合配套技术，农业废弃物无害化处理与资源化 利用技术与装备，畜禽标准化健康养殖产业模式，精准饲养与全程管理数字化技 术，草食家畜高效舍饲及补饲育肥技术，特色畜禽高效养殖技术及品牌化经营，

新型饲草产品生产技术及其设备，草原（地）生态保护、虫鼠害防治技术。

82、 安全高效淡水产品清洁养殖技术

经济适用的工厂化循环水养殖技术及设备，池塘生态工程化、标准化养殖技 术及设备，封闭型水生态维持系统及设施工程，鱼塘、陆基、滩涂集约式高效低

排放养殖设施及生物治理综合技术。

83、 农林节水技术与设备

低成本、智能型高效节水灌溉关键技术及设备，低水头、智能型田间量配水 技术与设备，移动机组式滴灌技术与装备，山区林果地表控制微灌技术及设备， 田间自控式地下滴灌系统，自压软管灌溉及配套技术，移动式小型抗旱灌溉系统， 小型低水头大流量水泵，高效环保节水生化制剂，新型环保覆盖材料，雨养农区

雨水与径流集汇技术与装备。

84、 新型高效生物肥料

区域型工农业废弃物、城市生活垃圾治理及生产高效多功能生物肥料技术，

快速腐熟秸秆生物技术，高效溶磷生物肥料技术，耐铵固氮生物肥料技术，高效

解钾生物肥料技术，长残留除草剂降解生物肥料技术，土壤保水抗旱生物肥料技 术，连作障碍防治生物肥料技术，高效载体灭菌技术与装备，生物肥料保活材料 技术，草炭载体的替代技术，火山灰改良土壤技术，肥料缓释技术，果园专用肥

料及其使用技术。

85、 新型安全饲料

饲用氨基酸添加剂、酶制剂、微生态制剂、植物提取添加剂、生物活性肽及 抗菌肽、高活性生物发酵饲料、幼龄动物专用饲料、风味饲料添加剂生产技术及 设备，高效渔用饲料配制技术及动物性饲料源替代技术，补充性饲料及低营养水

平饲料配制及投喂技术。

86、 农业动物重大疫病预防控制

预防控制禽流感、口蹄疫、狂犬病、猪蓝耳病、新城疫、布氏杆菌病等重大 动物疫病及人兽共患病的新型疫苗、诊断试剂、快速检测试剂盒、免疫增强剂以 及消毒药物等，动物源性食品安全相关微生物的检测与风险分析技术，经济动物 和野生动物疫源疫病监测技术，环境融合型畜禽疫病综合防治技术，高效、低毒、 使用安全的新型兽医专用药物、中兽药（制剂）生产技术、兽用标准物质制备技 术，兽药多残留快速检测技术、耐药性监控技术，佐剂、悬浮培养、发酵培养等

兽用生物制品产业化关键技术。

87、 水产疫病预防控制

水产养殖生物主要疫病快速诊断与检疫试剂盒、免疫增强剂、抗病微生物制 剂、病原感染阻断剂、非特异性免疫制剂，口服、浸泡型和注射型渔用疫苗，高

效、低残留渔用药物新剂型、新制剂，禁用渔药替代药物。

88、 农林植物有害生物检疫、预防、控制

高效、低毒、低残留、环保型农药新产品（制剂）生产技术，智能施药、施 肥技术及产品，农药残留快速检测技术及设备，农林作物有害生物及外来入侵生 物检疫、监测、检测、快速鉴定、预警、预防和长效控制技术与产品，物种资源 出入境快速查验及快速鉴定技术及装备，检疫检验隔离新技术与设施，食品中有

毒、有害物质检疫检验、预防和控制技术与装备。

89、 数字化农林技术与装备

农林生产的专家智能决策系统、农林科技服务“ 110 ”系统、农林业信息服

务云计算系统、农林科技服务业多媒体与 3G 技术，农业水土生物资源时空分布

数字化技术，数字农林资源与环境监测无线传感与成像光谱技术，农林灾害监测 预报、森林生物量与碳汇计量、作物估产、粮食安全预警及宏观决策等领域配套 应用的信息技术及装备，农林产品质量标准及安全生产全程质量监控与追溯信息 系统，农林机械总线控制系统、智能化农机，基于“多网融合 ”的农业综合信息

服务系统和低成本多功能信息服务终端，农业物联网技术与产品。

90、 农林产品加工技术与装备

农林产品精深加工与综合利用技术及设备，现代生物工程技术在农林产品加 工中的应用及设备，食品非热保鲜加工、高效分离提取、质构重组和高效节能干 燥技术与装备，食品包装新材料与新设备，基于减少营养损失和提高品质的果蔬 加工新技术，果蔬加工中的节能减排技术，天然橡胶标准胶节能清洁生产技术， 环氧化高性能轮胎专用天然橡胶技工技术，食品加工数字化监控与全程质量控制 技术与设备，农林产品产地加工、产品保鲜、贮运包装技术与成套设备，农林副 产品及加工副产物资源化高效利用与清洁生产技术及设备，农林产品新型功能性 食品生产技术及设备，新型方便食品的生产技术及设备，非木质林产品精深加工 生产技术，木材高效加工技术，木本粮油精深加工技术，农林特产资源精深加工

技术与装备。

91、 农林业生物质材料精深加工与利用

新型木基复合工程材料的生产技术，实体木材的功能改进和深加工，新型结 构用建材的生产技术，结构用木材单板层积材生产工艺、设备技术改造和在线检 测控制技术；竹纤维提取与加工，重组装饰材及薄木生产技术，竹材精深加工和 设备技术改造及其在线检测控制技术；结构用木质材料标准体系建设；特种功能 木质活性炭清洁生产技术；生物质基功能高分子新材料生产技术；木材和人造板 节能减排生产技术，木竹制品有害物质检测控制技术；渣纤维提取与加工，重组 装饰材料及高性能甘蔗渣基木塑复合材料生产技术，甘蔗渣精深加工和设备技术

改造及其在线检测控制技术。

92、 农林业机械

100 马力以上大型拖拉机及配套农机具，50-80 马力节能环保型水田拖拉机， 高地隙拖拉机，多功能高效谷物、玉米、甘蔗收获机，高效植保机械，高效节能

机泵设备，精确施肥滴灌技术装备，保护性耕作和播种复式作业机具，丘陵山地

小型耕、种、收机械，水稻高效精密低损伤栽插成套技术设备及半喂入水稻收割

机，油菜、棉花、茶叶、烟草、蔬菜全程机械化生产关键技术与装备，花生、马 铃薯、大蒜等根茎类种植与收获机械，养殖环境控制与疫病防治机械，名优绿茶 智能机械化加工关键技术与装备，农作物秸秆收集与贮运装备，移动式生物质成 型燃料制造成套设备，造林抚育机械，森林防火与病虫害防治专用器械，高效节

能降耗磨浆系统，农林产品冷链物流装备。

93、 粮食储藏与流通

现代粮食输送流通成套技术装备，粮食收购一体化快速检测技术，粮食节能 干燥技术，粮食储藏品质监测技术，粮食储藏品质控制与处理技术，数字化粮食

储藏技术，粮食绿色防护剂技术。

七、先进制造

94、 工业自动化

大型火电、石化、冶金、核电工程所需综合自动化系统，应用现场总线技术 的检测与控制仪表，高性能智能化控制器，大型传动装置用高效、节能调速系统， 数字化、智能化变送器和传感器，现场总线与无线网络集成的各种软件及硬件产 品，智能化工业控制部件、控制器和执行机构，自动化测量仪表，工业无线控制、

功能安全控制系统和设备。

95、 网络化制造

企业资源信息网及相应的企业核心信息、产品、图形语义和技术资源等分布 式数据库，支持网络化制造的 Web 运行平台，具有行业和专业特点的网络化制

造示范系统。

96、 现代科学仪器设备

近红外光谱仪、等离子体光谱仪、金属原位分析仪、辉光光谱仪、激光光谱 仪等光谱分析仪器，气相色谱仪、液相色谱仪等色谱分析仪器，等离子体质谱仪、 质谱联用仪等质谱分析仪器，新型 pH 计、电导仪、离子计、电位滴定仪等电化 学分析仪器，微机控制材料试验机、材料图像分析仪、智能化电磁超声探伤仪等 材料性能检测仪器，高性能工业 X 射线 CT 装置，环境保护、社会安全应急检测 仪器和系统，全自动气象测量系统，二维色谱、阵列毛细管电泳、表面等离子体

共振成像、激光诱导荧光等医学研究仪器，基于光纤传感技术的结构健康监测系

统。

97、 新型传感器

高性能、多功能的位移、力敏、磁敏、光敏、热敏、气敏、湿敏、离子敏和 生物敏型传感器以及红外传感器、光纤传感器，紫外传感器，声表面波传感器， 微纳传感器，生物、医学研究急需的新型传感器，新型环保、气象、地震、海洋、 大气环境监测传感器，工业过程控制传感器，汽车传感器，多传感器的集成与融

合技术，结构健康监测传感器、腐蚀监测传感器。

98、 精密高效和成形设备

精密微细加工技术，特殊用途光学薄膜加工技术及设备，近净成形技术与装 备，纳米精度高效光学加工技术及设备，大型数控锻压机床及生产线，高精度大 型复合材料缠绕、铺带、铺丝设备及相关工艺过程分析、模拟和优化软件，高精

度塑料加工成形设备。

99、 激光加工技术及设备

性能稳定的大功率激光器及其晶体，大功率光纤激光器，大型轧辊激光表面 强化设备，激光精密加工技术和设备，激光切割技术和设备，激光焊接技术和设 备，激光热处理和熔覆技术及设备，激光强化技术和装备，激光复合加工技术和

装备，激光加工基础装置和系统，激光测量仪器和校准标准仪器。

100、 高精度数控机床及功能部件

高精密车、铣数控机床及加工中心，车铣（铣车）复合机床，高精度数控磨 床，数控齿轮加工机床，重型、超重型数控机床，数控特种加工机床，数控专用 机床及生产线，中高档数控系统和数字伺服控制器，大功率、高刚度电主轴及其 伺服单元，直线电机、力矩电机及伺服控制器，高速滚珠丝杠副和导轨副，高速、 精密、重载直线导轨，万能铣头，高速防护装置，刀库及自动换刀装置，全功能

数控刀架、数控回转工作台，高精度数字化测量仪器，高速切削刀具。

101、 机器人

新型工业机器人及其在自动化生产线的应用，面向危险作业环境的应急救 援、灭火、深海作业等机器人，面向人类健康的医疗机器人，面向社会公众的家 用机器人、保安机器人、教育和娱乐机器人，面向残障人员、无障碍行动的辅助

机器人。

102、 大型石油化工成套设备

重质、含酸、含硫原油加工成套技术与设备、百万吨级乙烯成套系统设计制 造技术，精对苯二甲酸（PTA）成套系统设计制造技术，乙烯裂解炉，“三机 ”、 冷箱、氢气压缩机、空气压缩机组、氧化反应器、回转干燥机、真空过滤机等关

键设备的设计制造技术，煤化工成套设备设计制造技术。

103、 关键机械基础件

轿车三代轮毂轴承单元，冶金矿山设备重载荷专用轴承，数控机床用精密轴 承，航空航天特殊轴承，大功率风力发电机增速器、发电机主轴轴承，长寿命工 程机械轴承；工程机械用大流量高压柱塞泵/马达、高压液压阀，航空、船舶用 比例、伺服元件和系统，高频电液伺服阀和比例阀，液力变矩器，核电站用核级 泵和阀门，轨道交通用高可靠性气动元件及系统；清洁高效发电设备用关键密封， 盾构机主轴密封；兆瓦级风电齿轮箱，大模数齿轮齿条传动装置，轨道交通专用 齿轮箱，高强度紧固件；高应力弹簧；汽车覆盖件模具，多功能级进模，大型精

密塑料模具，压铸模具。

104、 电力电子器件及变流装置

电机节能高压变频装置，大功率高端电机调速系统；新能源风电、光伏、储 能等并网逆变器和高压直流输变电交流器，新型动态无功补偿及谐波治理装置， 高精度、高性能的开关电源及不间断电源；轨道交通电气化、船舶推进、电动汽

车用变流器及驱动装置。

105、 汽车关键零部件

无级变速器、自动变速器、电动转向装置、主动（半主动）悬架系统，防抱 死制动系统/牵引控制系统/电子稳定装置，环保薄膜安全气囊，铝车身及零部件， 智能前灯和中央照明系统，载重车用盘式制动器，商用车与越野车用空气悬架； 启动/发电机/飞轮一体电机，驱动电机； 42V 电源系统，电子控制系统，混合动 力汽车动力总成，环保冷酶汽车空调压缩机，汽车尾气处理系统及控制模块；商

用车无内胎车轮，汽车再制造技术。

106、 高效节能内燃机

满足国家标准排放和节能的汽车用柴油机、汽油机，船舶、铁路机车、农业 机械、工程机械、核电等配套用新一代柴油机，汽车配套用各种代用燃料发动机、

混合动力车用发动机，电控直列式喷油泵、电控高压共轨喷射系统、电控高压单

体泵以及各种新型喷油器、喷油嘴。

107、 数字化专用设备

平张纸多色高速胶印机、喷墨数字印刷机，卫星式柔板印刷机，高速卷筒纸 胶印机、多色凹版印刷机，智能化多色双面印刷设备，基于光刻技术的压印设备， 计算机 CTP 直接制版技术与设备，基于纳米材料的绿色制版技术及设备，数字 化电子轴传动技术；数字式扫描制版打印一体化速印机，日产 200 吨及以上涤纶 短纤维成套设备，高速粘胶长丝连续纺织机，剑杆织机和喷气织机，节能、环保

染整设备、数字化喷印设备。

108、 快速制造技术及设备

激光快速原型成形机、紫外光固化成型机、三维打印机等激光快速成形技术 和设备；多点数字化成形技术与装备，板材逐渐成形技术与装备，基于快速成型 （RP）的铸造法直接制造金属零件的技术与装备，直接制作功能零件的技术与

装备。

109、 大型构件制造技术及装备

重大装备中大型构件的冶炼、铸造、锻压、焊接、轧制、热处理及表面处理 技术与装备，大型发电机组及民用航空喷气推进发动机等高效节能涡轮发动机组

制造技术，大型构件热加工工艺模拟技术。

110、 大型部件自动化柔性装配技术及装备

面向数字化装配的结构设计、数字标工与数字量协调技术，数字化装配生产 线，装配变形误差分析与容差分配技术，数字化装配仿真平台，数字化自动定位

设备，数字化自动化制孔设备，柔性工装。

111、 核技术应用

辐照交联电线电缆、热缩材料、辐照材料、发泡材料、交联聚烯烃管材及附 件、橡胶硫化、高分子 PIC 器件、绿色环保涂料，用辐射技术处理三废，电子束 固化等辐射加工，医疗保健用品辐射灭菌消毒，同位素辐照设备、大功率辐照加 速器、电子加速器及成套设备等辐射装置及成套设备，以同位素 γ源和加速器为

射线源的大型工业在线检测、危险物品的安全检测装备，同位素药物及辐射治疗。

112、 高技术船舶

30 万吨以上超大型矿砂船和原油船、超大型集装箱船、液化石油气船、液

化天然气船、化学品船、滚装船、大型疏浚船舶、冰区级船舶、豪华游艇及游船，

全铝合金海峡运输船、海洋资源考察船、大型物探船、测量船、超大型半潜式运

输船，大型船用柴油机、大型船舶动力系统、电力推进装置、大型甲板机械和舱

室机械、导航和自动化装置。

113、 海洋工程装备

大型自升式钻井平台、半潜式平台、TPL 平台、SPAR 平台，钻井船，大型 起重兼铺管船、超大型浮式生产储油装置，海洋平台中高压电站、平台升级及锁 紧装置，海洋平台钻井模块及多功能深井钻机，海上稠油及边际油田开发装置， 深水水下动力定位及采收系统，无人遥控潜器、载人潜器，海洋工程作业船及辅

助船。

114、 轨道交通设备

350km/h 及以上高速列车成套关键技术与设备，200km/h 及以上动力分散式 交流传动动车组，200km/h 及以上交流传动客运电力机车，160km/h 大功率交流 传动货运电力机车，城市轨道交通车辆、大功率交流传动内燃机，交流电传动及 其控制系统，机车、地铁网络控制及信号系统，高速铁路通信信号、牵引供电、 列车控制、客运服务、防灾系统，高速轨道交通安全监测系统，高速铁路、城市 轨道交通维修养护成套设备，路基/轨道/车辆姿态监控，重载铁路建设关键设备，

中低速磁悬浮交通车辆、牵引供电系统、运行控制系统。

八、节能环保和资源综合利用

115、 先进节能技术

燃煤工业炉窑改造技术，节约和替代石油技术，流程工业能量系统优化技术 与装备，工业余热余压利用技术，能量转换系统效能提高及改造技术，能量梯级 利用技术，仿真节能控制技术，半导体照明与照明节能控制技术，建筑节能及节

能改造技术，电机系统节能控制及改造技术，热电冷等联产联供技术。

116、 饮用水安全保障技术

灵敏、快速的水源地水质自动监测技术，水质在线检测和预警技术，饮用水 强化处理技术，高效安全消毒技术，微污染净化技术，高效控藻、除藻和藻毒素 去除技术，管网水质在线检测技术，多功能自动化捞藻船及二次污染控制技术， 多物种智能生物预警仪，管网水质稳定技术和直饮水净化技术，农村饮用水除氟、

除砷技术与装置。

117、 工业和城市节水、废水处理

洗涤等废水循环利用技术及装备，供水管网防漏技术，高浓度有毒工业废水 处理技术和设备，石油废水处理与分质回用技术，高效水处理药剂的研制与开发， 工业、污泥安全处置与资源化技术，高含盐废水处理工艺与技术，城市污水、工

业废水深度处理及资源化再生利用技术。

118、 雨水、海水、苦咸水利用

雨水收集利用与回渗技术与装置，海水、苦咸水淡化技术与装备，海水膜法 低成本淡化技术及关键材料，规模化海水淡化热能设备和海水淡化设备，海水、 卤水直接利用及综合利用技术与装备，耦合海水淡化技术，浓盐水浓缩结晶零排

放技术与装备。

119、 大气污染与温室气体排放控制

机动车尾气排放控制用高性能蜂窝载体、满足国 IV、国 V 标准汽车净化器， 高性能除尘滤料和高性能电、袋组合式除尘技术与设备，燃煤烟气脱硫、脱硝、 脱汞或一体化的高效技术和装备，工业排放有毒废气控制技术与设备，选择性催 化还原法（SCR）烟气脱硝催化剂及再生技术，室内空气污染物控制与削减技术，

挥发性有机化合物（VOC）的控制技术，油库、加油站油气回收技术与设备，

碳减排及碳转化利用技术，消耗臭氧层物质的低温室潜能替代技术及产品。

120、 固体废弃物的资源综合利用

垃圾分选、破碎、生化脱水等预处理和综合处理技术与装备，城市及农林固 体废弃物处置及能源利用技术，厨余垃圾处理技术与配套设备，利用工业固体废 弃物生产复合材料、工程结构制品等技术及设备，电厂粉煤灰及煤矿矸石、冶金 废渣、低品位矿及尾矿废渣、建筑废弃物等资源回收与综合利用技术，废旧家电 与电子产品、汽车等拆解、废弃物资源化处理成套设备，矿山尾矿资源生态型管

理与综合利用技术，贵金属资源二次高效回收利用技术。

121、 危险固体废弃物处置技术及设备

危险废物高效、安全、可靠的收集、存储、运输与焚烧技术及设备，焚烧渣、 飞灰熔融无害化等处置技术和设备，危险废物安全填埋处置技术及设备，危险废 物固化技术、设备和固化药剂，医疗废物收运、高温消毒处理技术与设备，有害 化学品处理技术，放射性废物处理与整备技术与装备，危险废物污染事故应急处

理设备，电池回收和再利用技术及设备，废旧荧光灯管汞回收处理技术（MRT)

及装备，利用水泥窑处置危险废弃物技术及装备。

122、 环境自动监测系统

水质及污染源在线监测系统备，水中微量有机污染物富集装置，持久性有机 污染物采样、分析系统，环境遥感监测系统和量值溯源标准设备，空气质量及污 染源在线监测系统，温室气体（GHG）排放监测技术与设备，污染事故应急监 测等便携式现场快速测定仪及预警、警报仪器，大气中污染物在线检测系统，矿 山安全监测、预警与防治技术，滑坡、崩塌、泥石流等地质灾害监控预警设备及

系统。

123、 生态环境建设与保护

环保基础材料制备及其应用技术，水土流失及荒漠化防治技术，湿地恢复与 利用技术，污染土壤修复、污染水体修复、衬泥治理及富营养化防治技术，面源 污染控制技术，持久性有机污染物（POPs）替代技术及替代产品，重金属污染 物农田治理改造技术与产品，垃圾填埋防渗材料、渗滤液处理、填埋气回收技术 和设备，高效、节能、环保和可循环的新型制造工艺及装备，机电产品表面修复

和再制造技术。

九、海洋

124、 海洋监测技术与装备

特异、灵敏度高、抗污染、抗海洋生物附着和耐腐蚀的各类传感器，适合海 洋动力和生态环境现场连续快速测量的浮标、潜标、海床基、岸基及智能化走航 的平台技术，适应海上固定平台及船舶观测相关技术，经济型投弃式测量装备， 海洋环境探测雷达技术及装备，海洋环境声学探测技术及装备，海洋突发性污损 灾害事故应急监测等便携式现场快速测定仪和预警、警报专用技术及系统平台， 海洋地震观测技术设备，海洋遥感技术，水下通讯和能源补充技术，海洋信息处

理和应用技术。

125、 海洋生物活性物质及生物制品

源于海洋生物的抗菌、抗病毒、抗肿瘤、抗氧化、抗骨关节病、降血糖、减 肥及心脑血管、神经系统等高效海洋新药物规模提取、纯化和合成，应用现代生 物技术从海洋生物中获取海洋功能食品、酶制剂以及特异性诊断试剂等生物制

品，大型藻类生物酿造、生物能源技术开发。

126、 海水养殖良种繁育和育苗技术

海洋生物优良种质挖掘与创制、及规模化繁育，海水养殖动物细胞工程和性 控制技术育种、育苗和大规模海水养殖技术，海水养殖植物细胞工程育苗、育种 技术，滩涂耐盐蔬菜、优良生物材质的规模培育与栽培技术，海水养殖病害控制 技术，海洋生物资源养护与环境修复技术，基于生态工程的海洋牧场构建与海珍

品养殖技术。

127、 设施渔业和渔业工程装备

远洋捕捞作业装备和选择性助渔仪器及设备；深水养殖用抗风浪网箱设施、 配套设备及养殖技术，陆基集约式节能减排和工厂化循环水设施和养殖技术，池

塘、滩涂等高效养殖和资源综合利用设备和技术。

128、 海底资源环境监测、勘探技术与装备

海底资源勘探、采样和评价技术与装备，水下组网技术，水下移动观测平台 技术，海底极端环境监测、探查技术与装备，深海观察及运载技术与装备，海洋

勘探、开采的防污与封闭装备。

129、 海洋环境保护与生态修复技术及装备

海洋环境污染防治与处理技术及装备，海洋环境污染处理材料与制剂，海洋

生态系统功能修复与恢复技术。

十、高技术服务

130、 信息技术服务

信息技术咨询服务，信息系统工程监理服务，信息系统设计服务、集成实施 服务等信息系统集成服务；信息系统托管服务；数据挖掘与管理服务， SaaS（软 件即服务）、PaaS（平台及服务）和 IaaS（基础设施即服务）等云计算服务，面 向应用的高性能计算机软件研发和服务业务。数据恢复和灾备服务，信息安全防 护、网络安全应急支援服务，云计算安全服务，信息安全风险评估与咨询服务， 信息装备和软件安全评测服务，密码技术产品测试服务，信息系统等级保护安全 方案设计服务。软件评测服务。基于固定宽带互联网、移动互联网的业务，IPv6 商业化应用；三网融合应用服务，网络电视、手机电视、数字电视宽带上网等服 务；基于宽带网络的信息增值服务；基于物联网技术等的智能城市管理、智能环

保、智能交通等信息服务解决方案及服务平台。

131、 电子商务服务

面向行业、区域、企业及消费者的网络交易服务，应用具有自主知识产权的 加密和认证技术的电子认证服务，在线支付服务，物流配送信息服务；网络信用 信息及评估服务；网络身份管理与验证服务；网络信息管理与检测服务；网络交

易安全保障服务；网络维权服务；电子商务系统技术支持服务。

132、 数字内容服务

基于三网融合的数字内容播控平台与集成分发服务体系，移动数字内容服 务，数字影像、数字动漫、数字文学、数字学习及数字版权保护关键技术及应用 服务体系；中华民族文化资源保护数字化技术服务，三维数字动漫/影视协同创 作与网络交易服务；人口、地理、社保、教育培训、医疗卫生及高技术产业相关

领域数字内容资源开发和多媒体互动应用服务。

133、 研发设计服务

面向科研开发的试验、测试、分析、评估等专业化服务，工程整体解决方案 和产品系统化集成高端研发设计服务，面向装备制造、消费产品等工业设计专业 服务，面向区域和专业领域的研发设计网络化协同公共服务平台开发与应用服 务，人机工程设计、仿真测试系统、设计软件平台等研发设计工具开发和应用服

务。

134、 生物技术服务

生物医药、生物农业、生物能源、生物制造、生物环保等领域的生物技术服 务。创新药物和以生物芯片为代表的生物医学工程产品上市前全过程的技术开发 与评价、产业工程技术、检测与标准技术服务等专业化服务；生物技术外包服务， 健康管理技术支撑服务。依托基因工程、细胞工程、分子育种等现代生物技术的

生物农业技术服务。生物能源、生物基新材料研发服务和生物环保技术服务。

135、 检验检测服务

支持分析、测试、计量、检疫、认证、溯源等技术服务。特种设备安全与节 能检测服务，质量安全风险监测预警与应急预警技术服务，生物安全检疫技术服 务，基于产品检测分析的综合解决方案服务，检测仪器设备、检测试剂盒、试剂 耗材的开发和研究。标准一致性（符合性）测试检验服务。标准信息分析及标准

中创新技术的分析、应用和保护等标准咨询服务。

136、 知识产权服务

知识产权信息检索、专利技术分析、知识产权布局研究、知识产权风险预警、 知识产权战略制定与管理咨询、法律事务等服务；知识产权数据加工、翻译、专 题数据库建设等信息加工服务；知识产权申请、注册、登记等代理服务；知识产

权的交易、推广、评估、投融资、证券化、托管、公证、培训等服务。

137、 科技成果转化服务

科技评估、科技招投标、科技情报咨询等科技信息服务；公共实验室、测试 中心、中试基地、研发环境等技术支持服务；创业辅导、孵化器、大学科技园等 科技成果转化平台服务；技术产权交易、技术经纪等中介服务，生产力促进中心

等科技中介服务。

国家发展改革委
　　　　　　　　　　　　　　　　　　　　　　　　　　科　　技　　部
　　　　　　　　　　　　　　　　　　　　　　　　　　工业和信息化部
　　　　　　　　　　　　　　　　　　　　　　　　　　商　　务　　部
　　　　　　　　　　　　　　　　　　　　　　　　　　知 识 产 权 局
　　　　　　　　　　　　　　　　　　　　　　　二〇一一年六月二十三日

**P3**

**Outline for the Development of Information Technology in the Construction Industry (2011-2015)**

[**2011-2015年建筑业信息化发展纲要**](http://www.mohurd.gov.cn/zcfg/jswj/gczl/201105/P020110517580718435647.doc)

建质[2011]67号

一、指导思想
　　深入贯彻落实科学发展观，坚持自主创新、重点跨越、支撑发展、引领未来的方针，高度重视信息化对建筑业发展的推动作用，通过统筹规划、政策导向，进一步加强建筑企业信息化建设，不断提高信息技术应用水平，促进建筑业技术进步和管理水平提升。
　　二、发展目标
　　（一）总体目标
　　“十二五”期间，基本实现建筑企业信息系统的普及应用，加快建筑信息模型（BIM）、基于网络的协同工作等新技术在工程中的应用，推动信息化标准建设，促进具有自主知识产权软件的产业化，形成一批信息技术应用达到国际先进水平的建筑企业。
　　（二）具体目标
　　1.企业信息化建设
　　工程总承包类　进一步优化业务流程，整合信息资源，完善提升设计集成、项目管理、企业运营管理等应用系统，构建基于网络的协同工作平台，提高集成化、智能化与自动化程度，推进设计施工一体化。
　　勘察设计类　完善提升企业管理系统，强化勘察设计信息资源整合，逐步建立信息资源的开发、管理及利用体系。推动基于BIM技术的协同设计系统建设与应用，提高工程勘察问题分析能力，提升检测监测分析水平，提高设计集成化与智能化程度。
　　施工类　优化企业和项目管理流程，提升企业和项目管理信息系统的集成应用水平，建设协同工作平台，研究实施企业资源计划（ERP）系统，支撑企业的集约化管理和持续发展。
　　以上各类企业应加强信息基础设施建设，提高企业信息系统安全水平，初步建立知识管理、决策支持等企业层面的信息系统，实现与企业和项目管理等信息系统的集成，提升企业决策水平和集中管控能力。
　　2.专项信息技术应用
　　加快推广BIM、协同设计、移动通讯、无线射频、虚拟现实、4D项目管理等技术在勘察设计、施工和工程项目管理中的应用，改进传统的生产与管理模式，提升企业的生产效率和管理水平。
　　3.信息化标准
　　完善建筑业行业与企业信息化标准体系和相关的信息化标准，推动信息资源整合，提高信息综合利用水平。
　　三、发展重点
　　（一）建筑企业信息系统
　　1.工程总承包类企业
　　围绕企业应用的两个层面，重点建设一个平台、八大应用系统。
　　两个层面指核心业务层和企业管理层；一个平台指信息基础设施平台；八大应用系统指核心业务层的设计集成、项目管理、项目文档管理、材料与采购管理、运营管理等系统，以及企业管理层的综合管理、辅助决策、知识管理与智能企业门户等系统。
　　（1）信息基础设施平台
　　加强信息基础设施和信息系统安全体系建设。重点强化数据中心和服务体系建设，打造安全可靠、资源共享的信息基础设施，支撑信息系统高效高质量运行。遵循国家信息安全等级保护要求，对重要应用系统实现分级保护，提升信息安全防护能力。
　　建立和完善信息标准体系，支撑信息系统开发和应用。重点建设信息基础设施、信息安全、信息编码、信息资源（如数据模型、模板等）以及信息系统应用等方面的标准。
　　（2）应用系统
　　①设计与施工集成系统
　　重点研究与应用智能化、可视化、模型设计、协同等技术，在提升各设计专业软件和普及应用新型智能二维和三维设计系统的基础上，逐步建立方案/工艺设计集成系统和专有技术与方案设计数据库，集成主要方案/工艺设计软件，创建方案/工艺设计协同工作平台；逐步建立工程设计集成系统和工程数据库，集成主要工程设计软件，创建工程设计协同工作平台；同时，逐步实现方案/工艺设计、工程设计、项目管理、施工管理、企业级管理等系统的集成。
　　②工程项目管理系统
　　以项目组合管理和项目群管理理论为基础，完善提升项目管理系统构架、管理工作流和信息流，整合项目资源，建立集成项目管理系统，提升项目管理整体执行力。规范与整合项目资源分解结构（WBS、CBS、OBS、RBS等）和编码体系；深化估算、投标报价和费用控制等系统，逐步建立适应国际工程估算、报价与费用控制的体系；完善商务与合同管理、风险管理及工程财务管理等系统，提升项目法律、融资、商务、资金、费用与成本管理水平和风险管控能力；深化应用计划进度控制系统，逐步建立施工管理和开车管理系统。同时，逐步实现与其他核心业务系统及企业级管理系统的集成。
　　③项目文档管理系统
　　整合与提升项目文档管理系统。优化文档管理流程，建立管理标准，完善文件编码体系；强化以工作流和状态为核心的过程管理和沟通管理，开发推广文档计划、跟踪、检测等控制功能，实现文档产生、批准、发布、升版、作废的生命周期管理，并逐步实现该系统与其他核心业务系统及企业级管理系统的集成。
　　④材料与采购管理系统
　　完善材料与采购管理系统。建立企业级材料标准库和编码库，实现材料表、请购、询价、评标、采购、催交、检验、运输、接运、仓库管理、材料预测、配料、材料发放及结算等全过程一体化的材料和采购管理；逐步建立以信誉认证、交易和电子支付等为核心的采购电子商务系统，优化材料供销过程；实现材料库与工厂安装模拟可视化系统的集成；逐步实现该系统与设计、项目管理、施工管理等系统的集成。
　　⑤企业运营管理系统
　　应用工作流、内容管理、电子印章、数字签名等技术，优化工作流程，有效组织和利用信息资源，增强运营管理的体系化和流程化，提高远程办公和协同工作能力；逐步实现与其他核心业务系统及企业级管理系统的集成。
　　⑥ 综合管理系统
　　以现代项目管理理论为基础，以经营管理、预算管理、成本管理、项目管理体系和核心业务系统为支撑，建立企业级综合管理系统，为决策层和职能管理层提供综合管理平台。整合企业项目与组织分解结构，建立项目核算和管控体系，加强经营、综合和执行计划的管理，实现预算、调度、成本核算和绩效考核的一体化，以及企业层面的统筹、协同、分级管控和资源优化配置。
　　⑦辅助决策系统
　　逐步建立企业数据仓库，并利用商业智能（BI）和数据挖掘等技术，依据决策理论，逐步建立辅助决策系统。
　　⑧知识管理系统与智能企业门户
　　收集、整理、组织和整合描述设计对象和专业技术的信息资源，研究知识管理机制与体系及知识管理系统建立的工具、方法、过程，建立知识管理的体系和系统。基于企业核心业务系统、综合管理系统、知识管理系统和企业数据仓库，整合企业内外网络信息资源，逐步建立智能企业门户，方便知识的利用，形成企业信息资源中心与个人信息资源中心。
　　2.勘察设计类企业
　　（1）信息基础设施平台
　　按需提升局域网、广域网和通信系统的性能。网络的主干带宽与客户端带宽能满足应用需求；条件具备时采用万兆网络平台，满足国际合作、异地协同工作及多媒体应用等需求。
　　加强网络新技术的应用，如虚拟专用网技术、3G无线通讯技术等，重视工程项目专网的建设。
　　适时更新和配备计算机设备，提高存储与备份系统的容量和性能，建立异地容灾备份系统，满足不断发展的企业应用需求。
　　配备有效的网络管理工具，实现对企业局域网与广域网、服务器、数据库系统及应用系统的有效监控和管理。
　　根据信息安全建设规划和应用需求，逐步建立较为完整的集防入侵、防病毒、传输加密、认证和访问控制于一体、具有较完备安全制度的信息安全体系。
　　（2）应用系统
　　推进BIM技术、基于网络的协同工作技术应用，提升和完善企业综合管理平台，实现企业信息管理与工程项目信息管理的集成，促进企业设计水平和管理水平的提高。
　　研究发展基于BIM技术的集成设计系统，逐步实现建筑、结构、水暖电等专业的信息共享及协同。
　　企业运营管理。完善财务管理、人力资源管理、办公自动化、档案管理等系统，并实现上述系统的集成；建设企业门户网站和客户关系管理系统；探索研究电子商务在工程建设过程中的应用。实现企业管理信息系统的提升。
　　生产经营管理。完善包含经营管理、合同管理、项目管理、技术管理、质量管理等功能的生产经营管理系统，与企业运营管理等系统有效集成，实现生产经营活动全过程的监控与管理。
　　（3）数据中心
　　逐步建立勘察设计信息资源的开发、管理及利用体系，探索发展信息资源产业机制，实现信息资源科学采集、广泛共享、快速流动、深度开发、有序配置、有效利用。
　　建立企业资源数据库，包括勘察设计标准、规范和标准图数据库，建筑材料、部品、工艺和设备数据库，岩土工程、区域水文地质、地下工程和相关检测监测数据库，建筑方案和典型设计数据库，以及工程项目信息与文档数据库等。
　　建设企业数字图书馆系统，实现设计图档、文档、图书、期刊、技术资料、有关政策法规和标准规范的数字化管理。
　　探索研究勘察设计知识的采集模式和表达方式，构建勘察设计知识库，积累并科学利用勘察设计知识资源，辅助设计创新能力的提升。
　　进一步研究制定企业资源数据库和知识库相关标准，重点研究制定资料信息数据、三维模型数据、电子工程图档信息等标准，为行业数据共享创造条件。
　　针对不同类型、不同规模勘察设计企业的特点，探索建立企业数据中心，并研究相应的管理模式和运行机制，为企业提供信息保障。
　　3.施工类企业
　　（1）特级资质施工总承包企业
　　研究实施企业资源计划系统（ERP），结合企业需求实现企业现有管理信息系统的集成，或者基于企业资源计划的理念建立新的管理信息系统，支撑企业向集约化管理和协同管理发展。
　　依据现代企业管理制度的需求，梳理、优化企业管理和主营业务流程，整合资源，适应信息化处理需求。
　　①信息基础设施平台
　　建设与软件应用需求相匹配、覆盖下属企业的专用网络，并实现项目现场与企业网络的连接。完善安全措施，保障应用系统的高效、安全、稳定运行。
　　参考国家及行业标准，借鉴其他企业标准，制定本企业的信息化标准，重点建设基础信息编码及施工项目信息化管理等标准。
　　②应用系统
　　项目综合管理系统。进一步推进项目综合管理系统的普及应用，全面提升施工项目管理水平。
　　企业管理信息系统。重点实现人力资源、财务资金、物资设备、工程项目等管理的集成，消除信息孤岛，在此基础上，逐步建立企业资源计划系统。
　　企业知识管理系统。研究相关知识的采集和管理方法，建立知识管理机制，实现知识管理系统化，为企业提供便利的知识资源再利用平台。
　　企业商业智能和决策支持系统。在完善企业管理信息系统的基础上，探索建立企业数据仓库，逐步发展企业商业智能和决策支持系统。
　　企业间的协同工作平台。围绕施工项目，建立企业间的协同工作平台，实现企业与项目其他参与方的有序信息沟通和数据共享。
　　（2）一级施工企业
　　①信息基础设施平台
　　建设与软件应用需求相匹配的企业网络系统，实现与下属企业及项目现场的网络连接。完善安全措施，保障应用系统的高效、安全、稳定运行。
　　②应用系统
　　企业办公自动化系统。普及应用企业办公自动化系统，提高企业办公效率。
　　项目综合管理系统。普及应用项目综合管理系统，提升施工项目管理水平。
　　企业管理信息系统。重点建设并集成人力资源、财务资金、物资材料等三大系统，实现企业管理与主营业务的信息化。
　　企业间的协同工作平台。围绕施工项目，逐步建立企业间的协同工作平台，实现企业与项目其他参与方的有序信息沟通和数据共享。
　　（3）二级及专业分包施工企业
　　①信息基础设施平台
　　建设与软件应用需求相匹配的企业网络系统，实现与项目现场的网络连接。完善安全措施，保障应用系统的高效、安全、稳定运行。
　　②应用系统
　　企业办公自动化系统。建设企业办公自动化系统，提高企业办公效率。
　　企业管理信息系统。重点建设并集成财务资金及物资材料等系统，逐步实现企业管理与主营业务的信息化。
　　（二）专项信息技术应用
　　1.设计阶段
　　（1）积极推进协同设计技术的普及应用，通过协同设计技术改变工程设计的沟通方式，减少“错、漏、碰、缺”等错误的发生，提高设计产品质量。
　　（2）探索研究基于BIM技术的三维设计技术，提高参数化、可视化和性能化设计能力，并为设计施工一体化提供技术支撑。
　　（3）积极探索项目全生命期管理（PLM）技术的研究和应用，实现工程全生命期信息的有效管理和共享。
　　（4）研究高性能计算技术在各类超高、超长、大跨等复杂工程设计中的应用，解决大型复杂结构高精度分析、优化和控制等问题，促进工程结构设计水平和设计质量的提高。
　　（5）推进仿真模拟和虚拟现实技术的应用，方便客户参与设计过程，提高设计质量。
　　（6）探索研究勘察设计成果电子交付与存档技术，逐步实现从传统文档管理到电子文档管理的转变。
　　2.施工阶段
　　（1）在施工阶段开展BIM技术的研究与应用，推进BIM技术从设计阶段向施工阶段的应用延伸，降低信息传递过程中的衰减。
　　（2）继续推广应用工程施工组织设计、施工过程变形监测、施工深化设计、大体积混凝土计算机测温等计算机应用系统。
　　（3）推广应用虚拟现实和仿真模拟技术，辅助大型复杂工程施工过程管理和控制，实现事前控制和动态管理。
　　（4）在工程项目现场管理中应用移动通讯和射频技术，通过与工程项目管理信息系统结合，实现工程现场远程监控和管理。
　　（5）研究基于BIM技术的4D项目管理信息系统在大型复杂工程施工过程中的应用，实现对建筑工程有效的可视化管理。
　　（6）研究工程测量与定位信息技术在大型复杂超高建筑工程以及隧道、深基坑施工中的应用，实现对工程施工进度、质量、安全的有效控制。
　　（7）研究工程结构健康监测技术在建筑及构筑物建造和使用过程中的应用。
　　（三）信息化标准
　　进一步完善建筑业行业与企业信息化标准体系，重点完善建筑工程设计、施工、验收全过程的信息化标准体系，推动信息资源的整合，提高信息综合利用水平。
　　进一步完善相关的信息化标准，重点完善建筑行业信息编码标准、数据交换标准、电子工程图档标准、电子文档交付标准等。
　　建立覆盖信息化应用水平、技术水平、普及程度以及应用成效等方面的建筑企业信息化绩效评价标准。
　　四、保障措施
　　（一）加强各级住房和城乡建设主管部门的引导作用
　　1. 加强建筑业信息化软科学研究，为建筑业信息化发展提供理论支撑。
　　2. 组织制定建筑企业信息化水平评价标准，推动企业开展信息化水平评价，促进企业信息化水平的提高。
　　3. 鼓励企业进行信息化标准建设，支持企业信息化标准上升为行业标准。
　　4. 积极推动企业信息系统安全等级保护工作和信息化保障体系的建设，提高企业信息安全水平。
　　5. 组织开展建筑业信息化示范工程，发挥示范企业与工程的示范带动作用，引导并推动本地区以及建筑行业整体信息化水平的提升。
　　6. 培育产业化示范基地，扶持自主产权软件企业，带动建筑业应用软件的产业化发展。
　　（二）发挥行业协会的服务作用
　　1. 组织编制行业信息化标准，规范信息资源，促进信息共享与集成。
　　2. 组织行业信息化经验和技术交流，开展企业信息化水平评价活动，促进企业信息化建设。
　　3. 开展行业信息化培训，推动信息技术的普及应用。
　　4. 开展行业应用软件的评价和推荐活动，保障企业信息化的投资效益。
　　（三）加强企业信息化保障体系建设
　　1. 加强企业信息化管理组织建设，设立专职的信息化管理部门，推进企业信息化主管（CIO）制度。
　　2. 加强企业信息化人才建设，建立和完善多渠道、多层次的信息化人才培养和考核制度，制定吸引与稳定信息化人才的措施。
　　3. 加大企业信息化资金投入，每年应编制独立的信息化预算，保障信息化建设资金需要。
　　4. 重视企业信息化标准建设工作，重点进行业务流程与信息的标准化。
　　5. 建立企业信息安全保障体系，确保企业信息安全。

中华人民共和国住房和城乡建设部
二〇一一年五月十日

**P4**

**National New Urbanization Plan (2014-2020)**

**国家新型城镇化规划（2014－2020年）**

[国务院公报](https://www.gov.cn/gongbao/currentissue.htm) > [2014年第9号](https://www.gov.cn/gongbao/content/)

目　　录

第一篇　规划背景
　第一章　重大意义
　第二章　发展现状
　第三章　发展态势
第二篇　指导思想和发展目标
　第四章　指导思想
　第五章　发展目标
第三篇　有序推进农业转移人口市民化
　第六章　推进符合条件农业转移人口落户城镇
　　第一节　健全农业转移人口落户制度
　　第二节　实施差别化落户政策
　第七章　推进农业转移人口享有城镇基本公共服务
　　第一节　保障随迁子女平等享有受教育权利
　　第二节　完善公共就业创业服务体系
　　第三节　扩大社会保障覆盖面
　　第四节　改善基本医疗卫生条件
　　第五节　拓宽住房保障渠道
　第八章　建立健全农业转移人口市民化推进机制
　　第一节　建立成本分担机制
　　第二节　合理确定各级政府职责
　　第三节　完善农业转移人口社会参与机制
第四篇　优化城镇化布局和形态
　第九章　优化提升东部地区城市群
　第十章　培育发展中西部地区城市群
　第十一章　建立城市群发展协调机制
　第十二章　促进各类城市协调发展
　　第一节　增强中心城市辐射带动功能
　　第二节　加快发展中小城市
　　第三节　有重点地发展小城镇
　第十三章　强化综合交通运输网络支撑
　　第一节　完善城市群之间综合交通运输网络
　　第二节　构建城市群内部综合交通运输网络
　　第三节　建设城市综合交通枢纽
　　第四节　改善中小城市和小城镇交通条件
第五篇　提高城市可持续发展能力
　第十四章　强化城市产业就业支撑
　　第一节　优化城市产业结构
　　第二节　增强城市创新能力
　　第三节　营造良好就业创业环境
　第十五章　优化城市空间结构和管理格局
　　第一节　改造提升中心城区功能
　　第二节　严格规范新城新区建设
　　第三节　改善城乡接合部环境
　第十六章　提升城市基本公共服务水平
　　第一节　优先发展城市公共交通
　　第二节　加强市政公用设施建设
　　第三节　完善基本公共服务体系
　第十七章　提高城市规划建设水平
　　第一节　创新规划理念
　　第二节　完善规划程序
　　第三节　强化规划管控
　　第四节　严格建筑质量管理
　第十八章　推动新型城市建设
　　第一节　加快绿色城市建设
　　第二节　推进智慧城市建设
　　第三节　注重人文城市建设
　第十九章　加强和创新城市社会治理
　　第一节　完善城市治理结构
　　第二节　强化社区自治和服务功能
　　第三节　创新社会治安综合治理
　　第四节　健全防灾减灾救灾体制
第六篇　推动城乡发展一体化
　第二十章　完善城乡发展一体化体制机制
　　第一节　推进城乡统一要素市场建设
　　第二节　推进城乡规划、基础设施和公共服务一体化
　第二十一章　加快农业现代化进程
　　第一节　保障国家粮食安全和重要农产品有效供给
　　第二节　提升现代农业发展水平
　　第三节　完善农产品流通体系
　第二十二章　建设社会主义新农村
　　第一节　提升乡镇村庄规划管理水平
　　第二节　加强农村基础设施和服务网络建设
　　第三节　加快农村社会事业发展
第七篇　改革完善城镇化发展体制机制
　第二十三章　推进人口管理制度改革
　第二十四章　深化土地管理制度改革
　第二十五章　创新城镇化资金保障机制
　第二十六章　健全城镇住房制度
　第二十七章　强化生态环境保护制度
第八篇　规划实施
　第二十八章　加强组织协调
　第二十九章　强化政策统筹
　第三十章　开展试点示范
　第三十一章　健全监测评估

　　国家新型城镇化规划（2014-2020年），根据中国共产党第十八次全国代表大会报告、《中共中央关于全面深化改革若干重大问题的决定》、中央城镇化工作会议精神、《中华人民共和国国民经济和社会发展第十二个五年规划纲要》和《全国主体功能区规划》编制，按照走中国特色新型城镇化道路、全面提高城镇化质量的新要求，明确未来城镇化的发展路径、主要目标和战略任务，统筹相关领域制度和政策创新，是指导全国城镇化健康发展的宏观性、战略性、基础性规划。

第一篇　规划背景

　　我国已进入全面建成小康社会的决定性阶段，正处于经济转型升级、加快推进社会主义现代化的重要时期，也处于城镇化深入发展的关键时期，必须深刻认识城镇化对经济社会发展的重大意义，牢牢把握城镇化蕴含的巨大机遇，准确研判城镇化发展的新趋势新特点，妥善应对城镇化面临的风险挑战。

第一章　重大意义

　　城镇化是伴随工业化发展，非农产业在城镇集聚、农村人口向城镇集中的自然历史过程，是人类社会发展的客观趋势，是国家现代化的重要标志。按照建设中国特色社会主义五位一体总体布局，顺应发展规律，因势利导，趋利避害，积极稳妥扎实有序推进城镇化，对全面建成小康社会、加快社会主义现代化建设进程、实现中华民族伟大复兴的中国梦，具有重大现实意义和深远历史意义。
　　——城镇化是现代化的必由之路。工业革命以来的经济社会发展史表明，一国要成功实现现代化，在工业化发展的同时，必须注重城镇化发展。当今中国，城镇化与工业化、信息化和农业现代化同步发展，是现代化建设的核心内容，彼此相辅相成。工业化处于主导地位，是发展的动力；农业现代化是重要基础，是发展的根基；信息化具有后发优势，为发展注入新的活力；城镇化是载体和平台，承载工业化和信息化发展空间，带动农业现代化加快发展，发挥着不可替代的融合作用。
　　——城镇化是保持经济持续健康发展的强大引擎。内需是我国经济发展的根本动力，扩大内需的最大潜力在于城镇化。目前我国常住人口城镇化率为53.7%，户籍人口城镇化率只有36%左右，不仅远低于发达国家80%的平均水平，也低于人均收入与我国相近的发展中国家60%的平均水平，还有较大的发展空间。城镇化水平持续提高，会使更多农民通过转移就业提高收入，通过转为市民享受更好的公共服务，从而使城镇消费群体不断扩大、消费结构不断升级、消费潜力不断释放，也会带来城市基础设施、公共服务设施和住宅建设等巨大投资需求，这将为经济发展提供持续的动力。
　　——城镇化是加快产业结构转型升级的重要抓手。产业结构转型升级是转变经济发展方式的战略任务，加快发展服务业是产业结构优化升级的主攻方向。目前我国服务业增加值占国内生产总值比重仅为46.1%，与发达国家74%的平均水平相距甚远，与中等收入国家53%的平均水平也有较大差距。城镇化与服务业发展密切相关，服务业是就业的最大容纳器。城镇化过程中的人口集聚、生活方式的变革、生活水平的提高，都会扩大生活性服务需求；生产要素的优化配置、三次产业的联动、社会分工的细化，也会扩大生产性服务需求。城镇化带来的创新要素集聚和知识传播扩散，有利于增强创新活力，驱动传统产业升级和新兴产业发展。
　　——城镇化是解决农业农村农民问题的重要途径。我国农村人口过多、农业水土资源紧缺，在城乡二元体制下，土地规模经营难以推行，传统生产方式难以改变，这是“三农”问题的根源。我国人均耕地仅0.1公顷，农户户均土地经营规模约0.6公顷，远远达不到农业规模化经营的门槛。城镇化总体上有利于集约节约利用土地，为发展现代农业腾出宝贵空间。随着农村人口逐步向城镇转移，农民人均资源占有量相应增加，可以促进农业生产规模化和机械化，提高农业现代化水平和农民生活水平。城镇经济实力提升，会进一步增强以工促农、以城带乡能力，加快农村经济社会发展。
　　——城镇化是推动区域协调发展的有力支撑。改革开放以来，我国东部沿海地区率先开放发展，形成了京津冀、长江三角洲、珠江三角洲等一批城市群，有力推动了东部地区快速发展，成为国民经济重要的增长极。但与此同时，中西部地区发展相对滞后，一个重要原因就是城镇化发展很不平衡，中西部城市发育明显不足。目前东部地区常住人口城镇化率达到62.2%，而中部、西部地区分别只有48.5%、44.8%。随着西部大开发和中部崛起战略的深入推进，东部沿海地区产业转移加快，在中西部资源环境承载能力较强地区，加快城镇化进程，培育形成新的增长极，有利于促进经济增长和市场空间由东向西、由南向北梯次拓展，推动人口经济布局更加合理、区域发展更加协调。
　　——城镇化是促进社会全面进步的必然要求。城镇化作为人类文明进步的产物，既能提高生产活动效率，又能富裕农民、造福人民，全面提升生活质量。随着城镇经济的繁荣，城镇功能的完善，公共服务水平和生态环境质量的提升，人们的物质生活会更加殷实充裕，精神生活会更加丰富多彩；随着城乡二元体制逐步破除，城市内部二元结构矛盾逐步化解，全体人民将共享现代文明成果。这既有利于维护社会公平正义、消除社会风险隐患，也有利于促进人的全面发展和社会和谐进步。

第二章　发展现状

　　改革开放以来，伴随着工业化进程加速，我国城镇化经历了一个起点低、速度快的发展过程。1978-2013年，城镇常住人口从1.7亿人增加到7.3亿人，城镇化率从17.9%提升到53.7%，年均提高1.02个百分点；城市数量从193个增加到658个，建制镇数量从2173个增加到20113个。京津冀、长江三角洲、珠江三角洲三大城市群，以2.8%的国土面积集聚了18%的人口，创造了36%的国内生产总值，成为带动我国经济快速增长和参与国际经济合作与竞争的主要平台。城市水、电、路、气、信息网络等基础设施显著改善，教育、医疗、文化体育、社会保障等公共服务水平明显提高，人均住宅、公园绿地面积大幅增加。城镇化的快速推进，吸纳了大量农村劳动力转移就业，提高了城乡生产要素配置效率，推动了国民经济持续快速发展，带来了社会结构深刻变革，促进了城乡居民生活水平全面提升，取得的成就举世瞩目。（见图1、表1、表2）
　　在城镇化快速发展过程中，也存在一些必须高度重视并着力解决的突出矛盾和问题。
　　——大量农业转移人口难以融入城市社会，市民化进程滞后。目前农民工已成为我国产业工人的主体，受城乡分割的户籍制度影响，被统计为城镇人口的2.34亿农民工及其随迁家属，未能在教育、就业、医疗、养老、保障性住房等方面享受城镇居民的基本公共服务，产城融合不紧密，产业集聚与人口集聚不同步，城镇化滞后于工业化。城镇内部出现新的二元矛盾，农村留守儿童、妇女和老人问题日益凸显，给经济社会发展带来诸多风险隐患。（见图2）
　　——“土地城镇化”快于人口城镇化，建设用地粗放低效。一些城市“摊大饼”式扩张，过分追求宽马路、大广场，新城新区、开发区和工业园区占地过大，建成区人口密度偏低。1996-2012年，全国建设用地年均增加724万亩，其中城镇建设用地年均增加357万亩；2010-2012年，全国建设用地年均增加953万亩，其中城镇建设用地年均增加515万亩。2000-2011年，城镇建成区面积增长76.4%，远高于城镇人口50.5%的增长速度；农村人口减少1.33亿人，农村居民点用地却增加了3045万亩。一些地方过度依赖土地出让收入和土地抵押融资推进城镇建设，加剧了土地粗放利用，浪费了大量耕地资源，威胁到国家粮食安全和生态安全，也加大了地方政府性债务等财政金融风险。
　　——城镇空间分布和规模结构不合理，与资源环境承载能力不匹配。东部一些城镇密集地区资源环境约束趋紧，中西部资源环境承载能力较强地区的城镇化潜力有待挖掘；城市群布局不尽合理，城市群内部分工协作不够、集群效率不高；部分特大城市主城区人口压力偏大，与综合承载能力之间的矛盾加剧；中小城市集聚产业和人口不足，潜力没有得到充分发挥；小城镇数量多、规模小、服务功能弱，这些都增加了经济社会和生态环境成本。
　　——城市管理服务水平不高，“城市病”问题日益突出。一些城市空间无序开发、人口过度集聚，重经济发展、轻环境保护，重城市建设、轻管理服务，交通拥堵问题严重，公共安全事件频发，城市污水和垃圾处理能力不足，大气、水、土壤等环境污染加剧，城市管理运行效率不高，公共服务供给能力不足，城中村和城乡接合部等外来人口集聚区人居环境较差。
　　——自然历史文化遗产保护不力，城乡建设缺乏特色。一些城市景观结构与所处区域的自然地理特征不协调，部分城市贪大求洋、照搬照抄，脱离实际建设国际大都市，“建设性”破坏不断蔓延，城市的自然和文化个性被破坏。一些农村地区大拆大建，照搬城市小区模式建设新农村，简单用城市元素与风格取代传统民居和田园风光，导致乡土特色和民俗文化流失。
　　——体制机制不健全，阻碍了城镇化健康发展。现行城乡分割的户籍管理、土地管理、社会保障制度，以及财税金融、行政管理等制度，固化着已经形成的城乡利益失衡格局，制约着农业转移人口市民化，阻碍着城乡发展一体化。

第三章　发展态势

　　根据世界城镇化发展普遍规律，我国仍处于城镇化率30%-70%的快速发展区间，但延续过去传统粗放的城镇化模式，会带来产业升级缓慢、资源环境恶化、社会矛盾增多等诸多风险，可能落入“中等收入陷阱”，进而影响现代化进程。随着内外部环境和条件的深刻变化，城镇化必须进入以提升质量为主的转型发展新阶段。
　　——城镇化发展面临的外部挑战日益严峻。在全球经济再平衡和产业格局再调整的背景下，全球供给结构和需求结构正在发生深刻变化，庞大生产能力与有限市场空间的矛盾更加突出，国际市场竞争更加激烈，我国面临产业转型升级和消化严重过剩产能的挑战巨大；发达国家能源资源消费总量居高不下，人口庞大的新兴市场国家和发展中国家对能源资源的需求迅速膨胀，全球资源供需矛盾和碳排放权争夺更加尖锐，我国能源资源和生态环境面临的国际压力前所未有，传统高投入、高消耗、高排放的工业化城镇化发展模式难以为继。
　　——城镇化转型发展的内在要求更加紧迫。随着我国农业富余劳动力减少和人口老龄化程度提高，主要依靠劳动力廉价供给推动城镇化快速发展的模式不可持续；随着资源环境瓶颈制约日益加剧，主要依靠土地等资源粗放消耗推动城镇化快速发展的模式不可持续；随着户籍人口与外来人口公共服务差距造成的城市内部二元结构矛盾日益凸显，主要依靠非均等化基本公共服务压低成本推动城镇化快速发展的模式不可持续。工业化、信息化、城镇化和农业现代化发展不同步，导致农业根基不稳、城乡区域差距过大、产业结构不合理等突出问题。我国城镇化发展由速度型向质量型转型势在必行。
　　——城镇化转型发展的基础条件日趋成熟。改革开放30多年来我国经济快速增长，为城镇化转型发展奠定了良好物质基础。国家着力推动基本公共服务均等化，为农业转移人口市民化创造了条件。交通运输网络的不断完善、节能环保等新技术的突破应用，以及信息化的快速推进，为优化城镇化空间布局和形态，推动城镇可持续发展提供了有力支撑。各地在城镇化方面的改革探索，为创新体制机制积累了经验。

第二篇　指导思想和发展目标

　　我国城镇化是在人口多、资源相对短缺、生态环境比较脆弱、城乡区域发展不平衡的背景下推进的，这决定了我国必须从社会主义初级阶段这个最大实际出发，遵循城镇化发展规律，走中国特色新型城镇化道路。

第四章　指导思想

　　高举中国特色社会主义伟大旗帜，以邓小平理论、“三个代表”重要思想、科学发展观为指导，紧紧围绕全面提高城镇化质量，加快转变城镇化发展方式，以人的城镇化为核心，有序推进农业转移人口市民化；以城市群为主体形态，推动大中小城市和小城镇协调发展；以综合承载能力为支撑，提升城市可持续发展水平；以体制机制创新为保障，通过改革释放城镇化发展潜力，走以人为本、四化同步、优化布局、生态文明、文化传承的中国特色新型城镇化道路，促进经济转型升级和社会和谐进步，为全面建成小康社会、加快推进社会主义现代化、实现中华民族伟大复兴的中国梦奠定坚实基础。
　　要坚持以下基本原则：
　　——以人为本，公平共享。以人的城镇化为核心，合理引导人口流动，有序推进农业转移人口市民化，稳步推进城镇基本公共服务常住人口全覆盖，不断提高人口素质，促进人的全面发展和社会公平正义，使全体居民共享现代化建设成果。
　　——四化同步，统筹城乡。推动信息化和工业化深度融合、工业化和城镇化良性互动、城镇化和农业现代化相互协调，促进城镇发展与产业支撑、就业转移和人口集聚相统一，促进城乡要素平等交换和公共资源均衡配置，形成以工促农、以城带乡、工农互惠、城乡一体的新型工农、城乡关系。
　　——优化布局，集约高效。根据资源环境承载能力构建科学合理的城镇化宏观布局，以综合交通网络和信息网络为依托，科学规划建设城市群，严格控制城镇建设用地规模，严格划定永久基本农田，合理控制城镇开发边界，优化城市内部空间结构，促进城市紧凑发展，提高国土空间利用效率。
　　——生态文明，绿色低碳。把生态文明理念全面融入城镇化进程，着力推进绿色发展、循环发展、低碳发展，节约集约利用土地、水、能源等资源，强化环境保护和生态修复，减少对自然的干扰和损害，推动形成绿色低碳的生产生活方式和城市建设运营模式。
　　——文化传承，彰显特色。根据不同地区的自然历史文化禀赋，体现区域差异性，提倡形态多样性，防止千城一面，发展有历史记忆、文化脉络、地域风貌、民族特点的美丽城镇，形成符合实际、各具特色的城镇化发展模式。
　　——市场主导，政府引导。正确处理政府和市场关系，更加尊重市场规律，坚持使市场在资源配置中起决定性作用，更好发挥政府作用，切实履行政府制定规划政策、提供公共服务和营造制度环境的重要职责，使城镇化成为市场主导、自然发展的过程，成为政府引导、科学发展的过程。
　　——统筹规划，分类指导。中央政府统筹总体规划、战略布局和制度安排，加强分类指导；地方政府因地制宜、循序渐进抓好贯彻落实；尊重基层首创精神，鼓励探索创新和试点先行，凝聚各方共识，实现重点突破，总结推广经验，积极稳妥扎实有序推进新型城镇化。

第五章　发展目标

　　——城镇化水平和质量稳步提升。城镇化健康有序发展，常住人口城镇化率达到60%左右，户籍人口城镇化率达到45%左右，户籍人口城镇化率与常住人口城镇化率差距缩小2个百分点左右，努力实现1亿左右农业转移人口和其他常住人口在城镇落户。
　　——城镇化格局更加优化。“两横三纵”为主体的城镇化战略格局基本形成，城市群集聚经济、人口能力明显增强，东部地区城市群一体化水平和国际竞争力明显提高，中西部地区城市群成为推动区域协调发展的新的重要增长极。城市规模结构更加完善，中心城市辐射带动作用更加突出，中小城市数量增加，小城镇服务功能增强。
　　——城市发展模式科学合理。密度较高、功能混用和公交导向的集约紧凑型开发模式成为主导，人均城市建设用地严格控制在100平方米以内，建成区人口密度逐步提高。绿色生产、绿色消费成为城市经济生活的主流，节能节水产品、再生利用产品和绿色建筑比例大幅提高。城市地下管网覆盖率明显提高。
　　——城市生活和谐宜人。稳步推进义务教育、就业服务、基本养老、基本医疗卫生、保障性住房等城镇基本公共服务覆盖全部常住人口，基础设施和公共服务设施更加完善，消费环境更加便利，生态环境明显改善，空气质量逐步好转，饮用水安全得到保障。自然景观和文化特色得到有效保护，城市发展个性化，城市管理人性化、智能化。
　　——城镇化体制机制不断完善。户籍管理、土地管理、社会保障、财税金融、行政管理、生态环境等制度改革取得重大进展，阻碍城镇化健康发展的体制机制障碍基本消除。（见专栏1）

第三篇　有序推进农业转移人口市民化

　　按照尊重意愿、自主选择，因地制宜、分步推进，存量优先、带动增量的原则，以农业转移人口为重点，兼顾高校和职业技术院校毕业生、城镇间异地就业人员和城区城郊农业人口，统筹推进户籍制度改革和基本公共服务均等化。

第六章　推进符合条件农业转移人口落户城镇

　　逐步使符合条件的农业转移人口落户城镇，不仅要放开小城镇落户限制，也要放宽大中城市落户条件。

第一节　健全农业转移人口落户制度

　　各类城镇要健全农业转移人口落户制度，根据综合承载能力和发展潜力，以就业年限、居住年限、城镇社会保险参保年限等为基准条件，因地制宜制定具体的农业转移人口落户标准，并向全社会公布，引导农业转移人口在城镇落户的预期和选择。

第二节　实施差别化落户政策

　　以合法稳定就业和合法稳定住所（含租赁）等为前置条件，全面放开建制镇和小城市落户限制，有序放开城区人口50万-100万的城市落户限制，合理放开城区人口100万-300万的大城市落户限制，合理确定城区人口300万-500万的大城市落户条件，严格控制城区人口500万以上的特大城市人口规模。大中城市可设置参加城镇社会保险年限的要求，但最高年限不得超过5年。特大城市可采取积分制等方式设置阶梯式落户通道调控落户规模和节奏。

第七章　推进农业转移人口
享有城镇基本公共服务

　　农村劳动力在城乡间流动就业是长期现象，按照保障基本、循序渐进的原则，积极推进城镇基本公共服务由主要对本地户籍人口提供向对常住人口提供转变，逐步解决在城镇就业居住但未落户的农业转移人口享有城镇基本公共服务问题。

第一节　保障随迁子女平等享有受教育权利

　　建立健全全国中小学生学籍信息管理系统，为学生学籍转接提供便捷服务。将农民工随迁子女义务教育纳入各级政府教育发展规划和财政保障范畴，合理规划学校布局，科学核定教师编制，足额拨付教育经费，保障农民工随迁子女以公办学校为主接受义务教育。对未能在公办学校就学的，采取政府购买服务等方式，保障农民工随迁子女在普惠性民办学校接受义务教育的权利。逐步完善农民工随迁子女在流入地接受中等职业教育免学费和普惠性学前教育的政策，推动各地建立健全农民工随迁子女接受义务教育后在流入地参加升学考试的实施办法。

第二节　完善公共就业创业服务体系

　　加强农民工职业技能培训，提高就业创业能力和职业素质。整合职业教育和培训资源，全面提供政府补贴职业技能培训服务。强化企业开展农民工岗位技能培训责任，足额提取并合理使用职工教育培训经费。鼓励高等学校、各类职业院校和培训机构积极开展职业教育和技能培训，推进职业技能实训基地建设。鼓励农民工取得职业资格证书和专项职业能力证书，并按规定给予职业技能鉴定补贴。加大农民工创业政策扶持力度，健全农民工劳动权益保护机制。实现就业信息全国联网，为农民工提供免费的就业信息和政策咨询。（见专栏2）

第三节　扩大社会保障覆盖面

　　扩大参保缴费覆盖面，适时适当降低社会保险费率。完善职工基本养老保险制度，实现基础养老金全国统筹，鼓励农民工积极参保、连续参保。依法将农民工纳入城镇职工基本医疗保险，允许灵活就业农民工参加当地城镇居民基本医疗保险。完善社会保险关系转移接续政策，在农村参加的养老保险和医疗保险规范接入城镇社保体系，建立全国统一的城乡居民基本养老保险制度，整合城乡居民基本医疗保险制度。强化企业缴费责任，扩大农民工参加城镇职工工伤保险、失业保险、生育保险比例。推进商业保险与社会保险衔接合作，开办各类补充性养老、医疗、健康保险。

第四节　改善基本医疗卫生条件

　　根据常住人口配置城镇基本医疗卫生服务资源，将农民工及其随迁家属纳入社区卫生服务体系，免费提供健康教育、妇幼保健、预防接种、传染病防控、计划生育等公共卫生服务。加强农民工聚居地疾病监测、疫情处理和突发公共卫生事件应对。鼓励有条件的地方将符合条件的农民工及其随迁家属纳入当地医疗救助范围。

第五节　拓宽住房保障渠道

　　采取廉租住房、公共租赁住房、租赁补贴等多种方式改善农民工居住条件。完善商品房配建保障性住房政策，鼓励社会资本参与建设。农民工集中的开发区和产业园区可以建设单元型或宿舍型公共租赁住房，农民工数量较多的企业可以在符合规定标准的用地范围内建设农民工集体宿舍。审慎探索由集体经济组织利用农村集体建设用地建设公共租赁住房。把进城落户农民完全纳入城镇住房保障体系。

第八章　建立健全农业转移人口市民化推进机制

　　强化各级政府责任，合理分担公共成本，充分调动社会力量，构建政府主导、多方参与、成本共担、协同推进的农业转移人口市民化机制。

第一节　建立成本分担机制

　　建立健全由政府、企业、个人共同参与的农业转移人口市民化成本分担机制，根据农业转移人口市民化成本分类，明确成本承担主体和支出责任。
　　政府要承担农业转移人口市民化在义务教育、劳动就业、基本养老、基本医疗卫生、保障性住房以及市政设施等方面的公共成本。企业要落实农民工与城镇职工同工同酬制度，加大职工技能培训投入，依法为农民工缴纳职工养老、医疗、工伤、失业、生育等社会保险费用。农民工要积极参加城镇社会保险、职业教育和技能培训等，并按照规定承担相关费用，提升融入城市社会的能力。

第二节　合理确定各级政府职责

　　中央政府负责统筹推进农业转移人口市民化的制度安排和政策制定，省级政府负责制定本行政区农业转移人口市民化总体安排和配套政策，市县政府负责制定本行政区城市和建制镇农业转移人口市民化的具体方案和实施细则。各级政府根据基本公共服务的事权划分，承担相应的财政支出责任，增强农业转移人口落户较多地区政府的公共服务保障能力。

第三节　完善农业转移人口社会参与机制

　　推进农民工融入企业、子女融入学校、家庭融入社区、群体融入社会，建设包容性城市。提高各级党代会代表、人大代表、政协委员中农民工的比例，积极引导农民工参加党组织、工会和社团组织，引导农业转移人口有序参政议政和参加社会管理。加强科普宣传教育，提高农民工科学文化和文明素质，营造农业转移人口参与社区公共活动、建设和管理的氛围。城市政府和用工企业要加强对农业转移人口的人文关怀，丰富其精神文化生活。

第四篇　优化城镇化布局和形态

　　根据土地、水资源、大气环流特征和生态环境承载能力，优化城镇化空间布局和城镇规模结构，在《全国主体功能区规划》确定的城镇化地区，按照统筹规划、合理布局、分工协作、以大带小的原则，发展集聚效率高、辐射作用大、城镇体系优、功能互补强的城市群，使之成为支撑全国经济增长、促进区域协调发展、参与国际竞争合作的重要平台。构建以陆桥通道、沿长江通道为两条横轴，以沿海、京哈京广、包昆通道为三条纵轴，以轴线上城市群和节点城市为依托、其他城镇化地区为重要组成部分，大中小城市和小城镇协调发展的“两横三纵”城镇化战略格局。（见图3）

第九章　优化提升东部地区城市群

　　东部地区城市群主要分布在优化开发区域，面临水土资源和生态环境压力加大、要素成本快速上升、国际市场竞争加剧等制约，必须加快经济转型升级、空间结构优化、资源永续利用和环境质量提升。
　　京津冀、长江三角洲和珠江三角洲城市群，是我国经济最具活力、开放程度最高、创新能力最强、吸纳外来人口最多的地区，要以建设世界级城市群为目标，继续在制度创新、科技进步、产业升级、绿色发展等方面走在全国前列，加快形成国际竞争新优势，在更高层次参与国际合作和竞争，发挥其对全国经济社会发展的重要支撑和引领作用。科学定位各城市功能，增强城市群内中小城市和小城镇的人口经济集聚能力，引导人口和产业由特大城市主城区向周边和其他城镇疏散转移。依托河流、湖泊、山峦等自然地理格局建设区域生态网络。
　　东部地区其他城市群，要根据区域主体功能定位，在优化结构、提高效益、降低消耗、保护环境的基础上，壮大先进装备制造业、战略性新兴产业和现代服务业，推进海洋经济发展。充分发挥区位优势，全面提高开放水平，集聚创新要素，增强创新能力，提升国际竞争力。统筹区域、城乡基础设施网络和信息网络建设，深化城市间分工协作和功能互补，加快一体化发展。

第十章　培育发展中西部地区城市群

　　中西部城镇体系比较健全、城镇经济比较发达、中心城市辐射带动作用明显的重点开发区域，要在严格保护生态环境的基础上，引导有市场、有效益的劳动密集型产业优先向中西部转移，吸纳东部返乡和就近转移的农民工，加快产业集群发展和人口集聚，培育发展若干新的城市群，在优化全国城镇化战略格局中发挥更加重要作用。
　　加快培育成渝、中原、长江中游、哈长等城市群，使之成为推动国土空间均衡开发、引领区域经济发展的重要增长极。加大对内对外开放力度，有序承接国际及沿海地区产业转移，依托优势资源发展特色产业，加快新型工业化进程，壮大现代产业体系，完善基础设施网络，健全功能完备、布局合理的城镇体系，强化城市分工合作，提升中心城市辐射带动能力，形成经济充满活力、生活品质优良、生态环境优美的新型城市群。依托陆桥通道上的城市群和节点城市，构建丝绸之路经济带，推动形成与中亚乃至整个欧亚大陆的区域大合作。
　　中部地区是我国重要粮食主产区，西部地区是我国水源保护区和生态涵养区。培育发展中西部地区城市群，必须严格保护耕地特别是基本农田，严格保护水资源，严格控制城市边界无序扩张，严格控制污染物排放，切实加强生态保护和环境治理，彻底改变粗放低效的发展模式，确保流域生态安全和粮食生产安全。

第十一章　建立城市群发展协调机制

　　统筹制定实施城市群规划，明确城市群发展目标、空间结构和开发方向，明确各城市的功能定位和分工，统筹交通基础设施和信息网络布局，加快推进城市群一体化进程。加强城市群规划与城镇体系规划、土地利用规划、生态环境规划等的衔接，依法开展规划环境影响评价。中央政府负责跨省级行政区的城市群规划编制和组织实施，省级政府负责本行政区内的城市群规划编制和组织实施。
　　建立完善跨区域城市发展协调机制。以城市群为主要平台，推动跨区域城市间产业分工、基础设施、环境治理等协调联动。重点探索建立城市群管理协调模式，创新城市群要素市场管理机制，破除行政壁垒和垄断，促进生产要素自由流动和优化配置。建立城市群成本共担和利益共享机制，加快城市公共交通“一卡通”服务平台建设，推进跨区域互联互通，促进基础设施和公共服务设施共建共享，促进创新资源高效配置和开放共享，推动区域环境联防联控联治，实现城市群一体化发展。

第十二章　促进各类城市协调发展

　　优化城镇规模结构，增强中心城市辐射带动功能，加快发展中小城市，有重点地发展小城镇，促进大中小城市和小城镇协调发展。

第一节　增强中心城市辐射带动功能

　　直辖市、省会城市、计划单列市和重要节点城市等中心城市，是我国城镇化发展的重要支撑。沿海中心城市要加快产业转型升级，提高参与全球产业分工的层次，延伸面向腹地的产业和服务链，加快提升国际化程度和国际竞争力。内陆中心城市要加大开发开放力度，健全以先进制造业、战略性新兴产业、现代服务业为主的产业体系，提升要素集聚、科技创新、高端服务能力，发挥规模效应和带动效应。区域重要节点城市要完善城市功能，壮大经济实力，加强协作对接，实现集约发展、联动发展、互补发展。特大城市要适当疏散经济功能和其他功能，推进劳动密集型加工业向外转移，加强与周边城镇基础设施连接和公共服务共享，推进中心城区功能向1小时交通圈地区扩散，培育形成通勤高效、一体发展的都市圈。

第二节　加快发展中小城市

　　把加快发展中小城市作为优化城镇规模结构的主攻方向，加强产业和公共服务资源布局引导，提升质量，增加数量。鼓励引导产业项目在资源环境承载力强、发展潜力大的中小城市和县城布局，依托优势资源发展特色产业，夯实产业基础。加强市政基础设施和公共服务设施建设，教育医疗等公共资源配置要向中小城市和县城倾斜，引导高等学校和职业院校在中小城市布局、优质教育和医疗机构在中小城市设立分支机构，增强集聚要素的吸引力。完善设市标准，严格审批程序，对具备行政区划调整条件的县可有序改市，把有条件的县城和重点镇发展成为中小城市。培育壮大陆路边境口岸城镇，完善边境贸易、金融服务、交通枢纽等功能，建设国际贸易物流节点和加工基地。（见专栏3）

第三节　有重点地发展小城镇

　　按照控制数量、提高质量，节约用地、体现特色的要求，推动小城镇发展与疏解大城市中心城区功能相结合、与特色产业发展相结合、与服务“三农”相结合。大城市周边的重点镇，要加强与城市发展的统筹规划与功能配套，逐步发展成为卫星城。具有特色资源、区位优势的小城镇，要通过规划引导、市场运作，培育成为文化旅游、商贸物流、资源加工、交通枢纽等专业特色镇。远离中心城市的小城镇和林场、农场等，要完善基础设施和公共服务，发展成为服务农村、带动周边的综合性小城镇。对吸纳人口多、经济实力强的镇，可赋予同人口和经济规模相适应的管理权。（见专栏4）

第十三章　强化综合交通运输网络支撑

　　完善综合运输通道和区际交通骨干网络，强化城市群之间交通联系，加快城市群交通一体化规划建设，改善中小城市和小城镇对外交通，发挥综合交通运输网络对城镇化格局的支撑和引导作用。到2020年，普通铁路网覆盖20万以上人口城市，快速铁路网基本覆盖50万以上人口城市；普通国道基本覆盖县城，国家高速公路基本覆盖20万以上人口城市；民用航空网络不断扩展，航空服务覆盖全国90%左右的人口。

第一节　完善城市群之间
综合交通运输网络

　　依托国家“五纵五横”综合运输大通道，加强东中部城市群对外交通骨干网络薄弱环节建设，加快西部城市群对外交通骨干网络建设，形成以铁路、高速公路为骨干，以普通国省道为基础，与民航、水路和管道共同组成的连接东西、纵贯南北的综合交通运输网络，支撑国家“两横三纵”城镇化战略格局。

第二节　构建城市群内部
综合交通运输网络

　　按照优化结构的要求，在城市群内部建设以轨道交通和高速公路为骨干，以普通公路为基础，有效衔接大中小城市和小城镇的多层次快速交通运输网络。提升东部地区城市群综合交通运输一体化水平，建成以城际铁路、高速公路为主体的快速客运和大能力货运网络。推进中西部地区城市群内主要城市之间的快速铁路、高速公路建设，逐步形成城市群内快速交通运输网络。

第三节　建设城市综合交通枢纽

　　建设以铁路、公路客运站和机场等为主的综合客运枢纽，以铁路和公路货运场站、港口和机场等为主的综合货运枢纽，优化布局，提升功能。依托综合交通枢纽，加强铁路、公路、民航、水运与城市轨道交通、地面公共交通等多种交通方式的衔接，完善集疏运系统与配送系统，实现客运“零距离”换乘和货运无缝衔接。

第四节　改善中小城市和小城镇交通条件

　　加强中小城市和小城镇与交通干线、交通枢纽城市的连接，加快国省干线公路升级改造，提高中小城市和小城镇公路技术等级、通行能力和铁路覆盖率，改善交通条件，提升服务水平。（见图4）

第五篇　提高城市可持续发展能力

　　加快转变城市发展方式，优化城市空间结构，增强城市经济、基础设施、公共服务和资源环境对人口的承载能力，有效预防和治理“城市病”，建设和谐宜居、富有特色、充满活力的现代城市。

第十四章　强化城市产业就业支撑

　　调整优化城市产业布局和结构，促进城市经济转型升级，改善营商环境，增强经济活力，扩大就业容量，把城市打造成为创业乐园和创新摇篮。

第一节　优化城市产业结构

　　根据城市资源环境承载能力、要素禀赋和比较优势，培育发展各具特色的城市产业体系。改造提升传统产业，淘汰落后产能，壮大先进制造业和节能环保、新一代信息技术、生物、新能源、新材料、新能源汽车等战略性新兴产业。适应制造业转型升级要求，推动生产性服务业专业化、市场化、社会化发展，引导生产性服务业在中心城市、制造业密集区域集聚；适应居民消费需求多样化，提升生活性服务业水平，扩大服务供给，提高服务质量，推动特大城市和大城市形成以服务经济为主的产业结构。强化城市间专业化分工协作，增强中小城市产业承接能力，构建大中小城市和小城镇特色鲜明、优势互补的产业发展格局。推进城市污染企业治理改造和环保搬迁。支持资源枯竭城市发展接续替代产业。

第二节　增强城市创新能力

　　顺应科技进步和产业变革新趋势，发挥城市创新载体作用，依托科技、教育和人才资源优势，推动城市走创新驱动发展道路。营造创新的制度环境、政策环境、金融环境和文化氛围，激发全社会创新活力，推动技术创新、商业模式创新和管理创新。建立产学研协同创新机制，强化企业在技术创新中的主体地位，发挥大型企业创新骨干作用，激发中小企业创新活力。建设创新基地，集聚创新人才，培育创新集群，完善创新服务体系，发展创新公共平台和风险投资机构，推进创新成果资本化、产业化。加强知识产权运用和保护，健全技术创新激励机制。推动高等学校提高创新人才培养能力，加快现代职业教育体系建设，系统构建从中职、高职、本科层次职业教育到专业学位研究生教育的技术技能人才培养通道，推进中高职衔接和职普沟通。引导部分地方本科高等学校转型发展为应用技术类型高校。试行普通高校、高职院校、成人高校之间的学分转换，为学生多样化成才提供选择。

第三节　营造良好就业创业环境

　　发挥城市创业平台作用，充分利用城市规模经济产生的专业化分工效应，放宽政府管制，降低交易成本，激发创业活力。完善扶持创业的优惠政策，形成政府激励创业、社会支持创业、劳动者勇于创业新机制。运用财政支持、税费减免、创业投资引导、政策性金融服务、小额贷款担保等手段，为中小企业特别是创业型企业发展提供良好的经营环境，促进以创业带动就业。促进以高校毕业生为重点的青年就业和农村转移劳动力、城镇困难人员、退役军人就业。结合产业升级开发更多适合高校毕业生的就业岗位，实行激励高校毕业生自主创业政策，实施离校未就业高校毕业生就业促进计划。合理引导高校毕业生就业流向，鼓励其到中小城市创业就业。

第十五章　优化城市空间结构和管理格局

　　按照统一规划、协调推进、集约紧凑、疏密有致、环境优先的原则，统筹中心城区改造和新城新区建设，提高城市空间利用效率，改善城市人居环境。

第一节　改造提升中心城区功能

　　推动特大城市中心城区部分功能向卫星城疏散，强化大中城市中心城区高端服务、现代商贸、信息中介、创意创新等功能。完善中心城区功能组合，统筹规划地上地下空间开发，推动商业、办公、居住、生态空间与交通站点的合理布局与综合利用开发。制定城市市辖区设置标准，优化市辖区规模和结构。按照改造更新与保护修复并重的要求，健全旧城改造机制，优化提升旧城功能。加快城区老工业区搬迁改造，大力推进棚户区改造，稳步实施城中村改造，有序推进旧住宅小区综合整治、危旧住房和非成套住房改造，全面改善人居环境。（见专栏5）

第二节　严格规范新城新区建设

　　严格新城新区设立条件，防止城市边界无序蔓延。因中心城区功能过度叠加、人口密度过高或规避自然灾害等原因，确需规划建设新城新区，必须以人口密度、产出强度和资源环境承载力为基准，与行政区划相协调，科学合理编制规划，严格控制建设用地规模，控制建设标准过度超前。统筹生产区、办公区、生活区、商业区等功能区规划建设，推进功能混合和产城融合，在集聚产业的同时集聚人口，防止新城新区空心化。加强现有开发区城市功能改造，推动单一生产功能向城市综合功能转型，为促进人口集聚、发展服务经济拓展空间。

第三节　改善城乡接合部环境

　　提升城乡接合部规划建设和管理服务水平，促进社区化发展，增强服务城市、带动农村、承接转移人口功能。加快城区基础设施和公共服务设施向城乡接合部地区延伸覆盖，规范建设行为，加强环境整治和社会综合治理，改善生活居住条件。保护生态用地和农用地，形成有利于改善城市生态环境质量的生态缓冲地带。

第十六章　提升城市基本公共服务水平

　　加强市政公用设施和公共服务设施建设，增加基本公共服务供给，增强对人口集聚和服务的支撑能力。

第一节　优先发展城市公共交通

　　将公共交通放在城市交通发展的首要位置，加快构建以公共交通为主体的城市机动化出行系统，积极发展快速公共汽车、现代有轨电车等大容量地面公共交通系统，科学有序推进城市轨道交通建设。优化公共交通站点和线路设置，推动形成公共交通优先通行网络，提高覆盖率、准点率和运行速度，基本实现100万人口以上城市中心城区公共交通站点500米全覆盖。强化交通综合管理，有效调控、合理引导个体机动化交通需求。推动各种交通方式、城市道路交通管理系统的信息共享和资源整合。

第二节　加强市政公用设施建设

　　建设安全高效便利的生活服务和市政公用设施网络体系。优化社区生活设施布局，健全社区养老服务体系，完善便民利民服务网络，打造包括物流配送、便民超市、平价菜店、家庭服务中心等在内的便捷生活服务圈。加强无障碍环境建设。合理布局建设公益性菜市场、农产品批发市场。统筹电力、通信、给排水、供热、燃气等地下管网建设，推行城市综合管廊，新建城市主干道路、城市新区、各类园区应实行城市地下管网综合管廊模式。加强城镇水源地保护与建设和供水设施改造与建设，确保城镇供水安全。加强防洪设施建设，完善城市排水与暴雨外洪内涝防治体系，提高应对极端天气能力。建设安全可靠、技术先进、管理规范的新型配电网络体系，加快推进城市清洁能源供应设施建设，完善燃气输配、储备和供应保障系统，大力发展热电联产，淘汰燃煤小锅炉。加强城镇污水处理及再生利用设施建设，推进雨污分流改造和污泥无害化处置。提高城镇生活垃圾无害化处理能力。合理布局建设城市停车场和立体车库，新建大中型商业设施要配建货物装卸作业区和停车场，新建办公区和住宅小区要配建地下停车场。

第三节　完善基本公共服务体系

　　根据城镇常住人口增长趋势和空间分布，统筹布局建设学校、医疗卫生机构、文化设施、体育场所等公共服务设施。优化学校布局和建设规模，合理配置中小学和幼儿园资源。加强社区卫生服务机构建设，健全与医院分工协作、双向转诊的城市医疗服务体系。完善重大疾病防控、妇幼保健等专业公共卫生和计划生育服务网络。加强公共文化、公共体育、就业服务、社保经办和便民利民服务设施建设。创新公共服务供给方式，引入市场机制，扩大政府购买服务规模，实现供给主体和方式多元化，根据经济社会发展状况和财力水平，逐步提高城镇居民基本公共服务水平，在学有所教、劳有所得、病有所医、老有所养、住有所居上持续取得新进展。

第十七章　提高城市规划建设水平

　　适应新型城镇化发展要求，提高城市规划科学性，加强空间开发管制，健全规划管理体制机制，严格建筑规范和质量管理，强化实施监督，提高城市规划管理水平和建筑质量。

第一节　创新规划理念

　　把以人为本、尊重自然、传承历史、绿色低碳理念融入城市规划全过程。城市规划要由扩张性规划逐步转向限定城市边界、优化空间结构的规划，科学确立城市功能定位和形态，加强城市空间开发利用管制，合理划定城市“三区四线”，合理确定城市规模、开发边界、开发强度和保护性空间，加强道路红线和建筑红线对建设项目的定位控制。统筹规划城市空间功能布局，促进城市用地功能适度混合。合理设定不同功能区土地开发利用的容积率、绿化率、地面渗透率等规范性要求。建立健全城市地下空间开发利用协调机制。统筹规划市区、城郊和周边乡村发展。（见专栏6）

第二节　完善规划程序

　　完善城市规划前期研究、规划编制、衔接协调、专家论证、公众参与、审查审批、实施管理、评估修编等工作程序，探索设立城市总规划师制度，提高规划编制科学化、民主化水平。推行城市规划政务公开，加大公开公示力度。加强城市规划与经济社会发展、主体功能区建设、国土资源利用、生态环境保护、基础设施建设等规划的相互衔接。推动有条件地区的经济社会发展总体规划、城市规划、土地利用规划等“多规合一”。

第三节　强化规划管控

　　保持城市规划权威性、严肃性和连续性，坚持一本规划一张蓝图持之以恒加以落实，防止换一届领导改一次规划。加强规划实施全过程监管，确保依规划进行开发建设。健全国家城乡规划督察员制度，以规划强制性内容为重点，加强规划实施督察，对违反规划行为进行事前事中监管。严格实行规划实施责任追究制度，加大对政府部门、开发主体、居民个人违法违规行为的责任追究和处罚力度。制定城市规划建设考核指标体系，加强地方人大对城市规划实施的监督检查，将城市规划实施情况纳入地方党政领导干部考核和离任审计。运用信息化等手段，强化对城市规划管控的技术支撑。

第四节　严格建筑质量管理

　　强化建筑设计、施工、监理和建筑材料、装修装饰等全流程质量管控。严格执行先勘察、后设计、再施工的基本建设程序，加强建筑市场各类主体的资质资格管理，推行质量体系认证制度，加大建筑工人职业技能培训力度。坚决打击建筑工程招投标、分包转包、材料采购、竣工验收等环节的违法违规行为，惩治擅自改变房屋建筑主体和承重结构等违规行为。健全建筑档案登记、查询和管理制度，强化建筑质量责任追究和处罚，实行建筑质量责任终身追究制度。

第十八章　推动新型城市建设

　　顺应现代城市发展新理念新趋势，推动城市绿色发展，提高智能化水平，增强历史文化魅力，全面提升城市内在品质。

第一节　加快绿色城市建设

　　将生态文明理念全面融入城市发展，构建绿色生产方式、生活方式和消费模式。严格控制高耗能、高排放行业发展。节约集约利用土地、水和能源等资源，促进资源循环利用，控制总量，提高效率。加快建设可再生能源体系，推动分布式太阳能、风能、生物质能、地热能多元化、规模化应用，提高新能源和可再生能源利用比例。实施绿色建筑行动计划，完善绿色建筑标准及认证体系、扩大强制执行范围，加快既有建筑节能改造，大力发展绿色建材，强力推进建筑工业化。合理控制机动车保有量，加快新能源汽车推广应用，改善步行、自行车出行条件，倡导绿色出行。实施大气污染防治行动计划，开展区域联防联控联治，改善城市空气质量。完善废旧商品回收体系和垃圾分类处理系统，加强城市固体废弃物循环利用和无害化处置。合理划定生态保护红线，扩大城市生态空间，增加森林、湖泊、湿地面积，将农村废弃地、其他污染土地、工矿用地转化为生态用地，在城镇化地区合理建设绿色生态廊道。（见专栏7）

第二节　推进智慧城市建设

　　统筹城市发展的物质资源、信息资源和智力资源利用，推动物联网、云计算、大数据等新一代信息技术创新应用，实现与城市经济社会发展深度融合。强化信息网络、数据中心等信息基础设施建设。促进跨部门、跨行业、跨地区的政务信息共享和业务协同，强化信息资源社会化开发利用，推广智慧化信息应用和新型信息服务，促进城市规划管理信息化、基础设施智能化、公共服务便捷化、产业发展现代化、社会治理精细化。增强城市要害信息系统和关键信息资源的安全保障能力。（见专栏8）

第三节　注重人文城市建设

　　发掘城市文化资源，强化文化传承创新，把城市建设成为历史底蕴厚重、时代特色鲜明的人文魅力空间。注重在旧城改造中保护历史文化遗产、民族文化风格和传统风貌，促进功能提升与文化文物保护相结合。注重在新城新区建设中融入传统文化元素，与原有城市自然人文特征相协调。加强历史文化名城名镇、历史文化街区、民族风情小镇文化资源挖掘和文化生态的整体保护，传承和弘扬优秀传统文化，推动地方特色文化发展，保存城市文化记忆。培育和践行社会主义核心价值观，加快完善文化管理体制和文化生产经营机制，建立健全现代公共文化服务体系、现代文化市场体系。鼓励城市文化多样化发展，促进传统文化与现代文化、本土文化与外来文化交融，形成多元开放的现代城市文化。（见专栏9）

第十九章　加强和创新城市社会治理

　　树立以人为本、服务为先理念，完善城市治理结构，创新城市治理方式，提升城市社会治理水平。

第一节　完善城市治理结构

　　顺应城市社会结构变化新趋势，创新社会治理体制，加强党委领导，发挥政府主导作用，鼓励和支持社会各方面参与，实现政府治理和社会自我调节、居民自治良性互动。坚持依法治理，加强法治保障，运用法治思维和法治方式化解社会矛盾。坚持综合治理，强化道德约束，规范社会行为，调节利益关系，协调社会关系，解决社会问题。坚持源头治理，标本兼治、重在治本，以网格化管理、社会化服务为方向，健全基层综合服务管理平台，及时反映和协调人民群众各方面各层次利益诉求。加强城市社会治理法律法规、体制机制、人才队伍和信息化建设。激发社会组织活力，加快实施政社分开，推进社会组织明确权责、依法自治、发挥作用。适合由社会组织提供的公共服务和解决的事项，交由社会组织承担。

第二节　强化社区自治和服务功能

　　健全社区党组织领导的基层群众自治制度，推进社区居民依法民主管理社区公共事务和公益事业。加快公共服务向社区延伸，整合人口、劳动就业、社保、民政、卫生计生、文化以及综治、维稳、信访等管理职能和服务资源，加快社区信息化建设，构建社区综合服务管理平台。发挥业主委员会、物业管理机构、驻区单位积极作用，引导各类社会组织、志愿者参与社区服务和管理。加强社区社会工作专业人才和志愿者队伍建设，推进社区工作人员专业化和职业化。加强流动人口服务管理。

第三节　创新社会治安综合治理

　　建立健全源头治理、动态协调、应急处置相互衔接、相互支撑的社会治安综合治理机制。创新立体化社会治安防控体系，改进治理方式，促进多部门城市管理职能整合，鼓励社会力量积极参与社会治安综合治理。及时解决影响人民群众安全的社会治安问题，加强对城市治安复杂部位的治安整治和管理。理顺城管执法体制，提高执法和服务水平。加大依法管理网络力度，加快完善互联网管理领导体制，确保国家网络和信息安全。

第四节　健全防灾减灾救灾体制

　　完善城市应急管理体系，加强防灾减灾能力建设，强化行政问责制和责任追究制。着眼抵御台风、洪涝、沙尘暴、冰雪、干旱、地震、山体滑坡等自然灾害，完善灾害监测和预警体系，加强城市消防、防洪、排水防涝、抗震等设施和救援救助能力建设，提高城市建筑灾害设防标准，合理规划布局和建设应急避难场所，强化公共建筑物和设施应急避难功能。完善突发公共事件应急预案和应急保障体系。加强灾害分析和信息公开，开展市民风险防范和自救互救教育，建立巨灾保险制度，发挥社会力量在应急管理中的作用。

第六篇　推动城乡发展一体化

　　坚持工业反哺农业、城市支持农村和多予少取放活方针，加大统筹城乡发展力度，增强农村发展活力，逐步缩小城乡差距，促进城镇化和新农村建设协调推进。

第二十章　完善城乡发展一体化体制机制

　　加快消除城乡二元结构的体制机制障碍，推进城乡要素平等交换和公共资源均衡配置，让广大农民平等参与现代化进程、共同分享现代化成果。

第一节　推进城乡统一要素市场建设

　　加快建立城乡统一的人力资源市场，落实城乡劳动者平等就业、同工同酬制度。建立城乡统一的建设用地市场，保障农民公平分享土地增值收益。建立健全有利于农业科技人员下乡、农业科技成果转化、先进农业技术推广的激励和利益分享机制。创新面向“三农”的金融服务，统筹发挥政策性金融、商业性金融和合作性金融的作用，支持具备条件的民间资本依法发起设立中小型银行等金融机构，保障金融机构农村存款主要用于农业农村。加快农业保险产品创新和经营组织形式创新，完善农业保险制度。鼓励社会资本投向农村建设，引导更多人才、技术、资金等要素投向农业农村。

第二节　推进城乡规划、
基础设施和公共服务一体化

　　统筹经济社会发展规划、土地利用规划和城乡规划，合理安排市县域城镇建设、农田保护、产业集聚、村落分布、生态涵养等空间布局。扩大公共财政覆盖农村范围，提高基础设施和公共服务保障水平。统筹城乡基础设施建设，加快基础设施向农村延伸，强化城乡基础设施连接，推动水电路气等基础设施城乡联网、共建共享。加快公共服务向农村覆盖，推进公共就业服务网络向县以下延伸，全面建成覆盖城乡居民的社会保障体系，推进城乡社会保障制度衔接，加快形成政府主导、覆盖城乡、可持续的基本公共服务体系，推进城乡基本公共服务均等化。率先在一些经济发达地区实现城乡一体化。

第二十一章　加快农业现代化进程

　　坚持走中国特色新型农业现代化道路，加快转变农业发展方式，提高农业综合生产能力、抗风险能力、市场竞争能力和可持续发展能力。

第一节　保障国家粮食安全和
重要农产品有效供给

　　确保国家粮食安全是推进城镇化的重要保障。严守耕地保护红线，稳定粮食播种面积。加强农田水利设施建设和土地整理复垦，加快中低产田改造和高标准农田建设。继续加大中央财政对粮食主产区投入，完善粮食主产区利益补偿机制，健全农产品价格保护制度，提高粮食主产区和种粮农民的积极性，将粮食生产核心区和非主产区产粮大县建设成为高产稳产商品粮生产基地。支持优势产区棉花、油料、糖料生产，推进畜禽水产品标准化规模养殖。坚持“米袋子”省长负责制和“菜篮子”市长负责制。完善主要农产品市场调控机制和价格形成机制。积极发展都市现代农业。

第二节　提升现代农业发展水平

　　加快完善现代农业产业体系，发展高产、优质、高效、生态、安全农业。提高农业科技创新能力，做大做强现代种业，健全农技综合服务体系，完善科技特派员制度，推广现代化农业技术。鼓励农业机械企业研发制造先进实用的农业技术装备，促进农机农艺融合，改善农业设施装备条件，耕种收综合机械化水平达到70%左右。创新农业经营方式，坚持家庭经营在农业中的基础性地位，推进家庭经营、集体经营、合作经营、企业经营等共同发展。鼓励承包经营权在公开市场上向专业大户、家庭农场、农民合作社、农业企业流转，发展多种形式规模经营。鼓励和引导工商资本到农村发展适合企业化经营的现代种养业，向农业输入现代生产要素和经营模式。加快构建公益性服务与经营性服务相结合、专项服务与综合服务相协调的新型农业社会化服务体系。

第三节　完善农产品流通体系

　　统筹规划农产品市场流通网络布局，重点支持重要农产品集散地、优势农产品产地批发市场建设，加强农产品期货市场建设。加快推进以城市便民菜市场（菜店）、生鲜超市、城乡集贸市场为主体的农产品零售市场建设。实施粮食收储供应安全保障工程，加强粮油仓储物流设施建设，发展农产品低温仓储、分级包装、电子结算。健全覆盖农产品收集、存储、加工、运输、销售各环节的冷链物流体系。加快培育现代流通方式和新型流通业态，大力发展快捷高效配送。积极推进“农批对接”、“农超对接”等多种形式的产销衔接，加快发展农产品电子商务，降低流通费用。强化农产品商标和地理标志保护。

第二十二章　建设社会主义新农村

　　坚持遵循自然规律和城乡空间差异化发展原则，科学规划县域村镇体系，统筹安排农村基础设施建设和社会事业发展，建设农民幸福生活的美好家园。

第一节　提升乡镇村庄规划管理水平

　　适应农村人口转移和村庄变化的新形势，科学编制县域村镇体系规划和镇、乡、村庄规划，建设各具特色的美丽乡村。按照发展中心村、保护特色村、整治空心村的要求，在尊重农民意愿的基础上，科学引导农村住宅和居民点建设，方便农民生产生活。在提升自然村落功能基础上，保持乡村风貌、民族文化和地域文化特色，保护有历史、艺术、科学价值的传统村落、少数民族特色村寨和民居。

第二节　加强农村基础设施和
服务网络建设

　　加快农村饮水安全建设，因地制宜采取集中供水、分散供水和城镇供水管网向农村延伸的方式解决农村人口饮用水安全问题。继续实施农村电网改造升级工程，提高农村供电能力和可靠性，实现城乡用电同网同价。加强以太阳能、生物沼气为重点的清洁能源建设及相关技术服务。基本完成农村危房改造。完善农村公路网络，实现行政村通班车。加强乡村旅游服务网络、农村邮政设施和宽带网络建设，改善农村消防安全条件。继续实施新农村现代流通网络工程，培育面向农村的大型流通企业，增加农村商品零售、餐饮及其他生活服务网点。深入开展农村环境综合整治，实施乡村清洁工程，开展村庄整治，推进农村垃圾、污水处理和土壤环境整治，加快农村河道、水环境整治，严禁城市和工业污染向农村扩散。

第三节　加快农村社会事业发展

　　合理配置教育资源，重点向农村地区倾斜。推进义务教育学校标准化建设，加强农村中小学寄宿制学校建设，提高农村义务教育质量和均衡发展水平。积极发展农村学前教育。加强农村教师队伍建设。建立健全新型职业化农民教育、培训体系。优先建设发展县级医院，完善以县级医院为龙头、乡镇卫生院和村卫生室为基础的农村三级医疗卫生服务网络，向农民提供安全价廉可及的基本医疗卫生服务。加强乡镇综合文化站等农村公共文化和体育设施建设，提高文化产品和服务的有效供给能力，丰富农民精神文化生活。完善农村最低生活保障制度。健全农村留守儿童、妇女、老人关爱服务体系。

第七篇　改革完善城镇化发展体制机制

　　加强制度顶层设计，尊重市场规律，统筹推进人口管理、土地管理、财税金融、城镇住房、行政管理、生态环境等重点领域和关键环节体制机制改革，形成有利于城镇化健康发展的制度环境。

第二十三章　推进人口管理制度改革

　　在加快改革户籍制度的同时，创新和完善人口服务和管理制度，逐步消除城乡区域间户籍壁垒，还原户籍的人口登记管理功能，促进人口有序流动、合理分布和社会融合。
　　——建立居住证制度。全面推行流动人口居住证制度，以居住证为载体，建立健全与居住年限等条件相挂钩的基本公共服务提供机制，并作为申请登记居住地常住户口的重要依据。城镇流动人口暂住证持有年限累计进居住证。
　　——健全人口信息管理制度。加强和完善人口统计调查制度，进一步改进人口普查方法，健全人口变动调查制度。加快推进人口基础信息库建设，分类完善劳动就业、教育、收入、社保、房产、信用、计生、税务等信息系统，逐步实现跨部门、跨地区信息整合和共享，在此基础上建设覆盖全国、安全可靠的国家人口综合信息库和信息交换平台，到2020年在全国实行以公民身份号码为唯一标识，依法记录、查询和评估人口相关信息制度，为人口服务和管理提供支撑。

第二十四章　深化土地管理制度改革

　　实行最严格的耕地保护制度和集约节约用地制度，按照管住总量、严控增量、盘活存量的原则，创新土地管理制度，优化土地利用结构，提高土地利用效率，合理满足城镇化用地需求。
　　——建立城镇用地规模结构调控机制。严格控制新增城镇建设用地规模，严格执行城市用地分类与规划建设用地标准，实行增量供给与存量挖潜相结合的供地、用地政策，提高城镇建设使用存量用地比例。探索实行城镇建设用地增加规模与吸纳农业转移人口落户数量挂钩政策。有效控制特大城市新增建设用地规模，适度增加集约用地程度高、发展潜力大、吸纳人口多的卫星城、中小城市和县城建设用地供给。适当控制工业用地，优先安排和增加住宅用地，合理安排生态用地，保护城郊菜地和水田，统筹安排基础设施和公共服务设施用地。建立有效调节工业用地和居住用地合理比价机制，提高工业用地价格。
　　——健全节约集约用地制度。完善各类建设用地标准体系，严格执行土地使用标准，适当提高工业项目容积率、土地产出率门槛，探索实行长期租赁、先租后让、租让结合的工业用地供应制度，加强工程建设项目用地标准控制。建立健全规划统筹、政府引导、市场运作、公众参与、利益共享的城镇低效用地再开发激励约束机制，盘活利用现有城镇存量建设用地，建立存量建设用地退出激励机制，推进老城区、旧厂房、城中村的改造和保护性开发，发挥政府土地储备对盘活城镇低效用地的作用。加强农村土地综合整治，健全运行机制，规范推进城乡建设用地增减挂钩，总结推广工矿废弃地复垦利用等做法。禁止未经评估和无害化治理的污染场地进行土地流转和开发利用。完善土地租赁、转让、抵押二级市场。
　　——深化国有建设用地有偿使用制度改革。扩大国有土地有偿使用范围，逐步对经营性基础设施和社会事业用地实行有偿使用。减少非公益性用地划拨，对以划拨方式取得用于经营性项目的土地，通过征收土地年租金等多种方式纳入有偿使用范围。
　　——推进农村土地管理制度改革。全面完成农村土地确权登记颁证工作，依法维护农民土地承包经营权。在坚持和完善最严格的耕地保护制度前提下，赋予农民对承包地占有、使用、收益、流转及承包经营权抵押、担保权能。保障农户宅基地用益物权，改革完善农村宅基地制度，在试点基础上慎重稳妥推进农民住房财产权抵押、担保、转让，严格执行宅基地使用标准，严格禁止一户多宅。在符合规划和用途管制前提下，允许农村集体经营性建设用地出让、租赁、入股，实行与国有土地同等入市、同权同价。建立农村产权流转交易市场，推动农村产权流转交易公开、公正、规范运行。
　　——深化征地制度改革。缩小征地范围，规范征地程序，完善对被征地农民合理、规范、多元保障机制。建立兼顾国家、集体、个人的土地增值收益分配机制，合理提高个人收益，保障被征地农民长远发展生计。健全争议协调裁决制度。
　　——强化耕地保护制度。严格土地用途管制，统筹耕地数量管控和质量、生态管护，完善耕地占补平衡制度，建立健全耕地保护激励约束机制。落实地方各级政府耕地保护责任目标考核制度，建立健全耕地保护共同责任机制；加强基本农田管理，完善基本农田永久保护长效机制，强化耕地占补平衡和土地整理复垦监管。

第二十五章　创新城镇化资金保障机制

　　加快财税体制和投融资机制改革，创新金融服务，放开市场准入，逐步建立多元化、可持续的城镇化资金保障机制。
　　——完善财政转移支付制度。按照事权与支出责任相适应的原则，合理确定各级政府在教育、基本医疗、社会保障等公共服务方面的事权，建立健全城镇基本公共服务支出分担机制。建立财政转移支付同农业转移人口市民化挂钩机制，中央和省级财政安排转移支付要考虑常住人口因素。依托信息化管理手段，逐步完善城镇基本公共服务补贴办法。
　　——完善地方税体系。培育地方主体税种，增强地方政府提供基本公共服务能力。加快房地产税立法并适时推进改革。加快资源税改革，逐步将资源税征收范围扩展到占用各种自然生态空间。推动环境保护费改税。
　　——建立规范透明的城市建设投融资机制。在完善法律法规和健全地方政府债务管理制度基础上，建立健全地方债券发行管理制度和评级制度，允许地方政府发行市政债券，拓宽城市建设融资渠道。创新金融服务和产品，多渠道推动股权融资，提高直接融资比重。发挥现有政策性金融机构的重要作用，研究制定政策性金融专项支持政策，研究建立城市基础设施、住宅政策性金融机构，为城市基础设施和保障性安居工程建设提供规范透明、成本合理、期限匹配的融资服务。理顺市政公用产品和服务价格形成机制，放宽准入，完善监管，制定非公有制企业进入特许经营领域的办法，鼓励社会资本参与城市公用设施投资运营。鼓励公共基金、保险资金等参与项目自身具有稳定收益的城市基础设施项目建设和运营。

第二十六章　健全城镇住房制度

　　建立市场配置和政府保障相结合的住房制度，推动形成总量基本平衡、结构基本合理、房价与消费能力基本适应的住房供需格局，有效保障城镇常住人口的合理住房需求。
　　——健全住房供应体系。加快构建以政府为主提供基本保障、以市场为主满足多层次需求的住房供应体系。对城镇低收入和中等偏下收入住房困难家庭，实行租售并举、以租为主，提供保障性安居工程住房，满足基本住房需求。稳定增加商品住房供应，大力发展二手房市场和住房租赁市场，推进住房供应主体多元化，满足市场多样化住房需求。
　　——健全保障性住房制度。建立各级财政保障性住房稳定投入机制，扩大保障性住房有效供给。完善租赁补贴制度，推进廉租住房、公共租赁住房并轨运行。制定公平合理、公开透明的保障性住房配租政策和监管程序，严格准入和退出制度，提高保障性住房物业管理、服务水平和运营效率。
　　——健全房地产市场调控长效机制。调整完善住房、土地、财税、金融等方面政策，共同构建房地产市场调控长效机制。各城市要编制城市住房发展规划，确定住房建设总量、结构和布局。确保住房用地稳定供应，完善住房用地供应机制，保障性住房用地应保尽保，优先安排政策性商品住房用地，合理增加普通商品住房用地，严格控制大户型高档商品住房用地。实行差别化的住房税收、信贷政策，支持合理自住需求，抑制投机投资需求。依法规范市场秩序，健全法律法规体系，加大市场监管力度。建立以土地为基础的不动产统一登记制度，实现全国住房信息联网，推进部门信息共享。

第二十七章　强化生态环境保护制度

　　完善推动城镇化绿色循环低碳发展的体制机制，实行最严格的生态环境保护制度，形成节约资源和保护环境的空间格局、产业结构、生产方式和生活方式。
　　——建立生态文明考核评价机制。把资源消耗、环境损害、生态效益纳入城镇化发展评价体系，完善体现生态文明要求的目标体系、考核办法、奖惩机制。对限制开发区域和生态脆弱的国家扶贫开发工作重点县取消地区生产总值考核。
　　——建立国土空间开发保护制度。建立空间规划体系，坚定不移实施主体功能区制度，划定生态保护红线，严格按照主体功能区定位推动发展，加快完善城镇化地区、农产品主产区、重点生态功能区空间开发管控制度，建立资源环境承载能力监测预警机制。强化水资源开发利用控制、用水效率控制、水功能区限制纳污管理。对不同主体功能区实行差别化财政、投资、产业、土地、人口、环境、考核等政策。
　　——实行资源有偿使用制度和生态补偿制度。加快自然资源及其产品价格改革，全面反映市场供求、资源稀缺程度、生态环境损害成本和修复效益。建立健全居民生活用电、用水、用气等阶梯价格制度。制定并完善生态补偿方面的政策法规，切实加大生态补偿投入力度，扩大生态补偿范围，提高生态补偿标准。
　　——建立资源环境产权交易机制。发展环保市场，推行节能量、碳排放权、排污权、水权交易制度，建立吸引社会资本投入生态环境保护的市场化机制，推行环境污染第三方治理。
　　——实行最严格的环境监管制度。建立和完善严格监管所有污染物排放的环境保护管理制度，独立进行环境监管和行政执法。完善污染物排放许可制，实行企事业单位污染物排放总量控制制度。加大环境执法力度，严格环境影响评价制度，加强突发环境事件应急能力建设，完善以预防为主的环境风险管理制度。对造成生态环境损害的责任者严格实行赔偿制度，依法追究刑事责任。建立陆海统筹的生态系统保护修复和污染防治区域联动机制。开展环境污染强制责任保险试点。

第八篇　规划实施

　　本规划由国务院有关部门和地方各级政府组织实施。各地区各部门要高度重视、求真务实、开拓创新、攻坚克难，确保规划目标和任务如期完成。

第二十八章　加强组织协调

　　合理确定中央与地方分工，建立健全城镇化工作协调机制。中央政府要强化制度顶层设计，统筹重大政策研究和制定，协调解决城镇化发展中的重大问题。国家发展改革委要牵头推进规划实施和相关政策落实，监督检查工作进展情况。各有关部门要切实履行职责，根据本规划提出的各项任务和政策措施，研究制定具体实施方案。地方各级政府要全面贯彻落实本规划，建立健全工作机制，因地制宜研究制定符合本地实际的城镇化规划和具体政策措施。加快培养一批专家型城市管理干部，提高城镇化管理水平。

第二十九章　强化政策统筹

　　根据本规划制定配套政策，建立健全相关法律法规、标准体系。加强部门间政策制定和实施的协调配合，推动人口、土地、投融资、住房、生态环境等方面政策和改革举措形成合力、落到实处。城乡规划、土地利用规划、交通规划等要落实本规划要求，其他相关专项规划要加强与本规划的衔接协调。

第三十章　开展试点示范

　　本规划实施涉及诸多领域的改革创新，对已经形成普遍共识的问题，如长期进城务工经商的农业转移人口落户、城市棚户区改造、农民工随迁子女义务教育、农民工职业技能培训和中西部地区中小城市发展等，要加大力度，抓紧解决。对需要深入研究解决的难点问题，如建立农业转移人口市民化成本分担机制，建立多元化、可持续的城镇化投融资机制，建立创新行政管理、降低行政成本的设市设区模式，改革完善农村宅基地制度等，要选择不同区域不同城市分类开展试点。继续推进创新城市、智慧城市、低碳城镇试点。深化中欧城镇化伙伴关系等现有合作平台，拓展与其他国家和国际组织的交流，开展多形式、多领域的务实合作。

第三十一章　健全监测评估

　　加强城镇化统计工作，顺应城镇化发展态势，建立健全统计监测指标体系和统计综合评价指标体系，规范统计口径、统计标准和统计制度方法。加快制定城镇化发展监测评估体系，实施动态监测与跟踪分析，开展规划中期评估和专项监测，推动本规划顺利实施。

| 表1　城市（镇）数量和规模变化情况（单位：个） | | |
| --- | --- | --- |
|  | 1978年 | 2010年 |
| 城市 | 193 | 658 |
| 1000万以上人口城市 | 0 | 6 |
| 500万—1000万人口城市 | 2 | 10 |
| 300万—500万人口城市 | 2 | 21 |
| 100万—300万人口城市 | 25 | 103 |
| 50万—100万人口城市 | 35 | 138 |
| 50万以下人口城市 | 129 | 380 |
| 建制镇 | 2173 | 19410 |
| 注：2010年数据根据第六次全国人口普査数据整理。 | | |

| 表2　城市基础设施和服务设施变化情况 | | |
| --- | --- | --- |
| 指　　标 | 2000年 | 2012年 |
| 用水普及率（%） | 63.9 | 97.2 |
| 燃气普及率（%） | 44.6 | 93.2 |
| 人均道路面积（平方米） | 6.1 | 14.4 |
| 人均住宅建筑面积（平方米） | 20.3 | 32.9 |
| 污水处理率（%） | 34.3 | 87.3 |
| 人均公园绿地面积（平方米） | 3.7 | 12.3 |
| 普通中学（所） | 14473 | 17333 |
| 病床数（万张） | 142.6 | 273.3 |

| 专栏1　新型城镇化主要指标 | | |
| --- | --- | --- |
| 指　标 | 2012年 | 2020年 |
| 城镇化水平 |  |  |
| 常住人口城镇化率（%） | 52.6 | 60左右 |
| 户籍人口城镇化率（%） | 35.3 | 45左右 |
| 基本公共服务 |  |  |
| 农民工随迁子女接受义务教育比例（%） |  | ≥99 |
| 城镇失业人员、农民工、新成长劳动力 　　免费接受基本职业技能培训覆盖率（%） |  | ≥95 |
| 城镇常住人口基本养老保险覆盖率（%） | 66.9 | ≥90 |
| 城镇常住人口基本医疗保险覆盖率（%） | 95 | 98 |
| 城镇常住人口保障性住房覆盖率（%） | 12.5 | ≥23 |
| 基础设施 |  |  |
| 百万以上人口城市公共交通占机动化出行比例（%） | 45* | 60 |
| 城镇公共供水普及率（%） | 81.7 | 90 |
| 城市污水处理率（%） | 87.3 | 95 |
| 城市生活垃圾无害化处理率（%） | 84.8 | 95 |
| 城市家庭宽带接入能力（Mbps） | 4 | ≥50 |
| 城市社区综合服务设施覆盖率（%） | 72.5 | 100 |
| 资源环境 |  |  |
| 人均城市建设用地（平方米） |  | ≤100 |
| 城镇可再生能源消费比重（%） | 8.7 | 13 |
| 城镇绿色建筑占新建建筑比重（%） | 2 | 50 |
| 城市建成区绿地率（%） | 35.7 | 38.9 |
| 地级以上城市空气质量达到国家标准的比例（%） | 40.9 | 60 |
| 注：①带*为2011年数据。 　　②城镇常住人口基本养老保险覆盖率指标中，常住人口不含16周岁以下人员和在校学生。 　　③城镇保障性住房：包括公租房（含廉租房）、政策性商品住房和棚户区改造安置住房等。 　　④人均城市建设用地：国家《城市用地分类与规划建设用地标准》规定，人均城市建设用地标准为 　　　65.0－115.0平方米， 新建城市为85.1－105.0平方米。 　　⑤城市空气质量国家标准：在1996年标准基础上，增设了PM2.5浓度限值和臭氧8小时平均浓度限值，调 　　　整了PM10、二氧化氮、铅等浓度限值。 | | |

| 专栏2　农民工职业技能提升计划 |
| --- |
| 01　就业技能培训 　　　对转移到非农产业务工经商的农村劳动者开展专项技能或初级技能培训。依托技工院校、中高等职业 　　　院校、职业技能实训基地等培训机构，加大各级政府投入，开展政府补贴农民工就业技能培训，毎年 　　　培训1000万人次，基本消除新成长劳动力无技能从业现象。对少数民族转移就业人员实行双语技能培 　　　训。 |
| 02　岗位技能提升培训 　　　对与企业签订一定期限劳动合同的在岗农民工进行提高技能水平培训。鼓励企业结合行业特点和岗位 　　　技能需求，开展农民工在岗技能提升培训，毎年培训农民工1000万人次。 |
| 03　高技能人才和创业培训 　　　对符合条件的具备中高级技能的农民工实施高技能人才培训计划，完善补贴政策，每年培养100万髙 　　　技能人才。对有创业意愿并具备创业条件的农民工开展提升创业能力培训。 |
| 04　劳动预备制培训 　　　对农村未能继续升学并准备进入非农产业就业或进城务工的应届初高中毕业生、农村籍退役士兵进行 　　　储备性专业技能培训。 |
| 05　社区公益性培训 　　　组织中高等职业院校、普通高校、技工院校开展面向农民工的公益性教育培训，与街道、社区合作， 　　　举办灵活多样的社区培训，提升农民工的职业技能和综合素质。 |
| 06　职业技能培训能力建设 　　　依托现有各类职业教育和培训机构，提升改造一批职业技能实训基地。鼓励大中型企业联合技工院 　　　校、职业院校，建设一批农民工实训基地。支持一批职业教育优质特色学校和示范性中高等职业院校 　　　建设。 |

| 专栏3　重点建设的陆路边境口岸城镇 |
| --- |
| 01　面向东北亚 　　　丹东、集安、临江、长白、和龙、图们、珲春、黑河、绥芬河、抚远、同江、东宁、满洲里、二连浩 　　　特、甘其毛都、策克 |
| 02　面向中亚西亚 　　　喀什、霍尔果斯、伊宁、博乐、阿拉山口、塔城 |
| 03　面向东南亚 　　　东兴、凭祥、宁明、龙州、大新、靖西、那坡、瑞丽、磨憨、畹町、河口 |
| 04　面向南亚 　　　樟木、吉隆、亚东、普兰、日屋 |

| 专栏4　县城和重点镇基础设施提升工程 |
| --- |
| 01　公共供水 　　　加强供水设施建设，实现县城和重点镇公共供水普及率85%以上。 |
| 02　污水处理 　　　因地制宜建设集中污水处理厂或分散型生态处理设施，使所有县城和重点镇具备污水处理能力，实现 　　　县城污水处理率达85%左右、重点镇达70%左右。 |
| 03　垃圾处理 　　　实现县城具备垃圾无害化处理能力，按照以城带乡模式推进重点镇垃圾无害化处理，重点建设垃圾收 　　　集、转运设施，实现重点镇垃圾收集、转运全覆盖。 |
| 04　道路交通 　　　统筹城乡交通一体化发展，县城基本实现高等级公路连通，重点镇积极发展公共交通。 |
| 05　燃气供热 　　　加快城镇天然气（含煤层气等）管网、液化天然气（压缩天然气）站、集中供热等设施建设，因地制 　　　宜发展大中型沼气、生物质燃气和地热能，县城逐步推进燃气替代生活燃煤，北方地区县城和重点镇 　　　集中供热水平明显提高。 |
| 06　分布式能源 　　　城镇建设和改造要优先采用分布式能源，资源丰富地区的城镇新能源和可再生能源消费比重显著提 　　　高。鼓励条件适宜地区大力促进可再生能源建筑应用。 |

| 专栏5　棚户区改造行动计划 |
| --- |
| 01　城市棚户区改造 　　　加快推进集中成片城市棚户区改造，逐步将其他棚户区、城中村改造统一纳入城市棚户区改造范围， 　　　到2020年基本完成城市棚户区改造任务。 |
| 02　国有工矿棚户区改造 　　　将位于城市规划区内的国有工矿棚户区统一纳入城市棚户区改造范围，按照属地原则将铁路、钢铁、 　　　有色、黄金等行业棚户区纳入各地棚户区改造规划组织实施。 |
| 03　国有林区棚户区改造 　　　加快改造国有林区棚户区和国有林场危旧房，将国有林区（场）外其他林业基层单位符合条件的住房 　　　困难人员纳入当地城镇住房保障体系。 |
| 04　国有垦区危房改造 　　　加快改造国有垦区危房，将华侨农场非归难侨危房改造统一纳入垦区危房改造中央补助支持范围。 |

| 专栏6　城市“三区四线”规划管理 |
| --- |
| 01　禁建区 　　　基本农田、行洪河道、水源地一级保护区、风景名胜区核心区、自然保护区核心区和缓冲区、森林湿 　　　地公园生态保育区和恢复重建区、地质公园核心区、道路红线、区域性市政走廊用地范围内、城市绿 　　　地、地质灾害易发区、矿产采空区、文物保护单位保护范围等，禁止城市建设开发活动。 |
| 02　限建区 　　　水源地二级保护区、地下水防护区、风景名胜区非核心区、自然保护区非核心区和缓冲区、森林公园 　　　非生态保育区、湿地公园非保育区和恢复重建区、地质公园非核心区、海陆交界生态敏感区和灾害易 　　　发区、文物保护单位建设控制地带、文物地下埋藏区、机场噪声控制区、市政走廊预留和道路红线外 　　　控制区、矿产采空区外围、地质灾害低易发区、蓄滞洪区、行洪河道外围一定范围等，限制城市建设 　　　开发活动。 |
| 03　适建区 　　　在已经划定为城市建设用地的区域，合理安排生产用地、生活用地和生态用地，合理确定开发时序、 　　　开发模式和开发强度。 |
| 04　绿线 　　　划定城市各类绿地范围的控制线，规定保护要求和控制指标。 |
| 05　蓝线 　　　划定在城市规划中确定的江、河、湖、库、渠和湿地等城市地表水体保护和控制的地域界线，规定保 　　　护要求和控制指标。 |
| 06　紫线 　　　划定国家历史文化名城内的历史文化街区和省、自治区、直辖市人民政府公布的历史文化街区的保护 　　　范围界线，以及城市历史文化街区外经县级以上人民政府公布保护的历史建筑的保护范围界线。 |
| 07　黄线 　　　划定对城市发展全局有影响、必须控制的城市基础设施用地的控制界线，规定保护要求和控制指标。 |

| 专栏7　绿色城市建设重点 |
| --- |
| 01　绿色能源 　　　推进新能源示范城市建设和智能微电网示范工程建设，依托新能源示范城市建设分布式光伏发电示范 　　　区。在北方地区城镇开展风电清洁供暖示范工程。选择部分县城开展可再生能源热利用示范工程，加 　　　强绿色能源县建设。 |
| 02　绿色建筑 　　　推进既有建筑供热计量和节能改造，基本完成北方采暖地区居住建筑供热计量和节能改造，积极推进 　　　夏热冬冷地区建筑节能改造和公共建筑节能改造。逐步提高新建建筑能效水平，严格执行节能标准。 　　　积极推进建筑工业化、标准化，提高住宅工业化比例。政府投资的公益性建筑、保障性住房和大型公 　　　共建筑全面执行绿色建筑标准和认证。 |
| 03　绿色交通 　　　加快发展新能源、小排量等环保型汽车，加快充电站、充电桩、加气站等配套设施建设，加强步行和 　　　自行车等慢行交通系统建设，积极推进混合动力、纯电动、天然气等新能源和清洁燃料车辆在公共交 　　　通行业的示范应用。推进机场、车站、码头节能节水改造，推广使用太阳能等可再生能源。继续严格 　　　实行运营车辆燃料消耗量准入制度，到2020年淘汰全部黄标车。 |
| 04　产业园区循环化改造 　　　以国家级和省级产业园区为重点，推进循环化改造，实现土地集约利用、废物交换利用、能量梯级利 　　　用，废水循环利用和污染物集中处理。 |
| 05　城市环境综合整治 　　　实施清洁空气工程，强化大气污染综合防治，明显改善城市空气质量；实施安全饮用水工程，治理地 　　　表水、地下水，实现水质、 水量双保障；开展存量生活垃圾治理工作；实施重金属污染防治工程，推 　　　进重点地区污染场地和土壤修复治理。实施森林、湿地保护与修复。 |
| 06　绿色新生活行动 　　　在衣食住行游等方面，加快向简约适度、绿色低碳、文明节约方式转变。培育生态文化，引导绿色消 　　　费，推广节能环保型汽车、节能省地型住宅。健全城市废旧商品回收体系和餐厨废弃物资源化利用体 　　　系，减少使用一次性产品，抑制商品过度包装。 |

| 专栏8　智慧城市建设方向 |
| --- |
| 01　信息网络宽带化 　　　推进光纤到户和“光进铜退”，实现光纤网络基本覆盖城市家庭，城市宽带接入能力达到50Mbps， 　　　50%家庭达到100Mbps，发达城市部分家庭达到1Gbps。推动4G网络建设，加快城市公共热点区域无线 　　　局域网覆盖。 |
| 02　规划管理信息化 　　　发展数字化城市管理，推动平台建设和功能拓展，建立城市统一的地理空间信息平台及建（构）筑物 　　　数据库，构建智慧城市公共信息平台，统筹推进城市规划、国土利用、城市管网、园林绿化、环境保 　　　护等市政基础设施管理的数字化和精准化。 |
| 03　基础设施智能化 　　　发展智能交通，实现交通诱导、指挥控制、调度管理和应急处理的智能化。发展智能电网，支持分布 　　　式能源的接入、居民和企业用电的智能管理。发展智能水务，构建覆盖供水全过程、保障供水质量安 　　　全的智能供排水和污水处理系统。发展智能管网，实现城市地下空间、地下管网的信息化管理和运行 　　　监控智能化。发展智能建筑，实现建筑设施、设备、节能、安全的智慧化管控。 |
| 04　公共服务便捷化 　　　建立跨部门跨地区业务协同、共建共享的公共服务信息服务体系。利用信息技术，创新发展城市教 　　　育、就业、社保、养老、医疗和文化的服务模式。 |
| 05　产业发展现代化 　　　加快传统产业信息化改造，推进制造模式向数字化、网络化、智能化、服务化转变。积极发展信息服 　　　务业，推动电子商务和物流信息化集成发展，创新并培育新型业态。 |
| 06　社会治理精细化 　　　在市场监管、环境监管、信用服务、应急保障、治安防控、公共安全等社会治理领域，深化信息应 　　　用，建立完善相关信息服务体系，创新社会治理方式。 |

| 专栏9　人文城市建设重点 |
| --- |
| 01　文化和自然遗产保护 　　　加强国家重大文化和自然遗产地、国家考古遗址公园、全国重点文物保护单位、历史文化名城名镇名 　　　村保护设施建设，加强城市重要历史建筑和历史文化街区保护，推进非物质文化遗产保护利用设施建 　　　设。 |
| 02　文化设施 　　　建设城市公共图书馆、文化馆、博物馆、美术馆等文化设施，每个社区配套建设文化活动设施，发展 　　　中小城市影剧院。 |
| 03　体育设施 　　　建设城市体育场（馆）和群众性户外体育健身场地，每个社区有便捷实用的体育健身设施。 |
| 04　休闲设施 　　　建设城市生态休闲公园、文化休闲街区、休闲步道、城郊休憩带。 |
| 05　公共设施免费开放 　　　逐步免费开放公共图书馆、文化馆（站）、博物馆、美术馆、纪念馆、科技馆、青少年宫和公益性城 　　　市公园。 |

 
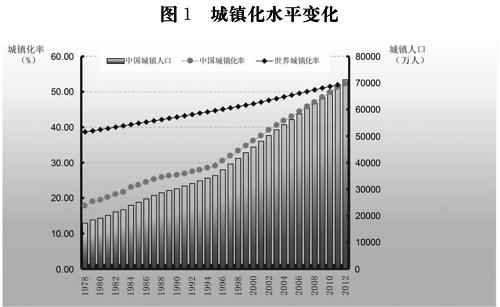


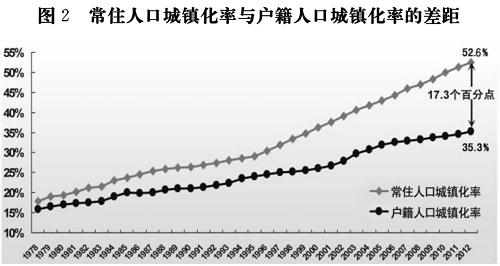


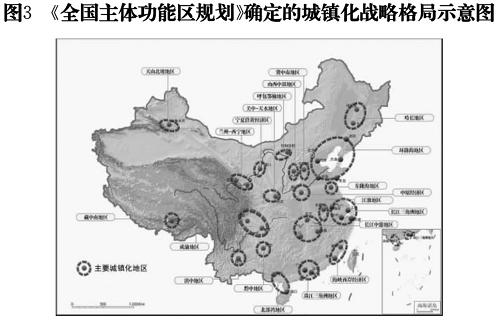


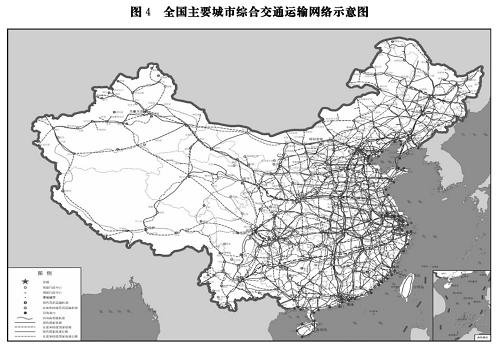


中共中央　国务院

（新华社北京2014年3月16日电）

**P5**

**Guidance on Promoting the Healthy Development of Smart Cities in China**

**关于促进智慧城市健康发展的指导意见**

发改高技〔2014〕1770号

智慧城市是运用物联网、云计算、大数据、空间地理信息集成等新一代信息技术，促进城市规划、建设、管理和服务智慧化的新理念和新模式。建设智慧城市，对加快工业化、信息化、城镇化、农业现代化融合，提升城市可持续发展能力具有重要意义。近年来，我国智慧城市建设取得了积极进展，但也暴露出缺乏顶层设计和统筹规划、体制机制创新滞后、网络安全隐患和风险突出等问题，一些地方出现思路不清、盲目建设的苗头，亟待加强引导。为贯彻落实《中共中央 国务院关于印发〈国家新型城镇化规划（2014-2020年）〉的通知》（中发〔2014〕4号）和《国务院关于促进信息消费扩大内需的若干意见》（国发〔2013〕32号）有关要求，促进智慧城市健康发展，经国务院同意，现提出以下意见。
　　一、指导思想、基本原则和主要目标
　　（一）指导思想。
　　按照走集约、智能、绿色、低碳的新型城镇化道路的总体要求，发挥市场在资源配置中的决定性作用，加强和完善政府引导，统筹物质、信息和智力资源，推动新一代信息技术创新应用，加强城市管理和服务体系智能化建设，积极发展民生服务智慧应用，强化网络安全保障，有效提高城市综合承载能力和居民幸福感受，促进城镇化发展质量和水平全面提升。
　　（二）基本原则。
　　以人为本，务实推进。智慧城市建设要突出为民、便民、惠民，推动创新城市管理和公共服务方式，向城市居民提供广覆盖、多层次、差异化、高质量的公共服务，避免重建设、轻实效，使公众分享智慧城市建设成果。
　　因地制宜，科学有序。以城市发展需求为导向，根据城市地理区位、历史文化、资源禀赋、产业特色、信息化基础等，应用先进适用技术科学推进智慧城市建设。在综合条件较好的区域或重点领域先行先试，有序推动智慧城市发展，避免贪大求全、重复建设。
　　市场为主，协同创新。积极探索智慧城市的发展路径、管理方式、推进模式和保障机制。鼓励建设和运营模式创新，注重激发市场活力，建立可持续发展机制。鼓励社会资本参与建设投资和运营，杜绝政府大包大揽和不必要的行政干预。
　　可管可控，确保安全。落实国家信息安全等级保护制度，强化网络和信息安全管理，落实责任机制，健全网络和信息安全标准体系，加大依法管理网络和保护个人信息的力度，加强要害信息系统和信息基础设施安全保障，确保安全可控。
　　（三）主要目标。
　　到2020年，建成一批特色鲜明的智慧城市，聚集和辐射带动作用大幅增强，综合竞争优势明显提高，在保障和改善民生服务、创新社会管理、维护网络安全等方面取得显著成效。
　　公共服务便捷化。在教育文化、医疗卫生、计划生育、劳动就业、社会保障、住房保障、环境保护、交通出行、防灾减灾、检验检测等公共服务领域，基本建成覆盖城乡居民、农民工及其随迁家属的信息服务体系，公众获取基本公共服务更加方便、及时、高效。
　　城市管理精细化。市政管理、人口管理、交通管理、公共安全、应急管理、社会诚信、市场监管、检验检疫、食品药品安全、饮用水安全等社会管理领域的信息化体系基本形成，统筹数字化城市管理信息系统、城市地理空间信息及建（构）筑物数据库等资源，实现城市规划和城市基础设施管理的数字化、精准化水平大幅提升，推动政府行政效能和城市管理水平大幅提升。
　　生活环境宜居化。居民生活数字化水平显著提高，水、大气、噪声、土壤和自然植被环境智能监测体系和污染物排放、能源消耗在线防控体系基本建成，促进城市人居环境得到改善。
　　基础设施智能化。宽带、融合、安全、泛在的下一代信息基础设施基本建成。电力、燃气、交通、水务、物流等公用基础设施的智能化水平大幅提升，运行管理实现精准化、协同化、一体化。工业化与信息化深度融合，信息服务业加快发展。
　　网络安全长效化。城市网络安全保障体系和管理制度基本建立，基础网络和要害信息系统安全可控，重要信息资源安全得到切实保障，居民、企业和政府的信息得到有效保护。
　　二、科学制定智慧城市建设顶层设计
　　（四）加强顶层设计。城市人民政府要从城市发展的战略全局出发研究制定智慧城市建设方案。方案要突出为人服务，深化重点领域智慧化应用，提供更加便捷、高效、低成本的社会服务；要明确推进信息资源共享和社会化开发利用、强化信息安全、保障信息准确可靠以及同步加强信用环境建设、完善法规标准等的具体措施；要加强与国民经济和社会发展总体规划、主体功能区规划、相关行业发展规划、区域规划、城乡规划以及有关专项规划的衔接，做好统筹城乡发展布局。
　　（五）推动构建普惠化公共服务体系。加快实施信息惠民工程。推进智慧医院、远程医疗建设，普及应用电子病历和健康档案，促进优质医疗资源纵向流动。建设具有随时看护、远程关爱等功能的养老信息化服务体系。建立公共就业信息服务平台，加快推进就业信息全国联网。加快社会保障经办信息化体系建设，推进医保费用跨市即时结算。推进社会保障卡、金融IC卡、市民服务卡、居民健康卡、交通卡等公共服务卡的应用集成和跨市一卡通用。围绕促进教育公平、提高教育质量和满足市民终身学习需求，建设完善教育信息化基础设施，构建利用信息化手段扩大优质教育资源覆盖面的有效机制，推进优质教育资源共享与服务。加强数字图书馆、数字档案馆、数字博物馆等公益设施建设。鼓励发展基于移动互联网的旅游服务系统和旅游管理信息平台。
　　（六）支撑建立精细化社会管理体系。建立全面设防、一体运作、精确定位、有效管控的社会治安防控体系。整合各类视频图像信息资源，推进公共安全视频联网应用。完善社会化、网络化、网格化的城乡公共安全保障体系，构建反应及时、恢复迅速、支援有力的应急保障体系。在食品药品、消费品安全、检验检疫等领域，建设完善具有溯源追查、社会监督等功能的市场监管信息服务体系，推进药品阳光采购。整合信贷、纳税、履约、产品质量、参保缴费和违法违纪等信用信息记录，加快征信信息系统建设。完善群众诉求表达和受理信访的网络平台，推进政府办事网上公开。
　　（七）促进宜居化生活环境建设。建立环境信息智能分析系统、预警应急系统和环境质量管理公共服务系统，对重点地区、重点企业和污染源实施智能化远程监测。依托城市统一公共服务信息平台建设社区公共服务信息系统，拓展社会管理和服务功能，发展面向家政、养老、社区照料和病患陪护的信息服务体系，为社区居民提供便捷的综合信息服务。推广智慧家庭，鼓励将医疗、教育、安防、政务等社会公共服务设施和服务资源接入家庭，提升家庭信息化服务水平。
　　（八）建立现代化产业发展体系。运用现代信息化手段，加快建立城市物流配送体系和城市消费需求与农产品供给紧密衔接的新型农业生产经营体系。加速工业化与信息化深度融合，推进大型工业企业深化信息技术的综合集成应用，建设完善中小企业公共信息服务平台，积极培育发展工业互联网等新兴业态。加快发展信息服务业，鼓励信息系统服务外包。建设完善电子商务基础设施，积极培育电子商务服务业，促进电子商务向旅游、餐饮、文化娱乐、家庭服务、养老服务、社区服务以及工业设计、文化创意等领域发展。
　　（九）加快建设智能化基础设施。加快构建城乡一体的宽带网络，推进下一代互联网和广播电视网建设，全面推广三网融合。推动城市公用设施、建筑等智能化改造，完善建筑数据库、房屋管理等信息系统和服务平台。加快智能电网建设。健全防灾减灾预报预警信息平台，建设全过程智能水务管理系统和饮用水安全电子监控系统。建设交通诱导、出行信息服务、公共交通、综合客运枢纽、综合运行协调指挥等智能系统，推进北斗导航卫星地基增强系统建设，发展差异化交通信息增值服务。建设智能物流信息平台和仓储式物流平台枢纽，加强港口、航运、陆运等物流信息的开发共享和社会化应用。
　　三、切实加大信息资源开发共享力度
　　（十）加快推进信息资源共享与更新。统筹城市地理空间信息及建（构）筑物数据库等资源，加快智慧城市公共信息平台和应用体系建设。建立促进信息共享的跨部门协调机制，完善信息更新机制，进一步加强政务部门信息共享和信息更新管理。各政务部门应根据职能分工，将本部门建设管理的信息资源授权有需要的部门无偿使用，共享部门应按授权范围合理使用信息资源。以城市统一的地理空间框架和人口、法人等信息资源为基础，叠加各部门、各行业相关业务信息，加快促进跨部门协同应用。整合已建政务信息系统，统筹新建系统，建设信息资源共享设施，实现基础信息资源和业务信息资源的集约化采集、网络化汇聚和统一化管理。
　　（十一）深化重点领域信息资源开发利用。城市人民政府要将提高信息资源开发利用水平作为提升城市综合竞争力的重要手段，大力推动政府部门将企业信用、产品质量、食品药品安全、综合交通、公用设施、环境质量等信息资源向社会开放，鼓励市政公用企事业单位、公共服务事业单位等机构将教育、医疗、就业、旅游、生活等信息资源向社会开放。支持社会力量应用信息资源发展便民、惠民、实用的新型信息服务。鼓励发展以信息知识加工和创新为主的数据挖掘、商业分析等新型服务，加速信息知识向产品、资产及效益转化。
　　四、积极运用新技术新业态
　　（十二）加快重点领域物联网应用。支持物联网在高耗能行业的应用，促进生产制造、经营管理和能源利用智能化。鼓励物联网在农产品生产流通等领域应用。加快物联网在城市管理、交通运输、节能减排、食品药品安全、社会保障、医疗卫生、民生服务、公共安全、产品质量等领域的推广应用，提高城市管理精细化水平，逐步形成全面感知、广泛互联的城市智能管理和服务体系。
　　（十三）促进云计算和大数据健康发展。鼓励电子政务系统向云计算模式迁移。在教育、医疗卫生、劳动就业、社会保障等重点民生领域，推广低成本、高质量、广覆盖的云服务，支持各类企业充分利用公共云计算服务资源。加强基于云计算的大数据开发与利用，在电子商务、工业设计、科学研究、交通运输等领域，创新大数据商业模式，服务城市经济社会发展。
　　（十四）推动信息技术集成应用。面向公众实际需要，重点在交通运输联程联运、城市共同配送、灾害防范与应急处置、家居智能管理、居家看护与健康管理、集中养老与远程医疗、智能建筑与智慧社区、室内外统一位置服务、旅游娱乐消费等领域，加强移动互联网、遥感遥测、北斗导航、地理信息等技术的集成应用，创新服务模式，为城市居民提供方便、实用的新型服务。
　　五、着力加强网络信息安全管理和能力建设
　　（十五）严格全流程网络安全管理。城市人民政府在推进智慧城市建设中要同步加强网络安全保障工作。在重要信息系统设计阶段，要合理确定安全保护等级，同步设计安全防护方案；在实施阶段，要加强对技术、设备和服务提供商的安全审查，同步建设安全防护手段；在运行阶段，要加强管理，定期开展检查、等级评测和风险评估，认真排查安全风险隐患，增强日常监测和应急响应处置恢复能力。
　　（十六）加强要害信息设施和信息资源安全防护。加大对党政军、金融、能源、交通、电信、公共安全、公用事业等重要信息系统和涉密信息系统的安全防护，确保安全可控。完善网络安全设施，重点提高网络管理、态势预警、应急处理和信任服务能力。统筹建设容灾备份体系，推行联合灾备和异地灾备。建立重要信息使用管理和安全评价机制。严格落实国家有关法律法规及标准，加强行业和企业自律，切实加强个人信息保护。
　　（十七）强化安全责任和安全意识。建立网络安全责任制，明确城市人民政府及有关部门负责人、要害信息系统运营单位负责人的网络信息安全责任，建立责任追究机制。加大宣传教育力度，提高智慧城市规划、建设、管理、维护等各环节工作人员的网络信息安全风险意识、责任意识、工作技能和管理水平。鼓励发展专业化、社会化的信息安全认证服务，为保障智慧城市网络信息安全提供支持。
　　六、完善组织管理和制度建设
　　（十八）完善管理制度。国务院有关部门要加快研究制定智慧城市建设的标准体系、评价体系和审计监督体系，推行智慧城市重点工程项目风险和效益评估机制，定期公布智慧城市建设重点任务完成进展情况。城市人民政府要健全智慧城市建设重大项目监督听证制度和问责机制，将智慧城市建设成效纳入政府绩效考核体系；建立激励约束机制，推动电子政务和公益性信息服务外包和利用社会力量开发利用信息资源、发展便民信息服务。
　　（十九）完善投融资机制。在国务院批准发行的地方政府债券额度内，各省级人民政府要统筹安排部分资金用于智慧城市建设。城市人民政府要建立规范的投融资机制，通过特许经营、购买服务等多种形式，引导社会资金参与智慧城市建设，鼓励符合条件的企业发行企业债募集资金开展智慧城市建设，严禁以建设智慧城市名义变相推行土地财政和不切实际的举债融资。城市有关财政资金要重点投向基础性、公益性领域，优先支持涉及民生的智慧应用，鼓励市政公用企事业单位对市政设施进行智能化改造。
　　各地区、各有关部门要充分认识促进智慧城市健康发展的重要意义，切实加强组织领导，认真落实本指导意见提出的各项任务。发展改革委、工业和信息化部、科技部、公安部、财政部、国土资源部、环境保护部、住房城乡建设部、交通运输部等要建立部际协调机制，协调解决智慧城市建设中的重大问题，加强对各地区的指导和监督，研究出台促进智慧城市健康发展以及信息化促进城镇化发展的相关政策。各省级人民政府要切实加强对本地区智慧城市建设的领导，采取有力措施，抓好全过程监督管理。城市人民政府是智慧城市建设的责任主体，要加强组织，细化措施，扎实推进各项工作，主动接受社会监督，确保智慧城市建设健康有序推进。

发 展 改 革 委　　工业和信息化部　　　　　
科 学 技 术 部　　公　　安　　部　　　　　
财  　政 　 部　　国 土 资 源 部　　　　　
住房城乡建设部　　交 通 运 输 部　　　　　
2014年8月27日

**P6**

**Made in China 2025**

**中国制造2025**

国发〔2015〕28号

制造业是国民经济的主体，是立国之本、兴国之器、强国之基。十八世纪中叶开启工业文明以来，世界强国的兴衰史和中华民族的奋斗史一再证明，没有强大的制造业，就没有国家和民族的强盛。打造具有国际竞争力的制造业，是我国提升综合国力、保障国家安全、建设世界强国的必由之路。
　　新中国成立尤其是改革开放以来，我国制造业持续快速发展，建成了门类齐全、独立完整的产业体系，有力推动工业化和现代化进程，显著增强综合国力，支撑我世界大国地位。然而，与世界先进水平相比，我国制造业仍然大而不强，在自主创新能力、资源利用效率、产业结构水平、信息化程度、质量效益等方面差距明显，转型升级和跨越发展的任务紧迫而艰巨。
　　当前，新一轮科技革命和产业变革与我国加快转变经济发展方式形成历史性交汇，国际产业分工格局正在重塑。必须紧紧抓住这一重大历史机遇，按照“四个全面”战略布局要求，实施制造强国战略，加强统筹规划和前瞻部署，力争通过三个十年的努力，到新中国成立一百年时，把我国建设成为引领世界制造业发展的制造强国，为实现中华民族伟大复兴的中国梦打下坚实基础。
　　《中国制造2025》，是我国实施制造强国战略第一个十年的行动纲领。
　　一、发展形势和环境
　　（一）全球制造业格局面临重大调整。
　　新一代信息技术与制造业深度融合，正在引发影响深远的产业变革，形成新的生产方式、产业形态、商业模式和经济增长点。各国都在加大科技创新力度，推动三维（3D）打印、移动互联网、云计算、大数据、生物工程、新能源、新材料等领域取得新突破。基于信息物理系统的智能装备、智能工厂等智能制造正在引领制造方式变革；网络众包、协同设计、大规模个性化定制、精准供应链管理、全生命周期管理、电子商务等正在重塑产业价值链体系；可穿戴智能产品、智能家电、智能汽车等智能终端产品不断拓展制造业新领域。我国制造业转型升级、创新发展迎来重大机遇。
　　全球产业竞争格局正在发生重大调整，我国在新一轮发展中面临巨大挑战。国际金融危机发生后，发达国家纷纷实施“再工业化”战略，重塑制造业竞争新优势，加速推进新一轮全球贸易投资新格局。一些发展中国家也在加快谋划和布局，积极参与全球产业再分工，承接产业及资本转移，拓展国际市场空间。我国制造业面临发达国家和其他发展中国家“双向挤压”的严峻挑战，必须放眼全球，加紧战略部署，着眼建设制造强国，固本培元，化挑战为机遇，抢占制造业新一轮竞争制高点。
　　（二）我国经济发展环境发生重大变化。
　　随着新型工业化、信息化、城镇化、农业现代化同步推进，超大规模内需潜力不断释放，为我国制造业发展提供了广阔空间。各行业新的装备需求、人民群众新的消费需求、社会管理和公共服务新的民生需求、国防建设新的安全需求，都要求制造业在重大技术装备创新、消费品质量和安全、公共服务设施设备供给和国防装备保障等方面迅速提升水平和能力。全面深化改革和进一步扩大开放，将不断激发制造业发展活力和创造力，促进制造业转型升级。
　　我国经济发展进入新常态，制造业发展面临新挑战。资源和环境约束不断强化，劳动力等生产要素成本不断上升，投资和出口增速明显放缓，主要依靠资源要素投入、规模扩张的粗放发展模式难以为继，调整结构、转型升级、提质增效刻不容缓。形成经济增长新动力，塑造国际竞争新优势，重点在制造业，难点在制造业，出路也在制造业。
　　（三）建设制造强国任务艰巨而紧迫。
　　经过几十年的快速发展，我国制造业规模跃居世界第一位，建立起门类齐全、独立完整的制造体系，成为支撑我国经济社会发展的重要基石和促进世界经济发展的重要力量。持续的技术创新，大大提高了我国制造业的综合竞争力。载人航天、载人深潜、大型飞机、北斗卫星导航、超级计算机、高铁装备、百万千瓦级发电装备、万米深海石油钻探设备等一批重大技术装备取得突破，形成了若干具有国际竞争力的优势产业和骨干企业，我国已具备了建设工业强国的基础和条件。
　　但我国仍处于工业化进程中，与先进国家相比还有较大差距。制造业大而不强，自主创新能力弱，关键核心技术与高端装备对外依存度高，以企业为主体的制造业创新体系不完善；产品档次不高，缺乏世界知名品牌；资源能源利用效率低，环境污染问题较为突出；产业结构不合理，高端装备制造业和生产性服务业发展滞后；信息化水平不高，与工业化融合深度不够；产业国际化程度不高，企业全球化经营能力不足。推进制造强国建设，必须着力解决以上问题。
　　建设制造强国，必须紧紧抓住当前难得的战略机遇，积极应对挑战，加强统筹规划，突出创新驱动，制定特殊政策，发挥制度优势，动员全社会力量奋力拼搏，更多依靠中国装备、依托中国品牌，实现中国制造向中国创造的转变，中国速度向中国质量的转变，中国产品向中国品牌的转变，完成中国制造由大变强的战略任务。
　　二、战略方针和目标
　　（一）指导思想。
　　全面贯彻党的十八大和十八届二中、三中、四中全会精神，坚持走中国特色新型工业化道路，以促进制造业创新发展为主题，以提质增效为中心，以加快新一代信息技术与制造业深度融合为主线，以推进智能制造为主攻方向，以满足经济社会发展和国防建设对重大技术装备的需求为目标，强化工业基础能力，提高综合集成水平，完善多层次多类型人才培养体系，促进产业转型升级，培育有中国特色的制造文化，实现制造业由大变强的历史跨越。基本方针是：
　　——创新驱动。坚持把创新摆在制造业发展全局的核心位置，完善有利于创新的制度环境，推动跨领域跨行业协同创新，突破一批重点领域关键共性技术，促进制造业数字化网络化智能化，走创新驱动的发展道路。
　　——质量为先。坚持把质量作为建设制造强国的生命线，强化企业质量主体责任，加强质量技术攻关、自主品牌培育。建设法规标准体系、质量监管体系、先进质量文化，营造诚信经营的市场环境，走以质取胜的发展道路。
　　——绿色发展。坚持把可持续发展作为建设制造强国的重要着力点，加强节能环保技术、工艺、装备推广应用，全面推行清洁生产。发展循环经济，提高资源回收利用效率，构建绿色制造体系，走生态文明的发展道路。
　　——结构优化。坚持把结构调整作为建设制造强国的关键环节，大力发展先进制造业，改造提升传统产业，推动生产型制造向服务型制造转变。优化产业空间布局，培育一批具有核心竞争力的产业集群和企业群体，走提质增效的发展道路。
　　——人才为本。坚持把人才作为建设制造强国的根本，建立健全科学合理的选人、用人、育人机制，加快培养制造业发展急需的专业技术人才、经营管理人才、技能人才。营造大众创业、万众创新的氛围，建设一支素质优良、结构合理的制造业人才队伍，走人才引领的发展道路。
　　（二）基本原则。
　　市场主导，政府引导。全面深化改革，充分发挥市场在资源配置中的决定性作用，强化企业主体地位，激发企业活力和创造力。积极转变政府职能，加强战略研究和规划引导，完善相关支持政策，为企业发展创造良好环境。
　　立足当前，着眼长远。针对制约制造业发展的瓶颈和薄弱环节，加快转型升级和提质增效，切实提高制造业的核心竞争力和可持续发展能力。准确把握新一轮科技革命和产业变革趋势，加强战略谋划和前瞻部署，扎扎实实打基础，在未来竞争中占据制高点。
　　整体推进，重点突破。坚持制造业发展全国一盘棋和分类指导相结合，统筹规划，合理布局，明确创新发展方向，促进军民融合深度发展，加快推动制造业整体水平提升。围绕经济社会发展和国家安全重大需求，整合资源，突出重点，实施若干重大工程，实现率先突破。
　　自主发展，开放合作。在关系国计民生和产业安全的基础性、战略性、全局性领域，着力掌握关键核心技术，完善产业链条，形成自主发展能力。继续扩大开放，积极利用全球资源和市场，加强产业全球布局和国际交流合作，形成新的比较优势，提升制造业开放发展水平。
　　（三）战略目标。
　　立足国情，立足现实，力争通过“三步走”实现制造强国的战略目标。
　　第一步：力争用十年时间，迈入制造强国行列。
　　到2020年，基本实现工业化，制造业大国地位进一步巩固，制造业信息化水平大幅提升。掌握一批重点领域关键核心技术，优势领域竞争力进一步增强，产品质量有较大提高。制造业数字化、网络化、智能化取得明显进展。重点行业单位工业增加值能耗、物耗及污染物排放明显下降。
　　到2025年，制造业整体素质大幅提升，创新能力显著增强，全员劳动生产率明显提高，两化（工业化和信息化）融合迈上新台阶。重点行业单位工业增加值能耗、物耗及污染物排放达到世界先进水平。形成一批具有较强国际竞争力的跨国公司和产业集群，在全球产业分工和价值链中的地位明显提升。
　　第二步：到2035年，我国制造业整体达到世界制造强国阵营中等水平。创新能力大幅提升，重点领域发展取得重大突破，整体竞争力明显增强，优势行业形成全球创新引领能力，全面实现工业化。
　　第三步：新中国成立一百年时，制造业大国地位更加巩固，综合实力进入世界制造强国前列。制造业主要领域具有创新引领能力和明显竞争优势，建成全球领先的技术体系和产业体系。

2020年和2025年制造业主要指标

| 类别 | 指　　标 | 2013年 | 2015年 | 2020年 | 2025年 |
| --- | --- | --- | --- | --- | --- |
| 创新能力 | 规模以上制造业研发经费内部支出占主营业务收入比重（%） | 0.88 | 0.95 | 1.26 | 1.68 |
|  | 规模以上制造业每亿元主营业务收入有效发明专利数1（件） | 0.36 | 0.44 | 0.70 | 1.10 |
| 质量效益 | 制造业质量竞争力指数2 | 83.1 | 83.5 | 84.5 | 85.5 |
|  | 制造业增加值率提高 | - | - | 比2015年提高2个百分点 | 比2015年提高4个百分点 |
|  | 制造业全员劳动生产率增速（%） | - | - | 7.5左右（“十三五”期间年均增速） | 6.5左右（“十四五”期间年均增速） |
| 两化融合 | 宽带普及率3（%） | 37 | 50 | 70 | 82 |
|  | 数字化研发设计工具普及率4（%） | 52 | 58 | 72 | 84 |
|  | 关键工序数控化率5（%） | 27 | 33 | 50 | 64 |
| 绿色发展 | 规模以上单位工业增加值能耗下降幅度 | - | - | 比2015年下降18% | 比2015年下降34% |
|  | 单位工业增加值二氧化碳排放量下降幅度 | - | - | 比2015年下降22% | 比2015年下降40% |
|  | 单位工业增加值用水量下降幅度 | - | - | 比2015年下降23% | 比2015年下降41% |
|  | 工业固体废物综合利用率（%） | 62 | 65 | 73 | 79 |

　　1 规模以上制造业每亿元主营业务收入有效发明专利数=规模以上制造企业有效发明专利数/规模以上制造企业主营业务收入。
　　2 制造业质量竞争力指数是反映我国制造业质量整体水平的经济技术综合指标，由质量水平和发展能力两个方面共计12项具体指标计算得出。
　　3 宽带普及率用固定宽带家庭普及率代表，固定宽带家庭普及率=固定宽带家庭用户数/家庭户数。
　　4 数字化研发设计工具普及率=应用数字化研发设计工具的规模以上企业数量/规模以上企业总数量（相关数据来源于3万家样本企业，下同）。
　　5 关键工序数控化率为规模以上工业企业关键工序数控化率的平均值。

　　三、战略任务和重点
　　实现制造强国的战略目标，必须坚持问题导向，统筹谋划，突出重点；必须凝聚全社会共识，加快制造业转型升级，全面提高发展质量和核心竞争力。
　　（一）提高国家制造业创新能力。
　　完善以企业为主体、市场为导向、政产学研用相结合的制造业创新体系。围绕产业链部署创新链，围绕创新链配置资源链，加强关键核心技术攻关，加速科技成果产业化，提高关键环节和重点领域的创新能力。
　　加强关键核心技术研发。强化企业技术创新主体地位，支持企业提升创新能力，推进国家技术创新示范企业和企业技术中心建设，充分吸纳企业参与国家科技计划的决策和实施。瞄准国家重大战略需求和未来产业发展制高点，定期研究制定发布制造业重点领域技术创新路线图。继续抓紧实施国家科技重大专项，通过国家科技计划（专项、基金等）支持关键核心技术研发。发挥行业骨干企业的主导作用和高等院校、科研院所的基础作用，建立一批产业创新联盟，开展政产学研用协同创新，攻克一批对产业竞争力整体提升具有全局性影响、带动性强的关键共性技术，加快成果转化。
　　提高创新设计能力。在传统制造业、战略性新兴产业、现代服务业等重点领域开展创新设计示范，全面推广应用以绿色、智能、协同为特征的先进设计技术。加强设计领域共性关键技术研发，攻克信息化设计、过程集成设计、复杂过程和系统设计等共性技术，开发一批具有自主知识产权的关键设计工具软件，建设完善创新设计生态系统。建设若干具有世界影响力的创新设计集群，培育一批专业化、开放型的工业设计企业，鼓励代工企业建立研究设计中心，向代设计和出口自主品牌产品转变。发展各类创新设计教育，设立国家工业设计奖，激发全社会创新设计的积极性和主动性。
　　推进科技成果产业化。完善科技成果转化运行机制，研究制定促进科技成果转化和产业化的指导意见，建立完善科技成果信息发布和共享平台，健全以技术交易市场为核心的技术转移和产业化服务体系。完善科技成果转化激励机制，推动事业单位科技成果使用、处置和收益管理改革，健全科技成果科学评估和市场定价机制。完善科技成果转化协同推进机制，引导政产学研用按照市场规律和创新规律加强合作，鼓励企业和社会资本建立一批从事技术集成、熟化和工程化的中试基地。加快国防科技成果转化和产业化进程，推进军民技术双向转移转化。
　　完善国家制造业创新体系。加强顶层设计，加快建立以创新中心为核心载体、以公共服务平台和工程数据中心为重要支撑的制造业创新网络，建立市场化的创新方向选择机制和鼓励创新的风险分担、利益共享机制。充分利用现有科技资源，围绕制造业重大共性需求，采取政府与社会合作、政产学研用产业创新战略联盟等新机制新模式，形成一批制造业创新中心（工业技术研究基地），开展关键共性重大技术研究和产业化应用示范。建设一批促进制造业协同创新的公共服务平台，规范服务标准，开展技术研发、检验检测、技术评价、技术交易、质量认证、人才培训等专业化服务，促进科技成果转化和推广应用。建设重点领域制造业工程数据中心，为企业提供创新知识和工程数据的开放共享服务。面向制造业关键共性技术，建设一批重大科学研究和实验设施，提高核心企业系统集成能力，促进向价值链高端延伸。

| 专栏1　制造业创新中心（工业技术研究基地）建设工程 |
| --- |
| 围绕重点行业转型升级和新一代信息技术、智能制造、增材制造、新材料、生物医药等领域创新发展的重大共性需求，形成一批制造业创新中心（工业技术研究基地），重点开展行业基础和共性关键技术研发、成果产业化、人才培训等工作。制定完善制造业创新中心遴选、考核、管理的标准和程序。 　　到2020年，重点形成15家左右制造业创新中心（工业技术研究基地），力争到2025年形成40家左右制造业创新中心（工业技术研究基地）。 |

　　加强标准体系建设。改革标准体系和标准化管理体制，组织实施制造业标准化提升计划，在智能制造等重点领域开展综合标准化工作。发挥企业在标准制定中的重要作用，支持组建重点领域标准推进联盟，建设标准创新研究基地，协同推进产品研发与标准制定。制定满足市场和创新需要的团体标准，建立企业产品和服务标准自我声明公开和监督制度。鼓励和支持企业、科研院所、行业组织等参与国际标准制定，加快我国标准国际化进程。大力推动国防装备采用先进的民用标准，推动军用技术标准向民用领域的转化和应用。做好标准的宣传贯彻，大力推动标准实施。
　　强化知识产权运用。加强制造业重点领域关键核心技术知识产权储备，构建产业化导向的专利组合和战略布局。鼓励和支持企业运用知识产权参与市场竞争，培育一批具备知识产权综合实力的优势企业，支持组建知识产权联盟，推动市场主体开展知识产权协同运用。稳妥推进国防知识产权解密和市场化应用。建立健全知识产权评议机制，鼓励和支持行业骨干企业与专业机构在重点领域合作开展专利评估、收购、运营、风险预警与应对。构建知识产权综合运用公共服务平台。鼓励开展跨国知识产权许可。研究制定降低中小企业知识产权申请、保护及维权成本的政策措施。
　　（二）推进信息化与工业化深度融合。
　　加快推动新一代信息技术与制造技术融合发展，把智能制造作为两化深度融合的主攻方向；着力发展智能装备和智能产品，推进生产过程智能化，培育新型生产方式，全面提升企业研发、生产、管理和服务的智能化水平。
　　研究制定智能制造发展战略。编制智能制造发展规划，明确发展目标、重点任务和重大布局。加快制定智能制造技术标准，建立完善智能制造和两化融合管理标准体系。强化应用牵引，建立智能制造产业联盟，协同推动智能装备和产品研发、系统集成创新与产业化。促进工业互联网、云计算、大数据在企业研发设计、生产制造、经营管理、销售服务等全流程和全产业链的综合集成应用。加强智能制造工业控制系统网络安全保障能力建设，健全综合保障体系。
　　加快发展智能制造装备和产品。组织研发具有深度感知、智慧决策、自动执行功能的高档数控机床、工业机器人、增材制造装备等智能制造装备以及智能化生产线，突破新型传感器、智能测量仪表、工业控制系统、伺服电机及驱动器和减速器等智能核心装置，推进工程化和产业化。加快机械、航空、船舶、汽车、轻工、纺织、食品、电子等行业生产设备的智能化改造，提高精准制造、敏捷制造能力。统筹布局和推动智能交通工具、智能工程机械、服务机器人、智能家电、智能照明电器、可穿戴设备等产品研发和产业化。
　　推进制造过程智能化。在重点领域试点建设智能工厂/数字化车间，加快人机智能交互、工业机器人、智能物流管理、增材制造等技术和装备在生产过程中的应用，促进制造工艺的仿真优化、数字化控制、状态信息实时监测和自适应控制。加快产品全生命周期管理、客户关系管理、供应链管理系统的推广应用，促进集团管控、设计与制造、产供销一体、业务和财务衔接等关键环节集成，实现智能管控。加快民用爆炸物品、危险化学品、食品、印染、稀土、农药等重点行业智能检测监管体系建设，提高智能化水平。
　　深化互联网在制造领域的应用。制定互联网与制造业融合发展的路线图，明确发展方向、目标和路径。发展基于互联网的个性化定制、众包设计、云制造等新型制造模式，推动形成基于消费需求动态感知的研发、制造和产业组织方式。建立优势互补、合作共赢的开放型产业生态体系。加快开展物联网技术研发和应用示范，培育智能监测、远程诊断管理、全产业链追溯等工业互联网新应用。实施工业云及工业大数据创新应用试点，建设一批高质量的工业云服务和工业大数据平台，推动软件与服务、设计与制造资源、关键技术与标准的开放共享。
　　加强互联网基础设施建设。加强工业互联网基础设施建设规划与布局，建设低时延、高可靠、广覆盖的工业互联网。加快制造业集聚区光纤网、移动通信网和无线局域网的部署和建设，实现信息网络宽带升级，提高企业宽带接入能力。针对信息物理系统网络研发及应用需求，组织开发智能控制系统、工业应用软件、故障诊断软件和相关工具、传感和通信系统协议，实现人、设备与产品的实时联通、精确识别、有效交互与智能控制。

| 专栏2　智能制造工程 |
| --- |
| 紧密围绕重点制造领域关键环节，开展新一代信息技术与制造装备融合的集成创新和工程应用。支持政产学研用联合攻关，开发智能产品和自主可控的智能装置并实现产业化。依托优势企业，紧扣关键工序智能化、关键岗位机器人替代、生产过程智能优化控制、供应链优化，建设重点领域智能工厂/数字化车间。在基础条件好、需求迫切的重点地区、行业和企业中，分类实施流程制造、离散制造、智能装备和产品、新业态新模式、智能化管理、智能化服务等试点示范及应用推广。建立智能制造标准体系和信息安全保障系统，搭建智能制造网络系统平台。 　　到2020年，制造业重点领域智能化水平显著提升，试点示范项目运营成本降低30%，产品生产周期缩短30%，不良品率降低30%。到2025年，制造业重点领域全面实现智能化，试点示范项目运营成本降低50%，产品生产周期缩短50%，不良品率降低50%。 |

　　（三）强化工业基础能力。
　　核心基础零部件（元器件）、先进基础工艺、关键基础材料和产业技术基础（以下统称“四基”）等工业基础能力薄弱，是制约我国制造业创新发展和质量提升的症结所在。要坚持问题导向、产需结合、协同创新、重点突破的原则，着力破解制约重点产业发展的瓶颈。
　　统筹推进“四基”发展。制定工业强基实施方案，明确重点方向、主要目标和实施路径。制定工业“四基”发展指导目录，发布工业强基发展报告，组织实施工业强基工程。统筹军民两方面资源，开展军民两用技术联合攻关，支持军民技术相互有效利用，促进基础领域融合发展。强化基础领域标准、计量体系建设，加快实施对标达标，提升基础产品的质量、可靠性和寿命。建立多部门协调推进机制，引导各类要素向基础领域集聚。
　　加强“四基”创新能力建设。强化前瞻性基础研究，着力解决影响核心基础零部件（元器件）产品性能和稳定性的关键共性技术。建立基础工艺创新体系，利用现有资源建立关键共性基础工艺研究机构，开展先进成型、加工等关键制造工艺联合攻关；支持企业开展工艺创新，培养工艺专业人才。加大基础专用材料研发力度，提高专用材料自给保障能力和制备技术水平。建立国家工业基础数据库，加强企业试验检测数据和计量数据的采集、管理、应用和积累。加大对“四基”领域技术研发的支持力度，引导产业投资基金和创业投资基金投向“四基”领域重点项目。
　　推动整机企业和“四基”企业协同发展。注重需求侧激励，产用结合，协同攻关。依托国家科技计划（专项、基金等）和相关工程等，在数控机床、轨道交通装备、航空航天、发电设备等重点领域，引导整机企业和“四基”企业、高校、科研院所产需对接，建立产业联盟，形成协同创新、产用结合、以市场促基础产业发展的新模式，提升重大装备自主可控水平。开展工业强基示范应用，完善首台（套）、首批次政策，支持核心基础零部件（元器件）、先进基础工艺、关键基础材料推广应用。

| 专栏3　工业强基工程 |
| --- |
| 开展示范应用，建立奖励和风险补偿机制，支持核心基础零部件（元器件）、先进基础工艺、关键基础材料的首批次或跨领域应用。组织重点突破，针对重大工程和重点装备的关键技术和产品急需，支持优势企业开展政产学研用联合攻关，突破关键基础材料、核心基础零部件的工程化、产业化瓶颈。强化平台支撑，布局和组建一批"四基"研究中心，创建一批公共服务平台，完善重点产业技术基础体系。 　　到2020年，40%的核心基础零部件、关键基础材料实现自主保障，受制于人的局面逐步缓解，航天装备、通信装备、发电与输变电设备、工程机械、轨道交通装备、家用电器等产业急需的核心基础零部件（元器件）和关键基础材料的先进制造工艺得到推广应用。到2025年，70%的核心基础零部件、关键基础材料实现自主保障，80种标志性先进工艺得到推广应用，部分达到国际领先水平，建成较为完善的产业技术基础服务体系，逐步形成整机牵引和基础支撑协调互动的产业创新发展格局。 |

　　（四）加强质量品牌建设。
　　提升质量控制技术，完善质量管理机制，夯实质量发展基础，优化质量发展环境，努力实现制造业质量大幅提升。鼓励企业追求卓越品质，形成具有自主知识产权的名牌产品，不断提升企业品牌价值和中国制造整体形象。
　　推广先进质量管理技术和方法。建设重点产品标准符合性认定平台，推动重点产品技术、安全标准全面达到国际先进水平。开展质量标杆和领先企业示范活动，普及卓越绩效、六西格玛、精益生产、质量诊断、质量持续改进等先进生产管理模式和方法。支持企业提高质量在线监测、在线控制和产品全生命周期质量追溯能力。组织开展重点行业工艺优化行动，提升关键工艺过程控制水平。开展质量管理小组、现场改进等群众性质量管理活动示范推广。加强中小企业质量管理，开展质量安全培训、诊断和辅导活动。
　　加快提升产品质量。实施工业产品质量提升行动计划，针对汽车、高档数控机床、轨道交通装备、大型成套技术装备、工程机械、特种设备、关键原材料、基础零部件、电子元器件等重点行业，组织攻克一批长期困扰产品质量提升的关键共性质量技术，加强可靠性设计、试验与验证技术开发应用，推广采用先进成型和加工方法、在线检测装置、智能化生产和物流系统及检测设备等，使重点实物产品的性能稳定性、质量可靠性、环境适应性、使用寿命等指标达到国际同类产品先进水平。在食品、药品、婴童用品、家电等领域实施覆盖产品全生命周期的质量管理、质量自我声明和质量追溯制度，保障重点消费品质量安全。大力提高国防装备质量可靠性，增强国防装备实战能力。
　　完善质量监管体系。健全产品质量标准体系、政策规划体系和质量管理法律法规。加强关系民生和安全等重点领域的行业准入与市场退出管理。建立消费品生产经营企业产品事故强制报告制度，健全质量信用信息收集和发布制度，强化企业质量主体责任。将质量违法违规记录作为企业诚信评级的重要内容，建立质量黑名单制度，加大对质量违法和假冒品牌行为的打击和惩处力度。建立区域和行业质量安全预警制度，防范化解产品质量安全风险。严格实施产品“三包”、产品召回等制度。强化监管检查和责任追究，切实保护消费者权益。
　　夯实质量发展基础。制定和实施与国际先进水平接轨的制造业质量、安全、卫生、环保及节能标准。加强计量科技基础及前沿技术研究，建立一批制造业发展急需的高准确度、高稳定性计量基标准，提升与制造业相关的国家量传溯源能力。加强国家产业计量测试中心建设，构建国家计量科技创新体系。完善检验检测技术保障体系，建设一批高水平的工业产品质量控制和技术评价实验室、产品质量监督检验中心，鼓励建立专业检测技术联盟。完善认证认可管理模式，提高强制性产品认证的有效性，推动自愿性产品认证健康发展，提升管理体系认证水平，稳步推进国际互认。支持行业组织发布自律规范或公约，开展质量信誉承诺活动。
　　推进制造业品牌建设。引导企业制定品牌管理体系，围绕研发创新、生产制造、质量管理和营销服务全过程，提升内在素质，夯实品牌发展基础。扶持一批品牌培育和运营专业服务机构，开展品牌管理咨询、市场推广等服务。健全集体商标、证明商标注册管理制度。打造一批特色鲜明、竞争力强、市场信誉好的产业集群区域品牌。建设品牌文化，引导企业增强以质量和信誉为核心的品牌意识，树立品牌消费理念，提升品牌附加值和软实力。加速我国品牌价值评价国际化进程，充分发挥各类媒体作用，加大中国品牌宣传推广力度，树立中国制造品牌良好形象。
　　（五）全面推行绿色制造。
　　加大先进节能环保技术、工艺和装备的研发力度，加快制造业绿色改造升级；积极推行低碳化、循环化和集约化，提高制造业资源利用效率；强化产品全生命周期绿色管理，努力构建高效、清洁、低碳、循环的绿色制造体系。
　　加快制造业绿色改造升级。全面推进钢铁、有色、化工、建材、轻工、印染等传统制造业绿色改造，大力研发推广余热余压回收、水循环利用、重金属污染减量化、有毒有害原料替代、废渣资源化、脱硫脱硝除尘等绿色工艺技术装备，加快应用清洁高效铸造、锻压、焊接、表面处理、切削等加工工艺，实现绿色生产。加强绿色产品研发应用，推广轻量化、低功耗、易回收等技术工艺，持续提升电机、锅炉、内燃机及电器等终端用能产品能效水平，加快淘汰落后机电产品和技术。积极引领新兴产业高起点绿色发展，大幅降低电子信息产品生产、使用能耗及限用物质含量，建设绿色数据中心和绿色基站，大力促进新材料、新能源、高端装备、生物产业绿色低碳发展。
　　推进资源高效循环利用。支持企业强化技术创新和管理，增强绿色精益制造能力，大幅降低能耗、物耗和水耗水平。持续提高绿色低碳能源使用比率，开展工业园区和企业分布式绿色智能微电网建设，控制和削减化石能源消费量。全面推行循环生产方式，促进企业、园区、行业间链接共生、原料互供、资源共享。推进资源再生利用产业规范化、规模化发展，强化技术装备支撑，提高大宗工业固体废弃物、废旧金属、废弃电器电子产品等综合利用水平。大力发展再制造产业，实施高端再制造、智能再制造、在役再制造，推进产品认定，促进再制造产业持续健康发展。
　　积极构建绿色制造体系。支持企业开发绿色产品，推行生态设计，显著提升产品节能环保低碳水平，引导绿色生产和绿色消费。建设绿色工厂，实现厂房集约化、原料无害化、生产洁净化、废物资源化、能源低碳化。发展绿色园区，推进工业园区产业耦合，实现近零排放。打造绿色供应链，加快建立以资源节约、环境友好为导向的采购、生产、营销、回收及物流体系，落实生产者责任延伸制度。壮大绿色企业，支持企业实施绿色战略、绿色标准、绿色管理和绿色生产。强化绿色监管，健全节能环保法规、标准体系，加强节能环保监察，推行企业社会责任报告制度，开展绿色评价。

| 专栏4　绿色制造工程 |
| --- |
| 组织实施传统制造业能效提升、清洁生产、节水治污、循环利用等专项技术改造。开展重大节能环保、资源综合利用、再制造、低碳技术产业化示范。实施重点区域、流域、行业清洁生产水平提升计划，扎实推进大气、水、土壤污染源头防治专项。制定绿色产品、绿色工厂、绿色园区、绿色企业标准体系，开展绿色评价。 　　到2020年，建成千家绿色示范工厂和百家绿色示范园区，部分重化工行业能源资源消耗出现拐点，重点行业主要污染物排放强度下降20%。到2025年，制造业绿色发展和主要产品单耗达到世界先进水平，绿色制造体系基本建立。 |

　　（六）大力推动重点领域突破发展。
　　瞄准新一代信息技术、高端装备、新材料、生物医药等战略重点，引导社会各类资源集聚，推动优势和战略产业快速发展。
　　1.新一代信息技术产业。
　　集成电路及专用装备。着力提升集成电路设计水平，不断丰富知识产权（IP）核和设计工具，突破关系国家信息与网络安全及电子整机产业发展的核心通用芯片，提升国产芯片的应用适配能力。掌握高密度封装及三维（3D）微组装技术，提升封装产业和测试的自主发展能力。形成关键制造装备供货能力。
　　信息通信设备。掌握新型计算、高速互联、先进存储、体系化安全保障等核心技术，全面突破第五代移动通信（5G）技术、核心路由交换技术、超高速大容量智能光传输技术、“未来网络”核心技术和体系架构，积极推动量子计算、神经网络等发展。研发高端服务器、大容量存储、新型路由交换、新型智能终端、新一代基站、网络安全等设备，推动核心信息通信设备体系化发展与规模化应用。
　　操作系统及工业软件。开发安全领域操作系统等工业基础软件。突破智能设计与仿真及其工具、制造物联与服务、工业大数据处理等高端工业软件核心技术，开发自主可控的高端工业平台软件和重点领域应用软件，建立完善工业软件集成标准与安全测评体系。推进自主工业软件体系化发展和产业化应用。
　　2.高档数控机床和机器人。
　　高档数控机床。开发一批精密、高速、高效、柔性数控机床与基础制造装备及集成制造系统。加快高档数控机床、增材制造等前沿技术和装备的研发。以提升可靠性、精度保持性为重点，开发高档数控系统、伺服电机、轴承、光栅等主要功能部件及关键应用软件，加快实现产业化。加强用户工艺验证能力建设。
　　机器人。围绕汽车、机械、电子、危险品制造、国防军工、化工、轻工等工业机器人、特种机器人，以及医疗健康、家庭服务、教育娱乐等服务机器人应用需求，积极研发新产品，促进机器人标准化、模块化发展，扩大市场应用。突破机器人本体、减速器、伺服电机、控制器、传感器与驱动器等关键零部件及系统集成设计制造等技术瓶颈。
　　3.航空航天装备。
　　航空装备。加快大型飞机研制，适时启动宽体客机研制，鼓励国际合作研制重型直升机；推进干支线飞机、直升机、无人机和通用飞机产业化。突破高推重比、先进涡桨（轴）发动机及大涵道比涡扇发动机技术，建立发动机自主发展工业体系。开发先进机载设备及系统，形成自主完整的航空产业链。
　　航天装备。发展新一代运载火箭、重型运载器，提升进入空间能力。加快推进国家民用空间基础设施建设，发展新型卫星等空间平台与有效载荷、空天地宽带互联网系统，形成长期持续稳定的卫星遥感、通信、导航等空间信息服务能力。推动载人航天、月球探测工程，适度发展深空探测。推进航天技术转化与空间技术应用。
　　4.海洋工程装备及高技术船舶。大力发展深海探测、资源开发利用、海上作业保障装备及其关键系统和专用设备。推动深海空间站、大型浮式结构物的开发和工程化。形成海洋工程装备综合试验、检测与鉴定能力，提高海洋开发利用水平。突破豪华邮轮设计建造技术，全面提升液化天然气船等高技术船舶国际竞争力，掌握重点配套设备集成化、智能化、模块化设计制造核心技术。
　　5.先进轨道交通装备。加快新材料、新技术和新工艺的应用，重点突破体系化安全保障、节能环保、数字化智能化网络化技术，研制先进可靠适用的产品和轻量化、模块化、谱系化产品。研发新一代绿色智能、高速重载轨道交通装备系统，围绕系统全寿命周期，向用户提供整体解决方案，建立世界领先的现代轨道交通产业体系。
　　6.节能与新能源汽车。继续支持电动汽车、燃料电池汽车发展，掌握汽车低碳化、信息化、智能化核心技术，提升动力电池、驱动电机、高效内燃机、先进变速器、轻量化材料、智能控制等核心技术的工程化和产业化能力，形成从关键零部件到整车的完整工业体系和创新体系，推动自主品牌节能与新能源汽车同国际先进水平接轨。
　　7.电力装备。推动大型高效超净排放煤电机组产业化和示范应用，进一步提高超大容量水电机组、核电机组、重型燃气轮机制造水平。推进新能源和可再生能源装备、先进储能装置、智能电网用输变电及用户端设备发展。突破大功率电力电子器件、高温超导材料等关键元器件和材料的制造及应用技术，形成产业化能力。
　　8.农机装备。重点发展粮、棉、油、糖等大宗粮食和战略性经济作物育、耕、种、管、收、运、贮等主要生产过程使用的先进农机装备，加快发展大型拖拉机及其复式作业机具、大型高效联合收割机等高端农业装备及关键核心零部件。提高农机装备信息收集、智能决策和精准作业能力，推进形成面向农业生产的信息化整体解决方案。
　　9.新材料。以特种金属功能材料、高性能结构材料、功能性高分子材料、特种无机非金属材料和先进复合材料为发展重点，加快研发先进熔炼、凝固成型、气相沉积、型材加工、高效合成等新材料制备关键技术和装备，加强基础研究和体系建设，突破产业化制备瓶颈。积极发展军民共用特种新材料，加快技术双向转移转化，促进新材料产业军民融合发展。高度关注颠覆性新材料对传统材料的影响，做好超导材料、纳米材料、石墨烯、生物基材料等战略前沿材料提前布局和研制。加快基础材料升级换代。
　　10.生物医药及高性能医疗器械。发展针对重大疾病的化学药、中药、生物技术药物新产品，重点包括新机制和新靶点化学药、抗体药物、抗体偶联药物、全新结构蛋白及多肽药物、新型疫苗、临床优势突出的创新中药及个性化治疗药物。提高医疗器械的创新能力和产业化水平，重点发展影像设备、医用机器人等高性能诊疗设备，全降解血管支架等高值医用耗材，可穿戴、远程诊疗等移动医疗产品。实现生物3D打印、诱导多能干细胞等新技术的突破和应用。

| 专栏5　高端装备创新工程 |
| --- |
| 组织实施大型飞机、航空发动机及燃气轮机、民用航天、智能绿色列车、节能与新能源汽车、海洋工程装备及高技术船舶、智能电网成套装备、高档数控机床、核电装备、高端诊疗设备等一批创新和产业化专项、重大工程。开发一批标志性、带动性强的重点产品和重大装备，提升自主设计水平和系统集成能力，突破共性关键技术与工程化、产业化瓶颈，组织开展应用试点和示范，提高创新发展能力和国际竞争力，抢占竞争制高点。 　　到2020年，上述领域实现自主研制及应用。到2025年，自主知识产权高端装备市场占有率大幅提升，核心技术对外依存度明显下降，基础配套能力显著增强，重要领域装备达到国际领先水平。 |

　　（七）深入推进制造业结构调整。
　　推动传统产业向中高端迈进，逐步化解过剩产能，促进大企业与中小企业协调发展，进一步优化制造业布局。
　　持续推进企业技术改造。明确支持战略性重大项目和高端装备实施技术改造的政策方向，稳定中央技术改造引导资金规模，通过贴息等方式，建立支持企业技术改造的长效机制。推动技术改造相关立法，强化激励约束机制，完善促进企业技术改造的政策体系。支持重点行业、高端产品、关键环节进行技术改造，引导企业采用先进适用技术，优化产品结构，全面提升设计、制造、工艺、管理水平，促进钢铁、石化、工程机械、轻工、纺织等产业向价值链高端发展。研究制定重点产业技术改造投资指南和重点项目导向计划，吸引社会资金参与，优化工业投资结构。围绕两化融合、节能降耗、质量提升、安全生产等传统领域改造，推广应用新技术、新工艺、新装备、新材料，提高企业生产技术水平和效益。
　　稳步化解产能过剩矛盾。加强和改善宏观调控，按照“消化一批、转移一批、整合一批、淘汰一批”的原则，分业分类施策，有效化解产能过剩矛盾。加强行业规范和准入管理，推动企业提升技术装备水平，优化存量产能。加强对产能严重过剩行业的动态监测分析，建立完善预警机制，引导企业主动退出过剩行业。切实发挥市场机制作用，综合运用法律、经济、技术及必要的行政手段，加快淘汰落后产能。
　　促进大中小企业协调发展。强化企业市场主体地位，支持企业间战略合作和跨行业、跨区域兼并重组，提高规模化、集约化经营水平，培育一批核心竞争力强的企业集团。激发中小企业创业创新活力，发展一批主营业务突出、竞争力强、成长性好、专注于细分市场的专业化“小巨人”企业。发挥中外中小企业合作园区示范作用，利用双边、多边中小企业合作机制，支持中小企业走出去和引进来。引导大企业与中小企业通过专业分工、服务外包、订单生产等多种方式，建立协同创新、合作共赢的协作关系。推动建设一批高水平的中小企业集群。
　　优化制造业发展布局。落实国家区域发展总体战略和主体功能区规划，综合考虑资源能源、环境容量、市场空间等因素，制定和实施重点行业布局规划，调整优化重大生产力布局。完善产业转移指导目录，建设国家产业转移信息服务平台，创建一批承接产业转移示范园区，引导产业合理有序转移，推动东中西部制造业协调发展。积极推动京津冀和长江经济带产业协同发展。按照新型工业化的要求，改造提升现有制造业集聚区，推动产业集聚向产业集群转型升级。建设一批特色和优势突出、产业链协同高效、核心竞争力强、公共服务体系健全的新型工业化示范基地。
　　（八）积极发展服务型制造和生产性服务业。
　　加快制造与服务的协同发展，推动商业模式创新和业态创新，促进生产型制造向服务型制造转变。大力发展与制造业紧密相关的生产性服务业，推动服务功能区和服务平台建设。
　　推动发展服务型制造。研究制定促进服务型制造发展的指导意见，实施服务型制造行动计划。开展试点示范，引导和支持制造业企业延伸服务链条，从主要提供产品制造向提供产品和服务转变。鼓励制造业企业增加服务环节投入，发展个性化定制服务、全生命周期管理、网络精准营销和在线支持服务等。支持有条件的企业由提供设备向提供系统集成总承包服务转变，由提供产品向提供整体解决方案转变。鼓励优势制造业企业“裂变”专业优势，通过业务流程再造，面向行业提供社会化、专业化服务。支持符合条件的制造业企业建立企业财务公司、金融租赁公司等金融机构，推广大型制造设备、生产线等融资租赁服务。
　　加快生产性服务业发展。大力发展面向制造业的信息技术服务，提高重点行业信息应用系统的方案设计、开发、综合集成能力。鼓励互联网等企业发展移动电子商务、在线定制、线上到线下等创新模式，积极发展对产品、市场的动态监控和预测预警等业务，实现与制造业企业的无缝对接，创新业务协作流程和价值创造模式。加快发展研发设计、技术转移、创业孵化、知识产权、科技咨询等科技服务业，发展壮大第三方物流、节能环保、检验检测认证、电子商务、服务外包、融资租赁、人力资源服务、售后服务、品牌建设等生产性服务业，提高对制造业转型升级的支撑能力。
　　强化服务功能区和公共服务平台建设。建设和提升生产性服务业功能区，重点发展研发设计、信息、物流、商务、金融等现代服务业，增强辐射能力。依托制造业集聚区，建设一批生产性服务业公共服务平台。鼓励东部地区企业加快制造业服务化转型，建立生产服务基地。支持中西部地区发展具有特色和竞争力的生产性服务业，加快产业转移承接地服务配套设施和能力建设，实现制造业和服务业协同发展。
　　（九）提高制造业国际化发展水平。
　　统筹利用两种资源、两个市场，实行更加积极的开放战略，将引进来与走出去更好结合，拓展新的开放领域和空间，提升国际合作的水平和层次，推动重点产业国际化布局，引导企业提高国际竞争力。
　　提高利用外资与国际合作水平。进一步放开一般制造业，优化开放结构，提高开放水平。引导外资投向新一代信息技术、高端装备、新材料、生物医药等高端制造领域，鼓励境外企业和科研机构在我国设立全球研发机构。支持符合条件的企业在境外发行股票、债券，鼓励与境外企业开展多种形式的技术合作。
　　提升跨国经营能力和国际竞争力。支持发展一批跨国公司，通过全球资源利用、业务流程再造、产业链整合、资本市场运作等方式，加快提升核心竞争力。支持企业在境外开展并购和股权投资、创业投资，建立研发中心、实验基地和全球营销及服务体系；依托互联网开展网络协同设计、精准营销、增值服务创新、媒体品牌推广等，建立全球产业链体系，提高国际化经营能力和服务水平。鼓励优势企业加快发展国际总承包、总集成。引导企业融入当地文化，增强社会责任意识，加强投资和经营风险管理，提高企业境外本土化能力。
　　深化产业国际合作，加快企业走出去。加强顶层设计，制定制造业走出去发展总体战略，建立完善统筹协调机制。积极参与和推动国际产业合作，贯彻落实丝绸之路经济带和21世纪海上丝绸之路等重大战略部署，加快推进与周边国家互联互通基础设施建设，深化产业合作。发挥沿边开放优势，在有条件的国家和地区建设一批境外制造业合作园区。坚持政府推动、企业主导，创新商业模式，鼓励高端装备、先进技术、优势产能向境外转移。加强政策引导，推动产业合作由加工制造环节为主向合作研发、联合设计、市场营销、品牌培育等高端环节延伸，提高国际合作水平。创新加工贸易模式，延长加工贸易国内增值链条，推动加工贸易转型升级。
　　四、战略支撑与保障
　　建设制造强国，必须发挥制度优势，动员各方面力量，进一步深化改革，完善政策措施，建立灵活高效的实施机制，营造良好环境；必须培育创新文化和中国特色制造文化，推动制造业由大变强。
　　（一）深化体制机制改革。
　　全面推进依法行政，加快转变政府职能，创新政府管理方式，加强制造业发展战略、规划、政策、标准等制定和实施，强化行业自律和公共服务能力建设，提高产业治理水平。简政放权，深化行政审批制度改革，规范审批事项，简化程序，明确时限；适时修订政府核准的投资项目目录，落实企业投资主体地位。完善政产学研用协同创新机制，改革技术创新管理体制机制和项目经费分配、成果评价和转化机制，促进科技成果资本化、产业化，激发制造业创新活力。加快生产要素价格市场化改革，完善主要由市场决定价格的机制，合理配置公共资源；推行节能量、碳排放权、排污权、水权交易制度改革，加快资源税从价计征，推动环境保护费改税。深化国有企业改革，完善公司治理结构，有序发展混合所有制经济，进一步破除各种形式的行业垄断，取消对非公有制经济的不合理限制。稳步推进国防科技工业改革，推动军民融合深度发展。健全产业安全审查机制和法规体系，加强关系国民经济命脉和国家安全的制造业重要领域投融资、并购重组、招标采购等方面的安全审查。
　　（二）营造公平竞争市场环境。
　　深化市场准入制度改革，实施负面清单管理，加强事中事后监管，全面清理和废止不利于全国统一市场建设的政策措施。实施科学规范的行业准入制度，制定和完善制造业节能节地节水、环保、技术、安全等准入标准，加强对国家强制性标准实施的监督检查，统一执法，以市场化手段引导企业进行结构调整和转型升级。切实加强监管，打击制售假冒伪劣行为，严厉惩处市场垄断和不正当竞争行为，为企业创造良好生产经营环境。加快发展技术市场，健全知识产权创造、运用、管理、保护机制。完善淘汰落后产能工作涉及的职工安置、债务清偿、企业转产等政策措施，健全市场退出机制。进一步减轻企业负担，实施涉企收费清单制度，建立全国涉企收费项目库，取缔各种不合理收费和摊派，加强监督检查和问责。推进制造业企业信用体系建设，建设中国制造信用数据库，建立健全企业信用动态评价、守信激励和失信惩戒机制。强化企业社会责任建设，推行企业产品标准、质量、安全自我声明和监督制度。
　　（三）完善金融扶持政策。
　　深化金融领域改革，拓宽制造业融资渠道，降低融资成本。积极发挥政策性金融、开发性金融和商业金融的优势，加大对新一代信息技术、高端装备、新材料等重点领域的支持力度。支持中国进出口银行在业务范围内加大对制造业走出去的服务力度，鼓励国家开发银行增加对制造业企业的贷款投放，引导金融机构创新符合制造业企业特点的产品和业务。健全多层次资本市场，推动区域性股权市场规范发展，支持符合条件的制造业企业在境内外上市融资、发行各类债务融资工具。引导风险投资、私募股权投资等支持制造业企业创新发展。鼓励符合条件的制造业贷款和租赁资产开展证券化试点。支持重点领域大型制造业企业集团开展产融结合试点，通过融资租赁方式促进制造业转型升级。探索开发适合制造业发展的保险产品和服务，鼓励发展贷款保证保险和信用保险业务。在风险可控和商业可持续的前提下，通过内保外贷、外汇及人民币贷款、债权融资、股权融资等方式，加大对制造业企业在境外开展资源勘探开发、设立研发中心和高技术企业以及收购兼并等的支持力度。
　　（四）加大财税政策支持力度。
　　充分利用现有渠道，加强财政资金对制造业的支持，重点投向智能制造、“四基”发展、高端装备等制造业转型升级的关键领域，为制造业发展创造良好政策环境。运用政府和社会资本合作（PPP）模式，引导社会资本参与制造业重大项目建设、企业技术改造和关键基础设施建设。创新财政资金支持方式，逐步从“补建设”向“补运营”转变，提高财政资金使用效益。深化科技计划（专项、基金等）管理改革，支持制造业重点领域科技研发和示范应用，促进制造业技术创新、转型升级和结构布局调整。完善和落实支持创新的政府采购政策，推动制造业创新产品的研发和规模化应用。落实和完善使用首台（套）重大技术装备等鼓励政策，健全研制、使用单位在产品创新、增值服务和示范应用等环节的激励约束机制。实施有利于制造业转型升级的税收政策，推进增值税改革，完善企业研发费用计核方法，切实减轻制造业企业税收负担。
　　（五）健全多层次人才培养体系。
　　加强制造业人才发展统筹规划和分类指导，组织实施制造业人才培养计划，加大专业技术人才、经营管理人才和技能人才的培养力度，完善从研发、转化、生产到管理的人才培养体系。以提高现代经营管理水平和企业竞争力为核心，实施企业经营管理人才素质提升工程和国家中小企业银河培训工程，培养造就一批优秀企业家和高水平经营管理人才。以高层次、急需紧缺专业技术人才和创新型人才为重点，实施专业技术人才知识更新工程和先进制造卓越工程师培养计划，在高等学校建设一批工程创新训练中心，打造高素质专业技术人才队伍。强化职业教育和技能培训，引导一批普通本科高等学校向应用技术类高等学校转型，建立一批实训基地，开展现代学徒制试点示范，形成一支门类齐全、技艺精湛的技术技能人才队伍。鼓励企业与学校合作，培养制造业急需的科研人员、技术技能人才与复合型人才，深化相关领域工程博士、硕士专业学位研究生招生和培养模式改革，积极推进产学研结合。加强产业人才需求预测，完善各类人才信息库，构建产业人才水平评价制度和信息发布平台。建立人才激励机制，加大对优秀人才的表彰和奖励力度。建立完善制造业人才服务机构，健全人才流动和使用的体制机制。采取多种形式选拔各类优秀人才重点是专业技术人才到国外学习培训，探索建立国际培训基地。加大制造业引智力度，引进领军人才和紧缺人才。
　　（六）完善中小微企业政策。
　　落实和完善支持小微企业发展的财税优惠政策，优化中小企业发展专项资金使用重点和方式。发挥财政资金杠杆撬动作用，吸引社会资本，加快设立国家中小企业发展基金。支持符合条件的民营资本依法设立中小型银行等金融机构，鼓励商业银行加大小微企业金融服务专营机构建设力度，建立完善小微企业融资担保体系，创新产品和服务。加快构建中小微企业征信体系，积极发展面向小微企业的融资租赁、知识产权质押贷款、信用保险保单质押贷款等。建设完善中小企业创业基地，引导各类创业投资基金投资小微企业。鼓励大学、科研院所、工程中心等对中小企业开放共享各种实（试）验设施。加强中小微企业综合服务体系建设，完善中小微企业公共服务平台网络，建立信息互联互通机制，为中小微企业提供创业、创新、融资、咨询、培训、人才等专业化服务。
　　（七）进一步扩大制造业对外开放。
　　深化外商投资管理体制改革，建立外商投资准入前国民待遇加负面清单管理机制，落实备案为主、核准为辅的管理模式，营造稳定、透明、可预期的营商环境。全面深化外汇管理、海关监管、检验检疫管理改革，提高贸易投资便利化水平。进一步放宽市场准入，修订钢铁、化工、船舶等产业政策，支持制造业企业通过委托开发、专利授权、众包众创等方式引进先进技术和高端人才，推动利用外资由重点引进技术、资金、设备向合资合作开发、对外并购及引进领军人才转变。加强对外投资立法，强化制造业企业走出去法律保障，规范企业境外经营行为，维护企业合法权益。探索利用产业基金、国有资本收益等渠道支持高铁、电力装备、汽车、工程施工等装备和优势产能走出去，实施海外投资并购。加快制造业走出去支撑服务机构建设和水平提升，建立制造业对外投资公共服务平台和出口产品技术性贸易服务平台，完善应对贸易摩擦和境外投资重大事项预警协调机制。
　　（八）健全组织实施机制。
　　成立国家制造强国建设领导小组，由国务院领导同志担任组长，成员由国务院相关部门和单位负责同志担任。领导小组主要职责是：统筹协调制造强国建设全局性工作，审议重大规划、重大政策、重大工程专项、重大问题和重要工作安排，加强战略谋划，指导部门、地方开展工作。领导小组办公室设在工业和信息化部，承担领导小组日常工作。设立制造强国建设战略咨询委员会，研究制造业发展的前瞻性、战略性重大问题，对制造业重大决策提供咨询评估。支持包括社会智库、企业智库在内的多层次、多领域、多形态的中国特色新型智库建设，为制造强国建设提供强大智力支持。建立《中国制造2025》任务落实情况督促检查和第三方评价机制，完善统计监测、绩效评估、动态调整和监督考核机制。建立《中国制造2025》中期评估机制，适时对目标任务进行必要调整。
　　各地区、各部门要充分认识建设制造强国的重大意义，加强组织领导，健全工作机制，强化部门协同和上下联动。各地区要结合当地实际，研究制定具体实施方案，细化政策措施，确保各项任务落实到位。工业和信息化部要会同相关部门加强跟踪分析和督促指导，重大事项及时向国务院报告。

国务院
　　　　　　　　　　　　　　　　　　　　　　　　　　　2015年5月8日

**P7**

**Guidance on Promoting the Application of Building Information Modeling**

**关于推进建筑信息模型应用的指导意见**

建质函[2015]159号

为贯彻《关于印发2011-2015年建筑业信息化发展纲要的通知》（建质[2011]67号）和《住房城乡建设部关于推进建筑业发展和改革的若干意见》（建市[2014]92号）的有关工作部署，现就推进建筑信息模型（Building Information Modeling，以下简称BIM）的应用提出以下意见。

一、BIM在建筑领域应用的重要意义

BIM是在计算机辅助设计（CAD）等技术基础上发展起来的多维模型信息集成技术，是对建筑工程物理特征和功能特性信息的数字化承载和可视化表达。

BIM能够应用于工程项目规划、勘察、设计、施工、运营维护等各阶段,实现建筑全生命期各参与方在同一多维建筑信息模型基础上的数据共享，为产业链贯通、工业化建造和繁荣建筑创作提供技术保障；支持对工程环境、能耗、经济、质量、安全等方面的分析、检查和模拟，为项目全过程的方案优化和科学决策提供依据；支持各专业协同工作、项目的虚拟建造和精细化管理，为建筑业的提质增效、节能环保创造条件。

信息化是建筑产业现代化的主要特征之一,BIM应用作为建筑业信息化的重要组成部分，必将极大地促进建筑领域生产方式的变革。

目前，BIM在建筑领域的推广应用还存在着政策法规和标准不完善、发展不平衡、本土应用软件不成熟、技术人才不足等问题，有必要采取切实可行的措施，推进BIM在建筑领域的应用。

二、指导思想与基本原则

（一）指导思想。

以工程建设法律法规、技术标准为依据，坚持科技进步和管理创新相结合，在建筑领域普及和深化BIM应用，提高工程项目全生命期各参与方的工作质量和效率，保障工程建设优质、安全、环保、节能。

（二）基本原则。

1.企业主导，需求牵引。发挥企业在BIM应用中的主体作用，聚焦于工程项目全生命期内的经济、社会和环境效益，通过BIM应用，提高工程项目管理水平，保证工程质量和综合效益。

2.行业服务，创新驱动。发挥行业协会、学会组织优势，自主创新与引进集成创新并重，研发具有自主知识产权的BIM应用软件，建立BIM数据库及信息平台，培养研发和应用人才队伍。

3.政策引导，示范推动。发挥政府在产业政策上的引领作用，研究出台推动BIM应用的政策措施和技术标准。坚持试点示范和普及应用相结合，培育龙头企业，总结成功经验，带动全行业的BIM应用。

三、发展目标

到2020年末，建筑行业甲级勘察、设计单位以及特级、一级房屋建筑工程施工企业应掌握并实现BIM与企业管理系统和其他信息技术的一体化集成应用。

到2020年末，以下新立项项目勘察设计、施工、运营维护中，集成应用BIM的项目比率达到90%：以国有资金投资为主的大中型建筑；申报绿色建筑的公共建筑和绿色生态示范小区。

四、工作重点

各级住房城乡建设主管部门要结合实际，制定BIM应用配套激励政策和措施，扶持和推进相关单位开展BIM的研发和集成应用，研究适合BIM应用的质量监管和档案管理模式。

有关单位和企业要根据实际需求制定BIM应用发展规划、分阶段目标和实施方案，合理配置BIM应用所需的软硬件。改进传统项目管理方法，建立适合BIM应用的工程管理模式。构建企业级各专业族库，逐步建立覆盖BIM创建、修改、交换、应用和交付全过程的企业BIM应用标准流程。通过科研合作、技术培训、人才引进等方式，推动相关人员掌握BIM应用技能，全面提升BIM应用能力。

（一）建设单位。

全面推行工程项目全生命期、各参与方的BIM应用，要求各参建方提供的数据信息具有便于集成、管理、更新、维护以及可快速检索、调用、传输、分析和可视化等特点。实现工程项目投资策划、勘察设计、施工、运营维护各阶段基于BIM标准的信息传递和信息共享。满足工程建设不同阶段对质量管控和工程进度、投资控制的需求。

建立科学的决策机制。在工程项目可行性研究和方案设计阶段,通过建立基于BIM的可视化信息模型，提高各参与方的决策参与度。

建立BIM应用框架。明确工程实施阶段各方的任务、交付标准和费用分配比例。

建立BIM数据管理平台。建立面向多参与方、多阶段的BIM数据管理平台，为各阶段的BIM应用及各参与方的数据交换提供一体化信息平台支持。

建筑方案优化。在工程项目勘察、设计阶段，要求各方利用BIM开展相关专业的性能分析和对比，对建筑方案进行优化。

施工监控和管理。在工程项目施工阶段，促进相关方利用BIM进行虚拟建造，通过施工过程模拟对施工组织方案进行优化，确定科学合理的施工工期，对物料、设备资源进行动态管控，切实提升工程质量和综合效益。

投资控制。在招标、工程变更、竣工结算等各个阶段，利用BIM进行工程量及造价的精确计算，并作为投资控制的依据。

运营维护和管理。在运营维护阶段，充分利用BIM和虚拟仿真技术，分析不同运营维护方案的投入产出效果，模拟维护工作对运营带来的影响，提出先进合理的运营维护方案。

（二）勘察单位。

研究建立基于BIM的工程勘察流程与工作模式，根据工程项目的实际需求和应用条件确定不同阶段的工作内容。开展BIM示范应用。

工程勘察模型建立。研究构建支持多种数据表达方式与信息传输的工程勘察数据库，研发和采用BIM应用软件与建模技术，建立可视化的工程勘察模型，实现建筑与其地下工程地质信息的三维融合。

模拟与分析。实现工程勘察基于BIM的数值模拟和空间分析，辅助用户进行科学决策和规避风险。

信息共享。开发岩土工程各种相关结构构件族库，建立统一数据格式标准和数据交换标准，实现信息的有效传递。

（三）设计单位。

研究建立基于BIM的协同设计工作模式，根据工程项目的实际需求和应用条件确定不同阶段的工作内容。开展BIM示范应用，积累和构建各专业族库，制定相关企业标准。

1. 投资策划与规划。在项目前期策划和规划设计阶段，基于BIM和地理信息系统（GIS）技术，对项目规划方案和投资策略进行模拟分析。

2. 设计模型建立。采用BIM应用软件和建模技术，构建包括建筑、结构、给排水、暖通空调、电气设备、消防等多专业信息的BIM模型。根据不同设计阶段任务要求，形成满足各参与方使用要求的数据信息。

3.分析与优化。进行包括节能、日照、风环境、光环境、声环境、热环境、交通、抗震等在内的建筑性能分析。根据分析结果，结合全生命期成本，进行优化设计。

4.设计成果审核。利用基于BIM的协同工作平台等手段，开展多专业间的数据共享和协同工作，实现各专业之间数据信息的无损传递和共享，进行各专业之间的碰撞检测和管线综合碰撞检测，最大限度减少错、漏、碰、缺等设计质量通病，提高设计质量和效率。

（四）施工企业。

改进传统项目管理方法，建立基于BIM应用的施工管理模式和协同工作机制。明确施工阶段各参与方的协同工作流程和成果提交内容，明确人员职责，制定管理制度。开展BIM应用示范，根据示范经验，逐步实现施工阶段的BIM集成应用。

1.施工模型建立。施工企业应利用基于BIM的数据库信息，导入和处理已有的BIM设计模型，形成BIM施工模型。

2.细化设计。利用BIM设计模型根据施工安装需要进一步细化、完善，指导建筑部品构件的生产以及现场施工安装。

3.专业协调。进行建筑、结构、设备等各专业以及管线在施工阶段综合的碰撞检测、分析和模拟，消除冲突，减少返工。

4.成本管理与控制。应用BIM施工模型，精确高效计算工程量，进而辅助工程预算的编制。在施工过程中，对工程动态成本进行实时、精确的分析和计算，提高对项目成本和工程造价的管理能力。

5.施工过程管理。应用 BIM施工模型，对施工进度、人力、材料、设备、质量、安全、场地布置等信息进行动态管理，实现施工过程的可视化模拟和施工方案的不断优化。

6.质量安全监控。综合应用数字监控、移动通讯和物联网技术，建立BIM与现场监测数据的融合机制，实现施工现场集成通讯与动态监管、施工时变结构及支撑体系安全分析、大型施工机械操作精度检测、复杂结构施工定位与精度分析等，进一步提高施工精度、效率和安全保障水平。

7.地下工程风险管控。利用基于BIM的岩土工程施工模型，模拟地下工程施工过程以及对周边环境影响，对地下工程施工过程可能存在的危险源进行分析评估，制定风险防控措施。

8.交付竣工模型。BIM竣工模型应包括建筑、结构和机电设备等各专业内容，在三维几何信息的基础上，还包含材料、荷载、技术参数和指标等设计信息，质量、安全、耗材、成本等施工信息，以及构件与设备信息等。

（五）工程总承包企业。

根据工程总承包项目的过程需求和应用条件确定BIM应用内容，分阶段（工程启动、工程策划、工程实施、工程控制、工程收尾）开展BIM应用。在综合设计、咨询服务、集成管理等建筑业价值链中技术含量高、知识密集型的环节大力推进BIM应用。优化项目实施方案，合理协调各阶段工作，缩短工期、提高质量、节省投资。实现与设计、施工、设备供应、专业分包、劳务分包等单位的无缝对接，优化供应链，提升自身价值。

1.设计控制。按照方案设计、初步设计、施工图设计等阶段的总包管理需求，逐步建立适宜的多方共享的BIM模型。使设计优化、设计深化、设计变更等业务基于统一的BIM模型，并实施动态控制。

2.成本控制。基于BIM施工模型，快速形成项目成本计划，高效、准确地进行成本预测、控制、核算、分析等，有效提高成本管控能力。

3.进度控制。基于BIM施工模型，对多参与方、多专业的进度计划进行集成化管理，全面、动态地掌握工程进度、资源需求以及供应商生产及配送状况，解决施工和资源配置的冲突和矛盾，确保工期目标实现。

4.质量安全管理。基于BIM施工模型，对复杂施工工艺进行数字化模拟，实现三维可视化技术交底；对复杂结构实现三维放样、定位和监测；实现工程危险源的自动识别分析和防护方案的模拟；实现远程质量验收。

5.协调管理。基于BIM，集成各分包单位的专业模型，管理各分包单位的深化设计和专业协调工作，提升工程信息交付质量和建造效率；优化施工现场环境和资源配置,减少施工现场各参与方、各专业之间的互相干扰。

6.交付工程总承包BIM竣工模型。工程总承包BIM竣工模型应包括工程启动、工程策划、工程实施、工程控制、工程收尾等工程总承包全过程中，用于竣工交付、资料归档、运营维护的相关信息。

（六）运营维护单位。

改进传统的运营维护管理方法，建立基于BIM应用的运营维护管理模式。建立基于BIM的运营维护管理协同工作机制、流程和制度。建立交付标准和制度，保证BIM竣工模型完整、准确地提交到运营维护阶段。

1.运营维护模型建立。可利用基于BIM的数据集成方法，导入和处理已有的BIM竣工交付模型，再通过运营维护信息录入和数据集成，建立项目BIM运营维护模型。也可以利用其他竣工资料直接建立BIM运营维护模型。

2.运营维护管理。应用BIM运营维护模型，集成BIM、物联网和GIS技术，构建综合BIM运营维护管理平台，支持大型公共建筑和住宅小区的基础设施和市政管网的信息化管理，实现建筑物业、设备、设施及其巡检维修的精细化和可视化管理，并为工程健康监测提供信息支持。

3.设备设施运行监控。综合应用智能建筑技术，将建筑设备及管线的BIM运营维护模型与楼宇设备自动控制系统相结合，通过运营维护管理平台，实现设备运行和排放的实时监测、分析和控制，支持设备设施运行的动态信息查询和异常情况快速定位。

4.应急管理。综合应用BIM运营维护模型和各类灾害分析、虚拟现实等技术，实现各种可预见灾害模拟和应急处置。

五、保障措施

（一）大力宣传BIM理念、意义、价值，通过政府投资工程招投标、工程创优评优、绿色建筑和建筑产业现代化评价等工作激励建筑领域的BIM应用。

（二）梳理、修订、补充有关法律法规、合同范本的条款规定，研究并建立基于BIM应用的工程建设项目政府监管流程；研究基于BIM的产业（企业）价值分配机制，形成市场化的工程各方应用BIM费用标准。

（三）制订有关工程建设标准和应用指南，建立BIM应用标准体系；研究建立基于BIM的公共建筑构件资源数据中心及服务平台。

（四）研究解决提升BIM应用软件数据集成水平等一系列重大技术问题；鼓励BIM应用软件产业化、系统化、标准化，支持软件开发企业自主研发适合国情的BIM应用软件；推动开发基于BIM的工程项目管理与企业管理系统。

（五）加强工程质量安全监管、施工图审查、工程监理、造价咨询以及工程档案管理等工作中的BIM应用研究，逐步将BIM融入到相关政府部门和企业的日常管理工作中。

（六）培育产、学、研、用相结合的BIM应用产业化示范基地和产业联盟；在条件具备的地区和行业，建设BIM应用示范（试点）工程。

（七）加强对企业管理人员和技术人员关于BIM应用的相关培训，在注册执业资格人员的继续教育必修课中增加有关BIM的内容；鼓励有条件的地区，建立企业和人员的BIM应用水平考核评价机制。

　中华人民共和国住房和城乡建设部　　　　　　　　　　　　　　　　　　　　　　　　　2015年6月16日

**P8**

**Outline for the Development of Information Technology in Construction Industry (2016-2020)**

**2016-2020年建筑业信息化发展纲要**

建质函[2016]183号

建筑业信息化是建筑业发展战略的重要组成部分，也是建筑业转变发展方式、提质增效、节能减排的必然要求，对建筑业绿色发展、提高人民生活品质具有重要意义。

一、指导思想

贯彻党的十八大以来、国务院推进信息化发展相关精神，落实创新、协调、绿色、开放、共享的发展理念及国家大数据战略、“互联网+”行动等相关要求，实施《国家信息化发展战略纲要》，增强建筑业信息化发展能力，优化建筑业信息化发展环境，加快推动信息技术与建筑业发展深度融合，充分发挥信息化的引领和支撑作用，塑造建筑业新业态。

二、发展目标

“十三五”时期，全面提高建筑业信息化水平，着力增强BIM、大数据、智能化、移动通讯、云计算、物联网等信息技术集成应用能力，建筑业数字化、网络化、智能化取得突破性进展，初步建成一体化行业监管和服务平台，数据资源利用水平和信息服务能力明显提升，形成一批具有较强信息技术创新能力和信息化应用达到国际先进水平的建筑企业及具有关键自主知识产权的建筑业信息技术企业。

三、主要任务

（一）企业信息化。

建筑企业应积极探索“互联网+”形势下管理、生产的新模式，深入研究BIM、物联网等技术的创新应用，创新商业模式，增强核心竞争力，实现跨越式发展。

1.勘察设计类企业。

（1）推进信息技术与企业管理深度融合。

进一步完善并集成企业运营管理信息系统、生产经营管理信息系统，实现企业管理信息系统的升级换代。深度融合BIM、大数据、智能化、移动通讯、云计算等信息技术，实现BIM与企业管理信息系统的一体化应用，促进企业设计水平和管理水平的提高。

（2）加快BIM普及应用，实现勘察设计技术升级。

在工程项目勘察中，推进基于BIM进行数值模拟、空间分析和可视化表达，研究构建支持异构数据和多种采集方式的工程勘察信息数据库，实现工程勘察信息的有效传递和共享。在工程项目策划、规划及监测中，集成应用BIM、GIS、物联网等技术，对相关方案及结果进行模拟分析及可视化展示。在工程项目设计中，普及应用BIM进行设计方案的性能和功能模拟分析、优化、绘图、审查，以及成果交付和可视化沟通，提高设计质量。

推广基于BIM的协同设计，开展多专业间的数据共享和协同，优化设计流程，提高设计质量和效率。研究开发基于BIM的集成设计系统及协同工作系统，实现建筑、结构、水暖电等专业的信息集成与共享。

（3）强化企业知识管理，支撑智慧企业建设。

研究改进勘察设计信息资源的获取和表达方式，探索知识管理和发展模式，建立勘察设计知识管理信息系统。不断开发勘察设计信息资源，完善知识库，实现知识的共享，充分挖掘和利用知识的价值，支撑智慧企业建设。

2.施工类企业。

（1）加强信息化基础设施建设。

建立满足企业多层级管理需求的数据中心，可采用私有云、公有云或混合云等方式。在施工现场建设互联网基础设施，广泛使用无线网络及移动终端，实现项目现场与企业管理的互联互通强化信息安全，完善信息化运维管理体系，保障设施及系统稳定可靠运行。

（2）推进管理信息系统升级换代。

普及项目管理信息系统，开展施工阶段的BIM基础应用。有条件的企业应研究BIM应用条件下的施工管理模式和协同工作机制，建立基于BIM的项目管理信息系统。

推进企业管理信息系统建设。完善并集成项目管理、人力资源管理、财务资金管理、劳务管理、物资材料管理等信息系统，实现企业管理与主营业务的信息化。有条件的企业应推进企业管理信息系统中项目业务管理和财务管理的深度集成，实现业务财务管理一体化。推动基于移动通讯、互联网的施工阶段多参与方协同工作系统的应用，实现企业与项目其他参与方的信息沟通和数据共享。注重推进企业知识管理信息系统、商业智能和决策支持系统的应用，有条件的企业应探索大数据技术的集成应用，支撑智慧企业建设。

（3）拓展管理信息系统新功能。

研究建立风险管理信息系统，提高企业风险管控能力。建立并完善电子商务系统，或利用第三方电子商务系统，开展物资设备采购和劳务分包，降低成本。开展BIM与物联网、云计算、3S等技术在施工过程中的集成应用研究，建立施工现场管理信息系统，创新施工管理模式和手段。

3.工程总承包类企业。

（1）优化工程总承包项目信息化管理，提升集成应用水平。

进一步优化工程总承包项目管理组织架构、工作流程及信息流，持续完善项目资源分解结构和编码体系。深化应用估算、投标报价、费用控制及计划进度控制等信息系统，逐步建立适应国际工程的估算、报价、费用及进度管控体系。继续完善商务管理、资金管理、财务管理、风险管理及电子商务等信息系统，提升成本管理和风险管控水平。利用新技术提升并深化应用项目管理信息系统，实现设计管理、采购管理、施工管理、企业管理等信息系统的集成及应用。

探索PPP等工程总承包项目的信息化管理模式，研究建立相应的管理信息系统。

（2）推进“互联网+”协同工作模式，实现全过程信息化。

研究“互联网+”环境下的工程总承包项目多参与方协同工作模式，建立并应用基于互联网的协同工作系统，实现工程项目多参与方之间的高效协同与信息共享。研究制定工程总承包项目基于BIM的多参与方成果交付标准，实现从设计、施工到运行维护阶段的数字化交付和全生命期信息共享。

（二）行业监管与服务信息化。

积极探索“互联网+”形势下建筑行业格局和资源整合的新模式，促进建筑业行业新业态，支持“互联网+”形势下企业创新发展。

1.建筑市场监管。

（1）深化行业诚信管理信息化。

研究建立基于互联网的建筑企业、从业人员基本信息及诚信信息的共享模式与方法。完善行业诚信管理信息系统，实现企业、从业人员诚信信息和项目信息的集成化信息服务。

（2）加强电子招投标的应用。

应用大数据技术识别围标、串标等不规范行为，保障招投标过程的公正、公平。

（3）推进信息技术在劳务实名制管理中应用。

应用物联网、大数据和基于位置的服务（LBS）等技术建立全国建筑工人信息管理平台，并与诚信管理信息系统进行对接，实现深层次的劳务人员信息共享。推进人脸识别、指纹识别、虹膜识别等技术在工程现场劳务人员管理中的应用，与工程现场劳务人员安全、职业健康、培训等信息联动。

2.工程建设监管。

（1）建立完善数字化成果交付体系。

建立设计成果数字化交付、审查及存档系统，推进基于二维图的、探索基于BIM的数字化成果交付、审查和存档管理。开展白图代蓝图和数字化审图试点、示范工作。完善工程竣工备案管理信息系统，探索基于BIM的工程竣工备案模式。

（2）加强信息技术在工程质量安全管理中的应用。

构建基于BIM、大数据、智能化、移动通讯、云计算等技术的工程质量、安全监管模式与机制。建立完善工程项目质量监管信息系统，对工程实体质量和工程建设、勘察、设计、施工、监理和质量检测单位的质量行为监管信息进行采集，实现工程竣工验收备案、建筑工程五方责任主体项目负责人等信息共享，保障数据可追溯，提高工程质量监管水平。建立完善建筑施工安全监管信息系统，对工程现场人员、机械设备、临时设施等安全信息进行采集和汇总分析，实现施工企业、人员、项目等安全监管信息互联共享，提高施工安全监管水平。

（3）推进信息技术在工程现场环境、能耗监测和建筑垃圾管理中的应用。

研究探索基于物联网、大数据等技术的环境、能耗监测模式，探索建立环境、能耗分析的动态监控系统，实现对工程现场空气、粉尘、用水、用电等的实时监测。建立建筑垃圾综合管理信息系统，实现项目建筑垃圾的申报、识别、计量、跟踪、结算等数据的实时监控，提升绿色建造水平。

3.重点工程信息化。

大力推进BIM、GIS等技术在综合管廊建设中的应用，建立综合管廊集成管理信息系统，逐步形成智能化城市综合管廊运营服务能力。在海绵城市建设中积极应用BIM、虚拟现实等技术开展规划、设计，探索基于云计算、大数据等的运营管理，并示范应用。加快BIM技术在城市轨道交通工程设计、施工中的应用，推动各参建方共享多维建筑信息模型进行工程管理。在“一带一路”重点工程中应用BIM进行建设，探索云计算、大数据、GIS等技术的应用。

4.建筑产业现代化。

加强信息技术在装配式建筑中的应用，推进基于BIM的建筑工程设计、生产、运输、装配及全生命期管理，促进工业化建造。建立基于BIM、物联网等技术的云服务平台，实现产业链各参与方之间在各阶段、各环节的协同工作。

5.行业信息共享与服务。

研究建立工程建设信息公开系统，为行业和公众提供地质勘察、环境及能耗监测等信息服务，提高行业公共信息利用水平。建立完善工程项目数字化档案管理信息系统，转变档案管理服务模式，推进可公开的档案信息共享。

（三）专项信息技术应用。

1.大数据技术。

研究建立建筑业大数据应用框架，统筹政务数据资源和社会数据资源，建设大数据应用系统，推进公共数据资源向社会开放。汇聚整合和分析建筑企业、项目、从业人员和信用信息等相关大数据，探索大数据在建筑业创新应用，推进数据资产管理，充分利用大数据价值。建立安全保障体系，规范大数据采集、传输、存储、应用等各环节安全保障措施。

2.云计算技术。

积极利用云计算技术改造提升现有电子政务信息系统、企业信息系统及软硬件资源，降低信息化成本。挖掘云计算技术在工程建设管理及设施运行监控等方面应用潜力。

3.物联网技术。

结合建筑业发展需求，加强低成本、低功耗、智能化传感器及相关设备的研发，实现物联网核心芯片、仪器仪表、配套软件等在建筑业的集成应用。开展传感器、高速移动通讯、无线射频、近场通讯及二维码识别等物联网技术与工程项目管理信息系统的集成应用研究，开展示范应用。

4.3D打印技术。

积极开展建筑业3D打印设备及材料的研究。结合BIM技术应用，探索3D打印技术运用于建筑部品、构件生产，开展示范应用。

5.智能化技术。

开展智能机器人、智能穿戴设备、手持智能终端设备、智能监测设备、3D扫描等设备在施工过程中的应用研究，提升施工质量和效率，降低安全风险。探索智能化技术与大数据、移动通讯、云计算、物联网等信息技术在建筑业中的集成应用，促进智慧建造和智慧企业发展。

（四）信息化标准。

强化建筑行业信息化标准顶层设计，继续完善建筑业行业与企业信息化标准体系，结合BIM等新技术应用，重点完善建筑工程勘察设计、施工、运维全生命期的信息化标准体系，为信息资源共享和深度挖掘奠定基础。

加快相关信息化标准的编制，重点编制和完善建筑行业及企业信息化相关的编码、数据交换、文档及图档交付等基础数据和通用标准。继续推进BIM技术应用标准的编制工作，结合物联网、云计算、大数据等新技术在建筑行业的应用，研究制定相关标准。

四、保障措施

（一）加强组织领导，完善配套政策，加快推进建筑业信息化。

各级城乡建设行政主管部门要制定本地区“十三五”建筑业信息化发展目标和措施，加快完善相关配套政策措施，形成信息化推进工作机制，落实信息化建设专项经费保障。探索建立信息化条件下的电子招投标、数字化交付和电子签章等相关制度。

建立信息化专家委员会及专家库，充分发挥专家作用，建立产学研用相结合的建筑业信息化创新体系，加强信息技术与建筑业结合的专项应用研究、建筑业信息化软科学研究。开展建筑业信息化示范工程，根据国家“双创”工程，开展基于“互联网+”的建筑业信息化创新创业示范。

（二）大力增强建筑企业信息化能力。

企业应制定企业信息化发展目标及配套管理制度，加强信息化在企业标准化管理中的带动作用。鼓励企业建立首席信息官（CIO）制度，按营业收入一定比例投入信息化建设，开辟投融资渠道，保证建设和运行的资金投入。注重引进BIM等信息技术专业人才，培育精通信息技术和业务的复合型人才，强化各类人员信息技术应用培训，提高全员信息化应用能力。大型企业要积极探索开发自有平台，瞄准国际前沿，加强信息化关键技术应用攻关，推动行业信息化发展。

（三）强化信息化安全建设。

各级城乡建设行政主管部门和广大企业要提高信息安全意识，建立健全信息安全保障体系，重视数据资产管理，积极开展信息系统安全等级保护工作，提高信息安全水平。

中华人民共和国住房和城乡建设部
2016年8月23日

**P9**

**Guidance on Vigorously Developing Prefabricated Buildings**

**国务院办公厅关于大力发展装配式建筑的指导意见**

国办发〔2016〕71号

各省、自治区、直辖市人民政府，国务院各部委、各直属机构：

装配式建筑是用预制部品部件在工地装配而成的建筑。发展装配式建筑是建造方式的重大变革，是推进供给侧结构性改革和新型城镇化发展的重要举措，有利于节约资源能源、减少施工污染、提升劳动生产效率和质量安全水平，有利于促进建筑业与信息化工业化深度融合、培育新产业新动能、推动化解过剩产能。近年来，我国积极探索发展装配式建筑，但建造方式大多仍以现场浇筑为主，装配式建筑比例和规模化程度较低，与发展绿色建筑的有关要求以及先进建造方式相比还有很大差距。为贯彻落实《中共中央　国务院关于进一步加强城市规划建设管理工作的若干意见》和《政府工作报告》部署，大力发展装配式建筑，经国务院同意，现提出以下意见。

一、总体要求

（一）指导思想。全面贯彻党的十八大和十八届三中、四中、五中全会以及中央城镇化工作会议、中央城市工作会议精神，认真落实党中央、国务院决策部署，按照“五位一体”总体布局和“四个全面”战略布局，牢固树立和贯彻落实创新、协调、绿色、开放、共享的发展理念，按照适用、经济、安全、绿色、美观的要求，推动建造方式创新，大力发展装配式混凝土建筑和钢结构建筑，在具备条件的地方倡导发展现代木结构建筑，不断提高装配式建筑在新建建筑中的比例。坚持标准化设计、工厂化生产、装配化施工、一体化装修、信息化管理、智能化应用，提高技术水平和工程质量，促进建筑产业转型升级。

（二）基本原则。

坚持市场主导、政府推动。适应市场需求，充分发挥市场在资源配置中的决定性作用，更好发挥政府规划引导和政策支持作用，形成有利的体制机制和市场环境，促进市场主体积极参与、协同配合，有序发展装配式建筑。

坚持分区推进、逐步推广。根据不同地区的经济社会发展状况和产业技术条件，划分重点推进地区、积极推进地区和鼓励推进地区，因地制宜、循序渐进，以点带面、试点先行，及时总结经验，形成局部带动整体的工作格局。

坚持顶层设计、协调发展。把协同推进标准、设计、生产、施工、使用维护等作为发展装配式建筑的有效抓手，推动各个环节有机结合，以建造方式变革促进工程建设全过程提质增效，带动建筑业整体水平的提升。

（三）工作目标。以京津冀、长三角、珠三角三大城市群为重点推进地区，常住人口超过300万的其他城市为积极推进地区，其余城市为鼓励推进地区，因地制宜发展装配式混凝土结构、钢结构和现代木结构等装配式建筑。力争用10年左右的时间，使装配式建筑占新建建筑面积的比例达到30%。同时，逐步完善法律法规、技术标准和监管体系，推动形成一批设计、施工、部品部件规模化生产企业，具有现代装配建造水平的工程总承包企业以及与之相适应的专业化技能队伍。

二、重点任务

（四）健全标准规范体系。加快编制装配式建筑国家标准、行业标准和地方标准，支持企业编制标准、加强技术创新，鼓励社会组织编制团体标准，促进关键技术和成套技术研究成果转化为标准规范。强化建筑材料标准、部品部件标准、工程标准之间的衔接。制修订装配式建筑工程定额等计价依据。完善装配式建筑防火抗震防灾标准。研究建立装配式建筑评价标准和方法。逐步建立完善覆盖设计、生产、施工和使用维护全过程的装配式建筑标准规范体系。

（五）创新装配式建筑设计。统筹建筑结构、机电设备、部品部件、装配施工、装饰装修，推行装配式建筑一体化集成设计。推广通用化、模数化、标准化设计方式，积极应用建筑信息模型技术，提高建筑领域各专业协同设计能力，加强对装配式建筑建设全过程的指导和服务。鼓励设计单位与科研院所、高校等联合开发装配式建筑设计技术和通用设计软件。

（六）优化部品部件生产。引导建筑行业部品部件生产企业合理布局，提高产业聚集度，培育一批技术先进、专业配套、管理规范的骨干企业和生产基地。支持部品部件生产企业完善产品品种和规格，促进专业化、标准化、规模化、信息化生产，优化物流管理，合理组织配送。积极引导设备制造企业研发部品部件生产装备机具，提高自动化和柔性加工技术水平。建立部品部件质量验收机制，确保产品质量。

（七）提升装配施工水平。引导企业研发应用与装配式施工相适应的技术、设备和机具，提高部品部件的装配施工连接质量和建筑安全性能。鼓励企业创新施工组织方式，推行绿色施工，应用结构工程与分部分项工程协同施工新模式。支持施工企业总结编制施工工法，提高装配施工技能，实现技术工艺、组织管理、技能队伍的转变，打造一批具有较高装配施工技术水平的骨干企业。

（八）推进建筑全装修。实行装配式建筑装饰装修与主体结构、机电设备协同施工。积极推广标准化、集成化、模块化的装修模式，促进整体厨卫、轻质隔墙等材料、产品和设备管线集成化技术的应用，提高装配化装修水平。倡导菜单式全装修，满足消费者个性化需求。

（九）推广绿色建材。提高绿色建材在装配式建筑中的应用比例。开发应用品质优良、节能环保、功能良好的新型建筑材料，并加快推进绿色建材评价。鼓励装饰与保温隔热材料一体化应用。推广应用高性能节能门窗。强制淘汰不符合节能环保要求、质量性能差的建筑材料，确保安全、绿色、环保。

（十）推行工程总承包。装配式建筑原则上应采用工程总承包模式，可按照技术复杂类工程项目招投标。工程总承包企业要对工程质量、安全、进度、造价负总责。要健全与装配式建筑总承包相适应的发包承包、施工许可、分包管理、工程造价、质量安全监管、竣工验收等制度，实现工程设计、部品部件生产、施工及采购的统一管理和深度融合，优化项目管理方式。鼓励建立装配式建筑产业技术创新联盟，加大研发投入，增强创新能力。支持大型设计、施工和部品部件生产企业通过调整组织架构、健全管理体系，向具有工程管理、设计、施工、生产、采购能力的工程总承包企业转型。

（十一）确保工程质量安全。完善装配式建筑工程质量安全管理制度，健全质量安全责任体系，落实各方主体质量安全责任。加强全过程监管，建设和监理等相关方可采用驻厂监造等方式加强部品部件生产质量管控；施工企业要加强施工过程质量安全控制和检验检测，完善装配施工质量保证体系；在建筑物明显部位设置永久性标牌，公示质量安全责任主体和主要责任人。加强行业监管，明确符合装配式建筑特点的施工图审查要求，建立全过程质量追溯制度，加大抽查抽测力度，严肃查处质量安全违法违规行为。

三、保障措施

（十二）加强组织领导。各地区要因地制宜研究提出发展装配式建筑的目标和任务，建立健全工作机制，完善配套政策，组织具体实施，确保各项任务落到实处。各有关部门要加大指导、协调和支持力度，将发展装配式建筑作为贯彻落实中央城市工作会议精神的重要工作，列入城市规划建设管理工作监督考核指标体系，定期通报考核结果。

（十三）加大政策支持。建立健全装配式建筑相关法律法规体系。结合节能减排、产业发展、科技创新、污染防治等方面政策，加大对装配式建筑的支持力度。支持符合高新技术企业条件的装配式建筑部品部件生产企业享受相关优惠政策。符合新型墙体材料目录的部品部件生产企业，可按规定享受增值税即征即退优惠政策。在土地供应中，可将发展装配式建筑的相关要求纳入供地方案，并落实到土地使用合同中。鼓励各地结合实际出台支持装配式建筑发展的规划审批、土地供应、基础设施配套、财政金融等相关政策措施。政府投资工程要带头发展装配式建筑，推动装配式建筑“走出去”。在中国人居环境奖评选、国家生态园林城市评估、绿色建筑评价等工作中增加装配式建筑方面的指标要求。

（十四）强化队伍建设。大力培养装配式建筑设计、生产、施工、管理等专业人才。鼓励高等学校、职业学校设置装配式建筑相关课程，推动装配式建筑企业开展校企合作，创新人才培养模式。在建筑行业专业技术人员继续教育中增加装配式建筑相关内容。加大职业技能培训资金投入，建立培训基地，加强岗位技能提升培训，促进建筑业农民工向技术工人转型。加强国际交流合作，积极引进海外专业人才参与装配式建筑的研发、生产和管理。

（十五）做好宣传引导。通过多种形式深入宣传发展装配式建筑的经济社会效益，广泛宣传装配式建筑基本知识，提高社会认知度，营造各方共同关注、支持装配式建筑发展的良好氛围，促进装配式建筑相关产业和市场发展。

　　　　　　　　　　　　　　　　　　　　　　　　　　国务院办公厅

　　　　　　　　　　　　　　　　　　　　　　　　　　2016年9月27日

**P10**

**Unified Standards for Building Information Model Application**

**建筑信息模型应用统一标准**

GB/T 51212-2016

1 总 则

1.0.1 为贯彻执行国家技术经济政策，推进工程建设信息化实 施，统一建筑信息模型应用基本要求，提高信息应用效率和效 益，制定本标准。

1.0.2 本标准适用于建设工程全生命期内建筑信息模型的创建、 使用和管理。

1.0.3 建筑信息模型应用，除应符合本标准外，尚应符合国家 现行有关标准的规定。

2 术语和缩略语

2.1 术 语

2.1.1 建筑信息模型 building information modeling,building information model(BIM)

在建设工程及设施全生命期内，对其物理和功能特性进行数 字化表达，并依此设计、施工、运营的过程和结果的总称。简称 模 型 。

2.1.2 建筑信息子模型 sub building information model(sub- BIM)

建筑信息模型中可独立支持特定任务或应用功能的模型子 集。简称子模型。

2.1.3 建筑信息模型元素 BIM element

建筑信息模型的基本组成单元。简称模型元素。

2.1.4 建筑信息模型软件 BIM software

对建筑信息模型进行创建、使用、管理的软件。简称 BIM 软 件 。

2.2 缩 略 语

2.2.1 P-BIM 基于工程实践的建筑信息模型应用方式 prac-

tice-based BIM mode

3 基 本 规 定

3.0.1 模型应用应能实现建设工程各相关方的协同工作、信息 共 享 。

3.0.2 模型应用宜贯穿建设工程全生命期，也可根据工程实际 情况在某一阶段或环节内应用。

3.0.3 模型应用宜采用基于工程实践的建筑信息模型应用方式 (P-BIM), 并应符合国家相关标准和管理流程的规定。

3.0.4 模型创建、使用和管理过程中，应采取措施保证信息 安 全 。

3.0.5 BIM 软件宜具有查验模型及其应用符合我国相关工程建 设标准的功能。

3.0.6 对 BIM 软件的专业技术水平、数据管理水平和数据互用 能力宜进行评估。

4 模型结构与扩展

4.1 一 般 规 定

4.1.1 模型中需要共享的数据应能在建设工程全生命期各个阶 段、各项任务和各相关方之间交换和应用。

4.1.2 通过不同途径获取的同一模型数据应具有唯一性。采用 不同方式表达的模型数据应具有一致性。

4.1.3 用于共享的模型元素应能在建设工程全生命期内被唯一 识 别 。

4.1.4 模型结构应具有开放性和可扩展性。

4.2 模 型 结 构

4.2.1 BIM软件宜采用开放的模型结构，也可采用自定义的 模型结构。BIM 软件创建的模型，其数据应能被完整提取和使 用。

4.2.2 模型结构由资源数据、共享元素、专业元素组成，可按 照不同应用需求形成子模型。

4.2.3 子模型应根据不同专业或任务需求创建和统一管理，并 确保相关子模型之间信息共享。

4.2.4 模型应根据建设工程各项任务的进展逐步细化，其详细 程度宜根据建设工程各项任务的需要和有关标准确定。

4.3 模 型 扩 展

4.3.1 模型扩展应根据专业或任务需要，增加模型元素种类及 模型元素数据。

4.3.2 增加模型元素种类宜采用实体扩展方式。增加模型元素 数据宜采用属性或属性集扩展方式。

4.3.3 模型元素宜根据适用范围、使用频率等进行创建、使用 和管理。

4.3.4 模型扩展不应改变原有模型结构，并应与原有模型结构 协调 一 致。

5 数 据 互 用

5.1 一 般 规 定

5.1.1 模型应满足建设工程全生命期协同工作的需要，支持各 个阶段、各项任务和各相关方获取、更新、管理信息。

5.1.2 模型交付应包含模型所有权的状态，模型的创建者、审 核者与更新者，模型创建、审核和更新的时间，以及所使用的软 件及版本。

5.1.3 建设工程各相关方之间模型数据互用协议应符合国家现 行有关标准的规定；当无相关标准时，应商定模型数据互用协 议，明确互用数据的内容、格式和验收条件。

5.1.4 建设工程全生命期各个阶段、各项任务的建筑信息模型 应用标准应明确模型数据交换内容与格式。

5.2 交付与交换

5.2.1 数据交付与交换前，应进行正确性、协调性和一致性检 查，检查应包括下列内容：

1 数据经过审核、清理；

2 数据是经过确认的版本；

3 数据内容、格式符合数据互用标准或数据互用协议。

5.2.2 互用数据的内容应根据专业或任务要求确定，并应符合 下列规定：

1 应包含任务承担方接收的模型数据；

2 应包含任务承担方交付的模型数据。

5.2.3 互用数据的格式应符合下列规定：

1 互用数据宜采用相同格式或兼容格式；

2 互用数据的格式转换应保证数据的正确性和完整性。

5.2.4 接收方在使用互用数据前，应进行核对和确认。

5.3 编码与存储

5.3.1 模型数据应根据模型创建、使用和管理的需要进行分类 和编码。分类和编码应满足数据互用的要求，并应符合建筑信息 模型数据分类和编码标准的规定。

5.3.2 模型数据应根据模型创建、使用和管理的要求，按建筑 信息模型存储标准进行存储。

5.3.3 模型数据的存储应满足数据安全的要求。

6 模 型 应 用

6.1 一 般 规 定

6.1.1 建设工程全生命期内，应根据各个阶段、各项任务的需 要创建、使用和管理模型，并应根据建设工程的实际条件，选择 合适的模型应用方式。

6.1.2 模型应用前，宜对建设工程各个阶段、各专业或任务的 工作流程进行调整和优化。

6.1.3 模型创建和使用应利用前一阶段或前置任务的模型数据， 交付后续阶段或后置任务创建模型所需要的相关数据，且应满足 本标准第5章的规定。

6.1.4 建设工程全生命期内，相关方应建立实现协同工作、数 据共享的支撑环境和条件。

6.1.5 模型的创建和使用应具有完善的数据存储与维护机制。

6.1.6 模型交付应满足各相关方合约要求及国家现行有关标准 的规定。

6.1.7 交付的模型、图纸、文档等相互之间应保持一致，并及 时保存。

6.2 BIM 软 件

6.2.1 BIM 软件应具有相应的专业功能和数据互用功能。

6.2.2 BIM 软件的专业功能应符合下列规定：

1 应满足专业或任务要求；

2 应符合相关工程建设标准及其强制性条文；

3 宜支持专业功能定制开发。

6.2.3 BIM 软件的数据互用功能应至少满足下列要求之一：

1 应支持开放的数据交换标准；

2 应实现与相关软件的数据交换；

3 应支持数据互用功能定制开发。

6.2.4 BIM 软件在工程应用前，宜对其专业功能和数据互用功 能进行测试。

6.3 模 型 创 建

6.3.1 模型创建前，应根据建设工程不同阶段、专业、任务的 需要，对模型及子模型的种类和数量进行总体规划。

6.3.2 模型可采用集成方式创建，也可采用分散方式按专业或 任务创建。

6.3.3 各相关方应根据任务需求建立统一的模型创建流程、坐 标系及度量单位、信息分类和命名等模型创建和管理规则。

6.3.4 不同类型或内容的模型创建宜采用数据格式相同或兼容 的软件。当采用数据格式不兼容的软件时，应能通过数据转换标 准或工具实现数据互用。

6.3.5 采用不同方式创建的模型之间应具有协调一致性。

6.4 模 型 使 用

6.4.1 模型的创建和使用宜与完成相关专业工作或任务同步 进行。

6.4.2 模型使用过程中，模型数据交换和更新可采用下列方式：

1 按单个或多个任务的需求，建立相应的工作流程；

2 完成一项任务的过程中，模型数据交换一次或多次完成；

3 从已形成的模型中提取满足任务需求的相关数据形成子 模型，并根据需要进行补充完善；

4 利用子模型完成任务，必要时使用完成任务生成的数据 更新模型。

6.4.3 对不同类型或内容的模型数据，宜进行统一管理和维护。

6.4.4 模型创建和使用过程中，应确定相关方各参与人员的管 理权限，并应针对更新进行版本控制。

6.5 组 织 实 施

6.5.1 企业应结合自身发展和信息化战略确立模型应用的目标、 重点和措施。

6.5.2 企业在模型应用过程中，宜将 BIM 软件与相关管理系统 相结合实施。

6.5.3 企业应建立支持建设工程数据共享、协同工作的环境和 条件，并结合建设工程相关方职责确定权限控制、版本控制及一 致性控制机制。

6.5.4 企业应按建设工程的特点和要求制定建筑信息模型应用 实施策略。实施策略宜包含下列内容：

1 工程概况、工作范围和进度，模型应用的深度和范围；

2 为所有子模型数据定义统一的通用坐标系；

3 建设工程应采用的数据标准及可能未遵循标准时的变通 方式；

4 完成任务拟使用的软件及软件之间数据互用性问题的解 决方案；

5 完成任务时执行相关工程建设标准的检查要求；

6 模型应用的负责人和核心协作团队及各方职责；

7 模型应用交付成果及交付格式；

8 各模型数据的责任人；

9 图纸和模型数据的一致性审核、确认流程；

10 模型数据交换方式及交换的频率和形式；

11 建设工程各相关方共同进行模型会审的日期。

本标准用词说明

1 为便于在执行本标准条文时区别对待，对要求严格程度 不同的用词说明如下：

1)表示很严格，非这样做不可的：

正面词采用“必须”,反面词采用“严禁”;

2)表示严格，在正常情况下均应这样做的：

正面词采用“应”,反面词采用“不应”或“不得”;

3)表示允许稍有选择，在条件许可时首先这样做的：

正面词采用“宜”,反面词采用“不宜”;

4) 表示有选择，在一定条件下可以这样做的，采用 “可”。

2 条文中指明应按其他有关标准执行的写法为： “应符 合……的规定”或“应按……执行”。

中华人民共和国国家标准

建筑信息模型应用统一标准

GB/T 51212-2016

条 文 说 明

制 订 说 明

《建筑信息模型应用统一标准》GB/T 51212-2016,经住房 和城乡建设部2016年12月2日以第1380号公告批准、发布。

本标准编制过程中，编制组进行了广泛的调查研究，组织了 大量的课题研究，总结了我国建筑信息模型应用的实践经验，同 时参考了有关国外技术标准，广泛征求了有关方面的意见，对具 体内容进行了反复讨论、协调和修改，最后经审查定稿。

《建筑信息模型应用统一标准》是我国第一部建筑信息模型 应用的工程建设标准，提出了建筑信息模型应用的基本要求，是 建筑信息模型应用的基础标准，可作为我国建筑信息模型应用及 相关标准研究和编制的依据。

为便于广大建设、勘察、设计、施工、工程监理、工程造 价、物业管理、构配件生产、软件、科研院所、学校等单位有关 人员在使用本标准时能正确理解和执行条文规定，《建筑信息模 型应用统一标准》编制组按章、节、条顺序编制了本标准的条文 说明，对条文规定的目的、依据以及执行中需要注意的有关事项 进行了说明。但是，本条文说明不具备与标准正文同等的法律效 力，仅供使用者作为理解和把握标准规定的参考。

中华人民共和国住房和城乡建设部 中华人民共和国国家质量监督检验检疫总局

联合发布

2016-12-02

**P11**

**Opinions on Promoting the Sustained and Healthy Development of the Construction Industry**

**国务院办公厅关于促进建筑业持续健康发展的意见**
国办发〔2017〕19号

各省、自治区、直辖市人民政府，国务院各部委、各直属机构：

建筑业是国民经济的支柱产业。改革开放以来，我国建筑业快速发展，建造能力不断增强，产业规模不断扩大，吸纳了大量农村转移劳动力，带动了大量关联产业，对经济社会发展、城乡建设和民生改善作出了重要贡献。但也要看到，建筑业仍然大而不强，监管体制机制不健全、工程建设组织方式落后、建筑设计水平有待提高、质量安全事故时有发生、市场违法违规行为较多、企业核心竞争力不强、工人技能素质偏低等问题较为突出。为贯彻落实《中共中央 国务院关于进一步加强城市规划建设管理工作的若干意见》，进一步深化建筑业“放管服”改革，加快产业升级，促进建筑业持续健康发展，为新型城镇化提供支撑，经国务院同意，现提出以下意见：

一、总体要求

全面贯彻党的十八大和十八届二中、三中、四中、五中、六中全会以及中央经济工作会议、中央城镇化工作会议、中央城市工作会议精神，深入贯彻习近平总书记系列重要讲话精神和治国理政新理念新思想新战略，认真落实党中央、国务院决策部署，统筹推进“五位一体”总体布局和协调推进“四个全面”战略布局，牢固树立和贯彻落实创新、协调、绿色、开放、共享的发展理念，坚持以推进供给侧结构性改革为主线，按照适用、经济、安全、绿色、美观的要求，深化建筑业“放管服”改革，完善监管体制机制，优化市场环境，提升工程质量安全水平，强化队伍建设，增强企业核心竞争力，促进建筑业持续健康发展，打造“中国建造”品牌。

二、深化建筑业简政放权改革

（一）优化资质资格管理。进一步简化工程建设企业资质类别和等级设置，减少不必要的资质认定。选择部分地区开展试点，对信用良好、具有相关专业技术能力、能够提供足额担保的企业，在其资质类别内放宽承揽业务范围限制，同时，加快完善信用体系、工程担保及个人执业资格等相关配套制度，加强事中事后监管。强化个人执业资格管理，明晰注册执业人员的权利、义务和责任，加大执业责任追究力度。有序发展个人执业事务所，推动建立个人执业保险制度。大力推行“互联网+政务服务”，实行“一站式”网上审批，进一步提高建筑领域行政审批效率。

（二）完善招标投标制度。加快修订《工程建设项目招标范围和规模标准规定》，缩小并严格界定必须进行招标的工程建设项目范围，放宽有关规模标准，防止工程建设项目实行招标“一刀切”。在民间投资的房屋建筑工程中，探索由建设单位自主决定发包方式。将依法必须招标的工程建设项目纳入统一的公共资源交易平台，遵循公平、公正、公开和诚信的原则，规范招标投标行为。进一步简化招标投标程序，尽快实现招标投标交易全过程电子化，推行网上异地评标。对依法通过竞争性谈判或单一来源方式确定供应商的政府采购工程建设项目，符合相应条件的应当颁发施工许可证。

三、完善工程建设组织模式

（三）加快推行工程总承包。装配式建筑原则上应采用工程总承包模式。政府投资工程应完善建设管理模式，带头推行工程总承包。加快完善工程总承包相关的招标投标、施工许可、竣工验收等制度规定。按照总承包负总责的原则，落实工程总承包单位在工程质量安全、进度控制、成本管理等方面的责任。除以暂估价形式包括在工程总承包范围内且依法必须进行招标的项目外，工程总承包单位可以直接发包总承包合同中涵盖的其他专业业务。

（四）培育全过程工程咨询。鼓励投资咨询、勘察、设计、监理、招标代理、造价等企业采取联合经营、并购重组等方式发展全过程工程咨询，培育一批具有国际水平的全过程工程咨询企业。制定全过程工程咨询服务技术标准和合同范本。政府投资工程应带头推行全过程工程咨询，鼓励非政府投资工程委托全过程工程咨询服务。在民用建筑项目中，充分发挥建筑师的主导作用，鼓励提供全过程工程咨询服务。

四、加强工程质量安全管理

（五）严格落实工程质量责任。全面落实各方主体的工程质量责任，特别要强化建设单位的首要责任和勘察、设计、施工单位的主体责任。严格执行工程质量终身责任制，在建筑物明显部位设置永久性标牌，公示质量责任主体和主要责任人。对违反有关规定、造成工程质量事故的，依法给予责任单位停业整顿、降低资质等级、吊销资质证书等行政处罚并通过国家企业信用信息公示系统予以公示，给予注册执业人员暂停执业、吊销资格证书、一定时间直至终身不得进入行业等处罚。对发生工程质量事故造成损失的，要依法追究经济赔偿责任，情节严重的要追究有关单位和人员的法律责任。参与房地产开发的建筑业企业应依法合规经营，提高住宅品质。

（六）加强安全生产管理。全面落实安全生产责任，加强施工现场安全防护，特别要强化对深基坑、高支模、起重机械等危险性较大的分部分项工程的管理，以及对不良地质地区重大工程项目的风险评估或论证。推进信息技术与安全生产深度融合，加快建设建筑施工安全监管信息系统，通过信息化手段加强安全生产管理。建立健全全覆盖、多层次、经常性的安全生产培训制度，提升从业人员安全素质以及各方主体的本质安全水平。

（七）全面提高监管水平。完善工程质量安全法律法规和管理制度，健全企业负责、政府监管、社会监督的工程质量安全保障体系。强化政府对工程质量的监管，明确监管范围，落实监管责任，加大抽查抽测力度，重点加强对涉及公共安全的工程地基基础、主体结构等部位和竣工验收等环节的监督检查。加强工程质量监督队伍建设，监督机构履行职能所需经费由同级财政预算全额保障。政府可采取购买服务的方式，委托具备条件的社会力量进行工程质量监督检查。推进工程质量安全标准化管理，督促各方主体健全质量安全管控机制。强化对工程监理的监管，选择部分地区开展监理单位向政府报告质量监理情况的试点。加强工程质量检测机构管理，严厉打击出具虚假报告等行为。推动发展工程质量保险。

五、优化建筑市场环境

（八）建立统一开放市场。打破区域市场准入壁垒，取消各地区、各行业在法律、行政法规和国务院规定外对建筑业企业设置的不合理准入条件；严禁擅自设立或变相设立审批、备案事项，为建筑业企业提供公平市场环境。完善全国建筑市场监管公共服务平台，加快实现与全国信用信息共享平台和国家企业信用信息公示系统的数据共享交换。建立建筑市场主体黑名单制度，依法依规全面公开企业和个人信用记录，接受社会监督。

（九）加强承包履约管理。引导承包企业以银行保函或担保公司保函的形式，向建设单位提供履约担保。对采用常规通用技术标准的政府投资工程，在原则上实行最低价中标的同时，有效发挥履约担保的作用，防止恶意低价中标，确保工程投资不超预算。严厉查处转包和违法分包等行为。完善工程量清单计价体系和工程造价信息发布机制，形成统一的工程造价计价规则，合理确定和有效控制工程造价。

（十）规范工程价款结算。审计机关应依法加强对以政府投资为主的公共工程建设项目的审计监督，建设单位不得将未完成审计作为延期工程结算、拖欠工程款的理由。未完成竣工结算的项目，有关部门不予办理产权登记。对长期拖欠工程款的单位不得批准新项目开工。严格执行工程预付款制度，及时按合同约定足额向承包单位支付预付款。通过工程款支付担保等经济、法律手段约束建设单位履约行为，预防拖欠工程款。

六、提高从业人员素质

（十一）加快培养建筑人才。积极培育既有国际视野又有民族自信的建筑师队伍。加快培养熟悉国际规则的建筑业高级管理人才。大力推进校企合作，培养建筑业专业人才。加强工程现场管理人员和建筑工人的教育培训。健全建筑业职业技能标准体系，全面实施建筑业技术工人职业技能鉴定制度。发展一批建筑工人技能鉴定机构，开展建筑工人技能评价工作。通过制定施工现场技能工人基本配备标准、发布各个技能等级和工种的人工成本信息等方式，引导企业将工资分配向关键技术技能岗位倾斜。大力弘扬工匠精神，培养高素质建筑工人，到2020年建筑业中级工技能水平以上的建筑工人数量达到300万，2025年达到1000万。

（十二）改革建筑用工制度。推动建筑业劳务企业转型，大力发展木工、电工、砌筑、钢筋制作等以作业为主的专业企业。以专业企业为建筑工人的主要载体，逐步实现建筑工人公司化、专业化管理。鼓励现有专业企业进一步做专做精，增强竞争力，推动形成一批以作业为主的建筑业专业企业。促进建筑业农民工向技术工人转型，着力稳定和扩大建筑业农民工就业创业。建立全国建筑工人管理服务信息平台，开展建筑工人实名制管理，记录建筑工人的身份信息、培训情况、职业技能、从业记录等信息，逐步实现全覆盖。

（十三）保护工人合法权益。全面落实劳动合同制度，加大监察力度，督促施工单位与招用的建筑工人依法签订劳动合同，到2020年基本实现劳动合同全覆盖。健全工资支付保障制度，按照谁用工谁负责和总承包负总责的原则，落实企业工资支付责任，依法按月足额发放工人工资。将存在拖欠工资行为的企业列入黑名单，对其采取限制市场准入等惩戒措施，情节严重的降低资质等级。建立健全与建筑业相适应的社会保险参保缴费方式，大力推进建筑施工单位参加工伤保险。施工单位应履行社会责任，不断改善建筑工人的工作环境，提升职业健康水平，促进建筑工人稳定就业。

七、推进建筑产业现代化

（十四）推广智能和装配式建筑。坚持标准化设计、工厂化生产、装配化施工、一体化装修、信息化管理、智能化应用，推动建造方式创新，大力发展装配式混凝土和钢结构建筑，在具备条件的地方倡导发展现代木结构建筑，不断提高装配式建筑在新建建筑中的比例。力争用10年左右的时间，使装配式建筑占新建建筑面积的比例达到30%。在新建建筑和既有建筑改造中推广普及智能化应用，完善智能化系统运行维护机制，实现建筑舒适安全、节能高效。

（十五）提升建筑设计水平。建筑设计应体现地域特征、民族特点和时代风貌，突出建筑使用功能及节能、节水、节地、节材和环保等要求，提供功能适用、经济合理、安全可靠、技术先进、环境协调的建筑设计产品。健全适应建筑设计特点的招标投标制度，推行设计团队招标、设计方案招标等方式。促进国内外建筑设计企业公平竞争，培育有国际竞争力的建筑设计队伍。倡导开展建筑评论，促进建筑设计理念的融合和升华。

（十六）加强技术研发应用。加快先进建造设备、智能设备的研发、制造和推广应用，提升各类施工机具的性能和效率，提高机械化施工程度。限制和淘汰落后、危险工艺工法，保障生产施工安全。积极支持建筑业科研工作，大幅提高技术创新对产业发展的贡献率。加快推进建筑信息模型（BIM）技术在规划、勘察、设计、施工和运营维护全过程的集成应用，实现工程建设项目全生命周期数据共享和信息化管理，为项目方案优化和科学决策提供依据，促进建筑业提质增效。

（十七）完善工程建设标准。整合精简强制性标准，适度提高安全、质量、性能、健康、节能等强制性指标要求，逐步提高标准水平。积极培育团体标准，鼓励具备相应能力的行业协会、产业联盟等主体共同制定满足市场和创新需要的标准，建立强制性标准与团体标准相结合的标准供给体制，增加标准有效供给。及时开展标准复审，加快标准修订，提高标准的时效性。加强科技研发与标准制定的信息沟通，建立全国工程建设标准专家委员会，为工程建设标准化工作提供技术支撑，提高标准的质量和水平。

八、加快建筑业企业“走出去”

（十八）加强中外标准衔接。积极开展中外标准对比研究，适应国际通行的标准内容结构、要素指标和相关术语，缩小中国标准与国外先进标准的技术差距。加大中国标准外文版翻译和宣传推广力度，以“一带一路”战略为引领，优先在对外投资、技术输出和援建工程项目中推广应用。积极参加国际标准认证、交流等活动，开展工程技术标准的双边合作。到2025年，实现工程建设国家标准全部有外文版。

（十九）提高对外承包能力。统筹协调建筑业“走出去”，充分发挥我国建筑业企业在高铁、公路、电力、港口、机场、油气长输管道、高层建筑等工程建设方面的比较优势，有目标、有重点、有组织地对外承包工程，参与“一带一路”建设。建筑业企业要加大对国际标准的研究力度，积极适应国际标准，加强对外承包工程质量、履约等方面管理，在援外住房等民生项目中发挥积极作用。鼓励大企业带动中小企业、沿海沿边地区企业合作“出海”，积极有序开拓国际市场，避免恶性竞争。引导对外承包工程企业向项目融资、设计咨询、后续运营维护管理等高附加值的领域有序拓展。推动企业提高属地化经营水平，实现与所在国家和地区互利共赢。

（二十）加大政策扶持力度。加强建筑业“走出去”相关主管部门间的沟通协调和信息共享。到2025年，与大部分“一带一路”沿线国家和地区签订双边工程建设合作备忘录，同时争取在双边自贸协定中纳入相关内容，推进建设领域执业资格国际互认。综合发挥各类金融工具的作用，重点支持对外经济合作中建筑领域的重大战略项目。借鉴国际通行的项目融资模式，按照风险可控、商业可持续原则，加大对建筑业“走出去”的金融支持力度。

各地区、各部门要高度重视深化建筑业改革工作，健全工作机制，明确任务分工，及时研究解决建筑业改革发展中的重大问题，完善相关政策，确保按期完成各项改革任务。加快推动修订建筑法、招标投标法等法律，完善相关法律法规。充分发挥协会商会熟悉行业、贴近企业的优势，及时反映企业诉求，反馈政策落实情况，发挥好规范行业秩序、建立从业人员行为准则、促进企业诚信经营等方面的自律作用。

　　　　　　　　　　　　　　　　　　　　　　　　　　　国务院办公厅

　　　　　　　　　　　　　　　　　　　　　　　　　　2017年2月21日

**P12**

**Construction Industry Development Plan for the 13th Five-Year Period**

**建筑业发展“十三五 ”规划**

建市[2017]98号

序 言

规划范围。根据国务院批准的住房城乡建设部“三定 ” 规定以及住房城乡建设部“十三五 ”专项规划编制工作安排， 本规划涵盖内容包括工程勘察设计、建筑施工、建设监理 、 工程造价等行业以及政府对建筑市场、工程质量安全、工程标准定额、建筑节能与技术进步等方面的监督管理工作。

规划背景。《国务院办公厅关于促进建筑业持续健康发 展的意见》（国办发〔2017〕19 号，以下简称《意见》 ）， 对进一步深化建筑业“放管服 ”改革，加快产业升级，促进 建筑业发展提出了具体要求。本规划旨在贯彻落实《意见》， 阐明“十三五 ”时期建筑业发展战略意图，明确发展目标和主要任务，推进建筑业持续健康发展。

规划编制。本规划是住房城乡建设事业“十三五 ”专项 规划之一。编制工作由住房城乡建设部建筑市场监管司牵 头，会同标准定额司、工程质量安全监管司、建筑节能与科 技司、人事司， 共同组织住房城乡建设部政策研究中心、中 国建筑业协会、中国勘察设计协会、中国建设监理协会 、中 国建设工程造价管理协会、中国建筑金属结构协会、 中国建筑节能协会等单位编制完成。

规划实施。本规划由各级住房城乡建设主管部门、各相 关行业组织以及工程勘察设计、建筑施工、建设监理、造价 咨询等单位实施。住房城乡建设部负责进行规划实施评估、规划调整、协调促进工作。

建筑业发展“十三五 ”规划

一、建筑业发展回顾

（一）发展成就。

“十二五 ”时期，我国建筑业发展取得了巨大成绩。全 国具有资质等级的施工总承包和专业承包企业完成建筑业 总产值年均增长 13.48%，建筑业增加值年均增长 8.99%；全 国工程勘察设计企业营业收入年均增长 23.19%；全国工程监 理企业营业收入年均增长 15.66%。2015 年，全社会建筑业 实现增加值 46547 亿元， 占国内生产总值的 6.79%；建筑业 从业人员达 5093.7 万人，占全国从业人员的 6.58%。建筑业 在国民经济中的支柱产业地位继续增强，为推进我国城乡建 设和新型城镇化发展，改善人民群众居住条件，吸纳农村转移劳动力，缓解社会就业压力做出重要贡献。

——设计建造能力显著提高。 “ 十二五 ”期间，我国 在高难度、大体量、技术复杂的超高层建筑、高速铁路 、公 路、水利工程、核电核能等领域具备完全自有知识产权的设 计建造能力，成功建设上海中心大厦、南水北调中线工程等 一大批设计理念先进、建造难度大、使用品质高的标志性工程，世界瞩目，成就辉煌。

——科技创新和信息化建设成效明显。“十二五 ”以 来，建筑业企业普遍加大科研投入，积极采用建筑业 10 项 新技术为代表的先进技术， 围绕承包项目开展关键技术研 究，提高创新能力，创造大批专利、工法，取得丰硕成果 。 加快推进信息化与建筑业的融合发展，建筑品质和建造效率 进一步提高。积极推进建筑市场监管信息化，基本建成全国 建筑市场监管公共服务平台，建筑市场监管方式发生根本性转变。

——建筑节能减排取得新进展。 “十二五 ”期间，建 筑节能法律法规体系初步形成，建筑节能标准进一步完善 。 供热计量和既有建筑节能改造力度加大，完成既有居住建筑 供热计量及节能改造面积 9.9 亿平方米，大型公共建筑节能 降耗提速，完成公共建筑节能改造面积 4450 万平方米，可 再生能源在建筑领域应用规模不断扩大。 积极推进绿色建 筑，建立集中示范城（区），在政府投资公益性建筑及大型公共建筑建设中全面推进绿色建筑行动，成效初步显现。

——行业人才队伍素质不断提高。 “ 十二五 ”期间， 行业专业人才队伍不断壮大，执业资格人员数量逐年增加。 截至 2015 年底，全国共有注册建筑师 5.5 万人，勘察设计 注册工程师 12.3 万人，注册监理工程师 16.6 万人，注册造 价工程师 15.0 万人，注册建造师 200 余万人。建筑业农民工技能培训力度不断加大，住房城乡建设系统培训建筑农民工 700 余万人，技能鉴定 500 余万人，建筑农民工培训覆盖面进一步扩大，技能素质水平进一步提升。

—— 国际市场开拓稳步增长。 “ 十二五 ”期间，我国 对外工程承包保持良好增长态势，对外工程承包营业额年均 增长 9.3%，新签合同额年均增长 10.8%。2015 年，对外承包 工程业务完成营业额 1540.7 亿美元，新签合同额 2100.7 亿 美元。企业在欧美等发达国家市场开拓取得新进展。企业海 外承揽工程项目形式更加丰富，投资开发建设、工程总承包 业务明显增加。企业进入国际工程承包前列的数量明显增多，国际竞争能力不断提升。

——建筑业发展环境持续优化。“ 十二五 ”期间，特 别是党的十八大以来，政府部门大力推进行政审批制度改 革，进一步简政放权，缩减归并企业资质种类，调整简化资 质标准，行政审批效率不断提高。积极推进统一建筑市场和 诚信体系建设，营造更加统一、公平的市场环境。开展工程 质量治理两年行动，严格执法，严厉打击建筑施工违法发包、 转包、违法分包等行为，落实工程建设五方主体项目负责人质量终身责任，保障工程质量，取得明显成效。

（二）主要问题。

——行业发展方式粗放。建筑业大而不强，仍属于粗放 式劳动密集型产业，企业规模化程度低，建设项目组织实施

方式和生产方式落后，产业现代化程度不高，技术创新能力不足，市场同质化竞争过度，企业负担较重，制约了建筑业企业总体竞争力提升。

——建筑工人技能素质不高。建筑工人普遍文化程度 低，年龄偏大，缺乏系统的技能培训和鉴定，直接影响工程 质量和安全。建筑业企业“只使用人、不培养人 ”的用工方 式，造成建筑工人组织化程度低、流动性大，技能水平低，

职业、技术素养与行业发展要求不匹配。

——监管体制机制不健全。行业监管方式带有计划经济 色彩，重审批、轻监管。监管信息化水平不高,工程担保、 工程保险、诚信管理等市场配套机制建设进展缓慢，市场机 制在行业准入清出、优胜劣汰方面作用不足，严重影响建筑业发展活力和资源配置效率。

二、指导思想、基本原则和发展目标

“十三五 ”时期，我国经济发展进入新常态，增速放缓， 结构优化升级，驱动力由投资驱动转向创新驱动。以发挥市 场在资源配置中起决定性作用和更好发挥政府作用为核心 的全面深化改革进入关键时期。新型城镇化、京津冀协调发 展、长江经济带发展和“一带一路 ”建设，形成建筑业未来 发展的重要推动力和宝贵机遇。尤为重要的是，党的十八大 以来，以习近平同志为核心的党中央毫不动摇地坚持和发展

中国特色社会主义，形成一系列治国理政新理念新思想新战略，为“十三五 ”时期深化建筑业改革，加快推进行业市场化、工业化、信息化、国际化提供了科学理论指导和行动指南。

综合判断，建筑业发展总体上仍处于重要战略机遇期， 也面临着市场风险增多、发展速度放缓的严峻挑战。必须准 确把握市场供需结构的重大变化，下决心转变依赖低成本要 素驱动的粗放增长方式，增强改革意识、创新意识，不断适 应新技术、新需求的建设能力调整及服务模式创新任务的需 要。必须积极应对产业结构不合理、创新任务艰巨、优秀人 才和优质劳动力供给不足等新挑战， 着力在健全市场机制、 推进建筑产业现代化、提升队伍素质、开拓国际市场上取得 突破，切实转变发展方式，增强发展动力，努力实现建筑业的转型升级。

（一）指导思想。

全面贯彻党的十八大和十八届三中、四中、五中、六中 全会精神，以马克思列宁主义、毛泽东思想、邓小平理论、 “三个代表 ”重要思想、科学发展观为指导，深入贯彻习近 平总书记系列重要讲话精神和治国理政新理念新思想新战 略，认真贯彻中央城镇化工作会议、 中央城市工作会议精神 和《意见》 ，牢固树立和贯彻创新、协调、绿色、开放 、共 享发展理念，以落实“适用、经济、绿色、美观 ”建筑方针为目标，以推进建筑业供给侧结构性改革为主线，以推进建筑产业现代化为抓手，以保障工程质量安全为核心，以优化建筑市场环境为保障，推动建造方式创新，深化监管方式改 革，着力提升建筑业企业核心竞争力，促进建筑业持续健康发展。

（二）基本原则。

——坚持科学发展。科学发展是建筑业发展的核心。必 须大力推行建筑业技术创新、管理创新和业态创新，加快传 统建筑业与先进制造技术、信息技术、节能技术等融合 ，以 创新带动产业组织结构调整和转型升级。必须把握发展新特 征， 加快转变建筑业生产方式，推广绿色建筑和绿色建材，全面提升建筑节能减排水平，实现建筑业可持续发展。

——坚持深化改革。改革是建筑业发展的动力。必须围 绕发挥市场在资源配置中的决定性作用和更好地发挥政府 作用，坚持推进建筑业供给侧结构性改革。以围绕体制机制 改革为重点，健全制度体系，破除制约科学发展的壁垒和障 碍，全面推动建筑业改革取得新突破，为建筑业发展提供持续动力。

——坚持质量安全为本。质量安全是建筑业发展的根本 要求。必须牢固树立底线思维，保障工程质量安全是一切工 作的出发点和立足点。必须健全质量安全保证体系，强化质 量安全监管，严格落实建设各方主体责任，构建更加科学合

理的工程质量安全责任及制度体系， 为建筑业发展夯实基础。

——坚持统筹国内国际两个市场。统一开放是建筑业发 展的必然要求。坚持建立统一开放的建筑市场，消除市场壁 垒，营造权力公开、机会均等、规则透明的建筑市场环境 。 以“一带一路 ”战略为引领，引导企业加快“走出去 ”步伐， 积极开拓国际市场，提高建筑企业的对外工程承包能力 ，推进有条件的企业实现国内国际两个市场共同发展。

（三）发展目标。

按照住房城乡建设事业“十三五 ”规划纲要的目标要求，今后五年建筑业发展的主要目标是：

——市场规模目标。以完成全社会固定资产投资建设任 务为基础，全国建筑业总产值年均增长 7%，建筑业增加值年 均增长 5.5%；全国工程勘察设计企业营业收入年均增长 7%； 全国工程监理、造价咨询、招标代理等工程咨询服务企业营 业收入年均增长 8%；全国建筑企业对外工程承包营业额年均

增长 6%，进一步巩固建筑业在国民经济中的支柱地位。

——产业结构调整目标。促进大型企业做优做强，形成 一批以开发建设一体化、全过程工程咨询服务、工程总承包 为业务主体、技术管理领先的龙头企业。大力发展专业化施 工，推进以特定产品、技术、工艺、工种、设备为基础的专 业承包企业快速发展。弘扬工匠精神，培育高素质建筑工人，到 2020 年建筑业中级工技能水平以上的建筑工人数量达到300 万。加强业态创新，推动以“互联网+ ”为特征的新型建筑承包服务方式和企业不断产生。

——技术进步目标。巩固保持超高层房屋建筑、高速铁 路、高速公路、大体量坝体、超长距离海上大桥、核电站等 领域的国际技术领先地位。加大信息化推广力度，应用 BIM 技术的新开工项目数量增加。 甲级工程勘察设计企业，一级 以上施工总承包企业技术研发投入占企业营业收入比重在“十二五 ”期末基础上提高 1 个百分点。

——建筑节能及绿色建筑发展目标。城镇新建民用建筑 全部达到节能标准要求， 能效水平比 2015 年提升 20%。到 2020 年，城镇绿色建筑占新建建筑比重达到 50%，新开工全 装修成品住宅面积达到 30%，绿色建材应用比例达到 40%。

装配式建筑面积占新建建筑面积比例达到 15%。

——建筑市场监管目标。加快修订建筑法等法律法规， 进一步完善建筑市场法律法规体系。工程担保、保险制度以 及与市场经济相适应的工程造价管理体系基本建立，建筑市 场准入制度更加科学完善，统一开放、公平有序的建筑市场 规则和格局基本形成。全国建筑工人培训、技能鉴定、职业 身份识别、信息管理系统基本完善。市场各方主体行为基本规范，建筑市场秩序明显好转。

——质量安全监管目标。建筑工程质量安全法规制度体系进一步完善，质量安全监管机制进一步健全，工程质量水平全面提升，国家重点工程质量保持国际先进水平。建筑安全生产形势稳定好转，建筑抗灾能力稳步提高。工程建设标准化改革取得阶段性成果。

三、 “十三五”时期主要任务

（一）深化建筑业体制机制改革。

改革承（发）包监管方式。缩小并严格界定必须进行招 标的工程建设项目范围，放宽有关规模标准。在民间投资的 房屋建筑工程中，试行由建设单位自主决定发包方式。完善 工程招标投标监管制度，落实招标人负责制，简化招标投标 程序，推进招标投标交易全过程电子化，促进招标投标过程 公开透明。对采用常规通用技术标准的政府投资工程，在原 则上实行最低价中标的同时，推行提供履约担保基础上的最低价中标， 制约恶意低价中标行为。

调整优化产业结构。以工程项目为核心，以先进技术应 用为手段，以专业分工为纽带，构建合理工程总分包关系， 建立总包管理有力，专业分包发达，组织形式扁平的项目组 织实施方式，形成专业齐全、分工合理、成龙配套的新型建 筑行业组织结构。发展行业的融资建设、工程总承包、施工 总承包管理能力，培育一批具有先进管理技术和国际竞争力的总承包企业。鼓励以技术专长、制造装配一体化、工序工种为基础的专业分包，促进基于专业能力的小微企业发展。支持“互联网+ ”模式整合资源，联通供需，降低成本。

提升工程咨询服务业发展质量。改革工程咨询服务委托 方式，研究制定咨询服务技术标准和合同范本，引导有能力 的企业开展项目投资咨询、工程勘察设计、施工招标咨询、 施工指导监督、工程竣工验收、项目运营管理等覆盖工程全 生命周期的一体化项目管理咨询服务，培育一批具有国际水 平的全过程工程咨询企业。提升建筑设计水平，健全适应建 筑设计特点的招标投标制度。完善注册建筑师制度，探索在 民用建筑项目中推行建筑师负责制。 完善工程监理制度，强化对工程监理的监管。

（二）推动建筑产业现代化。

推广智能和装配式建筑。加大政策支持力度，明确重点 应用领域，建立与装配式建筑相适应的工程建设管理制度。 鼓励企业进行工厂化制造、装配化施工、减少建筑垃圾，促 进建筑垃圾资源化利用。建设装配式建筑产业基地，推动装 配式混凝土结构、钢结构和现代木结构发展。大力发展钢结 构建筑，引导新建公共建筑优先采用钢结构，积极稳妥推广 钢结构住宅。在具备条件的地方，倡导发展现代木结构 ，鼓 励景区、农村建筑推广采用现代木结构。在新建建筑和既有 建筑改造中推广普及智能化应用，完善智能化系统运行维护机制，逐步推广智能建筑。

强化技术标准引领保障作用。加强建筑产业现代化标准建设，构建技术创新与技术标准制定快速转化机制，鼓励和 支持社会组织、企业编制团体标准、企业标准，建立装配式 建筑设计、部品部件生产、施工、质量检验检测、验收 、评 价等工程建设标准体系，完善模数协调、建筑部品协调等技 术标准，强化标准的权威性、公正性、科学性。建立以标准为依据的认证机制，约束工程和产品严格执行相关标准。

加强关键技术研发支撑。完善政产学研用协同创新机 制，着力优化新技术研发和应用环境，针对不同种类建筑产 品，总结推广先进建筑技术体系。组织资源投入，并支持产 业现代化基础研究，开展适用技术应用试点示范。培育国家 和区域性研发中心、技术人员培训中心，鼓励建设、工程勘 察设计、施工、构件生产和科研等单位建立产业联盟。加快 推进建筑信息模型（BIM）技术在规划、工程勘察设计、施 工和运营维护全过程的集成应用，支持基于具有自主知识产

权三维图形平台的国产 BIM 软件的研发和推广使用。

（三）推进建筑节能与绿色建筑发展。

提高建筑节能水平。推动北方采暖地区城镇新建居住建 筑普遍执行节能 75%的强制性标准。政府投资办公建筑、学 校、医院、文化等公益性公共建筑、保障性住房要率先执行 绿色建筑标准，鼓励有条件地区全面执行绿色建筑标准 。加

强建筑设计方案审查和施工图审查，确保新建建筑达到建筑节能要求。夏热冬冷、夏热冬暖地区探索实行比现行标准更 高节能水平的标准。积极开展超低能耗或近零能耗建筑示 范。大力发展绿色建筑，从使用材料、工艺等方面促进建筑 的绿色建造、品质升级。制定新建建筑全装修交付的鼓励政 策，提高新建住宅全装修成品交付比例，为用户提供标准化、 高品质服务。持续推进既有居住建筑节能改造，不断强化公共建筑节能管理，深入推进可再生能源建筑应用。

推广建筑节能技术。组织可再生能源、新型墙材和外墙 保温、高效节能门窗的研发。加快成熟建筑节能及绿色建筑 技术向标准的转化。加快推进绿色建筑、绿色建材评价标识 制度。建立全国绿色建筑和绿色建材评价标识管理信息平 台。开展绿色建造材料、工艺、技术、产品的独立和整合评 价，加强绿色建造技术、材料等的技术整合，推荐整体评价 的绿色建筑产品体系。选取典型地区和工程项目，开展绿色建材产业基地和工程应用试点示范。

推进绿色建筑规模化发展。制定完善绿色规划、绿色设 计、绿色施工、绿色运营等有关标准规范和评价体系。出台 绿色生态城区评价标准、生态城市规划技术准则，引导城市 绿色低碳循环发展。大力发展和使用绿色建材，充分利用可 再生能源，提升绿色建筑品质。加快建造工艺绿色化革新， 提升建造过程管理水平，控制施工过程水、土、声、光 、气污染。推动建筑废弃物的高效处理与再利用，实现工程建设全过程低碳环保、节能减排。完善监督管理机制。切实履行建筑节能减排监管责任， 构建建筑全生命期节能监管体系，加强对工程建设全过程执 行节能标准的监管和稽查。建立规范的能效数据统计报告制 度。严格明令淘汰建筑材料、工艺、部品部件的使用执法，保证节能减排标准执行到位。

（四）发展建筑产业工人队伍。

推动工人组织化和专业化。改革建筑用工制度，鼓励建 筑业企业培养和吸收一定数量自有技术工人。改革建筑劳务 用工组织形式，支持劳务班组成立木工、电工、砌筑、钢筋 制作等以作业为主的专业企业， 鼓励现有专业企业做专做 精，形成专业齐全、分工合理、成龙配套的新型建筑行业组 织结构。推行建筑劳务用工实名制管理，基本建立全国建筑 工人管理服务信息平台，记录建筑工人的身份信息、培训情 况、职业技能、从业记录等信息，构建统一的建筑工人职业

身份登记制度，逐步实现全覆盖。

健全技能培训和鉴定体系。建立政府引导、企业主导、 社会参与的建筑工人岗前培训、 岗位技能培训制度。研究优 惠政策，支持企业和培训机构开展工人岗前培训。发挥企业 在工人培训中的主导作用，积极开展工人岗位技能培训 。倡 导工匠精神，加大技能培训力度，发展一批建筑工人技能鉴定机构，试点开展建筑工人技能评价工作。改革完善技能鉴定制度，将技能水平与薪酬挂钩，引导企业将工资分配向关键技术技能岗位倾斜， 促进建筑业农民工向技术工人转型，努力营造重视技能、崇尚技能的行业氛围和社会环境。

完善权益保障机制。全面落实建筑工人劳动合同制度， 健全工资支付保障制度，落实工资月清月结制度，加大对拖 欠工资行为的打击力度，不断改善建筑工人的工作、生活环 境。探索与建筑业相适应的社会保险参保缴费方式，大力推 进建筑施工单位参加工伤保险。搭建劳务费纠纷争议快速调 解平台，引导有关企业和工人通过司法、仲裁等法律途径保障自身合法权益。

（五）深化建筑业“放管服”改革。

完善建筑市场准入制度。坚持弱化企业资质、强化个人 执业资格的改革方向，逐步构建资质许可、信用约束和经济 制衡相结合的建筑市场准入制度。改革建设工程企业资质管 理制度，加快修订企业资质标准和管理规定，简化企业资质 类别和等级设置，减少不必要的资质认定。推行“互联网+ 政务服务 ”，全面推进电子化审批，提高行政审批效率 。在 部分地区开展试点， 对信用良好、 具有相关专业技术能力、 能够提供足额履约担保的企业，在其资质类别内放宽承揽业 务范围限制。完善个人执业资格制度，优化建设领域个人执 业资格设置，严格落实注册执业人员权利、义务和责任 ，加大执业责任追究力度，严厉打击出租出借证书行为。有序发展个人执业事务所，推动建立个人执业保险制度。

改进工程造价管理体系。改革工程造价企业资质管理， 完善造价工程师执业格制度，建立健全与市场经济相适应 的工程造价管理体系。统一工程计价规则，完善工程量清单 计价体系，满足不同工程承包方式的计价需要。完善政府及 国有投资工程估算及概算计价依据的编制，提高工程定额编 制的科学性，及时准确反映工程造价构成要素的市场变化。 建立工程全寿命周期的成本核算制度，积极开展推动绿色建 筑、建筑产业现代化、城市地下综合管廊、海绵城市等各项 新型工程计价依据的编制。逐步实现工程造价信息的共享机

制，加强工程造价的监测及相关市场信息发布。

推进建筑市场的统一开放。打破区域市场准入壁垒，取 消各地区、各行业在法律法规和国务院规定外对企业设置的 不合理准入条件，严禁擅自设立或变相设立审批、备案事项。 加大对各地区设置市场壁垒、障碍的信息公开和问责力度 ， 为建筑企业提供公平市场环境。健全建筑市场监管和执法体 系，建立跨省承揽业务企业违法违规行为的查处督办、协调 机制，加强层级指导和监督，有效强化项目承建过程的事中事后监管。

加快诚信体系建设。加强履约管理，探索通过履约担保、 工程款支付担保等经济、法律手段约束建设单位和承包单位履约行为。研究制定信用信息采集和分类管理标准，完善全国建筑市场监管公共服务平台，加快实现与全国信用信息共

享平台和国家企业信用信息公示系统的数据共享交换 。建立 建筑市场主体黑名单制度，依法依规全面公开企业和个人信 用记录，接受社会监督。鼓励有条件的地区探索开展信用评 价，引导建设单位等市场主体通过市场化运作综合运用信用评价结果，营造“一处失信，处处受制 ”的建筑市场环境。

（六）提高工程质量安全水平。

严格落实工程质量安全责任。全面落实各方主体的工程 质量安全责任，强化建设单位的首要责任和勘察、设计、施 工、监理单位的主体责任。严格执行工程质量终身责任书面 承诺制、永久性标牌制、质量信息档案等制度。严肃查处质 量安全违法违规企业和人员，加大在企业资质、人员资格、 限制从业等方面的处罚力度，强化责任追究。推进工程质量 安全标准化管理，督促各方主体健全质量安全管控机制 ，提高工程质量安全管理水平。

全面提高质量监管水平。完善工程质量法律法规和管理 制度，健全企业负责、政府监管、社会监督的工程质量保障 体系。推进数字化审图，研究建立大型公共建筑后评估制度。 强化政府对工程质量的监管，充分发挥工程质量监督机构作 用，加强工程质量监督队伍建设，保障经费和人员，加大抽 查抽测力度，重点加强对涉及公共安全的工程地基基础 、主体结构等部位和竣工验收等环节的监督检查。探索推行政府以购买服务的方式，加强工程质量监督检查。加强工程质量 检测机构管理，严厉打击出具虚假报告等行为。推动发展工程质量保险。

强化建筑施工安全监管。健全完善建筑安全生产相关法 律法规、管理制度和责任体系。加强建筑施工安全监督队伍 建设，推进建筑施工安全监管规范化，完善随机抽查和差别 化监管机制，全面加强监督执法工作。完善对建筑施工企业 和工程项目安全生产标准化考评机制，提升建筑施工安全管 理水平。强化对深基坑、高支模、起重机械等危险性较大的 分部分项工程的管理，以及对不良地质地区重大工程项目的 风险评估或论证。建立完善轨道交通工程建设全过程风险控 制体系，确保质量安全水平。加快建设建筑施工安全监管信 息系统，通过信息化手段加强安全生产管理。建立健全全覆 盖、多层次、经常性的安全生产培训制度，提升从业人员安全素质以及各方主体的本质安全水平。

推进工程建设标准化建设。构建层级清晰、配套衔接的 新型工程建设标准体系。强化强制性标准、优化推荐性标准， 加强建筑业与建筑材料标准对接。培育团体标准，搞活企业 标准，为建筑业发展提供标准支撑。加强标准制定与技术创 新融合，通过提升标准水平，促进工程质量安全和建筑节能 水平提高。积极开展中外标准对比研究，提高中国标准与国际标准或发达国家标准的一致性。加强中国标准外文版译制，积极推广在当地适用的中国标准，提高中国标准国际认可度。建立新型城镇化标准图集体系，加快推进各项标准的 信息化应用。创新标准实施监督机制，加快构建强制性标准实施监督“双随机 ”机制。

（七）促进建筑业企业转型升级。

深化企业产权制度改革。建立以国有资产保值增值为核 心的国有建筑企业监管考核机制，放开企业的自主经营权 、 用人权和资源调配权，理顺并稳定分配关系，建立保证国有 资产保值增值的长效机制。科学稳妥推进产权制度改革步 伐，健全国有资本合理流动机制，引进社会资本，允许管理、 技术、资本等要素参与收益分配，探索发展混合所有制经济 的有效途径，规范董事会建设，完善国有企业法人治理结构， 建立市场化的选人用人机制。引导民营建筑企业继续优化产权结构，建立稳定的骨干队伍及科学有效的股权激励机制。

大力减轻企业负担。全面完成建筑业营业税改增值税改 革，加强调查研究和跟踪分析，完善相关政策，保证行业税 负只减不增。完善工程建设领域保留的投标、履约、工程质 量、农民工工资 4 类保证金管理制度。广泛推行银行保函， 逐步取代缴纳现金、预留工程款形式的各类保证金。逐步推 行工程款支付担保、预付款担保、履约担保、维修金担保等制度。

增强企业自主创新能力。鼓励企业坚持自主创新，引导企业建立自主创新的工作机制和激励制度。鼓励企业创建技术研发中心，加大科技研究专项投入，重点开发具有自主知 识产权的核心技术、专利和专有技术及产品，形成完备的科 研开发和技术运用体系。引导企业与工业企业、高等院校、 科研单位进行战略合作，开展产学研联合攻关，重点解决影 响行业发展的关键性技术。支持企业加大科技创新投入力度，加快科技成果的转化和应用，提高企业的技术创新水平。

（八）积极开拓国际市场。

加大市场开拓力度。充分把握“一带一路 ”战略契机， 发挥我国建筑业企业在高速铁路、公路、电力、港口、机场、 油气长输管道、高层建筑等工程建设方面的比较优势，培育 一批在融资、管理、人才、技术装备等方面核心竞争力强的 大型骨干企业，加大市场拓展力度，提高国际市场份额，打 造“ 中国建造 ”品牌。发挥融资建设优势，带动技术、设备、 建筑材料出口，加快建筑业和相关产业“走出去 ”步伐。鼓 励中央企业和地方企业合作， 大型企业和中小型企业合作， 共同有序开拓国际市场。引导企业有效利用当地资源拓展国际市场，实现更高程度的本土化运营。

提升风险防控能力。加强企业境外投资财务管理，防范 境外投资财务风险。加强地区和国别的风险研究，定期发布 重大国别风险评估报告，指导对外承包企业有效防范风险。完善国际承包工程信息发布平台，建立多部门协调的国际工程承包风险提示应急管理系统，提升企业风险防控能力。

加强政策支持。加大金融支持力度，综合发挥各类金融 工具作用，重点支持对外经济合作中建筑领域的重大战略项 目。完善与有关国家和地区在投资保护、税收、海关、人员 往来、执业资格和标准互认等方面的合作机制，签署双边或 多边合作备忘录，为企业“走出去 ”提供全方位的支持和保 障。加强信息披露，为企业提供金融、建设信息、投资贸易、风险提示、劳务合作等综合性的对外承包服务。

（九）发挥行业组织服务和自律作用。

充分发挥行业组织在订立行业规范及从业人员行为准 则、规范行业秩序、促进企业诚信经营、履行社会责任等方 面的自律作用。提高行业组织在促进行业技术进步、提升行 业管理水平、制定团体标准、反映企业诉求、反馈政策落实

情况、 提出政策建议等方面的服务能力。

中华人民共和国住房和城乡建设部
2017年4月26日

**P13**

**Several Opinions on Deepening the Integration of Production and Education**

**国务院办公厅关于深化产教融合的若干意见**

国办发〔2017〕95号

各省、自治区、直辖市人民政府，国务院各部委、各直属机构：

进入新世纪以来，我国教育事业蓬勃发展，为社会主义现代化建设培养输送了大批高素质人才，为加快发展壮大现代产业体系作出了重大贡献。但同时，受体制机制等多种因素影响，人才培养供给侧和产业需求侧在结构、质量、水平上还不能完全适应，“两张皮”问题仍然存在。深化产教融合，促进教育链、人才链与产业链、创新链有机衔接，是当前推进人力资源供给侧结构性改革的迫切要求，对新形势下全面提高教育质量、扩大就业创业、推进经济转型升级、培育经济发展新动能具有重要意义。为贯彻落实党的十九大精神，深化产教融合，全面提升人力资源质量，经国务院同意，现提出以下意见。

一、总体要求

（一）指导思想。

全面贯彻党的十九大精神，坚持以习近平新时代中国特色社会主义思想为指导，紧紧围绕统筹推进“五位一体”总体布局和协调推进“四个全面”战略布局，坚持以人民为中心，坚持新发展理念，认真落实党中央、国务院关于教育综合改革的决策部署，深化职业教育、高等教育等改革，发挥企业重要主体作用，促进人才培养供给侧和产业需求侧结构要素全方位融合，培养大批高素质创新人才和技术技能人才，为加快建设实体经济、科技创新、现代金融、人力资源协同发展的产业体系，增强产业核心竞争力，汇聚发展新动能提供有力支撑。

（二）原则和目标。

统筹协调，共同推进。将产教融合作为促进经济社会协调发展的重要举措，融入经济转型升级各环节，贯穿人才开发全过程，形成政府企业学校行业社会协同推进的工作格局。

服务需求，优化结构。面向产业和区域发展需求，完善教育资源布局，加快人才培养结构调整，创新教育组织形态，促进教育和产业联动发展。

校企协同，合作育人。充分调动企业参与产教融合的积极性和主动性，强化政策引导，鼓励先行先试，促进供需对接和流程再造，构建校企合作长效机制。

深化产教融合的主要目标是，逐步提高行业企业参与办学程度，健全多元化办学体制，全面推行校企协同育人，用10年左右时间，教育和产业统筹融合、良性互动的发展格局总体形成，需求导向的人才培养模式健全完善，人才教育供给与产业需求重大结构性矛盾基本解决，职业教育、高等教育对经济发展和产业升级的贡献显著增强。

二、构建教育和产业统筹融合发展格局

（三）同步规划产教融合与经济社会发展。制定实施经济社会发展规划，以及区域发展、产业发展、城市建设和重大生产力布局规划，要明确产教融合发展要求，将教育优先、人才先行融入各项政策。结合实施创新驱动发展、新型城镇化、制造强国战略，统筹优化教育和产业结构，同步规划产教融合发展政策措施、支持方式、实现途径和重大项目。

（四）统筹职业教育与区域发展布局。按照国家区域发展总体战略和主体功能区规划，优化职业教育布局，引导职业教育资源逐步向产业和人口集聚区集中。面向脱贫攻坚主战场，积极推进贫困地区学生到城市优质职业学校就学。加强东部对口西部、城市支援农村职业教育扶贫。支持中部打造全国重要的先进制造业职业教育基地。支持东北等老工业基地振兴发展急需的职业教育。加强京津冀、长江经济带城市间协同合作，引导各地结合区域功能、产业特点探索差别化职业教育发展路径。

（五）促进高等教育融入国家创新体系和新型城镇化建设。完善世界一流大学和一流学科建设推进机制，注重发挥对国家和区域创新中心发展的支撑引领作用。健全高等学校与行业骨干企业、中小微创业型企业紧密协同的创新生态系统，增强创新中心集聚人才资源、牵引产业升级能力。适应以城市群为主体的新型城镇化发展，合理布局高等教育资源，增强中小城市产业承载和创新能力，构建梯次有序、功能互补、资源共享、合作紧密的产教融合网络。

（六）推动学科专业建设与产业转型升级相适应。建立紧密对接产业链、创新链的学科专业体系。大力发展现代农业、智能制造、高端装备、新一代信息技术、生物医药、节能环保、新能源、新材料以及研发设计、数字创意、现代交通运输、高效物流、融资租赁、电子商务、服务外包等产业急需紧缺学科专业。积极支持家政、健康、养老、文化、旅游等社会领域专业发展，推进标准化、规范化、品牌化建设。加强智慧城市、智能建筑等城市可持续发展能力相关专业建设。大力支持集成电路、航空发动机及燃气轮机、网络安全、人工智能等事关国家战略、国家安全等学科专业建设。适应新一轮科技革命和产业变革及新经济发展，促进学科专业交叉融合，加快推进新工科建设。

（七）健全需求导向的人才培养结构调整机制。加快推进教育“放管服”改革，注重发挥市场机制配置非基本公共教育资源作用，强化就业市场对人才供给的有效调节。进一步完善高校毕业生就业质量年度报告发布制度，注重发挥行业组织人才需求预测、用人单位职业能力评价作用，把市场供求比例、就业质量作为学校设置调整学科专业、确定培养规模的重要依据。新增研究生招生计划向承担国家重大战略任务、积极推行校企协同育人的高校和学科倾斜。严格实行专业预警和退出机制，引导学校对设置雷同、就业连续不达标专业，及时调减或停止招生。

三、强化企业重要主体作用

（八）拓宽企业参与途径。鼓励企业以独资、合资、合作等方式依法参与举办职业教育、高等教育。坚持准入条件透明化、审批范围最小化，细化标准、简化流程、优化服务，改进办学准入条件和审批环节。通过购买服务、委托管理等，支持企业参与公办职业学校办学。鼓励有条件的地区探索推进职业学校股份制、混合所有制改革，允许企业以资本、技术、管理等要素依法参与办学并享有相应权利。

（九）深化“引企入教”改革。支持引导企业深度参与职业学校、高等学校教育教学改革，多种方式参与学校专业规划、教材开发、教学设计、课程设置、实习实训，促进企业需求融入人才培养环节。推行面向企业真实生产环境的任务式培养模式。职业学校新设专业原则上应有相关行业企业参与。鼓励企业依托或联合职业学校、高等学校设立产业学院和企业工作室、实验室、创新基地、实践基地。

（十）开展生产性实习实训。健全学生到企业实习实训制度。鼓励以引企驻校、引校进企、校企一体等方式，吸引优势企业与学校共建共享生产性实训基地。支持各地依托学校建设行业或区域性实训基地，带动中小微企业参与校企合作。通过探索购买服务、落实税收政策等方式，鼓励企业直接接收学生实习实训。推进实习实训规范化，保障学生享有获得合理报酬等合法权益。

（十一）以企业为主体推进协同创新和成果转化。支持企业、学校、科研院所围绕产业关键技术、核心工艺和共性问题开展协同创新，加快基础研究成果向产业技术转化。引导高校将企业生产一线实际需求作为工程技术研究选题的重要来源。完善财政科技计划管理，高校、科研机构牵头申请的应用型、工程技术研究项目原则上应有行业企业参与并制订成果转化方案。完善高校科研后评价体系，将成果转化作为项目和人才评价重要内容。继续加强企业技术中心和高校技术创新平台建设，鼓励企业和高校共建产业技术实验室、中试和工程化基地。利用产业投资基金支持高校创新成果和核心技术产业化。

（十二）强化企业职工在岗教育培训。落实企业职工培训制度，足额提取教育培训经费，确保教育培训经费60%以上用于一线职工。创新教育培训方式，鼓励企业向职业学校、高等学校和培训机构购买培训服务。鼓励有条件的企业开展职工技能竞赛，对参加培训提升技能等级的职工予以奖励或补贴。支持企业一线骨干技术人员技能提升，加强产能严重过剩行业转岗就业人员再就业培训。将不按规定提取使用教育培训经费并拒不改正的行为记入企业信用记录。

（十三）发挥骨干企业引领作用。鼓励区域、行业骨干企业联合职业学校、高等学校共同组建产教融合集团（联盟），带动中小企业参与，推进实体化运作。注重发挥国有企业特别是中央企业示范带头作用，支持各类企业依法参与校企合作。结合推进国有企业改革，支持有条件的国有企业继续办好做强职业学校。

四、推进产教融合人才培养改革

（十四）将工匠精神培育融入基础教育。将动手实践内容纳入中小学相关课程和学生综合素质评价。加强学校劳动教育，开展生产实践体验，支持学校聘请劳动模范和高技能人才兼职授课。组织开展“大国工匠进校园”活动。鼓励有条件的普通中学开设职业类选修课程，鼓励职业学校实训基地向普通中学开放。鼓励有条件的地方在大型企业、产业园区周边试点建设普职融通的综合高中。

（十五）推进产教协同育人。坚持职业教育校企合作、工学结合的办学制度，推进职业学校和企业联盟、与行业联合、同园区联结。大力发展校企双制、工学一体的技工教育。深化全日制职业学校办学体制改革，在技术性、实践性较强的专业，全面推行现代学徒制和企业新型学徒制，推动学校招生与企业招工相衔接，校企育人“双重主体”，学生学徒“双重身份”，学校、企业和学生三方权利义务关系明晰。实践性教学课时不少于总课时的50%。

健全高等教育学术人才和应用人才分类培养体系，提高应用型人才培养比重。推动高水平大学加强创新创业人才培养，为学生提供多样化成长路径。大力支持应用型本科和行业特色类高校建设，紧密围绕产业需求，强化实践教学，完善以应用型人才为主的培养体系。推进专业学位研究生产学结合培养模式改革，增强复合型人才培养能力。

（十六）加强产教融合师资队伍建设。支持企业技术和管理人才到学校任教，鼓励有条件的地方探索产业教师（导师）特设岗位计划。探索符合职业教育和应用型高校特点的教师资格标准和专业技术职务（职称）评聘办法。允许职业学校和高等学校依法依规自主聘请兼职教师和确定兼职报酬。推动职业学校、应用型本科高校与大中型企业合作建设“双师型”教师培养培训基地。完善职业学校和高等学校教师实践假期制度，支持在职教师定期到企业实践锻炼。

（十七）完善考试招生配套改革。加快高等职业学校分类招考，完善“文化素质+职业技能”评价方式。适度提高高等学校招收职业教育毕业生比例，建立复合型、创新型技术技能人才系统培养制度。逐步提高高等学校招收有工作实践经历人员的比例。

（十八）加快学校治理结构改革。建立健全职业学校和高等学校理事会制度，鼓励引入行业企业、科研院所、社会组织等多方参与。推动学校优化内部治理，充分体现一线教学科研机构自主权，积极发展跨学科、跨专业教学和科研组织。

（十九）创新教育培训服务供给。鼓励教育培训机构、行业企业联合开发优质教育资源，大力支持“互联网+教育培训”发展。支持有条件的社会组织整合校企资源，开发立体化、可选择的产业技术课程和职业培训包。推动探索高校和行业企业课程学分转换互认，允许和鼓励高校向行业企业和社会培训机构购买创新创业、前沿技术课程和教学服务。

五、促进产教供需双向对接

（二十）强化行业协调指导。行业主管部门要加强引导，通过职能转移、授权委托等方式，积极支持行业组织制定深化产教融合工作计划，开展人才需求预测、校企合作对接、教育教学指导、职业技能鉴定等服务。

（二十一）规范发展市场服务组织。鼓励地方政府、行业企业、学校通过购买服务、合作设立等方式，积极培育市场导向、对接供需、精准服务、规范运作的产教融合服务组织（企业）。支持利用市场合作和产业分工，提供专业化服务，构建校企利益共同体，形成稳定互惠的合作机制，促进校企紧密联结。

（二十二）打造信息服务平台。鼓励运用云计算、大数据等信息技术，建设市场化、专业化、开放共享的产教融合信息服务平台。依托平台汇聚区域和行业人才供需、校企合作、项目研发、技术服务等各类供求信息，向各类主体提供精准化产教融合信息发布、检索、推荐和相关增值服务。

（二十三）健全社会第三方评价。积极支持社会第三方机构开展产教融合效能评价，健全统计评价体系。强化监测评价结果运用，作为绩效考核、投入引导、试点开展、表彰激励的重要依据。

六、完善政策支持体系

（二十四）实施产教融合发展工程。“十三五”期间，支持一批中高等职业学校加强校企合作，共建共享技术技能实训设施。开展高水平应用型本科高校建设试点，加强产教融合实训环境、平台和载体建设。支持中西部普通本科高校面向产业需求，重点强化实践教学环节建设。支持世界一流大学和一流学科建设高校加强学科、人才、科研与产业互动，推进合作育人、协同创新和成果转化。

（二十五）落实财税用地等政策。优化政府投入，完善体现职业学校、应用型高校和行业特色类专业办学特点和成本的职业教育、高等教育拨款机制。职业学校、高等学校科研人员依法取得的科技成果转化奖励收入不纳入绩效工资，不纳入单位工资总额基数。各级财政、税务部门要把深化产教融合作为落实结构性减税政策，推进降成本、补短板的重要举措，落实社会力量举办教育有关财税政策，积极支持职业教育发展和企业参与办学。企业投资或与政府合作建设职业学校、高等学校的建设用地，按科教用地管理，符合《划拨用地目录》的，可通过划拨方式供地，鼓励企业自愿以出让、租赁方式取得土地。

（二十六）强化金融支持。鼓励金融机构按照风险可控、商业可持续原则支持产教融合项目。利用中国政企合作投资基金和国际金融组织、外国政府贷款，积极支持符合条件的产教融合项目建设。遵循相关程序、规则和章程，推动亚洲基础设施投资银行、丝路基金在业务领域内将“一带一路”职业教育项目纳入支持范围。引导银行业金融机构创新服务模式，开发适合产教融合项目特点的多元化融资品种，做好政府和社会资本合作模式的配套金融服务。积极支持符合条件的企业在资本市场进行股权融资，发行标准化债权产品，加大产教融合实训基地项目投资。加快发展学生实习责任保险和人身意外伤害保险，鼓励保险公司对现代学徒制、企业新型学徒制保险专门确定费率。

（二十七）开展产教融合建设试点。根据国家区域发展战略和产业布局，支持若干有较强代表性、影响力和改革意愿的城市、行业、企业开展试点。在认真总结试点经验基础上，鼓励第三方开展产教融合型城市和企业建设评价，完善支持激励政策。

（二十八）加强国际交流合作。鼓励职业学校、高等学校引进海外高层次人才和优质教育资源，开发符合国情、国际开放的校企合作培养人才和协同创新模式。探索构建应用技术教育创新国际合作网络，推动一批中外院校和企业结对联合培养国际化应用型人才。鼓励职业教育、高等教育参与配合“一带一路”建设和国际产能合作。

七、组织实施

（二十九）强化工作协调。加强组织领导，建立发展改革、教育、人力资源社会保障、财政、工业和信息化等部门密切配合，有关行业主管部门、国有资产监督管理部门积极参与的工作协调机制，加强协同联动，推进工作落实。各省级人民政府要结合本地实际制定具体实施办法。

（三十）营造良好环境。做好宣传动员和舆论引导，加快收入分配、企业用人制度以及学校编制、教学科研管理等配套改革，引导形成学校主动服务经济社会发展、企业重视“投资于人”的普遍共识，积极营造全社会充分理解、积极支持、主动参与产教融合的良好氛围。

国务院办公厅

2017年12月5日

**P14**

**Standards for Quality Inspection of Intelligent Building Engineering**

**智能建筑工程质量检测标准**

JGJ/T454-2019

1总则

1.0.1为加强智能建筑工程质量管理，规范智能建筑工程质量检测活动，保证智能建筑工程质量，制定本标准。

1.0.2本标准适用于新建、扩建和改建智能建筑工程质量的检测。

1.0.3智能建筑工程质量检测应以保证工程质量为目标，检测活动应方法科学、数据准确、程序规范，为工程验收提供依据。

1.0.4智能建筑工程的质量检测除应符合本标准外，尚应符合国家现行有关标准的规定。

2术语和缩略语

2.1术语

2.1.1智能建筑工程质量检测testing of quality of intelligent building systems

运用试验、测试等技术手段并通过专业知识的判断，确定工程的材料、设备，以及系统性能、运行功能等智能建筑工程实体质量特性的活动。

2.1.2智能建筑综合运行功效comprehensiveoperationfunc-tionandefficiencyofintelligentbuildings

为完成建筑整体的管理目标，建筑智能化系统通过智能化管理平台，实现系统之间信息共享，使建筑环境与建筑设备协调配合，表现出来的综合运行功能与效果。

2.1.3第三方检测third-partytesting

由处于建设单位和施工单位之外的一方，以公正、权威的非当事人身份，根据有关法律、标准和合同所进行的检测活动。

2.2缩略语

AAA——验证、授权和记账

AP——无线访问接入点

DHCP———动态主机配置协议

DNS——域名系统

DDOS——分布式拒绝服务

DDC——直接数字控制系统

ELTCTL——两端等效横向转换损耗

E-mail——电子邮件

FTP——文件传输协议

HFC——混合光纤同轴电缆网[HTTP](file:///C:\\Users\\oxq12\\Desktop\\政策文本计量分析\\样本与数据\\国家政策文本\\HTTP)——超文本传输协议

Internet——因特网

IP——网络互连协议

IPS—入侵防御系统

IPX——互联网数据包交换协议

ISP互联网服务提供商

MIBⅡ——第二代管理信息库

MAC——媒体访问控制

NAT——网络地址转换

OM3/OM4——一种多模光纤

OTDR——光时域反射仪

Ping—一种用于检查网络是否连通的操作命令

POE———有源以太网

POP3——邮局协议版本3

Portal———一种Web认证方法

QoS—服务质量

RPC——远程过程调用协议SMTP———简单邮件传输协议

S/N—信噪比

SNMP———简单网络管理协议

SSID——服务集标识

STIPA——扩声系统语言传输指数

TCL———横向变换损耗

TCP——传输控制协议

UDP——用户数据报协议VLAN—虚拟局域网

VGA——一种视频传输接口标准

Web——万维网

Wi-Fi——一种允许电子设备连接到无线局域网的技术

WLAN——无线局域网络

Y/C—(亮/色)分离信号接口

YPbPr——色差分量接口

3基本规定

3.1一般规定

3.1.1智能建筑工程应经过质量检测合格后进行验收。

3.1.2智能建筑工程质量检测范围应符合表3.1.2的规定，具体工程检测内容应根据工程设计文件涉及的智能化系统确定。

表3.1.2智能建筑工程质量检测范围

| 序号 | 系统名称 | 主要内容 |
| --- | --- | --- |
| 1 | 智能化集成系统 | 系统性能 |
|  |  | 系统功能 |
| 2  3 | 信息接入系统  用户电话交换系统 | 安装场地和环境  安装场地和环境 |
| 4 | 信息网络系统 | 计算机网络系统 |
|  |  | 网络安全 |
| 5 | 综合布线系统 | 电缆布线系统电气性能 |
|  |  | 光纤布线系统性能 |
|  |  | 布线管理系统功能 |
| 6 | 移动通信室内信号覆盖系统 | 安装场地和环境 |
| 7 | 卫星通信系统 | 安装场地和环境 |
| 8 | 有线电视及卫星电视接收系统 | 数字信号有线电视系统 |
|  |  | 模拟信号有线电视系统 |
|  |  | 卫星电视接收系统 |
| 9 | 公共广播系统 | 系统功能 |
|  |  | 电声性能 |
| 10 | 会议系统 | 会议扩声系统 |
|  |  | 会议视频显示系统 |

续表3.1.2

| 序号 | 系统名称 | 主要内容 |
| --- | --- | --- |
| 10 | 会议系统 | 会议灯光系统 |
|  |  | 会议电视系统 |
|  |  | 其他系统 |
| 11 | 信息导引及发布系统 | 信息播控设备 |
|  |  | 信息发布系统软件 |
|  |  | 信息显示屏 |
|  |  | 终端设备 |
| 12 | 时钟系统 | 标准时间源 |
|  |  | 石英谐振器母钟和子钟 |
|  |  | 时钟监控系统 |
| 13 | 信息化应用系统 | 硬件设备 |
|  |  | 应用软件 |
| 14 | 建筑设备管理系统 | 暖通空调监控系统 |
|  |  | 供配电监测系统 |
|  |  | 公共照明监控系统 |
|  |  | 给水排水监控系统 |
|  |  | 电梯和自动扶梯监测系统 |
|  |  | 能效监管系统 |
|  |  | 中央管理工作站 |
|  |  | 系统实时性、可靠性、可维护性及评测项目 |
| 15 | 安全技术防范系统 | 入侵报警系统 |
|  |  | 视频安防监控系统 |
|  |  | 出入口控制系统 |
|  |  | 电子巡查系统 |
|  |  | 停车库(场)管理系统 |
|  |  | 安全防范综合管理系统 |
| 16 | 应急响应系统 | 系统功能 |
|  |  | 系统性能 |

续表3.1.2

| 序号 | 系统名称 | 主要内容 |
| --- | --- | --- |
| 17 | 机房工程 | 供配电系统 |
|  |  | 空气调节系统 |
|  |  | 给水排水系统 |
|  |  | 监控与安全防范系统 |
|  |  | 机房室内环境 |
| 18 | 防雷与接地系统 | 接地装置 |
|  |  | 接地线 |
|  |  | 等电位联结 |
|  |  | 屏蔽设施 |
|  |  | 电涌保护器 |
|  |  | 各系统的防雷与接地 |

3.1.3智能建筑工程质量检测应按照先设备、后系统、再系统集成的顺序进行。

3.1.4智能建筑工程检测应提供检测数据和检测结论。检测结论分为合格和不合格两种。检测结论的判定依据应符合委托检测合同文件约定、工程设计文件要求和国家现行相关标准规定。

3.1.5火灾自动报警系统的性能、功能检测及检测方法应符合现行国家标准《火灾自动报警系统施工及验收规范》GB50166的有关规定。

3.1.6智能建筑工程质量检测报告应存入工程技术档案，作为工程验收的重要依据。

3.2检测活动

3.2.1智能建筑工程各系统检测，应以系统技术性能检测和系统运行功能检测为主，指标参数应量化，系统功能应验证。

3.2.2智能建筑工程综合运行功效检测应依据设计要求进行，应以智能化系统管理与建筑设备运行和建筑环境需求相融合的功

能集成为主，宜综合评价建筑物(群)内部智能化系统信息共享、协同动作的功能与效果。

3.2.3检测应按照本标准规定的方法和程序进行，用本标准规定之外的方法和程序取得的检测数据，采用时应经过建设单位组织的技术论证，并附技术认定过程文件。

3.2.4各系统检测记录应按本标准附录A～附录P的格式填写。对于系统规模大、检测数据多的工程，可按子系统或检测部位不同分别记录，也可以另附更详细的记录表格。

3.2.5检测现场条件应符合下列规定：

1系统安装、调试应完成，试运行应结束，并应自检合格；

2系统应正常带载运行；

3现场环境应符合检测设备要求；

4应有保证检测人员和检测设备安全工作的条件和措施。

3.2.6智能建筑工程质量的现场检测人员不应少于2人。

3.2.7智能建筑工程检测活动可依据检测合同，接受委托方和被检测方现场监督和见证。

3.3检测报告

3.3.1智能建筑工程质量检测报告的形式和内容应按本标准附录Q的格式填写。

3.3.2智能建筑工程质量检测报告中的数据应准确可靠。所有检测数据应有足够的现场检测记录支持。

3.3.3智能建筑工程质量检测报告应明确给出各系统检测结论。检测报告宜对建筑整体智能化系统综合运行功效进行评价。

3.3.4各系统检测结论应依据该系统检测项目和参数的检测结果判定。当其所有检测项目和参数全部合格时，该系统质量应为合格。

3.3.5建筑整体智能化系统综合运行功效评价应依据设计要求进行。评价宜从智能化系统的集成情况、信息共享程度、对建筑设备与环境的控制功能和系统整体的综合运行效果等方面进行。

3.3.6智能建筑工程质量不合格的项目和参数应整改直至重新检测合格。重新检测的抽样数量应加倍，当加倍抽样检测仍不合格时，整改后应全数检测。

4智能化集成系统

4.1一般规定

4.1.1智能建筑智能化集成系统工程质量检测内容应包括系统性能和系统功能检测。

4.1.2智能化集成系统检测应在被集成的子系统检测完成后进行。

4.1.3被集成子系统应全数检测，检测结果应符合设计要求。当设计文件无明确要求时，应符合国家现行标准要求。检测结果应全部符合相关要求。

4.2系统性能

4.2.1智能化集成系统网络性能检测应包括网络与接口性能。

4.2.2智能化集成系统软件性能检测应包括下列内容：

1软件静态数值性能；

2软件动态数值性能。

4.2.3智能化集成系统响应时间检测应包括下列内容：

1实时数据传送时间；

2控制命令传送时间；

3联动命令传送时间；

4数据存储时间；

5故障报警及查询时间。

4.3系统功能

4.3.1智能化集成系统整体功能检测应包括下列内容：

1系统构架；

2系统可靠性；

3系统冗余性。

4.3.2集中监视和管理功能检测应包括下列内容：

1通过统一界面显示各子系统设备运行数据和状态；

2支持同时在线的设备数量及用户数量以及并发访问能力。

4.3.3报警监视与处理功能检测应包括下列内容：

1报警数据显示和提醒；

2报警信息归类、排列和处理；

3数据显示的准确性。

4.3.4控制和调节功能检测应包括下列内容：

1控制和调节机电设备运行；

2手/自动模式切换；

3修改机电设备的启停状态和设定值；

4控制逻辑、运行模式的修改和编辑。

4.3.5跨系统联动配置与管理功能检测应包括下列内容：

1设置跨系统联动策略、流程及联动控制配置；

2根据预设模式，自动执行联动操作。

4.3.6数据分析和可视化功能检测应包括下列内容：

1实时信息和历史数据分析；

2可视化图形直观显示；

3趋势预测及辅助决策。

4.3.7信息和数据管理功能检测应包括下列内容：

1设备库的共享和管理；

2信息库的共享和管理；

3信息和数据的各类管理。

4.3.8综合管理和统一调度功能检测应包括下列内容：

1全局事件的调度和管理；

2控制策略和调度指挥策略的管理。

4.3.9远程和移动应用管理功能检测应包括下列内容：

1远程监视、控制和管理；

2移动端监视和管理。

4.3.10能耗管理功能检测应包括下列内容：

1能耗数据获取；

2能耗数据基本分析；

3能耗数据对标比较；

4用能超限警示。

4.3.11系统安全管理功能检测应包括下列内容：

1系统权限管理；

2系统账户管理；

3网络权限管理。

4.3.12文件报表生成和打印功能检测应包括下列内容：

1生成和打印报警记录及报表；

2生成和打印运行数据记录及报表；

3生成和打印用户日志记录及报表。

4.4检测设备

4.4.1计时器的准确度应为±0.1s。

4.4.2被集成子系统的检测设备，应符合本标准相应章节的有关规定。

4.5检测方法

4.5.1智能化集成系统网络性能检测应符合下列规定：

1根据网络拓扑图，检测工作站和任意一台网络设备的连通性能；

2检测各子网内用户之间的通信功能；

3检测局域网内的用户与公网之间的通信能力；

4通过手动设置接口相关参数，检测接口的性能。

4.5.2智能化集成系统软件性能检测应符合下列规定：

1软件静态数值性能，应对软件数据库进行检测；存储记录的条数与存储时间应满足设计要求；

2软件动态数值性能，应对软件从读取数据库到刷新用户

屏幕的响应时间进行检测，检测结果应为秒级，软件每秒的并发数据写入能力应满足设计要求。

4.5.3应使用计时器对智能化集成系统响应时间进行计时，实时数据传送时间、控制命令传送时间、联动命令传送时间、数据存储时间、故障报警及查询时间应满足设计要求。

4.5.4智能化集成系统整体功能检测可采用黑盒测试方法。检测应在服务器和客户端分别进行，监测点应包括每个被集成子系统，确保软件的整体架构、功能模块被全部检测，并应符合下列规定：

1系统架构检测，软件应满足建筑的业务功能、物业运营及管理模式的应用需求，软件应采用智能化信息资源共享和协同运行的架构形式；

2系统可靠性检测，软件应能对用户常见的误操作能进行提示；软件应能对重要动作的操作有警告和确认提示；软件应能判断数据的有效性，屏蔽用户的错误输入，识别非法值，并有相应的错误提示；当子系统出现故障，软件应能及时报警提示；

3系统冗余性检测，系统应具有双机热备及切换、数据库备份、备用电源及切换和通信链路的冗余切换功能，并能进行故障自诊断，事故情况下应有安全保障措施。

4.5.5智能化集成系统软件集中监视和管理功能检测可通过人工手动输入参数的方式观察监视、存储和统计的功能实现过程，并应符合下列规定：

1软件应能通过统一界面显示子系统各种数据，并能进行存储和统计，数据显示应与被集成子系统一致，界面上数据的位置与现场实际位置应一致，数据响应时间应满足设计要求；

2软件能够支持的同时在线设备数量及用户数量、并发访问能力应满足设计要求。

4.5.6智能化集成系统软件报警监视与处理功能检测可采用现场模拟报警信号的方法，观察报警信息的显示进行，并应符合下列规定：

1软件应能通过统一界面显示子系统报警数据以及报警概况图形、报警记录趋势图形，应能提供画面、声光、短信等多种报警方式；

2软件应能将报警信息按照产生时间、确认与否，以及优先级等信息排列。用户点击相应报警信息的条目，可在弹出窗口中看到该报警信息的具体信息和相应的处理状态，同时可按照预先设置发送给相应管理人员；

3软件数据显示应与被集成子系统一致。

4.5.7智能化集成系统软件控制和调节功能检测可在服务器和客户端分别输入设置参数，检测调节和控制的效果，并应符合下列规定：

1当通过集成系统设置参数，调节和控制子系统设备时，检测人员应可以浏览到机电设备的详细运行参数；

2软件应能够切换手/自动模式，并应能够在手动模式下，手动修改机电设备的启停状态，修改设定值；

3软件应能够修改局部闭环控制逻辑，能够修改、编辑运行模式。

4.5.8智能化集成系统软件联动配置与管理功能检测可现场逐项模拟触发信号，检测系统联动和管理的效果，并应符合下列规定：

1软件应能通过集成系统设置子系统之间的联动策略，实现跨系统之间的联动控制等；

2软件应能在用户选中该模式时，集成平台在相应的时段自动执行模式所规定的操作。

4.5.9智能化集成系统软件数据分析和可视化功能检测应通过人工操作的方式，查看图形显示功能，并应符合下列规定：

1软件应具有对实时信息和历史数据的分析能力，为整个建筑规范化运营、设备运行情况、设备维护预案、节能管理等业务提供标准化数据；

2软件应能提供柱状图、曲线图、折线图、饼图、散点图

等图形显示功能；

3软件应具有一定的趋势预测能力，为建筑的管理提供数据支撑和决策辅助。

4.5.10智能化集成系统信息和数据管理功能检测应通过人工操作、现场模拟的方式，查看建筑的资源和信息共享能力，并应符合下列规定：

1软件应具有共享设备数据库，并应能整合运营设备、通信设施以及公共设备等，满足统一调用、联动和共享等需求；

2软件应具有共享信息数据库，并应能接入和整合建筑信息、控制信息、报警信息、能耗信息以及业务运行需要的各类信息，实现系统信息的共享；

3软件应实现对各子系统信息和数据的统一管理，并应能进行存储和统计。

4.5.11智能化集成系统综合管理和统一调度功能检测应通过人工操作、现场模拟的方式，检测整个建筑的综合运行效果，并应符合下列规定：

1系统应结合规范化的管理方式和专业化的业务运行程序，设置满足建筑主体业务需求的跨系统联动策略和综合管理功能模块，并应能实现对全局事件的决策和管理功能；

2系统应具有控制策略和联动策略优化的功能。

4.5.12智能化集成系统远程和移动应用管理功能检测应通过人工操作的方式，利用远程和移动端应用，对智能化集成系统进行查看、控制和管理。

4.5.13智能化集成系统能耗管理功能检测可采用人工操作的方式，并应符合下列规定：

1软件应具有从能耗检测系统读取用能分项计量的数据，并应具有分析和对比等基本定量分析功能；

2软件宜具有能耗数据的对标功能，并能通过与标杆数据的比较，实现节能优化管理；

3软件应具有用能超限警示功能。

4.5.14智能化集成系统安全管理功能检测可采用人工操作的方式，并应符合下列规定：

1系统权限管理功能方面，软件应具有集中统一用户注册管理功能，并能根据注册用户的权限，设定每个用户对各个系统和设备的浏览、操作、编辑权限。系统用户可分为一般用户、技术用户和管理员用户三类。系统可预设三类用户的默认权限，形成默认用户类，在编辑每个用户的权限时，可在所选择默认用户类型的基础上修改具体的管理权限。

2系统账户管理功能方面，软件应实现所有用户只有在输入正确的用户名和密码，成功登录后才能访问集成平台。用户登录后可注销、切换用户。系统中的用户密码和密钥应以密文方式存储。软件应有记录运行日志功能，即保存用户操作日志、故障报警日志、各子系统运行日志、软件重要操作日志。

3网络权限管理功能方面，所有通过网络远程访问系统的客户端，应在输入正确的用户名和密码，成功登录后才能访问集成平台。

4.5.15智能化集成系统文件报表生成和打印功能检测应通过人工操作的方式，检测文件报表的生成并打印，并应符合下列规定：

1软件应具有历史报警记录功能，可根据报警类型、报警级别等过滤条件进行查询及统计，并可按用户定制格式生成和打印报表；

2软件应具有历史数据记录功能，可根据点名称、点值、时间段等过滤条件进行查询及统计，并可按用户定制格式生成和打印报表；

3软件应具有用户日志记录功能，可根据过滤条件对不同类型日志按用户定制格式生成和打印报表。

5信息接入系统

5.0.1智能建筑信息接入系统工程质量检测内容应包括设备安装场地和环境相关项目。

5.0.2信息接入系统检测范围应根据设计要求确定。

5.0.3检测项目应符合本标准第20章的有关规定，并应符合设计要求。

5.0.4检测设备、检测数量、检测方法及合格判定方法等应符合本标准第20章的有关规定。

6用户电话交换系统

6.0.1智能建筑用户电话交换系统工程质量检测内容应包括设备安装场地和环境相关项目。

6.0.2用户电话交换系统检测范围应根据设计要求确定。

6.0.3检测项目应符合本标准第20章的有关规定，并应符合设计要求。

6.0.4检测设备、检测数量、检测方法及合格判定方法等应符合本标准第20章的有关规定。

7信息网络系统

7.1一般规定

7.1.1智能建筑信息网络系统工程质量检测内容应包括计算机网络系统检测和网络安全检测。

7.1.2检测环境及条件除应符合本标准第3.2.5条规定外，尚应符合下列规定：

1系统工作环境应满足设计要求。

2信息网络系统检测应充分考虑建筑内信息网络系统组网的架构规划设计，检测项目的选择应与设计要求及工程实际使用的功能相符合。

3根据承载业务的不同，智能建筑信息网络系统可分为业务办公网和智能化设备网，检测项目选择应与信息网络业务特点和要求相符合。

4信息网络系统所选择的设备应符合相应产品标准及设计需要，信息网络设备应获得3C认证。系统安全专用产品应获得公安部计算机管理监察部门审批颁发的计算机信息系统安全专用产品销售许可证。

7.2计算机网络系统

7.2.1计算机网络系统的检测应包括系统功能、系统性能、系统应用、管理功能、无线局域网功能、无线局域网性能等。

7.2.2系统功能检测应包括下列内容：

1IP子网划分；

2VLAN划分；

3QoS设定；

4用户接入多ISP;

5NAT功能；

6AAA认证；

7DHCP功能；

8设备和线路备份；

9组播等功能。

7.2.3系统性能检测应包括下列内容：

1系统连通性；

2链路传输速率；

3吞吐率；

4传输时延；

5丢包率；

6链路层健康状况指标：链路利用率、错误率及各类错误、广播帧和组播帧和冲突率或碰撞率。

7.2.4系统应用检测应包括下列内容：

1DHCP服务；

2DNS服务；

3Web访问服务；

4E-mail服务和文件服务。

7.2.5管理功能检测应包括下列内容：

1配置管理功能；

2告警管理功能；

3性能管理功能；

4安全管理功能；

5管理信息库。

7.2.6无线局域网功能检测应包括下列内容：

1AP配置；

2用户隔离控制；

3AP间切换；

4热点压力测试；

5热点吞吐量测试。

7.2.7无线局域网性能检测应包括下列内容：

1信号覆盖强度；

2信噪比；

3同邻频干扰；

4AP关联平均时间；

5AP关联成功率；

6Portal页面弹出时延；

7Portal页面弹出成功率；

8Web认证时长；

9Web认证成功率；

10[HTTP](file:///C:\Users\oxq12\Desktop\政策文本计量分析\样本与数据\国家政策文本\HTTP)页面响应时延；

11[HTTP](file:///C:\Users\oxq12\Desktop\政策文本计量分析\样本与数据\国家政策文本\HTTP)完整显示时延；

12[HTTP](file:///C:\Users\oxq12\Desktop\政策文本计量分析\样本与数据\国家政策文本\HTTP)页面访问成功率；

13Ping测试平均时延；

14Ping测试成功率；

15FTP下载平均速率；

16FTP上传平均速率；

17手机终端关联WLAN网络测试；

18手机终端Portal认证上网测试；

19手机终端数据吞吐率。

7.2.8接入层链路应按总数的10%抽样检测，且抽样数不应少于10路，链路少于10路的，应全部检测。汇聚层到核心层的链路、核心层的骨干链路应全部检测。无线局域网WLAN热点设计中要求的覆盖区域不少于95%,测试点应符合用户习惯和行为，不应在非用户使用地点进行检测。检测结果符合设计要求应判定为合格；当设计要求未做规定时，检测结果应符合本标准的相关规定。

7.3网络安全

7.3.1网络安全系统检测应包含下列内容：

1结构安全；

2访问控制；

3安全审计；

4边界完整性；

5入侵防范；

6恶意代码防范；

7网络设备防护；

8数据流控制；

9用户数据保密性；

10剩余信息保护；

11可信路径；

12抗抵赖；

13网络安全监控。

7.3.2对于要求物理隔离的计算机网络系统，物理隔离方式应符合下列规定：

1物理实体上应完全分开；

2不应存在共享的物理设备；

3不应有任何链路上的连接。

7.3.3网络安全系统应全数检测，相同功能的设备应抽检1台。检测结果符合设计要求应判定为合格，信息安全等级保护定级的信息系统还应符合现行国家标准《信息安全技术信息系统安全等级保护基本要求》GB/T22239的有关规定。

7.4检测设备

7.4.1用于计算机网络系统功能检测的设备，应符合下列规定：

1应具备直接网络流量监听功能，能够对网络利用率、单播帧、广播帧、多播帧、碰撞、各种类型的出错帧进行统计；

2应能统计网络中产生业务量最多的节点、出错最多的节点、产生广播帧和多播帧最多的节点；

3应具备网络协议分析功能，能对网络中的协议进行解码

和流量分布统计；

4应具备自动网络节点和拓扑发现功能，能自动生成网络节点列表，包括节点的MAC地址、IP/IPX地址和名称的对应；

5应具备网络流量仿真功能，可指定数据包的内容和数据包长度，并可指定所产生流量的大小；

6应具备Ping和TraceRoute检测功能；

7应具备从网络设备上获取SNMP数据的功能；

8应具备检测结果分析及图表打印输出的功能；

9宜具备基本网络业务仿真检测功能；

10宜具备无线局域网功能检测功能。

7.4.2用于计算机网络系统性能检测的设备，应符合下列规定：

1应支持在10M/100M/1000M/10G以太网接口上的100%满线速流量产生功能；

2应具备RFC2544网络性能检测功能，包括吞吐率、传输时延和丢包率检测；

3应支持在10M/100M/1000M/10G以太网接口上的100%满线速流量统计功能；

410M/100M/1000M以太网测试时间标签精度应为10μs,10G以太网测试时间标签精度应为lμs。

7.4.3用于无线局域网检测的设备，应符合下列规定：

1WLAN测试卡应为支持802.11a/b/g/n/ac的外置测试卡；

2WLAN专用测试仪表应能够测试WLAN场强、信噪比，能够分析出测试区域内的同频干扰、邻频干扰及用户使用情况，同时可完成AP关联测试、Web接入认证、网络Ping包测试、FTP上下行速率测试、网站访问成功率测试、定时自动测试和多网卡压力测试；

3WLAN软件分析工具应能够采集、分析无线Wi-Fi协议参数工具，对WLAN协议抓包、解码，并进行分析。

7.4.4用于网络安全检测的设备，应符合下列规定：

1应具备自身安全防护能力，包括用户身份鉴别功能、扫描范围限制功能、策略涉及的敏感信息加密存储、完整的使用记录、扫描结果的完整性和机密性；

2应能对网络安全系统的脆弱性进行扫描，包括网络服务脆弱性、操作系统脆弱性、数据库脆弱性、中间件脆弱性、应用服务脆弱性、木马等，可以发现安全问题并能提供相应的安全建议；

3应能对网络旁路进行检测，可检查目标系统网段中存在的连通外网网络旁路，如代理服务器、拨号上网等；

4应能获取操作系统类型、版本号、已开启的各项TCP/IP服务、系统硬件信息、系统软件配置信息、系统网络配置信息、共享目录信息、系统运行状态信息等；

5应能对端口进行扫描，包括RPC端口、TCP端口、UDP端口，并可对端口协议进行分析；

6应提供用户权限和角色划分功能，至少应包括授权管理员、普通管理员、审计员；

7应能对扫描结果数据进行分析，并生成扫描结果报告，报告可支持导入、导出和删除，格式应包括主流文档格式；

8应提供扫描策略的定制功能，可根据不同扫描要求定制不同级别的策略；

9应具有完整的日志和审计功能；

10应在脆弱性探测的强度和深度上提供一定的控制手段，以避免对被扫描系统造成严重危害；

11应具有模拟典型的攻击、病毒和僵尸网络的功能；

12应具有通过仿真攻击、病毒的收发端，来验证安全网络架构中防火墙、IPS和抗DDOS攻击设备等的识别、报警和拦截等效率指标；

13应能够模拟出网络的典型恶意行为，如垃圾邮件、钓鱼网站和关键敏感信息等内容，来验证网络对于这些恶意行为的识别报警和拦截能力。

7.5检测方法

7.5.1计算机网络系统的系统功能检测应符合下列规定：

1IP子网划分检测结构如图7.5.1-1所示。在局域网系统中的路由器或三层交换机上进行子网检测，应至少存在两个子网，可将测试计算机1连接到一个子网的物理端口，测试计算机2连接到另一个子网的物理端口。检测应按下列步骤进行：

1)可通过测试计算机1向测试计算机2发送Ping,共发送10次，查看它们之间的连通性；

2)可将测试工具连接在被测子网的某一物理端口上，测试工具应通过发送Ping广播报文、SNMP查询、监听网络中数据包等方式，自动检测出在该子网上所连接的所有设备和终端，并应生成该子网的节点列表。


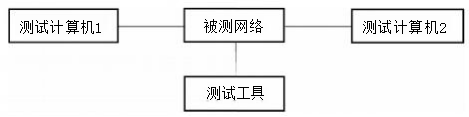


图7.5.1-1IP子网划分检测结构示意

2VLAN划分检测结构如图7.5.1-2所示。测试工具1应产生流量，测试工具2应接收流量。检测应按下列步骤进行：


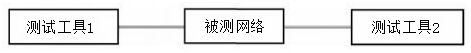


图7.5.1-2VLAN划分检测结构示意

1)在局域网系统中应进行VLAN划分，至少应划分两个VLAN;

2)应将测试工具1连接到一个VLAN的物理端口，测试工具2连接到另一个VLAN的物理端口；

3)应通过测试工具1向测试工具2发送Ping,共发送

10次，查看它们之间的连通性；

4)测试工具应通过发送Ping广播报文、SNMP查询、监听网络中数据包等方式，自动检测出在该子网上所连接的所有设备和终端，并应生成该VLAN的节点列表；

5)应通过测试工具1发送以太网广播包，测试工具2应能够接收到测试工具1发出的广播包；

6)应将测试工具2连接到与测试工具1所在的同一个VLAN的任一端口；

7)应通过测试工具1发送以太网广播包，测试工具2应能够正确接收到测试工具1发出的广播包。

3QoS功能检测结构如图7.5.1-3所示。测试工具1应产生流量，测试工具2应接收流量，测试工具3应统计丢弃包的情况。检测应按下列步骤进行：


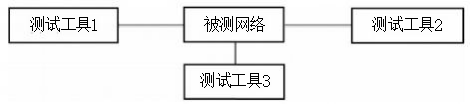


图7.5.1-3QoS功能检测结构示意

1)在局域网系统中应基于端口优先级配置一条具有QoS服务质量保证的链路，并在一端接上测试工具1,另一端接上测试工具2;

2)应由测试工具1向测试工具2发送端口号为80的UDP数据包，当用测试工具2捕获网络中的数据包时，测试工具1发出的数据包应被打上优先级的标记；

3)应逐渐加大被测网络内的负载流量，直至网络拥塞，统计测试工具2收到测试工具1发出的数据包的情况，测试工具2应收到测试工具1发出的全部数据包；

4)应用测试工具3统计被测网络数据包丢弃的状况；

5)应删除基于端口划分的优先级，再分别基于IP地址划分不同优先级；重复步骤2)～4)方法时，被测网络应保证高优先级IP地址报文的优先转发。

4用户接入多ISP功能检测结构如图7.5.1-4所示。测试工具3、测试工具4应模拟2个不同的ISP。检测应按下列步骤进行：

1)当测试工具1通过被测网络分别访问测试工具3和测试工具4时，应都能正常访问；

2)当测试工具2通过被测网络分别访问测试工具3和测试工具4时，应都能正常访问；

3)当断开测试工具3和被测网络的连接时，测试工具1通过被测网络访问测试工具4,正常访问不应受影响。


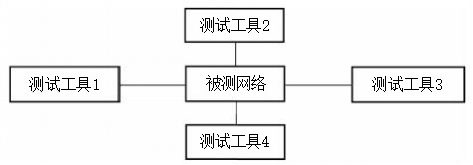


图7.5.1-4用户接入多ISP功能检测结构示意

5NAT功能检测结构如图7.5.1-5所示。对于公网IP地址缺乏的局域网系统，应能够支持NAT功能，来实现局域网系统内部用户对Internet公网上的资源访问。检测应按下列步骤进行：

1)在局域网系统中，应将网络设备上的NAT功能打开；

2)应将测试计算机1和测试计算机2连接到局域网上的接入用户端口，并应分别配置不同的内部网络IP地址；

3)应使用测试计算机1和测试计算机2同时访问Inter-net上某个公网IP地址，测试计算机1和测试计算机


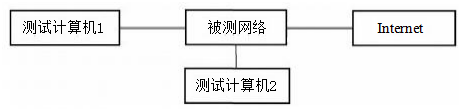


图7.5.1-5NAT功能检测结构示意

2应能同时连接到该公网IP地址。

6AAA功能检测结构如图7.5.1-6所示。检测可按下列步骤进行：

1)应在局域网系统中启用AAA功能；AAA服务器应正常运行；

2)测试计算机不经AAA认证，直接访问局域网外的地址，应无法直接访问；测试计算机经过AAA认证后，再访问局域网外的地址，应能够正常访问；

3)在测试计算机通过AAA认证一定时间后，检查AAA服务器上的记录，认证通过时间及访问局域网外的数据流量统计应准确；

4)测试计算机通过AAA认证3min后正常断开与网络的连接，2min后检查AAA服务器上面的记录，离线时间应符合计算机实际断开网络的时间；

5)测试计算机通过AAA认证3min后拔去测试计算机的网络连接线，5min后检查AAA服务器上面的记录，离线时间应符合计算机实际断开网络的时间。


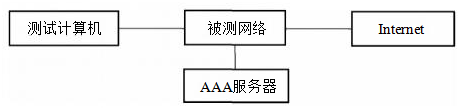


图7.5.1-6AAA功能检测结构示意

7DHCP功能检测结构如图7.5.1-7所示，测试计算机应支持自动获取IP地址功能。检测应按下列步骤进行：

1)应在局域网系统中启用DHCP功能，并应将测试计算机设置成自动获取IP地址模式；

2)重新启动测试计算机，应能够自动获得了IP地址及其他网络配置信息，如子网掩码、缺省网关地址和DNS服务器等。


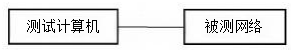


图7.5.1-7DHCP功能检测结构示意

8设备和线路备份功能检测结构如图7.5.1-8所示，测试计算机和测试目标节点之间的数据流应经过网络的主用设备和线路。检测应按下列步骤进行：

1)应由测试计算机向测试目标节点发送持续的Ping包，二者之间应连通；

2)应人为关闭核心层网络主设备电源，备份设备应启用，并且测试计算机和检测目标节点之间Ping应连通；

3)应人为断开主干线路，备份线路应启用，并且测试计算机和测试目标节点之间Ping应连通。


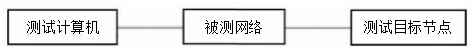


图7.5.1-8设备和线路备份功能检测结构示意

9组播功能检测结构如图7.5.1-9所示，组播服务器用于提供各种组播业务。检测应按下列步骤进行：


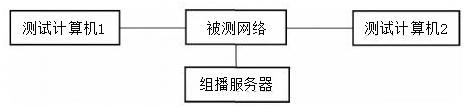


图7.5.1-9组播功能检测结构示意

1)应在被测链路中开启两组不同的组播业务；

2)应在测试计算机1和测试计算机2上同时点播第一组组播业务，分析被测网络与组播服务器间的数据流，组播服务器和网络之间应只有一条业务数据流，测试计算机1和测试计算机2应同时收到组播业务；

3)应在测试计算机1点播第一组组播业务，并在测试计算机2上点播第二组组播业务，分析被测网络与组播服务器间的数据流，组播服务器和网络之间应只有两条不同业务数据流，测试计算机1和测试计算机2应只收到各自点播的组播业务。

7.5.2计算机网络系统的系统性能检测应符合下列规定：

1系统连通性检测结构如图7.5.2-1所示。检测应按下列步骤进行：

1)应将测试工具连接到选定的接入层设备的端口，即测试点；

2)应由测试工具对网络的关键服务器、核心层和汇聚层的关键网络设备进行10次Ping测试，每次应间隔1s,被测网络应连通；测试路径应覆盖所有的子网和VLAN;

3)应将测试工具接入到其他位置测试点，重复步骤2)方法，应遍历所有测试抽样设备。


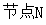

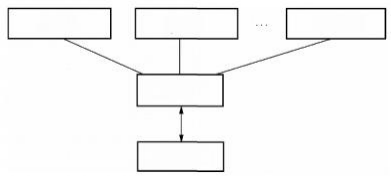
节点1节点2

被测网络

测试工具

图7.5.2-1系统连通性检测结构示意

2链路传输速率检测结构如图7.5.2-2所示。测试工具1应产生流量，测试工具2应接收流量。若发送端口和接收端口位

于同一机房，也可用一台具备双端口测试能力的测试工具实现。测试应在空载网络中进行。检测应按下列步骤进行：

1)应将测试工具1连接到被测网络链路的源交换机端口上，并将测试工具2连接到被测网络链路的目的交换机端口上；

2)测试工具1应在发送端口产生100%满线速流量，宜将帧长度设置为1518字节；

3)测试工具2应在接收端口对收到的流量进行统计，并计算其端口利用率。


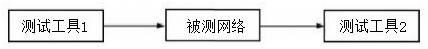


图7.5.2-2链路传输速率检测结构示意

3网络吞吐率检测结构如图7.5.2-3所示。测试工具1应产生流量，测试工具2应接收流量。若发送端口和接收端口位于同一机房，也可用一台具备双端口测试能力的测试工具实现。测试应在空载网络下分段进行，应包括接入层到汇聚层链路、汇聚层到核心层链路、核心层间骨干链路，及经过接入层、汇聚层和核心层的用户到用户链路。检测应按下列步骤进行：


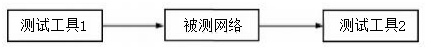


图7.5.2-3网络吞吐率检测结构示意

1)应将测试工具1连接到被测网络链路的源交换机端口上，并将测试工具2连接到被测网络链路的目的交换机端口上；

2)应由测试工具1向测试工具2发送数据包；

3)测试工具1应按照一定的帧速率，均匀地向被测网络发送一定数量的数据包；

4)当所有的数据包都被测试工具2正确接收到时，应增加发送的帧速率；否则应减少发送的帧速率；

5)重复步骤3)方法，应持续测试至测出被测网络或设备在未丢包的情况下能够处理的最大帧速率为止；

6)应分别按照不同的帧大小，包括64字节、128字节、256字节、512字节、1024字节、1280字节和1518字节，重复步骤2)～4)方法；

7)应由测试工具2向测试工具1发送数据包，重复步骤

3)～6)方法。

4传输时延检测时，若被测网络的收发端口位于不同的地理位置，检测结构如图7.5.2-4(a)所示。应由两台测试工具来完成，测试工具1应产生流量，测试工具2应接收流量，并将测试数据流环回。若被测网络的收发端口位于同一机房，测试结构如图7.5.2-4(b)所示。可由一台具有双端口测试能力测试工具完成，测试工具的一个端口应产生流量，另一个端口应接收流量。测试应在空载网络下分段进行，应包括接入层到汇聚层链路、汇聚层到核心层链路、核心层间骨干链路，及经过接入层、汇聚层和核心层的用户到用户链路。检测应按下列步骤进行：


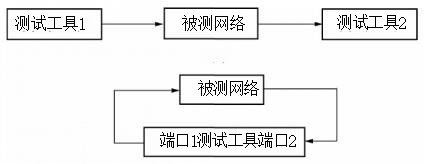


(b)

图7.5.2-4网络传输时延检测结构示意

1)应将测试工具1或测试端口1连接到被测网络链路的源交换机端口上，并将测试工具2或测试端口2连接到被测网络链路的目的交换机端口上；

2)应由测试工具1或测试端口1向测试工具2或测试端口2均匀地发送数据包；

3)应向被测网络发送一定数目的1518字节的数据帧，使网络达到7.5.2条第3款中所测得的最大吞吐率；

4)在图7.5.2-4(a)中，应由测试工具1向被测网络发送特定的测试帧，并在数据帧的发送和接收时刻都打上相应的时间标记；在图7.5.2-4(b)中，应由测试工具通过测试端口1发出带有时间标记的测试帧，并在测试端口2接收测试帧；

5)测试工具应计算发送和接收的时间标记之差，可得出一次测试结果；

6)重复步骤3)～4)方法20次，应对20次测试结果取平均值即为传输时延；

7)在图7.5.2-4(a)中，应由测试工具2向测试工具1发送数据包，并重复步骤3)～6)方法，所得到时延为双向往返时延，可通过除2计算获得单向时延；在图7.5.2-4(b)中，应交换端口1和端口2连接，并重复步骤3)～6)方法，所得到时延为单向时延。

5丢包率检测结构如图7.5.2-5所示。测试工具1应产生流量，测试工具2应接收流量。若发送端口和接收端口位于同一机房，也可用一台具备双端口测试能力的测试工具实现。测试链路应分段进行，应包括接入层到汇聚层链路、汇聚层到核心层链路、核心层间骨干链路，及经过接入层、汇聚层和核心层的用户到用户链路。检测应按下列步骤进行：


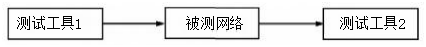


图7.5.2-5丢包率检测结构示意

1)应将测试工具1连接到被测网络链路的源交换机端口上，并将测试工具2连接到被测网络链路的目的交换机端口上；

2)应由测试工具1向被测网络加载70%的流量负荷，测

试工具2接收负荷，并测试数据帧丢失的比例；

3)应分别按照不同的帧大小，包括64字节、128字节、256字节、512字节、1024字节、1280字节和1518字节，重复步骤3)方法。

6以太网链路层健康状况检测结构如图7.5.2-6所示。对于共享式以太网，可将测试工具直接连接在空闲端口上；对于交换式以太网，可将测试工具串接在被监测的以太网链路上，如交换机和主机之间、交换机和路由器之间以及交换机和交换机之间。如果被测网络链路的设备端口具备SNMP流量监测功能，也可通过直接提取SNMP端口来替代测试工具。测试链路应分段进行，应包括接入层到汇聚层链路、汇聚层到核心层链路、核心层间骨干链路，及经过接入层、汇聚层和核心层的用户到用户链路。当进行以太网碰撞和出错率测试时，应保证在至少有30%的流量下进行。若没有达到该流量，则应人为加载一定的背景流量。检测应按下列步骤进行：


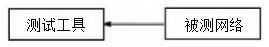


图7.5.2-6以太网链路层健康

状况检测结构示意

1)根据不同的网络类型，应按以上方式之一，将测试工具连接到网络中的某一网段；

2)应使用测试工具或通过SNMP流量监测功能，对被监测的网段进行流量统计，测试时间应达到5min以上，应测试广播和组播率、错误率、线路利用率、碰撞率等指标；

3)应将测试工具连接到其他网段，并重复步骤2)方法，直到遍历完所有需要测试的网段。

7.5.3计算机网络系统的系统应用检测应符合下列规定：

1DHCP服务性能检测结构如图7.5.3-1所示。检测应按

下列步骤进行：

1)应将测试工具连接到被测网络的某一用户接入端口或网段；

2)应用测试工具仿真一个终端用户，并由用户访问DH-CP服务器，应测试访问过程中DHCP服务器响应时间；如果测试工具未收到DHCP服务器的响应，则判定此次测试失败；

3)应按照一定的时间间隔重复步骤2)方法，进行10次测试，并记录10次测试结果的平均值；如果在测试过程中存在DHCP服务器无响应的情况，则判定测试失败；

4)应将测试工具连接到其他网段，重复步骤2)～3)方法，并测试网络不同接入位置访问DHCP服务器的性能水平。


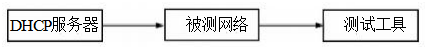


图7.5.3-1DHCP服务性能检测结构示意合格判据

2DNS服务性能检测结构如图7.5.3-2所示。检测应按下

列步骤进行：


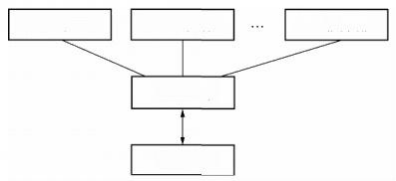
DNS服务器1DNS服务器2

被测网络

DNS服务器N

测试工具

图7.5.3-2DNS服务性能测试结构示意

1)应将测试工具连接到被测网络的某一用户接入端口或网段；

2)应用测试工具仿真一个终端用户，并由用户访问DNS服务器，应测试DNS服务器响应时间；如果测试工具未收到DNS服务器的响应，则判定此次测试失败；

3)应重复步骤2)方法，对下一个DNS服务器进行测试，直到测完所有为局域网提供服务的DNS服务器为止；

4)应按照一定的时间间隔，重复步骤2)～3)方法，进行10次测试，并记录10次测试结果的平均值；如果在测试过程中存在DNS服务器无响应的情况，则判定测试失败；

5)应将测试工具连接到其他网段，重复步骤2)～4)方法，并测试网络不同接入位置访问DNS服务器的性能水平。

3Web应用服务性能检测结构如图7.5.3-3所示。检测应按下列步骤进行：


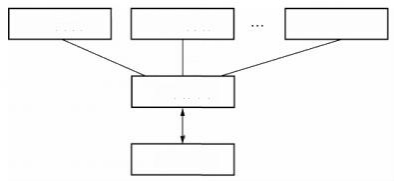
Web服务器1Web服务器2

被测网络

Web服务器N

测试工具

图7.5.3-3Web应用服务性能检测结构示意

1)应将测试工具连接到被测网络的某一用户接入端口或网段；

2)应用测试工具仿真Web一个终端用户，并由用户访问被测Web服务器所提供的网页服务，对访问过程中各阶段性能指标进行测试，包括[HTTP](file:///C:\Users\oxq12\Desktop\政策文本计量分析\样本与数据\国家政策文本\HTTP)第一响应时间和[HTTP](file:///C:\Users\oxq12\Desktop\政策文本计量分析\样本与数据\国家政策文本\HTTP)接收速率；

3)应重复步骤2)方法，对下一个Web服务器进行测

试，直到测完所有的Web服务器为止；

4)应按照一定的时间间隔，重复步骤2)～3)方法，进行10次测试，并记录10次测试结果的平均值；

5)应将测试工具连接到其他网段，重复步骤2)～3)方法，并测试网络不同接入位置访问Web服务的性能水平。

4E-mail应用服务性能检测结构如图7.5.3-4所示。检测应按下列步骤进行：


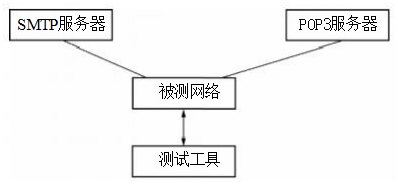


图7.5.3-4E-mail应用服务性能检测结构示意

1)应将测试工具连接到被测网络的某一用户接入端口或网段；

2)应用测试工具仿真E-mail的一个终端用户，并发送1KB大小的邮件，整个过程应包括测试工具向SMTP服务器发送一个邮件、SMTP服务器将邮件转发给POP3服务器、测试工具从POP3服务器下载该邮件以及测试工具对以上各阶段的邮件写入时间和邮件读取时间进行测试等4个阶段；

3)应重复步骤2)方法，对下一个E-mail服务器进行测试，直到测完所有的E-mail服务器为止；

4)应按照一定的时间间隔，重复步骤2)～3)方法，应进行10次测试，并记录10次测试结果的平均值；

5)应将测试工具连接到其他网段，重复步骤2)～4)方法，并测试网络不同接入位置访问E-mail服务的性能

水平。

5文件服务性能检测结构如图7.5.3-5所示。检测应按下列步骤进行：


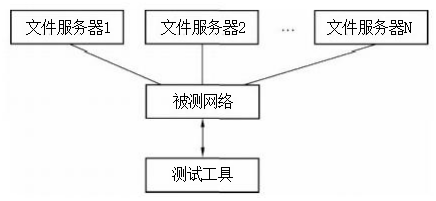


图7.5.3-5文件服务性能检测结构示意

1)应将测试工具连接到被测网络的某一用户接入端口或网段；

2)应用测试工具仿真文件服务器的终端用户，模拟一个用户访问被测文件服务器的全过程，应包括同文件服务器建立连接、向文件服务器指定目录写入一个100KB的文件、从服务器读取该文件、在服务器中删除该文件和断开同文件服务器的连接；

3)应测试访问过程中各阶段性能指标，包括服务器连接时间、写入速率、读取速率、删除时间和断开时间；

4)应重复步骤2)方法，对下一个文件服务器进行测试，直到测完所有的文件服务器为止；

5)应按照一定的时间间隔，重复步骤2)～3)方法，进行10次测试，并记录10次测试结果的平均值；

6)应将测试工具连接到其他网段，重复步骤2)～3)方法，并测试网络不同接入位置访问文件服务的性能水平。

7.5.4计算机网络系统的管理功能检测应符合下列规定。

1配置管理功能应包括设置和查看设备、端口和协议等信息，检测应按下列步骤进行：

1)应在局域网网管系统中选定一个网络设备；

2)应能够配置设备ID、IP地址、设备名称、网络标识、密码等信息，并应进行查看；

3)选择该设备中的一个端口，对该端口进行端口速率、端口管理状态、端口工作状态等设置，并进行查看；

4)交换机、路由器等网络设备的协议配置功能应参考《路由器设备测试方法边缘路由器》YD/T1098,《以太网交换机测试方法》YD/T1141,《路由器设备测试方法核心路由器》YD/T1156,《基于端口的虚拟局域网(VLAN)技术要求和测试方法》YD/T1260,《具有路由功能的以太网交换机测试方法》YD/T1287等协议配置的检测方法；

5)应选择另外的网络设备，重复步骤2)～4)方法。

2告警管理功能应包括告警信息配置、读取和管理功能，检测应按下列步骤进行：

1)应在局域网网管系统中选定一个网络设备；

2)应设置设备可产生告警信息的告警ID、告警级别、告警说明；

3)应设置设备中能够设置告警的限值；

4)应模拟制造设备故障，并查看网管上的告警信息；

5)应在一段时间内多次模拟制造不同类型的故障；

6)应在网管上对告警信息进行保存和备份；

7)应对告警信息进行查询；

8)应删除选定的告警信息；

9)应选择另外的网络设备，重复步骤2)～8)方法。

3性能管理功能应包括性能数据实时监视、性能数据采集和性能数据管理功能，检测应按下列步骤进行：

1)应在局域网网管系统中选定一个网络设备；

2)应选择需要实时监视的端口，显示端口的性能统计数据；

3)应选择需要采集的性能参数，并设定采集任务的开始时间和结束时间，进行数据采集；

4)应查看采集结果，并删除采集任务；

5)应对性能数据进行保存；

6)应对保存的性能数据进行查询，并以表格和图形的方式显示查询结果；

7)应选择另外的网络设备，重复步骤2)～6)方法。

4安全管理功能应包括访问控制、用户管理和日志管理功能，检测应按下列步骤进行：

1)打开登录局域网网管系统，输入正确用户名和口令后才能正常访问；

2)应在局域网系统中选择一个网络设备，并通过管理口连接设备进入命令行管理方式，输入正确用户名和口令后才能正常访问；

3)应分别通过登录局域网网管系统和网络设备进行用户管理，并进行添加、删除用户操作，应能够对已有用户信息进行查看和修改；

4)应分别通过登录局域网网管系统和网络设备进行日志信息查看，并对日志信息进行保存、查询和删除操作。

5管理信息库检测应按下列步骤进行：

1)应在局域网系统中选定一个网络设备；

2)应配置网络设备SNMP参数；

3)应通过网管接口向网络设备发送SNMP协议报文，并观察网络设备响应情况；

4)应通过网管接口查询网络设备MIBⅡ定义的所有管理对象，并观察网络设备响应情况；

5)应选择另外的网络设备，重复步骤2)～4)方法。

7.5.5计算机网络系统的无线局域网功能检测应符合下列规定：

1AP配置检测时，应使用WLAN测试仪表或专用网卡，在目标覆盖区域内用笔记本电脑通过测试软件测试所有AP信道

及SSID,并记录AP名称、覆盖范围、MAC、IP、无线接口方式等信息；

2用户隔离控制检测时，应使用两个终端分别通过Web认证方式接入网络，查看终端被分配的IP地址，并分别Ping对方的IP地址，两个终端Ping不通判定为通过，否则判定为不通过；

3AP间切换检测时，应使用笔记本终端通过Web认证方式接入网络，通过无线网卡Ping本地网关，终端由目前接入的AP切换至另一个AP的覆盖范围，并重复使终端在热点的各个AP间发生切换，切换次数应20次以上，应记录切换成功率；

4热点压力检测时，应使用专业测试仪表，并在同一热点AP下模拟多个用户同时接入WLAN网络下载50MB测试用文件，应记录每个用户下载速率指标及相关无线指标；

5热点吞吐量检测时，应将笔记本电脑终端通过登录认证接入到网络，并对服务器进行下载或上传100MB测试用文件。应记录在下载过程中明显中断的情况及下载和上传速率。

7.5.6计算机网络系统的无线局域网性能检测应符合下列规定。

1信号覆盖强度检测应按下列步骤进行：

1)应使用WLAN测试仪、测试卡或频谱仪，并在目标覆盖区域内用笔记本电脑通过测试软件进行覆盖电平测试；

2)每15m²测试地点不应少于1个，测试点的选取应均匀分布，并应能够反映该区域的覆盖情况；

3)如果AP为单独布放，则应对每个AP进行覆盖测试。

2信噪比检测应按下列步骤进行：

1)应使用WLAN测试仪、测试卡或频谱仪，并在目标覆盖区域内用笔记本电脑通过测试软件进行测试；

2)每15m²测试地点不应少于1个，测试点的选取应均匀分布，并应能够反映该区域的覆盖情况。

3同邻频干扰检测应按下列步骤进行：

1)明确某个AP覆盖的区域，应在AP覆盖区边缘选取测试点；

2)对于有多个AP同时使用的信道，应进行单信道测试，并查看同频干扰情况；

3)应观察在相邻信道上工作的AP情况，并查看邻频干扰情况。

4AP关联平均时间检测应按下列步骤进行：

1)应确定测试地点，每点应进行10次关联和去关联测试，SSID应设置为被测试的WLAN信号；

2)应记录用户开始连接网络到分配到有效IP的时间间隔。

5AP关联成功率检测应按下列步骤进行：

1)应确定测试地点，每点应进行10次关联和去关联测试，SSID应设置为被测试的WLAN信号；

2)应在用户选择“连接”SSID为被测试网络开始计时，应在60s内显示分配到有效IP地址。

6Portal页面弹出时延检测应按下列步骤进行：

1)客户端应关联成功，并随意打开一个网页；

2)每点应进行10次认证测试，SSID应设置为被测试WLAN信号。

7Portal页面弹出成功率检测应按下列步骤进行：

1)客户端应关联成功，并设定认证使用的账号和密码；

2)每点应进行10次认证测试，SSID应设置为被测试WLAN信号；

3)超时时间应设置为60s。

8Web认证时长检测应按下列步骤进行：

1)客户端应关联成功，并正确输入WLAN账号和密码；

2)每点应进行10次认证测试，SSID应设置为被测试WLAN信号；

3)应记录认证时长。

9Web认证成功率检测应按下列步骤进行：

1)客户端应关联成功，并正确输入WLAN账号和密码；

2)每点应进行10次认证测试，SSID应设置为被测试WLAN信号；

3)认证超时时间应设置为60s。

10[HTTP](file:///C:\Users\oxq12\Desktop\政策文本计量分析\样本与数据\国家政策文本\HTTP)页面响应时延检测应按下列步骤进行：

1)应分别对门户网站进行5次页面访问测试，并记录相应时间；

2)超时时间应设置为60s。

11[HTTP](file:///C:\Users\oxq12\Desktop\政策文本计量分析\样本与数据\国家政策文本\HTTP)完整显示时延检测可采用下列步骤进行：

1)应分别对门户网站进行5次页面访问测试，并记录相应时间；

2)超时时间应设置为60s。

12[HTTP](file:///C:\Users\oxq12\Desktop\政策文本计量分析\样本与数据\国家政策文本\HTTP)页面访问成功率检测应按下列步骤进行：

1)应分别对门户网站进行5次页面访问测试，并记录成功次数；

2)超时时间应设置为60s。

13Ping测试平均时延检测应按下列步骤进行：

1)应Ping该点AP的网关地址，Ping参数应设置为发送数据包20次、包大小为64字节的方式进行Ping测试，超时应为3s,间隔时间应为1s;

2)应记录Ping成功时数据包发出到接收到时的时间间隔。

14Ping测试成功率检测应按下列步骤进行：

1)应Ping该点AP的网关地址，Ping参数应设置为发送数据包20次、包大小为64字节的方式进行Ping测试，超时应为3s,间隔时间应为1s;

2)应记录Ping成功次数。

15FTP下载平均速率检测应按下列步骤进行：

1)服务器应位于内部本地网络，AP应支持802.11a/b/

g/n/ac协议，终端应加载可记录用户传输速率的软件和模拟多用户的测试软件；

2)用户应接入网络，并开启速率记录软件；

3)应使用802.11a/b/g协议进行10MB文件的FTP下载操作，并记录下载平均速率；应使用802.11n协议进行20MB文件的FTP下载操作，并记录下载平均速率；应使用802.11ac协议进行100MB文件的FTP下载操作，并记录下载平均速率。

16FTP上传平均速率检测应按下列步骤进行：

1)服务器应位于内部本地网络，AP应支持802.11a/b/g/n/ac协议，终端应加载可记录用户传输速率的软件，终端应加载模拟多用户的测试软件；

2)用户应接入网络，并开启速率记录软件；

3)应使用802.11a/b/g协议进行10MB文件的FTP上传操作，并使用802.11n协议进行20MB文件的FTP上传操作；应使用802.11ac协议进行100MB文件的FTP上传操作，并分别记录上传速率，若文件连续停传(无数据传送)超过60s认为上传失败。

17手机终端关联WLAN网络检测应按下列步骤进行：

1)应将手机测试终端的网卡设置为自动获取IP地址；

2)应接入搜索到的热点SSID,并验证测试终端能否通过接入AP获取规划的IP地址。

18手机终端Portal认证上网检测应按下列步骤进行：

1)手机应接入WLAN网络；

2)应打开浏览器，并输入任意网站，待弹出认证页面后进行认证；

3)应下载较大文件，并于大约30s后记录稳定的下行平均速率；

4)应将手机终端设置为休眠状态后下载较大文件，并于大约30s后记录稳定的下行平均速率。

19终端数据吞吐率检测应按下列步骤进行：

1)手机应接入WLAN网络；

2)应在无线信号强情况下，记录单用户接入最大下行业务速率。

7.5.7网络安全系统检测应根据设计要求的项目，按现行国家标准《信息安全技术信息系统安全等级保护实施指南》GB/T

25058、《信息安全技术信息系统安全等级保护测评过程指南》GB/T28449规定的方法进行检测。

8综合布线系统

8.1一般规定

8.1.1智能建筑综合布线系统工程质量检测内容应包括电缆布线系统电气性能、光纤布线系统性能和布线管理系统功能。

8.1.2综合布线系统检测单项合格判定应符合下列规定：

1一个及以上被测项目技术参数检测结果不合格的，该项目应为不合格；某一被测项目的检测结果与相应规定的差值在仪表准确度范围内的，该被测项目应为合格；

2采用4对对绞电缆作为水平电缆或主干电缆，所组成的链路或信道有一项及以上指标检测结果不合格的，该链路或信道应为不合格；

3主干布线大对数电缆中按4对对绞线对组成的链路一项及以上检测指标不合格的，该线对应为不合格；

4光纤链路或信道检测结果不满足设计要求的，该光纤链路或信道应为不合格；

5检测未通过的链路或信道应在修复后复检。

8.1.3综合布线系统检测的综合合格判定应符合下列规定：

1当对绞电缆布线全部检测时，无法修复的链路、信道或不合格线对的数量有一项及以上超过被测总数1%的，结论应为不合格；光缆布线检测，有一条及以上光纤链路或信道无法修复的，结论应为不合格。

2对于抽样检测，被抽样检测点(线对)不合格比例不大于被测总数1%的，抽样检测为合格，且不合格点(线对)应予以修复并复检；被抽样检测点(线对)不合格比例大于1%的，为一次抽样检测不合格，应进行加倍抽样，加倍抽样不合格比例不大于1%的，抽样检测为合格。不合格比例仍大于1%的，抽

样检测应为不合格，且应进行全数检测，并应按全数检测要求进行判定。

3全数检测或抽样检测结论为合格的，系统检测的结论应为合格；全数检测结论为不合格的，系统检测的结论应为不合格。

8.2电缆布线系统电气性能

8.2.1各等级电缆布线系统电气性能检测项目应符合表8.2.1的规定。

表8.2.1电缆布线系统电气性能检测项目

| 等级  检测项目 | C级 | D级 | E级 | EA级 | F级 | FA级 |
| --- | --- | --- | --- | --- | --- | --- |
| 连接图 | √ | √ | √ | √ | √ |  |
| 长度 | √ | √ | √ | √ | √ | √ |
| 回波损耗(RL) | √ | √ | √ | √ | √ | √ |
| 插入损耗(IL) | √ | √ | √ | √ | √ | √ |
| 近端串音(NEXT) | √ | √ | √ | √ | √ |  |
| 近端串音功率和(PSNEXT) | — | √ | √ | √ | √ | √ |
| 衰减串音比(ACR) | 一 | √ | √ | √ | √ | √ |
| 衰减串音比功率和(PSACR) | — | √ | √ | √ | √ | √ |
| 等电平远端串音(ELFEXT) | — | √ | √ | √ | √ |  |
| 等电平远端串音功率和(PSELFEXT) | — | √ | √ | √ | √ | √ |
| 直流环路电阻 | √ | √ | √ | √ | √ | √ |
| 传播时延 | √ | √ | √ | √ | √ | √ |
| 传播时延偏差 | √ | √ |  | √ | √ | √ |

注：“√”表示对应等级的布线系统应包括的检测项目；“一”表示对应等级的布线系统不包括的检测项目。

8.2.2电缆布线系统检测除应符合本标准第8.2.1条规定外，尚应符合下列规定：

1综合布线工程应对每一个完工后的信息点进行永久链路测试；主干缆线采用电缆时也可按照永久链路的连接模型进行测试。

2对包含设备缆线和跳线在内的拟用或在用电缆链路进行质量认证时可按信道方式测试。

3现场条件允许时，宜对EA级、FA级电缆布线系统的外部近端串音功率和(PSANEXT)及外部远端衰减串音比功率和(PSAACR-F)指标进行抽检。

4屏蔽布线系统应考虑屏蔽层的导通性能；屏蔽布线系统用于工业级以太网和数据中心时，应排除虚接地的情况。

5当电缆布线系统应用于工业以太网、POE及高速信道等场景时，还可检测TCL、ELTCTL、不平衡电阻、耦合衰减等屏蔽特性指标。

8.2.3电缆布线系统的主干缆线应全数检测；水平缆线应按照不小于10%的比例抽检，数量不足5条应全数检测。

8.3光纤布线系统性能

8.3.1光纤布线系统应检测光纤信道或链路的衰减和长度。

8.3.2光纤链路各检测项目的性能指标检测结果应符合现行国家标准《综合布线系统工程验收规范》GB/T50312的有关规定。

8.3.3光纤布线系统的主干光纤应全数检测；水平光纤应按照不小于10%的比例抽检，数量不足5条应全数检测。

8.4布线管理系统

8.4.1布线管理系统检测应包括下列内容：

1系统管理软件版本及软件功能；

2管理区域平面显示；

3硬件设施及关键元件位置及工作状态显示；

4硬件设施管理；

5布线的标签和标识。

8.4.2综合布线系统的标签和标识应按不低于10%的比例抽检，综合布线系统管理功能应全数检测。检测结果应符合设计要求。

8.5检测设备

8.5.1电缆及光纤布线系统测试仪表的精度应符合表8.5.1的规定并应向下兼容。

表8.5.1测试仪表精度

| 布线等级 | D级 | E级 | EA级 | F级 | FA级 |
| --- | --- | --- | --- | --- | --- |
| 仪表精度 | Ie | Ⅲ | Ⅲe | IV | V |

8.5.2测试仪表应有输出接口，应能将存储的检测数据输出至计算机。检测原始数据应用专用软件打开，且不应被篡改。

8.6检测方法

8.6.1当C级和D级布线系统采用大对数对绞电缆时，应按照信道方式进行测试。各等级的布线系统应按照永久链路或信道方式进行测试。检测应按图8.6.1进行连接，并应符合下列规定：


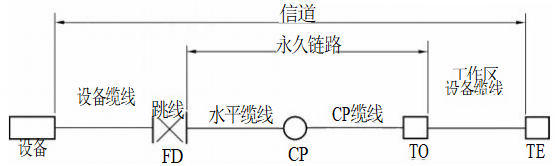


图8.6.1永久链路和布线系统信道

FD一楼层配线设备；TO一信息插模块；CP—集合点；TE—终端设备

1永久链路应由长度不大于90m的水平缆线及最多3个连接器件组成。

2布线系统信道应由长度不大于90m的水平缆线、10m的

跳线和设备缆线及最多4个连接器件组成。

3对于屏蔽布线系统，应依据图8.6.1所示模型检测系统屏蔽层的连续性及链路屏蔽线屏蔽层与两端接地的电位差，电位差值应小于1Vr.m.s。

4对于开放式办公室综合布线系统，应允许开放办公室空间频繁重组而不破坏原来的水平布线路由，在配线架与信息插座之间加入集合点或多用户中间插座。对于集合方式，其集合点的位置距配线间应大于15m,对于多用户中间插座方式，从中间插座至设备终端的工作区电缆的最大长度不宜大于20m。对于这类系统进行检测时应选用“信道检测模型”。

5双绞线电缆主干布线系统应依据图8.6.1所示模型进行检测。

8.6.2电缆布线系统永久链路和信道方式各检测项目的性能指标检测结果应符合现行国家标准《综合布线系统工程验收规范》GB/T50312的有关规定。对绞电缆两端的连接器件也可为配线架模块。

8.6.3光纤链路检测应符合下列规定：

1应根据被测光纤规格及接口类型，选择对应的光纤检测跳线；光跳线及其连接件的衰减值应加入光纤测试链路设置基准；

2多模光纤应进行850nm及1300nm波长的检测，单模光纤应进行1310nm及1550nm波长的检测；

3在两端对光纤进行双向(收与发)检测，连接模型(图8.6.3)应由光功率计、光源、光纤跳线以及被测的链路

组成。


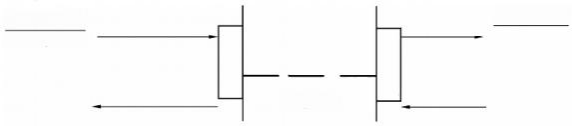
光纤检测仪

光纤检测仪

光纤布线

工作区光纤插座光纤插座

图8.6.3光纤布线检测模型

8.6.4光纤到用户单元系统工程光纤链路检测应符合下列规定：

1检测连接方式如图8.6.4所示；


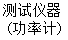

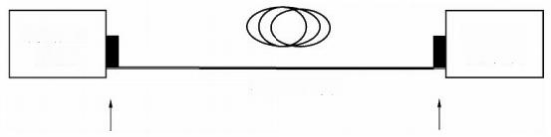
测试仪器

(光源)

被测光纤链路

图8.6.4光纤链路衰减检测连接方式

2工程检测中应对上述光纤链路只采用1310nm波长进行衰减指标检测；

3当OM3/OM4光纤应用于10Gbit/s及以上链路时，应使用发射和接收补偿光纤进行双向OTDR测试。

8.6.5布线管理系统检测应符合下列规定：

1在管理计算机上打开软件，查看系统管理软件版本；

2检测所有硬件设备及其楼层平面图的显示功能；

3检测干线子系统和配线子系统的元件位置和工作状态的显示功能；

4对于使用电子配线架的系统，插拔配线架端口跳线，进行跳接试验，验证链路数据库更新情况，并检测硬件设施工作状态的显示功能；

5标签标识应位置明显、固定牢靠、内容清晰并满足唯一性要求。

9移动通信室内信号覆盖系统

9.0.1智能建筑移动通信室内信号覆盖系统工程质量检测内容应包括设备安装场地和环境相关项目。

9.0.2移动通信室内信号覆盖系统检测范围应根据设计要求确定。

9.0.3检测项目应符合本标准第20章的有关规定，并应符合设计要求。

9.0.4检测设备、检测数量、检测方法及合格判定方法等应符合本标准第20章的有关规定。

10卫星通信系统

10.0.1智能建筑卫星通信系统工程质量检测内容应包括设备安装场地和环境相关项目。

10.0.2卫星通信系统检测范围应根据设计要求确定。

10.0.3检测项目应符合本标准第20章的有关规定，并应符合设计要求。

10.0.4检测设备、检测数量、检测方法及合格判定方法等应符合本标准第20章的有关规定。

11有线电视及卫星电视接收系统

11.1一般规定

11.1.1智能建筑有线电视及卫星电视接收系统工程质量检测内容应包括数字信号有线电视系统、模拟信号有线电视系统、卫星电视接收系统。

11.1.2有线电视及卫星电视接收系统检测应采用参数检测和主观评价方式。

11.1.3检测环境及条件除应符合本标准第3.2.5条规定外，尚应符合下列规定：

1系统应处于正常运行状态，前端应具备电视信号输出条件；

2系统工作环境应满足设计要求；

3系统设备供电电压应满足设计要求。

11.1.4检测点、数量及合格判断标准应符合下列规定：

1当系统的输出端口数量小于1000时，测试点不得少于2个；当系统的输出端口数量大于等于1000时，每1000点应选取2个~3个测试点；

2对于基于HFC或同轴电缆传输的双向数字电视系统，主观评价的测试点数应符合本条第1款规定，客观测试点的数量不应少于系统输出端口数量的5%,且不应少于20个；

3测试点应至少有一个位于系统中主干线的最后一个分配放大器之后的点；

4检测结果符合设计要求为合格，被检项目的合格率应为100%。

11.2数字信号有线电视系统

11.2.1数字信号有线电视系统主观评价应包括下列内容：

1图像质量；

2声音质量；

3唇音同步；

4节目频道切换；

5字幕。

11.2.2双向数字电视系统下行指标性能检测应包括下列内容：

1数字频道输出口电平；

2频道间电平差；

3调制误码率；

4实际误码率；

5数字射频信号与噪声功率比；

6载波复合二次差拍比；

7载波复合三次差拍比；

8交扰调制比；

9载波交流声比；

10色/亮度时延差；

11回波值；

12微分增益；

13微分相位；

14频率稳定度；

15系统输出口相互隔离度；

16特性阻抗；

17相邻频道间隔；

18辐射与干扰。

11.2.3双向数字电视系统上行指标性能检测应包括下列内容：

1上行通道频率范围；

2上行端口输入电平；

3上行传输路由增益差；

4上行通道频率响应；

5上行最大过载电平；

6上行通道传输延时；

7上行通道群延时；

8载波/汇集噪声；

9信号交流声调制比；

10回波值；

11通道串扰抑制比；

12特性阻抗。

11.3模拟信号有线电视系统

11.3.1模拟信号有线电视系统主观评价应包括下列内容：

1系统载噪比；

2载波互调比；

3交扰调制比；

4回波值；

5色/亮度时延差；

6载波交流声；

7伴音和调频广播的声音。

11.3.2模拟信号有线电视系统应检测终端输出电平。

11.4卫星电视接收系统

11.4.1卫星电视接收系统应检测接收频段。

11.4.2卫星电视接收系统的视频系统指标检测应包括下列内容：

1幅频特性；

2K因子；

3亮度非线性失真；

4微分增益失真；

5微分相位失真；

6色度-亮度增益差；

7色度-亮度时延差；

8S/N加权值；

9行同步前沿抖动。

11.4.3卫星电视接收系统的音频系统指标检测应包括下列内容：

1音频幅频特性；

2总谐波失真；

3音频信噪比；

4左右声道串扰；

5左右声道电平差；

6左右声道相位差。

11.5检测设备

11.5.1电视信号场强仪应符合下列规定：

1频率范围应为48MHz~1000MHz;

2频率最大允许误差应为±10kHz;

3电平范围应为30dBμV~120dBμV;

4电平最大允许误差应为±3dB;

53dB带宽范围应为250kHz～350kHz;

6电压驻波比不应大于3.0(75Ω)。

11.5.2频谱分析仪应符合下列规定：

1频率范围应为150kHz~1GHz;

2分辨率带宽范围应为1Hz~5MHz。

11.6检测方法

11.6.1数字信号有线电视系统主观评价应符合下列规定。

1图像质量的主观评价应符合下列规定：

1)图像质量主观评价评分分级应符合表11.6.1的规定；

表11.6.1图像质量主观评价评分分级

| 图像质量主观评价 | 评分分级 |
| --- | --- |
| 质量极佳，十分满意 | 5分(优) |
| 质量好，比较满意 | 4分(良) |
| 质量一般，尚可接受 | 3分(中) |
| 质量差，勉强能看 | 2分(差) |
| 质量低劣，无法看清 | 1分(劣) |

2)评价人员数量不宜少于5人，各评价人员应独立评分，并应取算术平均值为评价结果；

3)评价项目的得分值不低于4分的应判定为合格。

2声音质量应音质无明显失真，对白清晰，不应出现明显的噪声和杂音。

3唇音同步应无明显的图像滞后或超前于声音的现象。

4节目频道切换检测应符合下列规定：

1)应使用遥控器进行节目频道切换，使用带有时间和帧数显示的摄像机拍摄记录切换动作和屏幕变化，计算节目切换所需时间，节目频道切换时不能出现严重的马赛克或长时间黑屏，节目切换时间平均等待时间应小于2.5s,最大不应超过3.5s;

2)以上测量至少进行3次，其中应包括有位于不同频点的节目频道切换。

5图像显示字幕应清晰、稳定。

11.6.2双向数字电视系统下行指标性能检测应按现行性行业标准《有线电视广播系统技术规范》GY/T106和《有线数字电视系统技术要求和测量方法》GY/T221的相关规定执行。

11.6.3双向数字电视系统上行指标性能检测应按现行行业标准《HFC网络上行传输物理通道技术规范》GY/T180的相关规定执行。

11.6.4模拟信号有线电视系统检测应符合下列规定：

1模拟信号有线电视系统检测可采用主观评价方法，评价标准应符合表11.6.4的规定；

表11.6.4模拟信号有线电视系统评价标准

| 序号 | 项目名称 | 评价标准 |
| --- | --- | --- |
| 1 | 系统载噪比 | 无噪波，即无“雪花干扰” |
| 2 | 载波互调比 | 图像中无垂直、倾斜或水平条纹 |
| 3 | 交扰调制比 | 图像中无移动、垂直或斜图案，即无"窜台" |
| 4 | 回波值 | 图像中无沿水平方向分布在右边  一条或多条轮廓线，即无“重影” |
| 5 | 色/亮度时延差 | 图像中色、亮信息对齐，即“无彩色鬼影” |
| 6 | 载波交流声 | 图像中无上下移动的水平条纹，即无“滚道”现象 |
| 7 | 伴音和调频广播的声音 | 无背景噪声，如咝咝声、哼声、蜂鸣声和串音等 |

2图像质量评价应按本标准表11.6.1进行评价。

11.6.5模拟信号有线电视系统终端输出电平值检测应符合下列规定：

1应连接电视信号场强仪至有线终端输出口；

2应调节场强仪的测量频率到被测频道的载频位置；

3完成设定应在稳定30s后，读出电平值并记录。

11.6.6卫星接收电视系统的接收频段检测方法应按现行行业标准《卫星数字电视接收站测量方法——系统测量》GY/T149的规定执行。

11.6.7卫星接收电视系统的视频系统检测方法应按现行行业标准《卫星数字电视接收站测量方法——系统测量》GY/T149的规定执行。

11.6.8卫星接收电视系统的音频系统检测方法应按现行行业标准《卫星数字电视接收站测量方法——系统测量》GY/T149的规定执行。

12公共广播系统

12.1一般规定

12.1.1智能建筑公共广播系统工程质量检测内容应包括系统功能和电声性能检测。

12.1.2检测环境及条件除应符合本标准第3.2.5条规定外，尚应符合下列规定：

1测量时，相关分区的广播扬声器应全部开启；

2测量点现场的信噪比不应小于15dB。

12.1.3应按广播分区进行检测，检测结果符合设计要求为合格，被检项目的合格率应为100%。

12.2系统功能

12.2.1公共广播系统功能检测应包括业务广播功能、背景广播功能和紧急广播功能。

12.2.2业务广播功能应符合表12.2.2的规定。

表12.2.2业务广播功能

| 级别 | 应具备的功能 |
| --- | --- |
| 一级 | 编程管理，自动定时运行(允许手动干预);矩阵分区；强制插入语声信  号；广播优先级排序；主/备功率放大器自动切换；支持寻呼台站；支持远程监控 |
| 二级 | 自动定时运行(允许手动干预);分区管理；可强制插入语声信号；功率放大器故障告警 |
| 三级 | — |

12.2.3背景广播功能应符合表12.2.3的规定。

表12.2.3背景广播功能

| 级别 | 应具备的功能 |
| --- | --- |
| 一级 | 编程管理，自动定时运行(允许手动干预);具备音量调节环节；矩阵分区；强制插入语声信号；广播优先级排序；支持远程监控 |
| 二级 | 自动定时运行(允许手动干预);具备音量调节环节；分区管理；可强制插入语声信号 |
| 三级 | — |

12.2.4紧急广播功能应符合下列规定：

1当公共广播系统有多种用途时，紧急广播应具有最高级别的优先权；公共广播系统应能在手动或警报信号触发的10s内，向相关广播区播放警示信号(含警笛)、警报语音文件或实时指挥语音；

2以现场环境噪声为基准，紧急广播的信噪比不应小于12dB;

3紧急广播系统设备应处于热备用状态，或具有定时自检和故障自动告警功能；

4紧急广播系统应具有应急备用电源，主电源与备用电源切换时间不应大于1s;应急备用电源应能满足20min以上的紧急广播；当以电池为备用电源时，系统应设置电池自动充电装置；

5紧急广播音量应能自动调节至不小于应备声压级界定的音量；

6当需手动发布紧急广播时，应设置一键到位功能；

7单台广播功率放大器失效不应导致广播系统整体失效；

8单个广播扬声器失效不应导致广播分区失效；

9紧急广播系统的其他应备功能尚应符合表12.2.4的规定。

表12.2.4紧急广播功能

| 级别 | 应具备的功能 |
| --- | --- |
| 一级 | 具有与事故处理系统联动的接口；与事故处理系统相容的强制插入语声信号；主/备电源自动切换；主/备功率放大器自动切换；支持有广播优先级排序的寻呼台站；支持远程监控；支持备份主机；自动生成运行记录 |
| 二级 | 与事故处理系统相容的强制插入语声信号；主/备功率放大器自动切换 |
| 三级 | 可强制插入紧急广播和警笛；功率放大器故障告警 |

12.2.5系统功能应全部检测，检测结果符合设计要求为合格，被检项目的合格率应为100%。

12.3电声性能

12.3.1电声性能检测应包括下列内容：

1应备声压级；

2声场不均匀度(室内);

3漏出声衰减；

4系统设备信噪比；

5扩声系统语言传输指数；

6传输频率特性。

12.3.2公共广播系统在各广播分区内的电声性能指标应符合现行国家标准《公共广播系统工程技术规范》GB50526的有关规定。

12.4检测设备

12.4.1声级计不应低于现行国家标准《电声学声级计第1部分：规范》GB/T3785.1中规定的2级声级计，且应具有1/3倍频程频谱分析功能。

12.4.2STIPA信号发生器在消声室内测得测试声源本身的语言传输指数值不应小于0.97。

12.4.3粉红噪声信号发生器在广播系统输入的宽带粉红噪声电

信号，其电平应能达到一般设备标称的额定输入电平。

12.4.4计时器的准确度应为±0.1s。

12.5检测方法

12.5.1业务广播的功能检测应符合下列规定：

1检测公共广播系统编程定时管理功能，应设定系统定时运行时间，使用秒表记录定时误差，该误差不应大于10s;

2检测公共广播系统广播分区功能，应调整分区，在相关广播服务区查看调整后分区状态；

3使用高级别优先级信号对广播分区强制插入语声信号，强插应有效；

4应设置广播信号优先级排序，播放不同优先级别的语音信号，验证广播优先级设置的有效性；

5当模拟主功率放大器故障时，验证系统应能产生故障报警，同时备用功率放大器应能自动切换投入并且正常工作；

6公共广播系统应配置有寻呼台站；

7公共广播系统应具有系统状态远程监控功能。

12.5.2背景广播的功能检测应符合下列规定：

1检测公共广播系统编程定时管理功能，应设定指定时间发布语声广播信号，使用秒表记录时间误差；

2应调节公共广播系统的音量控制系统，验证其功能；

3检测公共广播系统分区广播功能，应调整分区，在相关广播服务区查看调整后分区状态；

4使用高级别优先级信号对广播分区强制插入语声信号，强插应有效；

5应设置广播信号优先级排序，播放不同优先级别的语音信号，验证广播优先级设置的有效性；

6应利用远程控制台登录公共广播系统，检测公共广播系统远程监控功能的有效性。

12.5.3紧急广播的功能检测应符合下列规定：

1当公共广播系统有多种用途时，查看紧急广播应具有最高级别优先权。手动触发紧急广播警报功能，使用秒表检测，在10s内相关广播分区应能够正常播放警示信号(含警笛)、警报语声文件或实时指挥语声；

2对广播分区环境噪声进行检测，记录环境噪声，开启广播分区紧急广播警示信号(含警笛)、警报语声文件或实时指挥语声，平均声压级不应小于环境噪声12dB;

3可模拟功率放大器或扬声器故障状态，检测系统故障自动报警功能；

4可模拟电源切换状态，记录电源切换时间，备用电源应能够满足紧急广播系统20min以上的运行要求，且备用电源应具备自动充电功能；

5开启紧急广播功能后，紧急广播音量应能够自动调节至接近系统应备声压级界定的音量；

6公共广播系统应提供手动发布紧急广播所需的一键到位功能，检测时可使用一键到位功能发出模拟语声信息；

7当模拟单台广播功率放大器故障(断电)时，整个广播系统应可以继续运行；

8可取下广播分区内随机选定的扬声器，模拟扬声器故障，所在广播分区系统应可以继续运行；

9检测公共广播系统与消防中心联动的接口，可模拟消防警报，在与消防分区相容的紧急广播分区内应能强制插入语声信号；

10可模拟主功率放大器故障，检查备用功率放大器应能自动切换并检查系统运行状态；

11应检测公共广播系统所有寻呼台站的优先级设定；

12公共广播系统应具有系统状态远程监控功能；

13紧急广播系统应配置有备份主机，系统自动报警功能应能生成运行记录。

12.5.4测量电声性能时，测量点选择应符合下列规定：

1测量点距地面高度应为1.2m～1.5m,与墙体的距离应大于1.5m。

2测量点应有代表性，应处于广播服务区内公众经常活动的地方，并宜在被测广播服务区内均匀分布，但应避免选在广播扬声器附近且在其声辐射轴线上的地点。

3当公共广播服务区为室内时，每50m²应至少有一个测量点，且测量点总数不宜少于3个。

4当公共广播服务区为广场时，每20m×20m应至少有一个测量点，且测量点总数不宜少于3个。

5当室内和广场的空间结构以及广播扬声器的布局为轴对称时，可只在中线以及一侧选取测量点。

6当公共广播服务区为走廊、通道时，应在走廊行走方向的中轴线上选取测量点。在走廊、通道的中点附近和所有端点、拐角附近均应设测量点。两测量点的距离不大于5m时可合并；当走廊、通道的直线长度大于80m时，应每隔20m～30m追加一个测量点；当走廊、通道内广播扬声器的布局相同时，追加的测量点可不超过5个。

12.5.5应备声压级检测应符合下列规定：

1公共广播系统服务区内的每一个厅堂或每一个房间应分别测量；

2在公共广播系统设备的线路输入端口，输入宽带粉红噪声信号(图12.5.5),其电平应等于设备标称的额定输入电平；


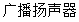

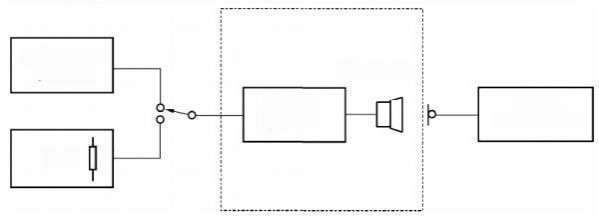
粉红噪声信号发生器


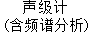
公共广播系统设备

600Ω

图12.5.5公共广播系统电声性能测试示意

3应调节公共广播系统增益使系统达到额定输出功率，并应在广播服务区内选定测量点，分别测量各点的宽带稳态有效值声压级；

4各测量点稳态有效声压级的平均值应为被测广播服务区的应备声压级，并应按下式计算：


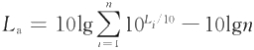
 (12.5.5)式中：L——各测量点稳态有效值声压级的平均值(dB);

L;———测量点i的宽带稳态有效值声压级(dB);

n——测量点数(个)。

5测得广播服务区应备声压级应符合本标准第12.3.2条的规定。

12.5.6声场不均匀度(室内)检测应符合下列规定：

1公共广播系统服务区内的每一个厅堂或每一个房间应分别测量；

2应按本标准第12.5.5条第2款的规定输入信号；

3应调节公共广播系统增益，并使广播服务区内测量点的声压级达到本标准第12.1.2条第2款的规定；

4应在服务区内选定的测量点测量各点的宽带稳态有效值声压级；

5各测量点之间宽带稳态有效值声压级的最大值和最小值之差应为广播服务区声场不均匀度；

6测得广播服务区声场不均匀度应符合本标准第12.3.2条的规定。

12.5.7漏出声衰减检测应符合下列规定：

1应在被测公共广播服务区边界外30m处；东南西北方位应各选一个最靠近广播扬声器或处于广播扬声器辐射轴线方向上的测量点；

2应按本标准第12.5.5条测得系统的应备声压级；

3应按本标准第12.5.5条第2款的规定输入测量信号，然后调节公共广播系统增益使系统达到额定输出功率，并在规定的测量点上，测量宽带稳态有效值声压级，并取其中最大值；

4广播服务区漏出声衰减应按下式计算：

LI=L。-Lm(12.5.7)

式中：L——漏出声衰减(dB);

La——被测公共广播系统的应备声压级(dB);

Lm——按本章规定测得的稳态有效值声压级的最大值(dB)。

5测得广播服务区漏出声衰减应符合本标准第12.3.2条的规定。

12.5.8系统设备信噪比检测应符合下列规定：

1系统设备信噪比应以广播分区为单位，分别进行测量；

2应按本标准第12.5.5条第2款的规定输入测量信号；

3应调节广播系统增益，使系统达到额定输出功率，并在广播区内任一个广播扬声器的输入端，测量广播扬声器输入信号的电平；

4应按本标准图12.5.5原理，采用600Ω电阻置换公共广播系统设备输入端的粉红噪声信号发生器，在同一个广播扬声器的输入端，测量该广播区的本底噪声电平(A计权);

5广播扬声器输入信号电平与本底噪声电平的差值应为系统设备信噪比；

6测得系统设备信噪比应符合本标准第12.3.2条的规定。

12.5.9扩声系统语言传输指数检测应符合下列规定：

1扩声系统语言传输指数测量点的选择应符合本标准第12.5.4条的规定；

2室外广播服务区应以广播分区为单位，分别进行测量，室内广播服务区每一个厅堂和每一个房间应分别测量；

3在距广播传声器0.5m处，配置STIPA测试声源(图12.5.9);


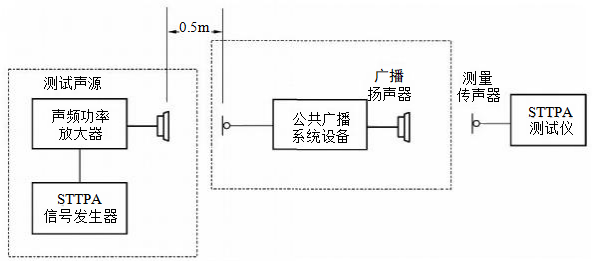


图12.5.9扩声系统语言传输指数测量示意

4测试声源应输出扩声系统语言传输指数测试信号，调节测试声源的输出，应使广播传声器输入的稳态有效值声压级等于80dB;

5应调节公共广播系统增益，并使测量现场信噪比等于或大于15dB;

6每一个测量点应测量3次，并应取其算数平均值作为该点的扩声系统语言传输指数值；

7每一个被测广播服务区中应有2/3及以上的测量点的扩声系统语言传输指数值符合本标准第12.3.2条的规定。

12.5.10传输频率特性检测应符合下列规定：

1公共广播系统服务区内的每一个厅堂或每一个房间应分别测量；

2应按本标准第12.5.5条第2款的规定输入测量信号；

3应调节公共广播系统增益，使广播服务区内测量点达到本标准第12.1.2条第2款的规定；

4应采用具有1/3倍频程频谱分析功能的2级声级计，在广播服务区内选定的测量点测量其传输频率特性曲线；

5应以测得的传输频率特性曲线上的最大声压级为0dB,该曲线的幅度变化不超过相应等级规定的容差域范围时，判定该测量点合格，并符合本标准第12.3.2条的规定；

6当被测广播服务区的传输频率特性测量点有2/3及以上合格时，可判定该被测广播服务区的传输频率特性符合规定。

13会议系统

13.1一般规定

13.1.1智能建筑会议系统工程质量检测内容应包括会议扩声系统、会议视频显示系统、会议灯光系统、会议电视系统和其他系统。

13.1.2系统检测应以声学特性指标和显示特性指标检测为主，声音质量和图像质量主观评价为辅。

13.1.3检测环境及条件除应符合本标准第3.2.5条规定外，尚应符合下列规定：

1会议系统检测前，宜检查会议系统引入电源和会场建声的检测记录；

2会议系统检测应根据设计要求确定检测内容。

13.1.4系统应全数检测，检测结果全部符合设计要求为合格。

13.2会议扩声系统

13.2.1会议扩声系统应检测语言传输指数，或直接检测下列声学特性指标：

1最大声压级；

2传输频率特性；

3传声增益；

4声场不均匀度；

5系统总噪声级。

13.2.2会议扩声系统应进行声音质量主观评价。

13.3会议视频显示系统

13.3.1会议视频显示系统显示特性指标检测应包括下列内容：

1显示屏亮度；

2图像对比度；

3亮度均匀性；

4图像水平清晰度；

5色域覆盖率；

6水平视角、垂直视角。

13.3.2会议视频显示系统应进行图像质量主观评价。

13.4会议灯光系统

13.4.1会议灯光系统光源性能检测包括下列内容：

1应检测平均照度；

2宜检测色温；

3宜检测显色指数。

13.4.2会议灯光系统光源控制功能检测应包括下列内容：

1分区控制功能；

2调光功能。

13.5会议电视系统

13.5.1会议电视系统应进行会议电视功能检测。

13.5.2会议电视系统性能检测应包括下列内容：

1声音延时；

2声像同步；

3会议电视回声；

4图像清晰度；

5图像连续性。

13.6其他系统

13.6.1会议同声传译系统应检测系统功能、性能以及与火灾自动报警系统的联动功能。

13.6.2会议讨论系统应检测会议讨论功能和与火灾自动报警系

统的联动功能。

13.6.3会议签到系统应检测签到功能、签到的准确性和报表功能。

13.6.4会议表决系统应检测表决功能，应包括表决速度和准确性。

13.6.5会议集中控制系统应检测对各子系统的控制功能。

13.6.6会议录播系统检测应包括下列内容：

1现场音视频录播功能；

2计算机数字信号的处理和录播功能；

3信号处理和录播的质量。

13.6.7会议摄像系统的检测应包括下列内容：

1摄像功能；

2摄像机的自动跟踪功能；

3摄像机的预置位调用功能。

13.7检测设备

13.7.1噪声信号发生器应符合下列规定：

1粉红噪声和白噪声信号的峰值因数不应小于2;

2粉红噪声频谱密度测量范围应为20Hz～20kHz,

±1.5dB(衰减器输出);20Hz～20kHz,±2dB(负载输出);2Hz～200kHz,±1.5dB(衰减器输出);

3白噪声频谱密度测量范围应为20Hz～20kHz,±1dB

(衰减器输出);20Hz～20kHz,±1.5dB(负载输出);2Hz~200kHz,±1dB(衰减器输出);

4衰减输出电压应为0.4mV~4V,准确度应为±1dB;

5信噪比不应低于60dB。

13.7.2限幅器应符合下列规定：

1频率特性测量范围应为20Hz～20kHz,±0.3dB(参考频率1kHz);

2总谐波失真不应大于0.5%;

3输入电平应为0dB(0.775V);

4输出电平应为0dB(0.775V);

5限幅范围应为-20dB~+6dB。

13.7.3测试功率放大器应符合下列规定：

1频率范围应满足20Hz~20kHz,不均匀度应为±0.5dB;

2总谐波失真不应大于0.5%;

3额定功率不应小于200W(RMS值);

4负载阻抗范围应为4Ω、8Ω、16Ω。

13.7.4测试传声器应符合现行行业标准《电声学测量电容传声器通用规范》SJ/T10724的有关规定。

13.7.5测量放大器应符合下列规定：

1频率响应范围应为20Hz～20kHz,准确度应为±0.5dB;

2测量范围应为100μV~300V;

3频率计权应为A,C;

4时间计权应为F(快挡),S(慢挡);

5固有噪声不应大于10μV;

6极化电压应为200V;

7检波器特性应包括有效值(应为±0.5dB,峰值因数应为5)、平均值、峰值；

8衰减器准确度应为±0.1dB。

13.7.61/3倍频程带通滤波器应符合现行国家标准《电声学倍频程和分数倍频程滤波器》GB/T3241的有关规定。

13.7.7声级记录仪应符合下列规定：

1频率响应范围应为20Hz～20kHz,±1dB;

2动态范围应为25dB、50dB对数；

3分辨力应为0.25dB(50dB量程电位计);

4整流响应应包括有效值、平均值、峰值；

5描划速度应至少具有100mm/s和250mm/s两挡。

13.7.8声级计不应低于现行国家标准《电声学声级计第1部分：规范》GB/T3785.1中规定的2级声级计。

13.7.9模拟节目信号应符合现行国家标准《声系统设备概述模拟节目信号》GB/T6278的规定。

13.7.10声频信号发生器应符合下列规定：

1频率特性应为20Hz～20kHz,±0.3dB(参考频率1kHz);

2频率示值准确度应为示值的1%±1Hz;

3频率稳定度应符合预热半小时后，1kHz频率点每小时漂移小于或等于6Hz;

4总谐波失真应符合衰减输出小于或等于0.5%,功率输出小于或等于1.0%;

5电压表准确度应为2.5%;

6衰减器准确度应为0.2dB;

7信噪比不应小于65dB;

8最大压缩量不应小于60dB(输入压缩电压不大于1V)。

13.7.11视频测试信号发生器应符合下列规定：

1应能产生现行国家标准《视频显示系统工程测量规范》GB/T50525中规定的测试信号；

2测试信号形式应为视频显示系统所采用的YPbPr色差分量信号、Y/C分量信号、复合视频信号、VGA信号、数字音视频信号或网络传输接口信号。

13.7.12亮度计应符合下列规定：

1当测量电视型视频显示系统时，测量范围应为0.2cd/m²~12000cd/m²;

2当测量LED、投影型视频显示系统时，测量范围应为2cd/m²~12000cd/m²。

13.7.13色度计应能在亮度低于2cd/m²时，测量屏幕上小面积色度坐标(u',v)。

13.7.14照度计不应低于一级。

13.7.15光谱辐射计应符合下列规定：

1波长范围应为380nm～780nm,测光重复性应在1%

以内；

2波长示值绝对误差应为±2.0nm;

3光谱带宽不应大于8nm;

4光谱测量间隔不应大于5nm;

5对A光源的色品坐标测量误差应为：|△x|≤0.0015,|△y|≤0.0015。

13.7.16示波器应符合下列规定：

1应具备长余辉功能；

2应具备幅度为0.02V~40V的正负脉冲波形；

3带宽不应小于100MHz。

13.8检测方法

13.8.1会议扩声系统语言传输指数和其他声学特性指标的检测方法应符合现行国家标准《会议电视会场系统工程施工及验收规范》GB50793的相关规定。

13.8.2会议扩声系统声音质量主观评价应符合下列规定：

1声音质量主观评价评分应符合表13.8.2的规定；

表13.8.2声音质量主观评价评分

| 声音质量主观评价 | 评分值(等级) |
| --- | --- |
| 质量极佳，十分满意 | 5分(优) |
| 质量好，比较满意 | 4分(良) |
| 质量一般，尚可接受 | 3分(中) |
| 质量差，勉强能听 | 2分(差) |
| 质量低劣，无法忍受 | 1分(劣) |

2评价人员不应少于5名；

3评价人员应对本会场音频扩声系统、远程会场音频播放系统独立评价打分，并应取算术平均值为评价结果。所有评价人员对本会场音频扩声系统、远程会场音频播放系统的评价得分的算术平均值不应小于4分。

13.8.3会议视频显示特性指标的检测方法应符合现行国家标准《视频显示系统工程测量规范》GB/T50525的相关规定。

13.8.4会议视频显示系统图像质量主观评价应符合本标准第11.6.1条第1款的规定。

13.8.5会议灯光系统光源性能的平均照度值、色温和显色指数的检测方法应符合现行国家标准《照明测量方法》GB/T5700

的相关规定。会议电视灯光平均照度值应符合表13.8.5的规定。光源的色温应为3200K、4000K或5600K,且所有光源的色温应一致。光源的显色指数R。不应小于85。

表13.8.5会议电视灯光平均照度值

| 照明区域 | 垂直照度(lx) | 参考平面 | 水平照度(lx) | 参考平面 |
| --- | --- | --- | --- | --- |
| 主席台座席区 | ≥400 | 1.40m垂直面 | ≥600 | 0.75m水平面 |
| 听众摄像区 | ≥300 | 1.40m垂直面 | ≥500 | 0.75m水平面 |

13.8.6会议灯光系统控制功能检测应符合下列规定：

1现场对不同分区进行灯光开关动作，检测灯光控制功能应符合设计要求；

2现场进行调光动作，检测灯光调光功能应符合设计要求。

13.8.7会议电视系统的功能检测应采用现场演示的方法，按设计要求对功能进行逐项检测。

13.8.8会议电视系统的性能检测应符合下列规定：

1声音延时的客观检测方法应符合现行国家标准《会议电视会场系统工程施工及验收规范》GB50793中“会议电视声音延时和声像同步测量方法”的规定；声音延时的主观评价可在两地会场由专人进行1至10交叉报数，用计时器记录时长，扣除不经电路传输进行交叉报数所需时间，除以10即得平均延时值；

2声像同步的客观检测方法应符合现行国家标准《会议电视会场系统工程施工及验收规范》GB50793中“会议电视声音延时和声像同步测量方法”的规定；可由远程会场专人拍手掌，本会场进行声像同步的主观评价；

3会议电视回声的检测可在两地会场由男声讲话和报数，两会场进行有无明显回声的主观评价；

4图像清晰度的检测可观看远程会场摄像机前静止特写画面，评价其静止图像的清晰度；

5图像连续性的检测可观看远程会场摄像机前人员快速走动画面，评价其活动图像的连续性。

13.8.9会议同声传译系统功能和性能检测方法应符合现行国家标准《红外线同声传译系统工程技术规范》GB50524的相关规定；会议同声传译系统与火灾自动报警系统联动功能的检测可现场模拟火灾发生，对联动功能进行逐项检测。

13.8.10会议讨论系统的其他功能采用现场演示的方法逐项检测；会议讨论系统与火灾自动报警系统联动功能的检测可现场模拟火灾发生，对联动功能进行逐项检测。

13.8.11会议签到系统的签到功能、签到的准确性和报表功能应采用现场演示的方法逐项检测。

13.8.12会议表决系统应采用现场演示的方法逐项检测；应采用计时器检测表决速度，同时检测表决结果应准确无误。

13.8.13会议集中控制系统功能应采用现场演示的方法逐项检测。

13.8.14会议录播系统音视频录播功能、计算机数字信号处理及录播功能应采用现场演示的方法逐项检测；信号处理和录播系统的质量检测，可人为对比录播前后的图像和声音质量，采用主观评价的方法进行。

13.8.15会议摄像系统检测应符合下列规定：

1摄像功能应采用现场演示的方法逐项检测；

2自动跟踪功能应根据设计要求触发跟踪条件，摄像机能够正确完成跟踪动作；

3现场模拟会议讨论，摄像机应能自动指向发言人位置。

14信息导引及发布系统

14.1一般规定

14.1.1智能建筑信息导引及发布系统工程质量检测内容应包括信息播控设备、信息发布系统软件、信息显示屏和终端设备等。

14.1.2系统检测应以系统功能和显示性能检测为主，图像质量主观评价为辅。

14.1.3检测环境及条件除应符合本标准第3.2.5条规定外，尚应符合下列规定：

1显示性能应在显示屏正常工作状态下工作30min后，再进行检测；

2室内显示屏的测量应在环境光照度(200±50)lx下进行；

3室外显示屏的测量应在环境光照度(10000±1000)lx下进行。

14.1.4系统应全数检测，检测结果全部符合设计要求为合格。

14.2信息播控设备

14.2.1信息播控设备的功能检测应包括下列内容：

1终端监控；

2格式支持；

3紧急插播；

4电源管理；

5应急处理；

6分屏显示；

7断电后来电启动。

14.2.2信息播控设备硬件性能检测应包括下列内容：

1应支持各种通用和开放的通信接口；

2CPU、内存、硬盘、视频接口应满足设计要求；

3输出应支持多种方式。

14.3信息发布系统软件

14.3.1信息发布系统软件功能检测应包括下列内容：

1任务管理；

2任务设定；

3节目管理；

4字幕管理；

5发送管理；

6终端管理；

7电源管理；

8终端监控；

9系统维护；

10远程控制。

14.3.2信息发布系统软件性能检测可包括下列内容：

1误操作提示；

2重要操作警告和确认提示；

3容错处理；

4可靠性；

5数据保护；

6数据加密；

7日志保存；

8权限合理性；

9可扩展性。

14.4信息显示屏

14.4.1信息显示屏的显示性能应包括光学性能、电性能和结构性能。其中，光学性能检测内容应根据LED显示屏、投影型显示屏和电视型显示屏等不同类型确定。

14.4.2LED显示屏光学性能的检测应包括下列内容：

1最大亮度；

2通断比；

3亮度均匀性；

4色度不均匀性；

5视角；

6换帧频率；

7刷新频率；

8像素失控率。

14.4.3投影型、电视型显示屏光学性能的检测应包括下列内容：

1亮度；

2对比度；

3亮度均匀性；

4色度不均匀性；

5视角；

6色域覆盖率。

14.4.4显示屏电性能的检测应包括下列内容：

1清晰度；

2亮度信噪比；

3调幅；

4调相色度信噪比；

5视频输出电平；

6亮度幅频响应；

7灰度等级；

8显示图像信噪比；

9图像拼缝等内容。

14.4.5显示屏结构性能的检测应包括下列内容：

1平整度；

2拼缝；

3图像拼接误差。

14.4.6信息显示屏应进行图像质量主观评价。

14.5终端设备

14.5.1信息导引及发布系统的终端设备应包括信息导引设施、控制终端和查询终端。

14.5.2信息导引及发布系统应检测信息导引设施的指示功能，以及系统终端设备的远程控制和查询功能。

14.6检测设备

14.6.1视频测试信号发生器应符合本标准第13.7.11条的规定。

14.6.2亮度计应符合本标准第13.7.12条的规定。

14.6.3色度计应符合本标准第13.7.13条的规定。

14.6.4示波器带宽不应小于100MHz。

14.6.5视频分析仪应能自动测量复合视频输出电平、亮度信噪比、色度信噪比和亮度通道带宽。

14.6.6摄像机信噪比应大于显示图像的信噪比。

14.7检测方法

14.7.1信息播控设备功能检测应符合下列规定：

1终端监控功能检测，应能够通过系统后台选择要监控的终端，系统后台的显示内容应与终端显示内容一致；

2格式支持功能检测，可通过后台管理工作站发送不同格式的媒体文件等，系统应能够正常显示；

3紧急插播功能检测，可通过后台管理工作站插播不同类型的文件，如图片、视频、紧急字幕等到指定显示屏，系统应无异常；

4电源管理功能应能够根据规则分别或分组定义指定时间和周期的开机或者关机，电源管理功能应可以远程启动及关闭终端；

5应急处理功能检测，可手动断开网络，终端能够正常播放已定节目表内容，如没有内容应能够自动播放默认素材；

6分屏显示功能应能将屏幕划分多个区域，区域大小比例应可调整，各区域显示内容应可支持不同格式的素材；

7手动将信息播控设备断电再次通电，信息播控设备应能自动启动。

14.7.2信息播控设备性能检测应符合下列规定：

1查看各设备通信接口支持情况，应满足设计要求；

2查看现场设备的硬件配置参数，应满足设计要求；

3查看信息播控设备支持的视频播出格式，应满足设计要求。

14.7.3信息发布系统软件功能检测应符合下列规定：

1任务管理功能检测，可进入系统创建播放任务表，系统应支持自定义间隔时间或固定间隔时间、可创建多个播放任务、播放任务可以跟指定的终端进行关联；

2任务设定功能检测，可进入系统根据任务时间表设定不同时间段需要播放的节目，软件应能够对不同的时间段进行节目的设定，并应能够在指定时间段进行播放；

3节目管理功能检测，可进入系统新建节目表，应能够选择节目内容、设定顺序；

4字幕管理功能检测，系统应能够对字幕内容、字体、颜色、滚动速度、边框、背景、显示位置、滚动速度等参数进行设定；

5发送管理功能检测，系统应能够向终端发送任务表和节目表、字幕表，并应支持插播内容的发送，中断插播后，原有的播放计划不应受影响；

6终端管理功能检测，应能够进行终端设备添加、分组和任务关联管理等操作，操作结果应正确；

7电源管理功能检测，系统应支持根据规则分别或分组定

义指定时间和周期的开机或者关机，电源管理功能应可以远程启动及关闭终端；

8终端监控功能检测，系统应支持根据终端地址查看终端设备播放内容；

9系统维护功能检测，系统应能够实现用户管理、系统备份、备份数据保存、系统恢复等功能；

10远程控制功能检测，可进入系统进行终端远程启停、远程调节参数等操作，终端端实际动作应与系统控制相一致。

14.7.4信息发布系统软件性能检测应符合下列规定：

1误操作提示检测，可进入系统人为进行错误操作，系统应有提示信息，提示信息应明确、显著;

2重要操作警告和确认提示检测，可进入系统进行重要操作，系统应有警告信息，警告信息应明确、显著;

3容错处理检测，可进入系统输入错误信息或命令，系统应能够识别错误并有提示信息；

4可靠性检测，可进入系统进行反复随机操作，系统不应出现错误动作；

5数据保护检测，可进入系统进行数据保存操作，系统应能够将数据保存到指定位置，并赋予指定属性；

6数据加密检测，可进入系统确认用户密码和密钥，应以密文方式存储；

7日志保存检测，系统应有日志记录保存功能，日志信息应包含用户登录、退出、关键操作等重要的系统事件；

8权限合理性检测，各用户权限应合理；使用不同权限用户验证应能够按照权限的限定进行操作；

9可扩展性检测，软件应能够正常安装卸载，应能够添加删除组件。

14.7.5LED显示屏光学性能的检测方法应符合现行国家标准《视频显示系统工程测量规范》GB/T50525中的相关规定。

14.7.6投影型、电视型显示屏光学性能的检测方法应符合现行国家标准《视频显示系统工程测量规范》GB/T50525中的相关规定。

14.7.7显示屏电性能的检测方法应符合现行国家标准《视频显示系统工程测量规范》GB/T50525中的相关规定。

14.7.8显示屏结构性能的检测方法应符合现行国家标准《视频显示系统工程测量规范》GB/T50525中的相关规定。

14.7.9图像质量主观评价应符合本标准第11.6.1条第1款的规定。

14.7.10信息导引设施的指示功能以及系统终端设备的远程控制和查询功能可根据设计要求采用现场模拟的方法逐条功能进行检测。

15时钟系统

15.1一般规定

15.1.1智能建筑时钟系统工程质量检测内容应包括标准时间源、石英谐振器母钟和子钟、时钟监控系统。

15.1.2时钟系统检测应以系统授时校准功能和系统显示的准确性检测为主，可靠性检测为辅。

15.1.3系统设备应全数检测。检测结果符合设计要求为合格，被检项目的合格率应为100%。

15.2标准时间源

15.2.1标准时间源功能检测应包括下列内容：

1接收和发送标准时间信号功能；

2自动恢复功能。

15.2.2备用标准时间源应检测标准时间源和备用标准时间源之间的时间信号切换功能。

15.3石英谐振器母钟和子钟

15.3.1母钟和子钟功能检测应包括下列内容：

1授时校准功能；

2自动恢复功能；

3换历功能。

15.3.2母钟和子钟走时性能检测应包括下列内容：

1平均瞬时日差；

2显示同步性；

3使用可靠性。

15.4时钟监控系统

15.4.1时钟监控系统应检测实时监控功能。

15.4.2时钟监控系统的故障反馈功能检测应包括下列内容：

1故障告警功能；

2日志存储、打印功能。

15.5检测设备

15.5.1频率计分辨力应为1×10⁻⁹s。

15.5.2示波器分辨力应为1×10-⁶s,带宽应为100MHz。

15.5.3计时器准确度应为±0.01s。

15.6检测方法

15.6.1标准时间源功能检测应符合下列规定：

1标准时间源的时间显示应与母钟的时间显示一致；

2人为切断标准时间源电源，恢复其供电后，标准时间源应能恢复正常的接收标准时间信号和授时功能；

3人为切断标准时间源电源并更改母钟时间，备用标准时间源应能对母钟进行授时校准。

15.6.2母钟和子钟功能检测应符合下列规定：

1人为设置标准时间源与母钟的时间显示偏差，启动母钟上授时校准功能，母钟上的显示时间应能调整为标准时间源的显示时间；人为设置子钟与母钟的时间显示偏差，启动自动校时功能，子钟上显示时间应调整为母钟的显示时间；

2人为切断时钟系统电源，恢复其供电后母钟和子钟应自动恢复为标准时间；

3人为将时间调至“23:55:00”时刻，在时钟运行到“00:00:00”时，母钟和子钟应能正常换历。

15.6.3母钟走时性能检测应符合下列规定：

1使用频率计检测母钟平均瞬时日差，一级母钟不应大于


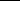
0.01s/d,二级母钟不应大于0.1s/d;

2使用示波器测量标准时间信号输出波形与母钟信号输出波形，母钟的输出口同步偏差不应大于50ms;

3观察母钟走时情况，母钟在正常运行72h期间不应停走，累计误差不应大于1min。

15.6.4子钟走时性能检测应符合下列规定：

1应使用频率计检测子钟平均瞬时日差，子钟的平均瞬时日差不应大于1s/d;

2应使用秒表检测子钟与母钟的时间显示偏差，显示偏差不应大于1s;

3在母钟正常运行72h期间，子钟的使用应可靠。

15.6.5在实时监控系统上查看系统运行状态，系统监控状态应与实际运行状态一致。

15.6.6系统故障反馈功能检测应符合下列规定：

1人为制造系统故障，系统监控终端的故障反馈应符合设计要求；

2人为制造系统故障，系统日志的记录和打印应符合设计要求。

16信息化应用系统

16.1一般规定

16.1.1智能建筑信息化应用系统工程质量检测内容应包括信息化应用系统的硬件设备和应用软件。

16.1.2信息化应用系统可包括公共服务系统、智能卡应用系统、物业管理系统、信息设施运行管理系统、信息安全管理系统、通用业务和专业业务系统等，系统检测应先检测硬件设备，后检测应用软件。

16.1.3系统应全数检测，检测结果全部符合设计要求为合格。

16.2硬件设备

16.2.1应查看服务器、工作站、系统专用设备等硬件设备性能指标，可包括中央处理器核心数量与频率、内存容量与频率、外存容量、外部接口带宽与速率等。

16.2.2能够安装软件的硬件设备应查看设备中安装运行的软件产品，不应有与业务应用无关的软件存在。

16.3应用软件

16.3.1应用软件的检测应包括业务功能和业务流程。

16.3.2应用软件的功能检测宜包括下列内容：

1重要数据删除的警告和确认提示；

2输入非法值的处理；

3密钥存储方式；

4对用户操作进行记录并保存的功能；

5各种权限用户的分配；

6数据备份和恢复功能；

7用户界面采用的语言；

8提示信息。

16.3.3应用软件的性能检测宜包括下列内容：

1响应时间；

2可扩展性。

16.4检测设备

16.4.1计时器的准确度应为±0.1s。

16.4.2用于网络性能检测的设备应符合本标准第7.4节的规定。

16.5检测方法

16.5.1硬件设备性能指标的检测应符合下列规定：

1硬件设备的实际性能指标应符合设计要求；

2应手动设置各外部接口参数，检测接口功能。

16.5.2应登录设备查看设备中安装的软件列表，软件列表中不应含有与业务应用无关的软件。

16.5.3应用软件的业务功能和业务流程的检测，应根据测试大纲中的测试内容和测试用例逐项操作，操作结果应满足设计要求。

16.5.4应用软件的功能检测应符合下列规定：

1重要数据删除的警告和确认提示功能，可人为进行重要数据删除操作，软件应有警告和提示；

2输入非法值的处理功能，可人为输入非法值，软件应有相应处理功能；

3密钥存储方式，可在软件运行环境中检查，应符合软件需求规格说明书的规定；

4对用户操作进行记录并保存的功能，可检查软件的日志功能，软件应开启日志功能且日志功能应包括用户操作；

5各种权限用户的分配功能，可检查软件的权限管理功能，

确认权限分配及权限控制功能有效；

6数据备份和恢复功能，应包含备份和恢复功能选项，可现场进行备份及恢复操作并确认功能正常；

7用户界面采用的语言，可进入软件各操作界面进行查看；

8提示信息功能，可按照软件需求规格说明书中的规定，逐项触发提示信息，确认提示信息的显示时机及内容应正确。

16.5.5应用软件的性能检测应符合下列规定：

1响应时间检测，可在各软件功能的检查过程中同步进行，可人为触发软件动作，并使用计时器记录从动作触发信号发出开始到系统接受信号响应动作为止的时间；

2可扩展性检测，可对软件需求规格说明书中相关功能的说明进行检查，应符合设计要求；可包括但不限于软件组件的添加删除功能、软件的安装卸载功能等。

17建筑设备管理系统

17.1一般规定

17.1.1智能建筑工程建筑设备管理系统工程质量检测内容应包括暖通空调监控系统、供配电监测系统、公共照明监控系统、给水排水监控系统、电梯和自动扶梯监测系统、能效监管系统，以及中央管理工作站和系统的实时性、可靠性、可维护性及评测项目。

17.1.2系统检测应在建筑给水排水及供暖、通风与空调、建筑电气及电梯等分部工程验收合格后进行。

17.2暖通空调监控系统

17.2.1暖通空调监控系统检测应包括制冷机组、冷水系统及冷却水系统、热源及热交换系统、新风系统、定风量空调系统及变风量空调系统等系统监控功能检测。

17.2.2制冷机组监控功能检测应包括下列内容：

1各类监控参数；

2制冷机启停控制、顺序控制、设备联动控制功能。

17.2.3冷水系统监控功能检测应包括下列内容：

1各类监控参数；

2冷水系统设备启停控制、顺序控制、设备联动控制功能；

3冷水旁通阀压差控制；

4冷水泵过载报警。

17.2.4冷却水系统监控功能检测应包括下列内容：

1系统监控参数；

2冷却水系统设备启停控制、顺序控制、设备联动控制功能；

3冷却塔风机台数或冷却塔风机速度控制；

4冷却水泵、冷却塔风机过载报警。

17.2.5热源系统监控功能检测应包括下列内容：

1热源系统各类监控参数；

2热源系统燃烧系统自动调节；

3锅炉、水泵等设备顺序启停控制；

4锅炉房可燃气体、有害物质浓度检测报警；

5烟道温度超限报警及蒸气压力超限报警；

6设备故障报警及安全保护功能；

7燃料消耗量统计记录。

17.2.6热交换系统监控功能检测应包括下列内容：

1系统各类监控参数；

2系统负荷自动调节功能；

3系统设备顺序启停控制功能；

4管网超压报警、循环泵故障报警及安全保护功能；

5能量消耗统计记录。

17.2.7新风系统监控功能检测应包括下列内容：

1送风温度控制；

2送风相对湿度控制；

3预定时间表自动启停功能；

4过滤网压差检测功能；

5防冻保护功能；

6电气联锁控制；

7报警功能。

17.2.8定风量空调系统监控功能检测应包括下列内容：

1回风温度(室内温度)控制；

2回风相对湿度(房间相对湿度)控制；

3预定时间表自动启/停功能；

4新风阀、排风阀、回风阀比例控制功能；

5过滤网压差检测功能；

6电气联锁控制；

7防冻保护功能；

8报警功能。

17.2.9变风量空调系统监控功能检测应包括下列内容：

1送风温度控制；

2回风相对湿度控制；

3送风量控制(包括静压法、压差法、总风量法等);

4回风量控制；

5新风量控制；

6室内(或使用区域)温度控制；

7预定时间表自动启/停功能；

8过滤网压差检测功能；

9联锁控制功能；

10防冻保护功能；

11报警功能。

17.2.10冷热源机组应全部检测，新风、空调机组每类按总数的20%抽检，且不得少于5台，每类机组不足5台时全部检测。检测结果全部符合设计要求为合格。

17.3供配电监测系统

17.3.1供配电监测系统检测应包括下列内容：

1高低压开关运行状况及故障报警；

2电源进线及主供电回路电流、电压、功率因数和电能计量等；

3电力变压器温度测量及超温报警；

4发电机组供电电流、电压、频率及储油罐液位监视；

5不间断电源装置、蓄电池组、充电设备工作及切换状态。

17.3.2供配电监测系统功能应全部检测，电气参数检测数量应按每类参数的20%抽检，且数量不得小于20点，数量小于20点时全部检测。检测结果全部符合设计要求为合格。

17.4公共照明监控系统

17.4.1公共照明监控系统检测应包括下列内容：

1照明设施及回路按分区与时间开、关控制功能；

2照明设施或回路按室外照度、室内有人与否进行开、关或照度控制功能；

3中央工作站对照明设施或回路的运行状态监视、用电量及用电费用统计等管理功能；

4当市电停电或有突发事件发生时，相应照明回路的联动配合功能；

5公共照明手动开关功能。

17.4.2检测数量应按照明回路总数的10%抽检，数量不得小于10路，总数小于10时应全部检测。检测结果全部符合设计要求为合格。

17.5给水排水监控系统

17.5.1给水排水监控系统检测应包括高位水箱给水系统、变频器恒压给水系统和排水监控系统检测。

17.5.2高位水箱给水监控系统检测应包括下列内容：

1依据液位测量，检测给水泵启/停控制的正确性；

2备用水泵的切换功能；

3水泵运行状态监测；

4高低液位报警、水泵过载报警与保护；

5设备运行时间累计及维护报告提示功能、各泵运行时间均衡功能。

17.5.3变频器恒压给水监控系统检测应包括下列内容：

1供水的恒压控制功能；

2水泵切换功能；

3水泵运行状态监测；

4超压报警、设备故障报警；5设备运行时间累计及维护报告提示功能、各泵运行时间均衡功能。

17.5.4排水监控系统检测应包括下列内容：

1依据污水池液位，检测排水泵启/停控制的正确性；

2备用水泵切换功能；

3水泵运行状态监测；

4污水池高低液位报警、水泵过载报警与保护；

5设备运行时间累计及维护报告提示功能；各泵运行时间均衡功能。

17.5.5给水监控系统应全部检测；排水监控系统应按50%抽检，且不得小于5套，总数小于5套时全部检测。检测结果全部符合设计要求为合格。

17.6电梯和自动扶梯监测系统

17.6.1电梯和自动扶梯监测系统检测应包括下列内容：

1电梯和自动扶梯运行状态的监测，包括电梯的启/停、上下行、位置等运行状态和故障状态，自动扶梯的运行状态和故障状态；

2在中央工作站以图形方式显示电梯与自动扶梯的运行状态信息及故障报警功能；

3电梯与自动扶梯运行和维护档案。

17.6.2系统功能应全部检测，检测结果全部符合设计要求为合格。

17.7能效监管系统

17.7.1能效监管系统检测应包括下列内容：

1水、电、气、热及冷量等能耗数据的显示、记录、统计和汇总，并形成分类分级报表；

2根据统计和汇总数据进行分析，能够发现问题、找出原因，可提供审计、公示及高能耗预警。

17.7.2能效监管系统对能耗数据的显示、记录、统计、汇总及分析等功能应全部检测，检测结果符合设计要求为合格。

17.8中央管理工作站

17.8.1中央管理工作站检测应包括下列内容：

1中央管理工作站的监控和管理功能；

2中央管理工作站显示和记录功能的实时性和准确性，以及对设备进行控制和管理功能的有效性和正确性；

3通过数据接口通信的子系统及机电设备，数据传输及控制的正确性和实时性，应符合设计要求；

4中央管理工作站数据的存储和统计功能包括检测数据、运行数据、历史数据趋势图显示、报警存储统计(包括各类参数报警、通信报警和设备报警)情况；中央管理工作站的历史数据存储时间应大于3个月；

5中央管理工作站数据报表生成及打印功能，报警信息的打印功能；

6中央管理工作站操作的方便性，人机界面应符合友好、汉化、图形化要求，图形切换流程清楚易懂，便于操作。报警信息的显示和处理应直观有效；

7操作权限，确保系统操作的安全性。

17.8.2中央管理工作站功能应全部检测，检测结果符合设计要求为合格。

17.9系统实时性、可靠性、可维护性及评测项目

17.9.1系统实时性检测应包括下列内容：

1系统控制命令响应时间；

2系统报警信号响应时间。

17.9.2系统可靠性检测应符合下列规定：

1网络故障时，现场控制器(DDC)应能保持正常工作；

2现场操作大型设备的启动/停止时，不应影响系统正常工

作，不应出现数据采集和传输错误，不应产生异常动作等；

3系统电网电源切换或UPS电源转换时系统运行不应中断；

4冗余主机自动投入时，系统运行不应中断。

17.9.3系统可维护性检测应包括下列内容：

1应用软件的在线编程(组态)、参数修改、下载功能；

2设备、网络通信故障的自检功能。

17.9.4评测项目宜包括下列内容：

1控制网络和数据库的标准化、开放性；

2系统的冗余配置，主要指控制网络、工作站、服务器、数据库和电源等；

3系统可扩展性，包括控制器I/O口备用量、机柜卡件安装空间、备用接线端等；

4节能措施评测。

17.9.5系统实时性、可靠性、可维护性及评测项目的检测数量及合格判定应符合下列规定：

1系统实时性应按控制器数量的10%抽检，且不应少于10台，少于10台时全部检测，检测结果全部符合设计要求为合格；

2系统可靠性中设备启停应按设备数量10%抽检，且不应少于10台，少于10台时全部检测；电源切换、UPS切换和主机切换应全数检测，检测结果全部符合设计要求为合格；

3系统可维护性检测结果符合设计要求为合格；

4测评项目检测结果符合设计要求为合格。

17.10检测设备

17.10.1温度测试仪的准确度应为±0.5℃。

17.10.2湿度测试仪的准确度应为±3%RH。

17.10.3风速计测量范围应为0m/s～30m/s,准确度应为±5%,读数应为±3字。

17.10.4压差计测量范围应为0kPa~±1kPa,准确度应为

±1%(满量程)。

17.10.5电能质量分析仪电压测量范围应为0V~500V,准确度应为±0.1V,频率准确度应为±0.15Hz,失真度准确度应为±3%~±5%。

17.10.6超声波流量计测量范围应为0m/s~±32m/s,准确度应为±1%。

17.10.7计时器的准确度应为±0.1s。

17.10.8照度计测量范围应为0lx~2500lx,准确度应为±3%,读数应为±5字。

17.11检测方法

17.11.1暖通空调监控系统的检测应符合下列规定：

1应通过工作站或现场控制器改变参数设定，检测制冷机、冷冻和冷却水系统的自动控制功能、预定时间表功能等，核实冷冻冷却水系统能耗计量与统计资料。

2应通过工作站或现场控制器改变参数设定，检测热源和热交换系统的自动控制功能、预定时间表功能等，核实热源和热交换系统能耗计量与统计资料。

3用便携式或其他类型的温湿度仪器在现场检测温度和相对湿度，与在中央工作站或现场控制器显示的温度和相对湿度值进行比对，其数据应一致。应检测风压开关、防冻开关工作状态；检测风机及相应冷/热水调节阀工作状态；检测风阀开关状态。

4应在中央工作站或现场控制器改变温度设定值，记录温度控制过程，检测控制效果和系统稳定性，应与系统运行历史记录一致。

5应在中央工作站或现场控制器改变相对湿度设定值，进行相对湿度调节，观察运行工况的稳定性、系统响应时间及控制效果，应与系统运行历史记录一致。

6应在中央工作站改变预定时间表设定，检测空调系统自

动启停功能。

7变风量空调系统送风量控制(静压法、压差法、总风量法)检测，应改变设定值，使之大于或小于测量值，变频风机转速应随之升高或降低，测量值应逐步趋于设定值。

8新风量控制检测，应通过改变新风量(或风速、空气质量)设定值，与新风量(或风速、空气质量)测量值比较，进行新风量调节。

9应启动/关闭新风空调系统、定风量空调系统、变风量空调系统，检查各设备的联锁控制功能。

10防冻保护功能检测可采用改变防冻开关动作设定值的方法，模拟进行。

11应人为设置故障，在中央工作站检测系统故障报警功能，包括过滤器压差开关报警、风机故障报警、送风温度传感器故障报警及处理。

17.11.2供配电监测系统的检测应符合下列规定：

1应利用中央工作站读取数据与现场使用仪器仪表测量的数据进行比较；

2应人工模拟变配电故障，监测中央工作站显示的设备实时状态及报警状态，并应进行现场核实。

17.11.3公共照明监控系统的检测应符合下列规定：

1应依据施工图设计文件，按照明回路分组，在中央工作站上设定回路的开与关，观察相应照明回路动作情况；

2应启动时间表，改变时间控制程序，观察相应照明回路动作情况；

3当照明回路采用光感或红外等方式触发时，以相应方式触发开/关，观察相应照明回路动作情况。

17.11.4给水排水监控系统检测应符合下列规定：

1应通过工作站参数设置或人为改变现场测控点状态，检测设备的启/停控制、运行状态、水泵转速的自动调节和水泵切换等功能；

2应人为设置故障，验证报警和保护措施；

3应查看工作站历史记录，核实设备运行时间。

17.11.5电梯和自动扶梯监控系统的检测应符合下列规定：

1应在中央工作站核实监测运行状态的正确性和准确性；

2应在现场模拟故障，在工作站进行故障报警、记录与打印功能检测；

3应核实电梯与自动扶梯运行统计资料。

17.11.6能效监管系统的检测应符合下列规定：

1应在中央工作站监测智能机电设备或子系统的运行参数，包括工作状态参数和报警信息，并与实际状态核实；

2应对数据进行统计和汇总并做趋势分析，找出其中的能耗高点。

17.11.7中央管理工作站检测应符合下列规定：

1应在中央管理工作站查看和操作各项功能；

2应与现场运行状态进行核实。

17.11.8系统实时性检测应符合下列规定：

1应在中央管理工作站操作启停设备、修改控制参数等；

2应在现场模拟设备报警；

3应在中央管理工作站检测并记录响应时间。

17.11.9系统可靠性检测应符合下列规定：

1应远程或手动启/停设备，观察中央站数据显示和系统及设备工作情况；

2切断系统电网电源，转为UPS供电时，应观察系统运行情况；

3中央站冗余主机切换投运，应观察系统运行情况。

17.11.10系统可维护性检测应符合下列规定：

1应在中央站或现场进行控制器或控制模块应用软件的在线编程(组态)、参数修改及下载，进行验证；

2应在现场设置设备故障和网络故障，在中央站观察结果显示和报警，指示的设备故障的名称和位置应一致。

17.11.11评测项目检测方法应符合下列规定：

1应根据工程实际情况对网络、数据库、系统冗余或系统扩展性等内容进行评价；

2应通过对各子系统的检测，对节能优化控制功能作出评价。应根据合同技术文件的要求，结合对能耗数据记录分析、现场控制效果测试和数据计算后作出能满足设计要求的评价。

18安全技术防范系统

18.1一般规定

18.1.1智能建筑安全技术防范系统的工程质量检测内容应包括入侵报警系统、视频安防监控系统、出入口控制系统、电子巡查系统、停车库(场)管理系统和安全防范综合管理系统等。

18.1.2智能建筑安全技术防范系统检测应包括系统功能和系统性能检测。

18.1.3检测环境及条件除应符合本标准第3.2.5条规定外，尚应符合下列规定：

1属于国家强制性认证的安全防范产品应检查产品的认证证书或检测报告；

2应先子系统检测、后安全防范综合管理系统检测。

18.1.4安全防范系统的检测数量和合格判定应符合下列规定：

1系统功能应全数检测；

2系统前端设备应按不低于总数的20%抽检，且不应少于3台，不足3台应全数检测；抽检应包括传输最远距离和安装环境最恶劣处设备；

3检测结果全部符合设计要求为合格，被检项目的合格率应为100%;

4全部子系统检测均合格的，系统检测应判定为合格。

18.1.5数据处理应符合下列规定：

1对于图像水平清晰度和灰度等级测试时应报出最大值；

2照度和响应时间的测试次数不应少于3次，并应计算平均值；

3其他测量数值应以一次性测量报出数值为准。

18.2入侵报警系统

18.2.1入侵报警系统的功能检测应包括下列内容：

1入侵报警功能检测应包括下列内容：

1)各类入侵探测器报警功能；

2)紧急报警功能；

3)多路同时报警功能；

4)报警后恢复功能。

2防破坏及故障报警功能检测应包括下列内容：

1)入侵探测器防拆报警功能；

2)报警控制主机防拆报警功能；

3)报警控制主机信号线防破坏报警功能；

4)入侵探测器电源线防破坏报警功能；

5)报警控制主机主备电源故障报警功能；

6)公共网络传输防破坏报警功能。

3记录和显示功能检测应包括下列内容：

1)信息显示功能；

2)信息记录功能：

3)管理功能。

4系统自检功能检测应包括下列内容：

1)自检功能；

2)周界和防区设定功能；

3)设防/撤防、旁路功能。

5报警复核功能。

6报警优先级功能。

18.2.2入侵报警系统的性能检测应包括下列内容：

1探测器灵敏度；

2入侵报警响应时间；

3故障报警响应时间；

4报警声压级。

18.3视频安防监控系统

18.3.1视频安防监控系统的功能检测应包括下列内容：

1系统控制功能检测应包括下列内容：

1)编程功能；

2)遥控功能。

2监视功能。

3显示功能。

4记录功能。

5回放功能。

6报警联动功能。

7图像丢失报警功能。

8视频监控联网系统信息传输、交换、控制功能。

18.3.2视频安防监控系统的性能检测应包括下列内容：

1模拟摄像机视频信号输出电压峰值；

2监视图像的水平清晰度和灰度；

3视频分辨率；

4监视区域照度；

5前端监控设备到直连平台之间，采用有线网传输的监控系统的信息时延；

6系统的图像存储时间。

18.4出入口控制系统

18.4.1系统功能检测应包括下列内容：

1出入目标识读装置功能；

2信息处理/控制设备功能；

3执行机构功能；

4报警功能；

5访客(可视)对讲系统功能。

18.4.2系统性能检测应包括下列内容：

1识别器的识别速度；

2非接触式识别器的识别距离；

3访客对讲系统振铃声压级。

18.5电子巡查系统

18.5.1系统的巡查设置功能检测应包括下列内容：

1在线式系统巡查设置功能；

2离线式系统巡查设置功能。

18.5.2系统的管理功能检测应包括下列内容：

1密码管理功能；

2记录打印功能。

18.6停车库(场)管理系统

18.6.1停车库(场)出入口的功能检测应包括下列内容：

1识别功能；

2控制功能；

3报警功能；

4出票验票功能。

18.6.2停车库(场)综合管理功能检测应包括下列内容：

1计费管理功能；

2显示功能；

3报表及其打印功能；

4车辆(车位)引导功能。

18.7安全防范综合管理系统

18.7.1安全防范综合管理系统功能检测应包括下列内容：

1防范设施的设置功能；

2图像质量及各类信息记录保存时间；

3系统时钟同步功能。

18.7.2安全防范综合管理系统对子系统的管理功能检测应包括

下列内容：

1各子系统间联动；

2各子系统对监控中心控制命令响应的准确性和实时性；

3监控中心对各子系统工作状态显示、报警信息的准确性和实时性。

18.8检测设备

18.8.1清晰度测试卡的水平清晰度不应低于1100TVL。

18.8.2灰度测试卡的灰度等级不应低于9级。

18.8.3计时器的准确度应为±0.1s。

18.8.4声级计不应低于现行国家标准《电声学声级计第1部分：规范》GB/T3785.1中规定的2级声级计。

18.8.5示波器的频带宽度不应小于20MHz,输入灵敏度不应小于5mVpp/cm,扫描精度应能达到微秒级。

18.8.6图形工作站的硬件应能满足图形处理要求，并可采集多种输入信号，显示分辨率不应低于1920×1080像素。

18.9检测方法

18.9.1入侵报警系统的功能检测应符合下列规定：

1入侵报警功能检测应符合下列规定：

1)在设防状态下，在被测入侵探测器的控制区域人为模拟入侵发生，从报警控制端检测探测器的报警信息、报警区域，应能发出声、光报警信号；应无漏报，防区内应无盲区，并应能手动将报警状态复位；

2)紧急报警装置应设置为不可撤防状态，从报警控制端应能依次显示报警发生区域，并发出声、光报警，然后将报警状态手动复位，报警信号应无丢失；检测紧急报警装置应有防误触发措施，触发后应自锁；

3)同时触发多路探测器报警，从报警控制端应能依次显示报警发生区域，并发出声、光报警，然后将报警状

态手动复位，报警信号应无丢失；

4)人为触发报警，然后将报警系统手动复位；系统设为布防状态，检测探测器应能正常工作；系统设为撤防，检测探测器不应发出报警信号。

2防破坏及故障报警功能检测应符合下列规定：

1)人为拆除探测器机壳，检测报警控制端的报警地址显示，声、光报警信号，并能保持到手动复位，报警信号不应丢失；

2)人为拆除报警控制主机机壳，检测报警控制端的声、光报警信号，并能保持到手动复位，报警信号不应丢失；

3)人为模拟报警信号传输线开路、短路或并接负载情况，检测报警控制端的声、光报警信号以及线路故障信息显示，并应能保持到手动复位，报警信号不应丢失；

4)人为模拟探测器电源线断开情况，检测报警控制端的声、光报警信号以及线路故障信息显示，并能保持到手动复位，报警信号不应丢失；

5)人为断开报警控制主机主电源，检测备用电源能否自动工作，并在控制端显示主电源故障信息；人为断开备用电源，控制端应能显示备用电源故障信息；报警控制端的声、光报警信号和电源故障信息显示应能保持到手动复位，报警信号不应丢失；

6)在有线公共网络传输系统中，切断传输线路开始计时；在无线网络传输系统中，将信号干扰器放置在干扰信号能覆盖报警主机的范围内，开启信号干扰器电源开关，开始计时；从计时开始至监控中心或手机客户端发出故障报警为计时结束，采用秒表进行记录，该时间应满足系统设计要求。

3记录、显示功能检测应符合下列规定：

1)应检测系统显示的开机时间、关机时间、报警、故障、

被破坏、设防时间、撤防时间、更改时间等信息功能；

2)应检测系统记录的报警发生时间、地点、报警信息性质、故障信息性质等，信息记录不应更改；

3)应检测系统自动显示和记录功能，应具备多级管理密码。

4系统自检功能检测应符合下列规定：

1)检测系统的自检或巡检功能，人为设置探测器或报警控制设备故障或破坏时，应有声、光报警信号，并能保持到手动复位；

2)检测系统的防区设定功能，可按防区报警延时时间分为瞬时防区或延时防区，也可按入侵探测器的安装位置及其防范功能不同分为内部防区、出入防区、周界防区、日夜防区、24小时防区、火警防区等；

3)检测系统的手动/自动设防/撤防功能，应能在任意时间和任意区域分别实现；设防/撤防状态应有显示，并有明显区别。

5人为触发探测器报警，系统应能对现场报警信息进行声音或图像复核；

6检测经市话网电话传输报警信息，主叫方式下应能报警优先。

18.9.2入侵报警系统的性能检测应符合下列规定：

1探测器灵敏度检测应符合下列规定：

1)被动红外、微波、超声及双鉴探测器。采用步行测试方法进行现场测试，作为参考目标的双臂交叉在胸前，在设计探测范围边界上分别以0.3m/s、1m/s、3m/s

三种速度移动，在3m或最大探测距离30%以内(两者取其小值),沿着入侵的方向移动，探测器应产生报警状态。本试验应在设计最大探测范围内至少选3点进行。

2)主动红外探测器，用一直径200mm圆柱形物体，其

长度应能充分遮断光束，以大于10m/s的速度垂直于射束轴线方向通过射束，探测器不应产生报警，当物体以小于5m/s的速度通过射束时，探测器应立即产生报警状态。本试验应在设计最大探测范围内至少选3点进行。

3)磁开关探测器，逐渐打开装有磁开关入侵探测器的门、窗，开启门隙最大为60mm,磁开关入侵探测器应产生报警状态。本试验应以不同速度进行，至少重复3次。

2系统入侵报警响应时间检测采用人为触发报警探测器的方法。从探测器产生报警信息，到入侵报警控制器发出声光报警信号，响应时间应符合下列规定：

1)分线制、总线制、无线制和基于局域网、电力网、广电网的入侵报警系统不应大于2s;

2)基于市话网电话线的入侵报警系统不应大于20s。

3系统故障报警响应时间检测采用人为制造系统故障的方法。从出现故障到报警控制器发出报警信息的响应时间，应符合设计要求。

4用声级计在距离报警发生器正前方1m处检测报警声音的声压级，不应小于80dB。

18.9.3视频安防监控系统的功能检测应符合下列规定。

1系统控制功能检测应符合下列规定：

1)应通过控制设备键盘检测手动或自动编程功能；

2)应通过控制设备对云台、镜头、防护罩等前端设备进行控制，检测其平稳性和准确性。

2检测监视区域应符合设计要求，重要部位的监视设置情况，监视应实时并无盲区；监视图像信息和声音信息应具有原始完整性。

3系统显示功能检测应符合下列规定：

1)查看单画面或多画面的显示图像，应清晰、稳定；

2)查看监视画面上应显示日期、时间及前端设备编号或

地址码；

3)应检测画面定格、切换显示、多路报警显示、任意设定视频警戒区域等功能；

4)应采用5级评分法对图像质量进行主观评价，评分分级应符合本标准第11.6.1条相关规定。

4系统记录功能检测应符合下列规定：

1)查看图像记录文件，记录图像应清晰稳定，显示方式应满足安全管理要求；

2)查看图像画面上应有记录日期、时间及前端摄像机的编号或地址码；

3)人为停电或关机，检测系统应能自动存储编程设置、摄像机编号、时间、地址等信息；恢复供电或开机，检测系统应能自动进人正常工作状态。

5系统回放功能检测应符合下列规定：

1)查看回放图像，图像应清晰稳定，显示方式应满足安全管理要求；

2)查看回放图像画面上应有日期、时间及前端摄像机的编号或地址码，文字显示应采用简体中文；

3)查看报警联动回放图像，回放图像应为报警现场摄像机的覆盖范围，报警现场应能完整再现；

4)比较回放图像与监视图像，画面质量应无明显劣化，移动目标的图像回放效果应符合设计要求。

6报警联动功能检测应符合下列规定：

1)人为触发入侵报警，检测联动装置应能将相应摄像机自动开启，并将报警现场画面显示在监视画面上，监视画面上应能显示摄像机的地址及时间，报警画面应能单画面记录，其联动响应时间不应大于4s;

2)其他系统联动功能检测应符合本款第1项方法。

7人为切断视频输入信号，检测系统应能发出报警信息。

8用网络分析仪及符合现行国家标准的测试软件对视频监

控联网系统信息传输、交换、控制功能进行测试，应分别满足《公共安全视频监控联网系统信息传输、交换、控制技术要求》GB/T28181中的相关规定。

18.9.4视频安防监控系统的性能检测应符合下列规定：

1用示波器检测模拟摄像机视频信号输出电压峰值，峰值的绝对值应为1Vpp±3dB之间。

2用清晰度测试卡和灰度测试卡检测监视图像的水平清晰度和灰度等级，结果应符合设计要求。

3通过图形工作站的软件对网络高清摄像机进行抓图，再通过软件得出所抓取图片的分辨率大小，结果应符合设计要求。

4用照度表检测监视区域内的照度，结果应符合设计要求。

5用秒表测试前端设备与用户终端设备间端到端的信息延迟时间，重复测试3次，均不应大于4s。

6存储设备的设置应符合设计要求。计算系统的图像存储时间应符合设计方案及公安业务管理的要求。监控中心的存储设备或数据库，应能同时存储与录像资料相关的检索信息，如设备、通道、时间、报警信息等。无论采用何种存储方式，系统应能存储下列信息并保持相应时间：

1)当配置报警联动功能时，存储报警发生前后一段时间内的视音频信息；

2)监控中心操作员人工指定或通过编程定时指定的现场视音频信息。

18.9.5出入口控制系统的功能检测应符合下列规定：

1通过智能卡或其他相应方式，对出入识读装置的识读功能进行检测，系统应能将识读信息传递给管理/控制部分，也可通过管理/控制部分的指令来进行操控。

2信息处理/控制设备功能检测应符合下列规定：

1)检测信息处理/控制/管理功能，应符合《出入口控制系统技术要求》GA/T394的有关规定；

2)检测对不同准入级别的对象，应具有实时控制和多级

程序控制功能；

3)使用不同级别的证卡识读不同级别的入口，应具有不同的识别密码；

4)检测有效证卡的密码修改功能，密码系统应有防非法复制功能；

5)控制设备对执行机构的控制应准确、可靠；

6)检测每次有效进入行为应能自动存储进入人员的相关信息和进入时间，并能做统计和记录存档。所有出入口数据都应能进行统计、筛选等数据处理；

7)查看系统应具有多级密码管理，系统的任何操作都应有相应记录；

8)检测系统的紧急开启功能。

3执行机构的动作应实时、安全、可靠，每次有效操作应只有一次有效动作。

4报警功能检测应符合下列规定：

1)模拟非授权进入或超时开启情况，系统应能发出报警，并能显示出非授权进入、超时开启发生的时间、区域或部位，应与授权进入显示有明显区别；

2)模拟识读装置和执行机构故障，系统应能发出报警。

5访客(可视)对讲系统功能检测应符合下列规定：

1)检测室外机与室内机双向通话，声音应清晰无明显噪声；

2)检测室内机开锁机构的工作状况；

3)检测电控开锁及手动开锁的工作状况；

4)访客(可视)对讲系统应具备报警功能；

5)访客(可视)对讲系统的图像应清晰、稳定；

18.9.6出入口控制系统的性能检测应符合下列规定：

1采用现场模拟的方法，检测识别器的“误识”和“拒识”情况；有效卡在识别后应能给出放行信号，用秒表检测识读响应时间，应符合设计要求；

2采用现场模拟的方法，用测距工具检测非接触式识别器的识别距离，应符合设计要求；

3使用声级计在室内机检测访客(可视)对讲系统振铃声压级，检测结果应符合设计要求。

18.9.7电子巡查系统的功能检测应符合下列规定：

1巡查设置功能检测应符合下列规定：

1)检测在线式系统保安人员巡查程序，应能对巡逻状态实时监督记录，当保安人员不到位时应能发出报警；

2)离线式系统信息识读应准确、可靠。

2系统管理功能检测应符合下列规定：

1)查看系统的多级管理密码，系统中发生的各种事件、状态应有记录；

2)应检测执行器编号、执行时间的记录打印功能。

18.9.8停车库(场)出入口的管理功能检测应符合下列规定：

1模拟车辆识别，应准确可靠，功能应符合设计要求；

2手动控制出入挡车器，应安全可靠；

3模拟意外情况发生，系统应能报警；

4验证出票验票功能，应符合设计要求。

18.9.9停车库(场)综合管理功能检测应符合下列规定：

1检测停车场的收费统计和管理功能，应能在安防监控中心对该功能进行核实；

2检测停车场的显示功能，应能显示车位、出入口指示、计费和收费金额等；

3停车场的报表及其打印功能应符合设计要求；

4在现场及管理系统终端检测从车辆探测器获得的车辆占用信息应实时可靠，引导信息反馈到指示信号器应有效，车位数量和占用情况在各级显示屏和管理界面应能实时同步。

18.9.10安全防范综合管理功能检测应符合下列规定：

1通过查看各子系统的设防情况及防范功能，查看总防范范围和防范效果；重点防范部位的设防情况、防范范围和防范效

果应符合设计要求；

2在监控中心检查图像质量以及图像或其他信息记录保存时间，看能否满足管理需求和设计要求。

3系统时钟同步功能检测应符合下列规定：

1)有时钟源的系统，时钟源服务应能正常开启，与服务对象之间通信应正常；对需要时间同步的设备进行抽检，相应的时钟同步服务应开启，应能设置到对应的时钟源地址；更改需要时间同步设备的本地时间，在定期同步时间到期时，本地时间应能够自动变化为时钟源时间；

2)无时钟源有时间服务的系统，提供时间服务的设备应能正常工作，时间服务应正常开启，与服务对象之间通信应正常；对需要时间同步的设备进行抽检，相应的时钟同步服务应开启，应能设置到提供时间服务的设备；更改需要时间同步设备的本地时间，在定期同步时间到期时，本地时间应能够自动变化为时钟服务设备时间；

3)无时间服务无时钟源的系统，各部分时间设置应在统一时区；各部分显示时间应一致(精确到分钟或以用户要求为准)。

18.9.11对子系统的管理功能检测应符合下列规定：

1模拟入侵报警的发生，在监控中心检测视频监控系统和出入口控制系统的动作，报警现场的画面应能自动调入，同时相关门禁系统应能对事件进行响应；

2在监控中心向子系统发出控制命令，现场的动作响应应准确可靠，响应时间应符合设计要求；

3在监控中心查看各子系统工作状态显示，同时在现场做情况核对；模拟报警发生，在监控中心检测报警信息的准确性；检测从现场报警发生到监控中心显示报警信息的时间，该响应时间应符合设计要求。

19应急响应系统

19.1一般规定

19.1.1智能建筑应急响应系统工程质量检测内容应包括系统功能和系统性能。

19.1.2检测环境及条件除应符合本标准第3.2.5规定外，本系统检测应在其关联子系统检测合格后进行。

19.1.3系统功能和系统性能全数检测，检测结果应符合设计要求，被检项目合格率应为100%。

19.2系统功能

19.2.1应急响应系统报警功能检测应包括下列内容：

1实时报警功能；

2就地和异地报警功能。

19.2.2应急响应系统指挥调度功能检测应包括下列内容：

1有线、无线通信功能；

2火灾自动报警系统与安全技术防范系统的联动功能；

3火灾自动报警系统与建筑设备管理系统的联动功能；

4紧急广播系统与信息引导及发布系统的联动功能；

5接收上级应急指挥系统指令功能。

19.2.3应急响应事故处置系统功能检测应包括下列内容：

1消防灭火功能；

2安全事件处置功能。

19.3系统性能

19.3.1报警系统应检测系统报警响应时间。

19.3.2指挥调度系统应检测系统指挥调度命令响应时间。

19.3.3事故处置系统应检测系统事故处置响应时间。

19.4检测设备

19.4.1计时器的准确度应为±0.1s。

19.4.2用于网络检测的设备应符合本标准第7章的有关检测设备的规定。

19.5检测方法

19.5.1应急响应系统报警功能检测应符合下列规定：

1应模拟突发事件，在应急系统控制端检测报警信号的实时性，控制主机端应有报警地点、时间等信息显示；

2应模拟突发事件，通过远程平台检测异地报警功能，查看事件发生地点，并能远程启动报警系统。

19.5.2应急响应系统指挥调度功能检测应符合下列规定：

1应模拟突发事件，检测应急响应系统与火灾自动报警系统、安全技术防范系统、建筑设备管理系统等相关联系统的有线和无线通信功能；

2应模拟突发事件，在应急响应系统平台上观察和操控火灾自动报警系统、安全技术防范系统、建筑设备管理系统等相关联系统，检测与相关系统的联动功能；

3应模拟触发应急安全指令，应急广播系统应能按照应急发布预案播放相关人员疏散语音广播；检测信息导引与发布系统能按照应急发布预案发布相关人员疏散指导信息；

4应模拟应急响应上级系统发布指令，检测指挥调度平台指令接收及执行情况。

19.5.3应急响应事故处置系统功能检测应符合下列规定：

1模拟火灾事件，检测火灾报警系统中子系统的运行情况，检测结果应符合设计要求；

2模拟安全事件，检测处置系统中子系统的运行情况，检测结果应符合设计要求。

19.5.4应急响应系统中各系统的响应时间检测应采用计时器测量。

20机房工程

20.1一般规定

20.1.1智能建筑机房工程质量检测内容应包括供配电系统、空气调节系统、给水排水系统、监控与安全防范系统和机房室内环境。

20.1.2检测环境及条件除应符合本标准第3.2.5条规定外，尚应符合下列规定：

1测试前应对整个机房进行清洁处理；

2空气调节系统连续运行时间不应少于48h。

20.1.3所有项目全数检测。所检测项目的结果应符合设计要求；当设计文件无明确要求时，应符合相关规范要求。被检项目合格率应为100%。

20.2供配电系统

20.2.1市电电源质量检测应包括下列内容：

1稳态电压偏移；

2频率偏移；

3电压波形畸变率；

4零地电压。

20.2.2不间断电源质量检测应包括下列内容：

1稳态电压偏移；

2频率偏移；

3电压波形畸变率；

4零地电压；

5市电与不间断电源装置切换时间；

6市电与柴油发电机切换时间。

20.2.3柴油发电机电源质量检测应包括下列内容：

1稳态电压偏移；

2频率偏移；

3电压波形畸变率；

4零地电压。

20.3空气调节系统

20.3.1机房内空气调节系统性能检测应包括下列内容：

1空气温度；

2空气相对湿度；

3空气含尘浓度；

4风速和风量；

5机房与走廊静压差。

20.3.2空气调节系统功能检测应包括下列内容：

1温度调节功能；

2湿度调节功能；

3风量调节功能。

20.4给水排水系统

20.4.1给水排水系统防水功能检测应包括下列内容：

1挡水坝高度；

2排水管道畅通性。

20.4.2给水排水系统应检测漏水报警功能。

20.5监控与安全防范系统

20.5.1环境监控系统检测应包括下列内容：

1机房环境参数监视功能；

2漏水报警监视功能。

20.5.2场地设备监控系统检测应包括下列内容：

1设备监视控制功能；

2故障报警功能。

20.5.3安全技术防范系统检测内容应按照本标准第18章的有关规定执行。

20.6机房室内环境

20.6.1机房空间环境检测应包括下列内容：

1机房长度、宽度、净高、面积；

2机房门长度、宽度、高度；

3噪声；

4工作面水平照度；

5静电地板泄漏电阻；

6无线电干扰场强；

7磁场干扰场强。

20.6.2机房空气质量检测应包括下列内容：

1CO含量；

2CO₂含量。

20.7检测设备

20.7.1电能质量分析仪电压测量范围应为0V~500V,准确度应为±0.1V,频率准确度应为±0.15Hz,失真度准确度应为±3%~±5%。

20.7.2照度计测量范围应为1lx~2000lx,准确度应为±1lx。

20.7.3示波器时基分辨力应为1×10-⁶s。

20.7.4温度测试仪测量范围应为-20℃~60℃,准确度应为±0.1℃。

20.7.5湿度测试仪测量范围应为5%RH～95%RH,准确度应为±0.1%RH。

20.7.6尘埃粒子计数器测量范围应为8级～9级，分辨力应为1粒。

20.7.7微压差计测量范围应为-1kPa～1kPa,准确度应为

±1%。

20.7.8风速计测量范围应为0m/s～30m/s,准确度应为±5%,读数应为±3字。

20.7.9CO测试仪测量范围应为Oppm～999ppm,分辨力应为lppm。

20.7.10CO₂测试仪测量范围应为Oppm～999ppm,分辨力应为lppm。

20.7.11声级计不应低于现行国家标准《电声学声级计第1部分：规范》GB/T3785.1中规定的2级声级计。

20.7.12电阻测试仪测量范围应为0.01Ω~2.5×10¹⁵Ω,分辨力应为0.01Ω。

20.7.13场强测量仪频率测量范围应为0.1MHz～1000MHz,准确度应为±2dB。

20.7.14高斯仪测量范围应为20mG~2000mG,准确度应为±5%。

20.7.15风量罩精度应为0.1m³/h,测量范围应为100m³/h~3000m³/h,准确度应为5%。

20.8检测方法

20.8.1供配电系统检测应符合下列规定：

1使用电能质量分析仪在市电电源配电柜、不间断电源装置和柴油发电机的输出端测量电压、频率、畸变率和零地电压。电压偏移范围应为±3%~±5%,频率偏移范围应为—0.5Hz~0.5Hz;电压波形畸变率应小于5%,零地电压应小于2V;

2使用示波器连接不间断电源和市电电源，人为切断市电电源，通过示波器查看不间断电源和市电之间切换时间，允许断电持续时间应按现行国家标准《数据中心设计规范》GB50174中的相关规定执行。

20.8.2空气调节系统性能检测应符合下列规定：

1温度、湿度检测应符合下列规定：

1)机房内的温度、相对湿度应满足通信信息设备的使用要求，使用温度、湿度测试仪，在机房区域进行测试。

2)检测应在电子设备正常运行1h之后进行。

3)测试点应选择离地面0.8m,距设备周围0.8m以外，并应避开送、回风口。机房面积在50m²以下布5个测点，按图20.8.2对角线5点布置。每增加20m²~50m²增加3个～5个测点；每个测点连续三次测试，取其平均值为该点的实测数值，各测点的实测数值均代表房间内的温湿度。


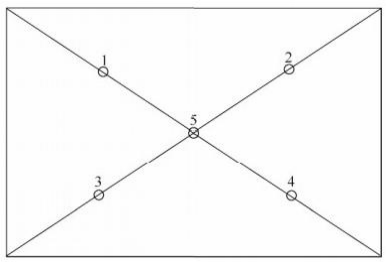


图20.8.2机房布点示意

2空气含尘浓度检测应符合下列规定：

1)电子设备机房的含尘浓度检测应在房间及空调系统彻底打扫后，并在空调系统正常运行24h以后进行。

2)粒径大于或等于0.5μm的尘粒计数宜采用光散射粒子计数法。

3)采样管内壁应干净，连接处不得渗漏；采样管的长度应依据仪器允许长度，当无规定时不宜大于1.5m;测试人员应在采样口的下风侧取样。

4)机房面积不大于50m²时，应按图20.8.2对角线5点布置。每增加20m²~50m²增加3个~5个测点；每个

测点连续三次测试，取其平均值为该点的实测数值，各测点的实测数值均代表房间内的含尘数量，机房空气中粒径大于或等于0.5μm的悬浮粒子数应小于

1.76×10⁷粒/m³。

3风速、风量检测应符合下列规定：

1)风口处的风速用风速仪测量，测量时应贴近格栅及网格。

2)风量采用风量罩测量法检测送风口风量，根据待测风口的尺寸、面积，选择与风口的面积较接近的风量罩罩体，且罩体的长边长度不得超过风口的长边长度的3倍；风口的面积不应小于罩体边界面积的15%,选择合适的罩体后，确定罩体的摆放位置来罩住风口，风口宜位于罩体的中间位置，保证无漏风，观察仪表的显示值，待显示值趋于稳定后，读取风量值。

4机房与外界静压差检测应符合下列规定：

1)测试时应关闭室内所有门窗；

2)检测点应选择室内气流扰动较小的位置；

3)测量时接口不应迎着气流方向，且应避免乳胶管被挤压而影响气流向微压计传感器的传送；

4)机房应维持正压，与走廊及辅助房间静压差不宜小于5Pa,与室外静压差不宜小于10Pa。

20.8.3空气调节系统功能检测应符合下列规定：

1人为调高和降低空调温度设定值2℃~3℃,机房室内温变化应能达到设定值并稳定运行；

2人为调高和降低空调湿度设定值59%～10%,机房室内湿度变化应能达到设定值并稳定运行。

3人为适量改变空调风量设定值，机房风量变化应能达到设定值并稳定运行。

20.8.4给水排水系统检测应符合下列规定：

1使用卷尺现场测量挡水坝高度，符合设计要求为合格，

通过目测观察水管压力表现场读数，给水管压力表指示应符合设计要求；

2检测排水管道畅通性时，可采用地漏周围临时围拢圆形小水坝的方法，水量以1桶水为准，瞬时向小水坝内倾入1桶水，并记录排走时间。制冷剂换热空调，因只需排走加湿漏水，水流尽时间应在30s内；冷冻水换热空调，水流尽时间应在10s内，且地漏数量应每10m²不少于1个。

3人为模拟漏水，检查漏水报警系统应符合设计要求。

20.8.5监控与安全防范系统检测应符合下列规定：

1环境监控系统检测应符合下列规定：

1)使用目测观察法，后台数据与前台显示数据应保持

一致；

2)人为模拟漏水情况，报警功能应能及时准确地显示数据。

2场地设备监控系统检测应符合下列规定：

1)使用目测观察法，后台数据与前台显示数据应保持

一致；

2)人为模拟故障报警情况，报警功能应能及时准确地显示数据。

3安全技术防范系统检测方法应按本标准第18章的有关规定执行。

20.8.6机房环境系统检测应符合下列规定：

1机房净高和门宽度、高度可使用卷尺在机房内进行测量。机房室内装修净高应根据机柜高度及通风要求确定，且不宜小于2.6m。

2机房噪声检测应符合下列规定：

1)电子设备、系统停机时在主机房中心处进行检测，测量噪声时，声级计的传感器应离开墙壁、地板等反射面一定距离，测量时高度距地面1.2m~1.5m;

2)测量时如背景噪声较大，会产生测量误差；如果被测

点前后两次噪声测量值差值在10dB以上，可忽略噪声的影响；若其差值在10dB以内，且背景噪声并无变化时，可进行修正；

3)取测量的稳定值，即为该房间的噪声值，在主操作员位置测量的噪声值应小于65dB。

3检测机房内的正常照明时，使用照度计在房间内距墙面1m、距地面0.75m的水平工作面上进行测试；测试点选择3个～5个点，大面积房间可多选几点进行测试。主机房照度不应低于500lx,机房通道疏散照明的照度值不应低于51x。

4静电泄露电阻检测方法应符合现行行业标准《防静电活动地板通用规范》SJ/T10796的有关规定。

5在电子设备机房内任一点使用场强仪进行检测，取最大值为测试值。无线电干扰频率为0.15MHz～1000MHz时，主机房和辅助区内的无线电干扰场强不应大于126dB。

6在电子机房内任意一点使用交直流高斯计进行检测，检测主机房和辅助区内磁场干扰环境场强不应大于800A/m。

7CO、CO₂含量检测应使用环境测试仪检测，在电子设备机房内任选一点进行检测，CO含量应小于10ppm,CO₂含量应小于1000ppm。

21防雷与接地系统

21.1一般规定

21.1.1智能建筑防雷与接地系统工程质量检测内容应包括智能化各系统的接地装置、接地线、等电位联结、屏蔽设施、电涌保护器各系统的防雷与接地。

21.1.2检测环境及条件除应符合本标准第3.2.5条规定外，尚应符合下列规定：

1天气环境应符合检测要求；

2检测前相关的隐蔽工程应已验收合格。

21.1.3智能化系统的防雷与接地应全数检测。检测结果符合设计要求为合格，被检项目的合格率应为100%。

21.2接地装置

21.2.1应查看室外接地测试点的设置数量和设置位置，并检测连接板与接地干线的连接质量。

21.2.2通过室外接地测试点检测接地装置的接地电阻值。

21.3接地线

21.3.1接地线的检测应包括下列内容：

1接地引出线；

2垂直接地干线；

3楼层端子板与机房局部端子板之间的连接导体；

4机房局部端子板之间的连接导体；

5机房内的等电位接地网格；

6智能化系统设备与机房等电位联结网格之间的连接导体。

21.3.2各类接地线的连通质量检测应包括连接方式、接触面积

以及材料和规格等。

21.4等电位联结

21.4.1等电位联结外观检测应包括下列内容：

1多重互联形成的等电位联结网络；

2总等电位接地端子板、楼层等电位接地端子板和机房局部端子板或机房等电位接地网格；

3智能化系统设备的等电位联结。

21.4.2等电位联结效果检测应包括下列内容：

1总等电位端子板的接地电阻值；

2等电位体的电气连通性。

21.5屏蔽设施

21.5.1智能化系统屏蔽设施检测应包括下列内容：

1机房的屏蔽措施；

2信号电缆的屏蔽措施。

21.5.2智能化系统的电力线缆与信号线缆应隔离敷设，对其安全距离进行检测。

21.6电涌保护器

21.6.1电信和信号网络中电涌保护器的设置检测应包括下列内容：

1出入建筑物的信号传输线，在出入口端检查电涌保护器的设置；

2建筑物内的信号传输线，在各级雷电防护区的交界处检查电涌保护器的设置；

3置于户外的智能系统前端设备，在输入输出端口检查电涌保护器的设置；

4电涌保护器的接地情况。

21.6.2限压型电涌保护器的检测应包括下列内容：

1外观和状态指示器；

2两端的连接线；

3压敏电阻的直流参考电压；

4接线端与电涌保护器壳体间的绝缘电阻；

5接地线连接的有效性。

21.7各系统的防雷与接地

21.7.1机房工程的防雷与接地检测应包括下列内容：

1机房局部等电位接地端子板；

2各类等电位接地端子板之间的连接导体；

3机房等电位联结网络与共用接地系统连接情况；

4机房设备接地连接情况。

21.7.2信息网络系统的防雷与接地检测应包括下列内容：

1进、出建筑物的传输线路上的信号电涌保护器；

2网络交换机、集线器、光电端机的配电箱内的电源电涌保护器；

3引入建筑物的电涌保护器的接地线与等电位端子板连接；

4测量设备处信号电涌保护器的接地线的截面积，并检测与机架或机房等电位联结情况；

5计算机网络的安全保护地、信号工作地、屏蔽接地、防静电接地和电涌保护器接地等与等电位联结网络的连接。

21.7.3安全防范系统的防雷与接地检测应包括下列内容：

1监控室外立杆及其主要构件的接地装置，并测量接地装置接地电阻；

2置于户外摄像机的视频信号线路电涌保护器、控制信号线接口处(如RS485、RS424等)电涌保护器，解码箱处供电线路配置的电源线路电涌保护器；

3主控机、分控机的信号控制线、通信线、各监视器的报警信号线，在线路进出建筑物直击雷非防护区(LPZOA)或直击雷防护区(LPZOB)与第一防护区(LPZ1)边界处，所配置

的线路电涌保护器；

4系统视频、控制信号线路及供电线路的电涌保护器；

5系统的户外供电线路、视频信号线路、控制信号线路敷设方式和接地情况；

6监控中心及设备机房的等电位联结网络。

21.7.4建筑设备监控系统防雷接地检测应包括下列内容：

1建筑设备监控系统的各种线路，包括电源线、通信线、控制线等，在线路进出建筑物直击雷非防护区(LPZOA)或直击雷防护区(LPZOB)与第一防护区(LPZ1)边界处，所配置的线路电涌保护器；

2系统中央控制室的等电位联结网络；

3室内所有设备金属机架(壳)、金属线槽、保护接地和电涌保护器的接地与等电位联结网的连接；

4测量系统接地干线多股铜芯绝缘导线的截面积。

21.7.5有线电视系统的防雷与接地检测应包括下列内容：

1进、出有线电视系统前端机房的金属芯信号传输线，在入、出口处所配置的电涌保护器；

2有线电视网络前端机房内的等电位接地端子板；

3测量系统接地干线多股铜芯绝缘导线的截面积；

4设备机房内电子设备的金属外壳、线缆金属屏蔽层、电涌保护器的接地以及PE线与等电位接地端子板的连接；

5有线电视信号传输线路所配置的电涌保护器；地区多雷区、强雷区的用户端终端放大器所配置的电涌保护器；

6有线电视信号传输网络的光缆、同轴电缆的承重钢绞线，在建筑物入户处的等电位联结接地；光缆内的金属加强芯及金属护层的接地情况。

21.8检测设备

21.8.1数字兆欧表的测量范围应为1MQ～2000MΩ,分辨力不应小于1MQ,准确度应为±5%,读数应为±2字。

21.8.2接地电阻测试仪的测量范围应为0Ω~20Ω,准确度应为±2%,读数应为±1字。

21.8.3数字式万用表的准确度应为±2%,读数±1字。

21.8.4毫欧表的准确度应为±0.5%,读数应为±1字。

21.8.5钢直尺的准确度应为±1mm。

21.8.6长卷尺或测距设备的准确度应为±1cm。

21.8.7游标卡尺的准确度应为±0.05mm。

21.9检测方法

21.9.1接地装置的检测应符合下列规定：

1检查室外接地测试点的数量和位置，应与设计图纸一致；检查连接板的材料和规格，并检测连接板的连接情况；

2应用接地电阻测试仪检测接地装置的接地电阻值，测量三次，取算术平均值，测量结果应符合设计要求。

21.9.2接地线的检测应符合下列规定。

1接地线的设置应符合下列规定：

1)在竖井中敷设的垂直接地干线与总等电位接地端子板应可靠连接，与建筑物各层钢筋或均压带应连通；

2)检查垂直接地干线与各楼层接地端子板连接情况；

3)检查垂直接地干线与机房局部等电位接地端子板连接情况；

4)检查机房接地网格与机房局部等电位接地端子板连接情况；

5)检查智能化设备与等电位接地网络连接情况。

2测量各类连接导体的截面积。

3测量接地线的连接导通情况。

21.9.3等电位联结的检测应符合下列规定。

1等电位联结的设置应符合下列规定：

1)检测等电位联结情况，应包括等电位联结网络的多重互联和等电位联结网络与接地装置的联结；

2)应检测总等电位的联结情况，包括各楼层等电位接地端子板和机房等电位接地端子板的设置；

3)应检测智能化设备和各种接地方式的等电位联结情况。

2采用接地电阻测试仪检测总等电位接地端子板的接地电阻值，每个接地端子板应测量三次，应取算术平均值作为该点的接地电阻值。测量等电位联结网均应处于良好的电气连通状态。

21.9.4屏蔽设施的检测应符合下列规定：

1检查机房位置设置的合理性，机房位置应处于第一防护区之后的防雷区域；逐一检测进入机房的金属导体、电缆屏蔽层及金属线槽，均应有等电位联结；

2检查屏蔽电缆的接地情况，非屏蔽电缆在金属管道中敷设，金属管道应做有效的接地连接；

3电力电缆和信号缆线应隔离敷设，安全距离应符合设计要求。

21.9.5电涌保护器的检测应符合下列规定：

1电信和信号网络中电涌保护器的检测应符合下列规定：

1)进出建筑物的信号传输线应在出入口端设置电涌保护器，并应符合设计要求；

2)建筑物内的信号传输线在各级雷电防护区的交界处应设置电涌保护器，并应符合设计要求；

3)在户外的系统前端设备的输入输出端口应设置电涌保护器，并应符合设计要求；

4)电涌保护器的接地线应就近做等电位联结，连接线应短直。

2限压型电涌保护器的检测应符合下列规定：

1)检查电涌保护器的外观，应无裂痕、烧灼痕或变形，标识的Up、Uc、In等参数应清晰；对比产品说明，状态指示器应处于正常状态；

2)电涌保护器两端的连接线应短直，其长度之和不宜超过0.5m;

3)将电涌保护器与所连接线路断开，用防雷元件测试仪在两端逐步加压，直到流通电流达到1mA时的电压即为直流参考电压，该测试值应在电涌保护器标称的直流参考电压的±10%范围内；

4)将电涌保护器与所连接线路断开，用绝缘电阻测试仪(兆欧表)正负极各测一次，施加500V电压1min,待测量值稳定后读取，绝缘电阻不应小于50MΩ。

5)用毫欧表检测电涌保护器接地线与等电位联结带之间的过渡电阻，不应大于0.03Ω。

21.9.6各系统的防雷与接地应符合本标准第21.2节～第21.6节相关检测方法。

中华人民共和国住房和城乡建设部
2019年3月27日

**P15**

**Guidance on Promoting the Coordinated Development of Intelligent Construction and Construction Industrialization**

**住房和城乡建设部等部门关于推动智能建造与建筑工业化协同发展的指导意见**

建市〔2020〕60号

各省、自治区、直辖市及计划单列市、新疆生产建设兵团住房和城乡建设厅（委、管委、局）、发展改革委、科技厅（局）、工业和信息化厅（局）、人力资源社会保障厅（局）、生态环境厅（局）、交通运输厅（局、委）、水利厅（局）、市场监管局，北京市规划和自然资源委，国家税务总局各省、自治区、直辖市和计划单列市税务局，各银保监局，各地区铁路监督管理局，民航各地区管理局：

建筑业是国民经济的支柱产业，为我国经济持续健康发展提供了有力支撑。但建筑业生产方式仍然比较粗放，与高质量发展要求相比还有很大差距。为推进建筑工业化、数字化、智能化升级，加快建造方式转变，推动建筑业高质量发展，制定本指导意见。

一、指导思想

以习近平新时代中国特色社会主义思想为指导，全面贯彻党的十九大和十九届二中、三中、四中全会精神，增强“四个意识”，坚定“四个自信”，做到“两个维护”，坚持稳中求进工作总基调，坚持新发展理念，坚持以供给侧结构性改革为主线，围绕建筑业高质量发展总体目标，以大力发展建筑工业化为载体，以数字化、智能化升级为动力，创新突破相关核心技术，加大智能建造在工程建设各环节应用，形成涵盖科研、设计、生产加工、施工装配、运营等全产业链融合一体的智能建造产业体系，提升工程质量安全、效益和品质，有效拉动内需，培育国民经济新的增长点，实现建筑业转型升级和持续健康发展。

二、基本原则

市场主导，政府引导。充分发挥市场在资源配置中的决定性作用，强化企业市场主体地位，积极探索智能建造与建筑工业化协同发展路径和模式，更好发挥政府在顶层设计、规划布局、政策制定等方面的引导作用，营造良好发展环境。

立足当前，着眼长远。准确把握新一轮科技革命和产业变革趋势，加强战略谋划和前瞻部署，引导各类要素有效聚集，加快推进建筑业转型升级和提质增效，全面提升智能建造水平。

跨界融合，协同创新。建立健全跨领域跨行业协同创新体系，推动智能建造核心技术联合攻关与示范应用，促进科技成果转化应用。激发企业创新创业活力，支持龙头企业与上下游中小企业加强协作，构建良好的产业创新生态。

节能环保，绿色发展。在建筑工业化、数字化、智能化升级过程中，注重能源资源节约和生态环境保护，严格标准规范，提高能源资源利用效率。

自主研发，开放合作。大力提升企业自主研发能力，掌握智能建造关键核心技术，完善产业链条，强化网络和信息安全管理，加强信息基础设施安全保障，促进国际交流合作，形成新的比较优势，提升建筑业开放发展水平。

三、发展目标

到2025年，我国智能建造与建筑工业化协同发展的政策体系和产业体系基本建立，建筑工业化、数字化、智能化水平显著提高，建筑产业互联网平台初步建立，产业基础、技术装备、科技创新能力以及建筑安全质量水平全面提升，劳动生产率明显提高，能源资源消耗及污染排放大幅下降，环境保护效应显著。推动形成一批智能建造龙头企业，引领并带动广大中小企业向智能建造转型升级，打造“中国建造”升级版。

到2035年，我国智能建造与建筑工业化协同发展取得显著进展，企业创新能力大幅提升，产业整体优势明显增强，“中国建造”核心竞争力世界领先，建筑工业化全面实现，迈入智能建造世界强国行列。

四、重点任务

（一）加快建筑工业化升级。

大力发展装配式建筑，推动建立以标准部品为基础的专业化、规模化、信息化生产体系。加快推动新一代信息技术与建筑工业化技术协同发展，在建造全过程加大建筑信息模型（BIM）、互联网、物联网、大数据、云计算、移动通信、人工智能、区块链等新技术的集成与创新应用。大力推进先进制造设备、智能设备及智慧工地相关装备的研发、制造和推广应用，提升各类施工机具的性能和效率，提高机械化施工程度。加快传感器、高速移动通讯、无线射频、近场通讯及二维码识别等建筑物联网技术应用，提升数据资源利用水平和信息服务能力。加快打造建筑产业互联网平台，推广应用钢结构构件智能制造生产线和预制混凝土构件智能生产线。

（二）加强技术创新。

加强技术攻关，推动智能建造和建筑工业化基础共性技术和关键核心技术研发、转移扩散和商业化应用，加快突破部品部件现代工艺制造、智能控制和优化、新型传感感知、工程质量检测监测、数据采集与分析、故障诊断与维护、专用软件等一批核心技术。探索具备人机协调、自然交互、自主学习功能的建筑机器人批量应用。研发自主知识产权的系统性软件与数据平台、集成建造平台。推进工业互联网平台在建筑领域的融合应用，建设建筑产业互联网平台，开发面向建筑领域的应用程序。加快智能建造科技成果转化应用，培育一批技术创新中心、重点实验室等科技创新基地。围绕数字设计、智能生产、智能施工，构建先进适用的智能建造及建筑工业化标准体系，开展基础共性标准、关键技术标准、行业应用标准研究。

（三）提升信息化水平。

推进数字化设计体系建设，统筹建筑结构、机电设备、部品部件、装配施工、装饰装修，推行一体化集成设计。积极应用自主可控的BIM技术，加快构建数字设计基础平台和集成系统，实现设计、工艺、制造协同。加快部品部件生产数字化、智能化升级，推广应用数字化技术、系统集成技术、智能化装备和建筑机器人，实现少人甚至无人工厂。加快人机智能交互、智能物流管理、增材制造等技术和智能装备的应用。以钢筋制作安装、模具安拆、混凝土浇筑、钢构件下料焊接、隔墙板和集成厨卫加工等工厂生产关键工艺环节为重点，推进工艺流程数字化和建筑机器人应用。以企业资源计划（ERP）平台为基础，进一步推动向生产管理子系统的延伸，实现工厂生产的信息化管理。推动在材料配送、钢筋加工、喷涂、铺贴地砖、安装隔墙板、高空焊接等现场施工环节，加强建筑机器人和智能控制造楼机等一体化施工设备的应用。

（四）培育产业体系。

探索适用于智能建造与建筑工业化协同发展的新型组织方式、流程和管理模式。加快培育具有智能建造系统解决方案能力的工程总承包企业，统筹建造活动全产业链，推动企业以多种形式紧密合作、协同创新，逐步形成以工程总承包企业为核心、相关领先企业深度参与的开放型产业体系。鼓励企业建立工程总承包项目多方协同智能建造工作平台，强化智能建造上下游协同工作，形成涵盖设计、生产、施工、技术服务的产业链。

（五）积极推行绿色建造。

实行工程建设项目全生命周期内的绿色建造，以节约资源、保护环境为核心，通过智能建造与建筑工业化协同发展，提高资源利用效率，减少建筑垃圾的产生，大幅降低能耗、物耗和水耗水平。推动建立建筑业绿色供应链，推行循环生产方式，提高建筑垃圾的综合利用水平。加大先进节能环保技术、工艺和装备的研发力度，提高能效水平，加快淘汰落后装备设备和技术，促进建筑业绿色改造升级。

（六）开放拓展应用场景。

加强智能建造及建筑工业化应用场景建设，推动科技成果转化、重大产品集成创新和示范应用。发挥重点项目以及大型项目示范引领作用，加大应用推广力度，拓宽各类技术的应用范围，初步形成集研发设计、数据训练、中试应用、科技金融于一体的综合应用模式。发挥龙头企业示范引领作用，在装配式建筑工厂打造“机器代人”应用场景，推动建立智能建造基地。梳理已经成熟应用的智能建造相关技术，定期发布成熟技术目录，并在基础条件较好、需求迫切的地区，率先推广应用。

（七）创新行业监管与服务模式。

推动各地加快研发适用于政府服务和决策的信息系统，探索建立大数据辅助科学决策和市场监管的机制，完善数字化成果交付、审查和存档管理体系。通过融合遥感信息、城市多维地理信息、建筑及地上地下设施的BIM、城市感知信息等多源信息，探索建立表达和管理城市三维空间全要素的城市信息模型（CIM）基础平台。建立健全与智能建造相适应的工程质量、安全监管模式与机制。引导大型总承包企业采购平台向行业电子商务平台转型，实现与供应链上下游企业间的互联互通，提高供应链协同水平。

五、保障措施

（一）加强组织实施。各地要建立智能建造和建筑工业化协同发展的体系框架，因地制宜制定具体实施方案，明确时间表、路线图及实施路径，强化部门联动，建立协同推进机制，落实属地管理责任，确保目标完成和任务落地。

（二）加大政策支持。各地要将现有各类产业支持政策进一步向智能建造领域倾斜，加大对智能建造关键技术研究、基础软硬件开发、智能系统和设备研制、项目应用示范等的支持力度。对经认定并取得高新技术企业资格的智能建造企业可按规定享受相关优惠政策。企业购置使用智能建造重大技术装备可按规定享受企业所得税、进口税收优惠等政策。推动建立和完善企业投入为主体的智能建造多元化投融资体系，鼓励创业投资和产业投资投向智能建造领域。各相关部门要加强跨部门、跨层级统筹协调，推动解决智能建造发展遇到的瓶颈问题。

（三）加大人才培育力度。各地要制定智能建造人才培育相关政策措施，明确目标任务，建立智能建造人才培养和发展的长效机制，打造多种形式的高层次人才培养平台。鼓励骨干企业和科研单位依托重大科研项目和示范应用工程，培养一批领军人才、专业技术人员、经营管理人员和产业工人队伍。加强后备人才培养，鼓励企业和高等院校深化合作，为智能建造发展提供人才后备保障。

（四）建立评估机制。各地要适时对智能建造与建筑工业化协同发展相关政策的实施情况进行评估，重点评估智能建造发展目标落实与完成情况、产业发展情况、政策出台情况、标准规范编制情况等，并通报结果。

（五）营造良好环境。要加强宣传推广，充分发挥相关企事业单位、行业学协会的作用，开展智能建造的政策宣传贯彻、技术指导、交流合作、成果推广。构建国际化创新合作机制，加强国际交流，推进开放合作，营造智能建造健康发展的良好环境。

中华人民共和国住房和城乡建设部
中华人民共和国国家发展和改革委员会
中华人民共和国科学技术部
中华人民共和国工业和信息化部
中华人民共和国人力资源和社会保障部
中华人民共和国生态环境部
中华人民共和国交通运输部
中华人民共和国水利部
国家税务总局
国家市场监督管理总局
中国银行保险监督管理委员会
国家铁路局
中国民用航空局
2020年7月3日

**P16**

**Action Plan for Green Building Creation**

**绿色建筑创建行动方案**

建标〔2020〕65号

为全面贯彻党的十九大和十九届二中、三中、四中全会精神，深入贯彻习近平生态文明思想，按照《国家发展改革委关于印发〈绿色生活创建行动总体方案〉的通知》（发改环资〔2019〕1696号）要求，推动绿色建筑高质量发展，制定本方案。

一、 创建对象

绿色建筑创建行动以城镇建筑作为创建对象。绿色建筑指在全寿命期内节约资源、保护环境、减少污染，为人们提供健康、适用、高效的使用空间，最大限度实现人与自然和谐共生的高质量建筑。

二、创建目标

到2022年，当年城镇新建建筑中绿色建筑面积占比达到70%，星级绿色建筑持续增加，既有建筑能效水平不断提高，住宅健康性能不断完善，装配化建造方式占比稳步提升，绿色建材应用进一步扩大，绿色住宅使用者监督全面推广，人民群众积极参与绿色建筑创建活动，形成崇尚绿色生活的社会氛围。

三、重点任务

（一）推动新建建筑全面实施绿色设计。制修订相关标准，将绿色建筑基本要求纳入工程建设强制规范，提高建筑建设底线控制水平。推动绿色建筑标准实施，加强设计、施工和运行管理。推动各地绿色建筑立法，明确各方主体责任，鼓励各地制定更高要求的绿色建筑强制性规范。

（二）完善星级绿色建筑标识制度。根据国民经济和社会发展第十三个五年规划纲要、国务院办公厅《绿色建筑行动方案》（国办发〔2013〕1号）等相关规定，规范绿色建筑标识管理，由住房和城乡建设部、省级政府住房和城乡建设部门、地市级政府住房和城乡建设部门分别授予三星、二星、一星绿色建筑标识。完善绿色建筑标识申报、审查、公示制度，统一全国认定标准和标识式样。建立标识撤销机制，对弄虚作假行为给予限期整改或直接撤销标识处理。建立全国绿色建筑标识管理平台，提高绿色建筑标识工作效率和水平。

（三）提升建筑能效水效水平。结合北方地区清洁取暖、城镇老旧小区改造、海绵城市建设等工作，推动既有居住建筑节能节水改造。开展公共建筑能效提升重点城市建设，建立完善运行管理制度，推广合同能源管理与合同节水管理，推进公共建筑能耗统计、能源审计及能效公示。鼓励各地因地制宜提高政府投资公益性建筑和大型公共建筑绿色等级，推动超低能耗建筑、近零能耗建筑发展，推广可再生能源应用和再生水利用。

（四）提高住宅健康性能。结合疫情防控和各地实际，完善实施住宅相关标准，提高建筑室内空气、水质、隔声等健康性能指标，提升建筑视觉和心理舒适性。推动一批住宅健康性能示范项目，强化住宅健康性能设计要求，严格竣工验收管理，推动绿色健康技术应用。

（五）推广装配化建造方式。大力发展钢结构等装配式建筑，新建公共建筑原则上采用钢结构。编制钢结构装配式住宅常用构件尺寸指南，强化设计要求，规范构件选型，提高装配式建筑构配件标准化水平。推动装配式装修。打造装配式建筑产业基地，提升建造水平。

（六）推动绿色建材应用。加快推进绿色建材评价认证和推广应用，建立绿色建材采信机制，推动建材产品质量提升。指导各地制定绿色建材推广应用政策措施，推动政府投资工程率先采用绿色建材，逐步提高城镇新建建筑中绿色建材应用比例。打造一批绿色建材应用示范工程，大力发展新型绿色建材。

（七）加强技术研发推广。加强绿色建筑科技研发，建立部省科技成果库，促进科技成果转化。积极探索5G、物联网、人工智能、建筑机器人等新技术在工程建设领域的应用，推动绿色建造与新技术融合发展。结合住房和城乡建设部科学技术计划和绿色建筑创新奖，推动绿色建筑新技术应用。

（八）建立绿色住宅使用者监督机制。制定《绿色住宅购房人验房指南》，向购房人提供房屋绿色性能和全装修质量验收方法，引导绿色住宅开发建设单位配合购房人做好验房工作。鼓励各地将住宅绿色性能和全装修质量相关指标纳入商品房买卖合同、住宅质量保证书和住宅使用说明书，明确质量保修责任和纠纷处理方式。

四、组织实施

（一）加强组织领导。省级政府住房和城乡建设、发展改革、教育、工业和信息化、机关事务管理等部门，要在各省（区、市）党委和政府直接指导下，认真落实绿色建筑创建行动方案，制定本地区创建实施方案，细化目标任务，落实支持政策，指导市、县编制绿色建筑创建行动实施计划，确保创建工作落实到位。各省（区、市）和新疆生产建设兵团住房和城乡建设部门应于2020年8月底前将本地区绿色建筑创建行动实施方案报住房和城乡建设部。

（二）加强财政金融支持。各地住房和城乡建设部门要加强与财政部门沟通，争取资金支持。各地要积极完善绿色金融支持绿色建筑的政策环境，推动绿色金融支持绿色建筑发展，用好国家绿色发展基金，鼓励采用政府和社会资本合作（PPP）等方式推进创建工作。

（三）强化绩效评价。住房和城乡建设部会同相关部门按照本方案，对各省（区、市）和新疆生产建设兵团绿色建筑创建行动工作落实情况和取得的成效开展年度总结评估，及时推广先进经验和典型做法。省级政府住房和城乡建设等部门负责组织本地区绿色建筑创建成效评价，及时总结当年进展情况和成效，形成年度报告，并于每年11月底前报住房和城乡建设部。

（四）加大宣传推广力度。各地要组织多渠道、多种形式的宣传活动，普及绿色建筑知识，宣传先进经验和典型做法，引导群众用好各类绿色设施，合理控制室内采暖空调温度，推动形成绿色生活方式。发挥街道、社区等基层组织作用，积极组织群众参与，通过共谋共建共管共评共享，营造有利于绿色建筑创建的社会氛围。

中华人民共和国住房和城乡建设部
中华人民共和国国家发展和改革委员会
中华人民共和国教育部
中华人民共和国工业和信息化部
中国人民银行
国家机关事务管理局
中国银行保险监督管理委员会
2020年7月15日

**P17**

**Several Opinions on Accelerating the Development of New Types of Construction Industrialization**

**住房和城乡建设部等部门关于加快新型建筑工业化发展的若干意见**

建标规〔2020〕8号

各省、自治区、直辖市住房和城乡建设厅（委、管委）、教育厅（委）、科技厅（委、局）、工业和信息化主管部门、自然资源主管部门、生态环境厅（局），人民银行上海总部、各分行、营业管理部、省会(首府)城市中心支行、副省级城市中心支行，市场监管局（厅、委），各银保监局，新疆生产建设兵团住房和城乡建设局、教育局、科技局、工业和信息化局、自然资源主管部门、生态环境局、市场监管局：

新型建筑工业化是通过新一代信息技术驱动，以工程全寿命期系统化集成设计、精益化生产施工为主要手段，整合工程全产业链、价值链和创新链，实现工程建设高效益、高质量、低消耗、低排放的建筑工业化。《国务院办公厅关于大力发展装配式建筑的指导意见》（国办发〔2016〕71号）印发实施以来，以装配式建筑为代表的新型建筑工业化快速推进，建造水平和建筑品质明显提高。为全面贯彻新发展理念，推动城乡建设绿色发展和高质量发展，以新型建筑工业化带动建筑业全面转型升级，打造具有国际竞争力的“中国建造”品牌，提出以下意见。

一、加强系统化集成设计

（一）推动全产业链协同。推行新型建筑工业化项目建筑师负责制，鼓励设计单位提供全过程咨询服务。优化项目前期技术策划方案，统筹规划设计、构件和部品部件生产运输、施工安装和运营维护管理。引导建设单位和工程总承包单位以建筑最终产品和综合效益为目标，推进产业链上下游资源共享、系统集成和联动发展。

（二）促进多专业协同。通过数字化设计手段推进建筑、结构、设备管线、装修等多专业一体化集成设计，提高建筑整体性，避免二次拆分设计，确保设计深度符合生产和施工要求，发挥新型建筑工业化系统集成综合优势。

（三）推进标准化设计。完善设计选型标准，实施建筑平面、立面、构件和部品部件、接口标准化设计，推广少规格、多组合设计方法，以学校、医院、办公楼、酒店、住宅等为重点，强化设计引领，推广装配式建筑体系。

（四）强化设计方案技术论证。落实新型建筑工业化项目标准化设计、工业化建造与建筑风貌有机统一的建筑设计要求，塑造城市特色风貌。在建筑设计方案审查阶段，加强对新型建筑工业化项目设计要求落实情况的论证，避免建筑风貌千篇一律。

二、优化构件和部品部件生产

（五）推动构件和部件标准化。编制主要构件尺寸指南，推进型钢和混凝土构件以及预制混凝土墙板、叠合楼板、楼梯等通用部件的工厂化生产，满足标准化设计选型要求，扩大标准化构件和部品部件使用规模，逐步降低构件和部件生产成本。

（六）完善集成化建筑部品。编制集成化、模块化建筑部品相关标准图集，提高整体卫浴、集成厨房、整体门窗等建筑部品的产业配套能力，逐步形成标准化、系列化的建筑部品供应体系。

（七）促进产能供需平衡。综合考虑构件、部品部件运输和服务半径，引导产能合理布局，加强市场信息监测，定期发布构件和部品部件产能供需情况，提高产能利用率。

（八）推进构件和部品部件认证工作。编制新型建筑工业化构件和部品部件相关技术要求，推行质量认证制度，健全配套保险制度，提高产品配套能力和质量水平。

（九）推广应用绿色建材。发展安全健康、环境友好、性能优良的新型建材，推进绿色建材认证和推广应用，推动装配式建筑等新型建筑工业化项目率先采用绿色建材，逐步提高城镇新建建筑中绿色建材应用比例。

三、推广精益化施工

（十）大力发展钢结构建筑。鼓励医院、学校等公共建筑优先采用钢结构，积极推进钢结构住宅和农房建设。完善钢结构建筑防火、防腐等性能与技术措施，加大热轧H型钢、耐候钢和耐火钢应用，推动钢结构建筑关键技术和相关产业全面发展。

（十一）推广装配式混凝土建筑。完善适用于不同建筑类型的装配式混凝土建筑结构体系，加大高性能混凝土、高强钢筋和消能减震、预应力技术的集成应用。在保障性住房和商品住宅中积极应用装配式混凝土结构，鼓励有条件的地区全面推广应用预制内隔墙、预制楼梯板和预制楼板。

（十二）推进建筑全装修。装配式建筑、星级绿色建筑工程项目应推广全装修，积极发展成品住宅，倡导菜单式全装修，满足消费者个性化需求。推进装配化装修方式在商品住房项目中的应用，推广管线分离、一体化装修技术，推广集成化模块化建筑部品，提高装修品质，降低运行维护成本。

（十三）优化施工工艺工法。推行装配化绿色施工方式，引导施工企业研发与精益化施工相适应的部品部件吊装、运输与堆放、部品部件连接等施工工艺工法，推广应用钢筋定位钢板等配套装备和机具，在材料搬运、钢筋加工、高空焊接等环节提升现场施工工业化水平。

（十四）创新施工组织方式。完善与新型建筑工业化相适应的精益化施工组织方式，推广设计、采购、生产、施工一体化模式，实行装配式建筑装饰装修与主体结构、机电设备协同施工，发挥结构与装修穿插施工优势，提高施工现场精细化管理水平。

（十五）提高施工质量和效益。加强构件和部品部件进场、施工安装、节点连接灌浆、密封防水等关键部位和工序质量安全管控，强化对施工管理人员和一线作业人员的质量安全技术交底，通过全过程组织管理和技术优化集成，全面提升施工质量和效益。

四、加快信息技术融合发展

（十六）大力推广建筑信息模型（BIM）技术。加快推进BIM技术在新型建筑工业化全寿命期的一体化集成应用。充分利用社会资源，共同建立、维护基于BIM技术的标准化部品部件库，实现设计、采购、生产、建造、交付、运行维护等阶段的信息互联互通和交互共享。试点推进BIM报建审批和施工图BIM审图模式，推进与城市信息模型（CIM）平台的融通联动，提高信息化监管能力，提高建筑行业全产业链资源配置效率。

（十七）加快应用大数据技术。推动大数据技术在工程项目管理、招标投标环节和信用体系建设中的应用，依托全国建筑市场监管公共服务平台，汇聚整合和分析相关企业、项目、从业人员和信用信息等相关大数据，支撑市场监测和数据分析，提高建筑行业公共服务能力和监管效率。

（十八）推广应用物联网技术。推动传感器网络、低功耗广域网、5G、边缘计算、射频识别（RFID）及二维码识别等物联网技术在智慧工地的集成应用，发展可穿戴设备，提高建筑工人健康及安全监测能力，推动物联网技术在监控管理、节能减排和智能建筑中的应用。

（十九）推进发展智能建造技术。加快新型建筑工业化与高端制造业深度融合，搭建建筑产业互联网平台。推动智能光伏应用示范，促进与建筑相结合的光伏发电系统应用。开展生产装备、施工设备的智能化升级行动，鼓励应用建筑机器人、工业机器人、智能移动终端等智能设备。推广智能家居、智能办公、楼宇自动化系统，提升建筑的便捷性和舒适度。

五、创新组织管理模式

（二十）大力推行工程总承包。新型建筑工业化项目积极推行工程总承包模式，促进设计、生产、施工深度融合。引导骨干企业提高项目管理、技术创新和资源配置能力，培育具有综合管理能力的工程总承包企业，落实工程总承包单位的主体责任，保障工程总承包单位的合法权益。

（二十一）发展全过程工程咨询。大力发展以市场需求为导向、满足委托方多样化需求的全过程工程咨询服务，培育具备勘察、设计、监理、招标代理、造价等业务能力的全过程工程咨询企业。

（二十二）完善预制构件监管。加强预制构件质量管理，积极采用驻厂监造制度，实行全过程质量责任追溯，鼓励采用构件生产企业备案管理、构件质量飞行检查等手段，建立长效机制。

（二十三）探索工程保险制度。建立完善工程质量保险和担保制度，通过保险的风险事故预防和费率调节机制帮助企业加强风险管控，保障建筑工程质量。

（二十四）建立使用者监督机制。编制绿色住宅购房人验房指南，鼓励将住宅绿色性能和全装修质量相关指标纳入商品房买卖合同、住宅质量保证书和住宅使用说明书，明确质量保修责任和纠纷处理方式，保障购房人权益。

六、强化科技支撑

（二十五）培育科技创新基地。组建一批新型建筑工业化技术创新中心、重点实验室等创新基地，鼓励骨干企业、高等院校、科研院所等联合建立新型建筑工业化产业技术创新联盟。

（二十六）加大科技研发力度。大力支持BIM底层平台软件的研发，加大钢结构住宅在围护体系、材料性能、连接工艺等方面的联合攻关，加快装配式混凝土结构灌浆质量检测和高效连接技术研发，加强建筑机器人等智能建造技术产品研发。

（二十七）推动科技成果转化。建立新型建筑工业化重大科技成果库，加大科技成果公开，促进科技成果转化应用，推动建筑领域新技术、新材料、新产品、新工艺创新发展。

七、加快专业人才培育

（二十八）培育专业技术管理人才。大力培养新型建筑工业化专业人才，壮大设计、生产、施工、管理等方面人才队伍，加强新型建筑工业化专业技术人员继续教育，鼓励企业建立首席信息官（CIO）制度。

（二十九）培育技能型产业工人。深化建筑用工制度改革，完善建筑业从业人员技能水平评价体系，促进学历证书与职业技能等级证书融通衔接。打通建筑工人职业化发展道路，弘扬工匠精神，加强职业技能培训，大力培育产业工人队伍。

（三十）加大后备人才培养。推动新型建筑工业化相关企业开展校企合作，支持校企共建一批现代产业学院，支持院校对接建筑行业发展新需求、新业态、新技术，开设装配式建筑相关课程，创新人才培养模式，提供专业人才保障。

八、开展新型建筑工业化项目评价

（三十一）制定评价标准。建立新型建筑工业化项目评价技术指标体系，重点突出信息化技术应用情况，引领建筑工程项目不断提高劳动生产率和建筑品质。

（三十二）建立评价结果应用机制。鼓励新型建筑工业化项目单位在项目竣工后，按照评价标准开展自评价或委托第三方评价，积极探索区域性新型建筑工业化系统评价，评价结果可作为奖励政策重要参考。

九、加大政策扶持力度

（三十三）强化项目落地。各地住房和城乡建设部门要会同有关部门组织编制新型建筑工业化专项规划和年度发展计划，明确发展目标、重点任务和具体实施范围。要加大推进力度，在项目立项、项目审批、项目管理各环节明确新型建筑工业化的鼓励性措施。政府投资工程要带头按照新型建筑工业化方式建设，鼓励支持社会投资项目采用新型建筑工业化方式。

（三十四）加大金融扶持。支持新型建筑工业化企业通过发行企业债券、公司债券等方式开展融资。完善绿色金融支持新型建筑工业化的政策环境，积极探索多元化绿色金融支持方式，对达到绿色建筑星级标准的新型建筑工业化项目给予绿色金融支持。用好国家绿色发展基金，在不新增隐性债务的前提下鼓励各地设立专项基金。

（三十五）加大环保政策支持。支持施工企业做好环境影响评价和监测，在重污染天气期间，装配式等新型建筑工业化项目在非土石方作业的施工环节可以不停工。建立建筑垃圾排放限额标准，开展施工现场建筑垃圾排放公示，鼓励各地对施工现场达到建筑垃圾减量化要求的施工企业给予奖励。

（三十六）加强科技推广支持。推动国家重点研发计划和科研项目支持新型建筑工业化技术研发，鼓励各地优先将新型建筑工业化相关技术纳入住房和城乡建设领域推广应用技术公告和科技成果推广目录。

（三十七）加大评奖评优政策支持。将城市新型建筑工业化发展水平纳入中国人居环境奖评选、国家生态园林城市评估指标体系。大力支持新型建筑工业化项目参与绿色建筑创新奖评选。

中华人民共和国住房和城乡建设部
中华人民共和国教育部
中华人民共和国科学技术部
中华人民共和国工业和信息化部
中华人民共和国自然资源部
中华人民共和国生态环境部
中国人民银行
国家市场监督管理总局
中国银行保险监督管理委员会
2020年8月28日

**P18**

**Guidance on Expanding Investment in Strategic Emerging Industries to Nurture New Growth Points and Poles**

**关于扩大战略性新兴产业投资培育壮大新增长点增长极的指导意见**

发改高技〔2020〕1409号

国务院有关部门，各省、自治区、直辖市、新疆生产建设兵团发展改革委、科技厅（委、局）、工业和信息化委（厅）、财政厅（局）：
  为深入贯彻落实党中央、国务院关于在常态化疫情防控中扎实做好“六稳”工作，全面落实“六保”任务，扩大战略性新兴产业投资、培育壮大新的增长点增长极的决策部署，更好发挥战略性新兴产业重要引擎作用，加快构建现代化产业体系，推动经济高质量发展，现提出如下意见：
  一、总体要求
  以习近平新时代中国特色社会主义思想为指导，全面贯彻党的十九大和十九届二中、三中、四中全会精神，统筹做好疫情防控和经济社会发展工作，坚定不移贯彻新发展理念，围绕重点产业链、龙头企业、重大投资项目，加强要素保障，促进上下游、产供销、大中小企业协同，加快推动战略性新兴产业高质量发展，培育壮大经济发展新动能。
  ——聚焦重点产业领域。着力扬优势、补短板、强弱项，加快适应、引领、创造新需求，推动重点产业领域形成规模效应。
  ——打造集聚发展高地。充分发挥产业集群要素资源集聚、产业协同高效、产业生态完备等优势，利用好自由贸易试验区、自由贸易港等开放平台，促进形成新的区域增长极。
  ——增强要素保障能力。按照“资金跟着项目走、要素跟着项目走”原则，引导人才、用地、用能等要素合理配置、有效集聚。
  ——优化投资服务环境。通过优化营商环境、加大财政金融支持、创新投资模式，畅通供需对接渠道，释放市场活力和投资潜力。
  二、聚焦重点产业投资领域
  （一）加快新一代信息技术产业提质增效。加大5G建设投资，加快5G商用发展步伐，将各级政府机关、企事业单位、公共机构优先向基站建设开放，研究推动将5G基站纳入商业楼宇、居民住宅建设规范。加快基础材料、关键芯片、高端元器件、新型显示器件、关键软件等核心技术攻关，大力推动重点工程和重大项目建设，积极扩大合理有效投资。稳步推进工业互联网、人工智能、物联网、车联网、大数据、云计算、区块链等技术集成创新和融合应用。加快推进基于信息化、数字化、智能化的新型城市基础设施建设。围绕智慧广电、媒体融合、5G广播、智慧水利、智慧港口、智慧物流、智慧市政、智慧社区、智慧家政、智慧旅游、在线消费、在线教育、医疗健康等成长潜力大的新兴方向，实施中小企业数字化赋能专项行动，推动中小微企业“上云用数赋智”，培育形成一批支柱性产业。实施数字乡村发展战略，加快补全农村互联网基础设施短板，加强数字乡村产业体系建设，鼓励开发满足农民生产生活需求的信息化产品和应用，发展农村互联网新业态新模式。实施“互联网+”农产品出村进城工程，推进农业农村大数据中心和重要农产品全产业链大数据建设，加快农业全产业链的数字化转型。（责任部门：发展改革委、工业和信息化部、科技部、教育部、住房城乡建设部、交通运输部、水利部、农业农村部、商务部、卫生健康委、广电总局、国铁集团等按职责分工负责）
  （二）加快生物产业创新发展步伐。加快推动创新疫苗、体外诊断与检测试剂、抗体药物等产业重大工程和项目落实落地，鼓励疫苗品种及工艺升级换代。系统规划国家生物安全风险防控和治理体系建设，加大生物安全与应急领域投资，加强国家生物制品检验检定创新平台建设，支持遗传细胞与遗传育种技术研发中心、合成生物技术创新中心、生物药技术创新中心建设，促进生物技术健康发展。改革完善中药审评审批机制，促进中药新药研发和产业发展。实施生物技术惠民工程，为自主创新药品、医疗装备等产品创造市场。（责任部门：发展改革委、卫生健康委、科技部、工业和信息化部、中医药局、药监局等按职责分工负责）
  （三）加快高端装备制造产业补短板。重点支持工业机器人、建筑、医疗等特种机器人、高端仪器仪表、轨道交通装备、高档五轴数控机床、节能异步牵引电动机、高端医疗装备和制药装备、航空航天装备、海洋工程装备及高技术船舶等高端装备生产，实施智能制造、智能建造试点示范。研发推广城市市政基础设施运维、农业生产专用传感器、智能装备、自动化系统和管理平台，建设一批创新中心和示范基地、试点县。鼓励龙头企业建设“互联网+”协同制造示范工厂，建立高标准工业互联网平台。（责任部门：发展改革委、工业和信息化部、住房城乡建设部、农业农村部、国铁集团等按职责分工负责）
  （四）加快新材料产业强弱项。围绕保障大飞机、微电子制造、深海采矿等重点领域产业链供应链稳定，加快在光刻胶、高纯靶材、高温合金、高性能纤维材料、高强高导耐热材料、耐腐蚀材料、大尺寸硅片、电子封装材料等领域实现突破。实施新材料创新发展行动计划，提升稀土、钒钛、钨钼、锂、铷铯、石墨等特色资源在开采、冶炼、深加工等环节的技术水平，加快拓展石墨烯、纳米材料等在光电子、航空装备、新能源、生物医药等领域的应用。（责任部门：发展改革委、工业和信息化部等按职责分工负责）
  （五）加快新能源产业跨越式发展。聚焦新能源装备制造“卡脖子”问题，加快主轴承、IGBT、控制系统、高压直流海底电缆等核心技术部件研发。加快突破风光水储互补、先进燃料电池、高效储能与海洋能发电等新能源电力技术瓶颈，建设智能电网、微电网、分布式能源、新型储能、制氢加氢设施、燃料电池系统等基础设施网络。提升先进燃煤发电、核能、非常规油气勘探开发等基础设施网络的数字化、智能化水平。大力开展综合能源服务，推动源网荷储协同互动，有条件的地区开展秸秆能源化利用。（责任部门：发展改革委、工业和信息化部、自然资源部、能源局等按职责分工负责）
  （六）加快智能及新能源汽车产业基础支撑能力建设。开展公共领域车辆全面电动化城市示范，提高城市公交、出租、环卫、城市物流配送等领域车辆电动化比例。加快新能源汽车充/换电站建设，提升高速公路服务区和公共停车位的快速充/换电站覆盖率。实施智能网联汽车道路测试和示范应用，加大车联网车路协同基础设施建设力度，加快智能汽车特定场景应用和产业化发展。支持建设一批自动驾驶运营大数据中心。以支撑智能汽车应用和改善出行为切入点，建设城市道路、建筑、公共设施融合感知体系，打造基于城市信息模型（CIM）、融合城市动态和静态数据于一体的“车城网”平台，推动智能汽车与智慧城市协同发展。（责任部门：发展改革委、工业和信息化部、住房城乡建设部、交通运输部等按职责分工负责）
  （七）加快节能环保产业试点示范。实施城市绿色发展综合示范工程，支持有条件的地区结合城市更新和城镇老旧小区改造，开展城市生态环境改善和小区内建筑节能节水改造及相关设施改造提升，推广节水效益分享等合同节水管理典型模式，鼓励创新发展合同节水管理商业模式，推动节水服务产业发展。开展共用物流集装化体系示范，实现仓储物流标准化周转箱高效循环利用。组织开展多式联运示范工程建设。发展智慧农业，推进农业生产环境自动监测、生产过程智能管理。试点在超大城市建立基于人工智能与区块链技术的生态环境新型治理体系。探索开展环境综合治理托管、生态环境导向的开发（EOD）模式等环境治理模式创新，提升环境治理服务水平，推动环保产业持续发展。加大节能、节水环保装备产业和海水淡化产业培育力度，加快先进技术装备示范和推广应用。实施绿色消费示范，鼓励绿色出行、绿色商场、绿色饭店、绿色电商等绿色流通主体加快发展。积极推行绿色建造，加快推动智能建造与建筑工业化协同发展，大力发展钢结构建筑，提高资源利用效率，大幅降低能耗、物耗和水耗水平。（责任部门：发展改革委、科技部、工业和信息化部、自然资源部、生态环境部、住房和城乡建设部、交通运输部、农业农村部、商务部、国铁集团等按职责分工负责）
  （八）加快数字创意产业融合发展。鼓励数字创意产业与生产制造、文化教育、旅游体育、健康医疗与养老、智慧农业等领域融合发展，激发市场消费活力。建设一批数字创意产业集群，加强数字内容供给和技术装备研发平台，打造高水平直播和短视频基地、一流电竞中心、高沉浸式产品体验展示中心，提供VR旅游、AR营销、数字文博馆、创意设计、智慧广电、智能体育等多元化消费体验。发展高清电视、超高清电视和5G高新视频，发挥网络视听平台和产业园区融合集聚作用，贯通内容生产传播价值链和电子信息设备产业链，联动线上线下文化娱乐和综合信息消费，构建新时代大视听全产业链市场发展格局。（责任部门：发展改革委、教育部、工业和信息化部、农业农村部、文化和旅游部、广电总局、体育总局等按职责分工负责）
  三、打造产业集聚发展新高地
  （九）深入推进国家战略性新兴产业集群发展工程。构建产业集群梯次发展体系，培育和打造10个具有全球影响力的战略性新兴产业基地、100个具备国际竞争力的战略性新兴产业集群，引导和储备1000个各具特色的战略性新兴产业生态，形成分工明确、相互衔接的发展格局。适时启动新一批国家战略性新兴产业集群建设。培育若干世界级先进制造业集群。综合运用财政、土地、金融、科技、人才、知识产权等政策，协同支持产业集群建设、领军企业培育、关键技术研发和人才培养等项目。（责任部门：发展改革委、科技部、工业和信息化部、财政部、人力资源社会保障部、自然资源部、商务部、人民银行、知识产权局等按职责分工负责）
  （十）增强产业集群创新引领力。启动实施产业集群创新能力提升工程。发挥科技创新中心、综合性国家科学中心创新资源丰富的优势，推动特色产业集群发展壮大。依托集群内优势产学研单位联合建设一批产业创新中心、工程研究中心、产业计量测试中心、质检中心、企业技术中心、标准创新基地、技术创新中心、制造业创新中心、产业知识产权运营中心等创新平台和重点地区承接产业转移平台。推动产业链关键环节企业建设产业集群协同创新中心和产业研究院。（责任部门：发展改革委、科技部、工业和信息化部、市场监管总局、中科院、知识产权局等按职责分工负责）
  （十一）推进产城深度融合。启动实施产业集群产城融合示范工程。以产业集群建设推动生产、生活、生态融合发展，促进加快形成创新引领、要素富集、空间集约、宜居宜业的产业生态综合体。加快产业集群交通、物流、生态环保、水利等基础设施数字化改造。推进产业集群资源环境设施共建共享、能源资源智能利用、污染物集中处理等设施建设。探索“核心承载区管理机构+投资建设公司+专业运营公司”建设新模式，推进核心承载区加快向企业综合服务、产业链资源整合、价值再造平台转型。推动符合条件的战略性新兴产业集群通过市场化方式开展基础设施领域不动产投资信托基金（REITs）试点。（责任部门：发展改革委、住房城乡建设部、交通运输部、水利部、证监会、国铁集团等按职责分工负责）
  （十二）聚焦产业集群应用场景营造。启动实施产业集群应用场景建设工程。围绕5G、人工智能、车联网、大数据、区块链、工业互联网等领域，率先在具备条件的集群内试点建设一批应用场景示范工程，定期面向特定市场主体发布应用场景项目清单，择优评选若干新兴产业应用场景进行示范推广，并给予应用方一定支持。鼓励集群内企业发展面向定制化应用场景的“产品+服务”模式，创新自主知识产权产品推广应用方式和可再生能源综合应用，壮大国内产业循环。（责任部门：发展改革委、工业和信息化部、住房城乡建设部、能源局、知识产权局等按职责分工负责）
  （十三）提高产业集群公共服务能力。实施产业集群公共服务能力提升工程。依托行业协会、专业机构、科研单位等建设一批专业化产业集群促进机构。推进国家标准参考数据体系建设。建设产业集群创新和公共服务综合体，强化研发设计、计量测试、标准认证、中试验证、检验检测、智能制造、产业互联网、创新转化等产业公共服务平台支撑，打造集技术转移、产业加速、孵化转化等为一体的高品质产业空间。在智能制造、绿色制造、工业互联网等领域培育一批解决方案供应商。支持有条件的集群聚焦新兴应用开展5G、数据中心、人工智能、工业互联网、车联网、物联网等新型基础设施建设。（责任部门：发展改革委、工业和信息化部、住房城乡建设部、商务部、市场监管总局、中科院等按职责分工负责）
  四、增强资金保障能力
  （十四）加强政府资金引导。统筹用好各级各类政府资金、创业投资和政府出资产业投资基金，创新政府资金支持方式，强化对战略性新兴产业重大工程项目的投资牵引作用。鼓励地方政府设立战略性新兴产业专项资金计划，按市场化方式引导带动社会资本设立产业投资基金。围绕保障重点领域产业链供应链稳定，鼓励建立中小微企业信贷风险补偿机制，加大对战略性新兴产业的支持力度。（责任部门：发展改革委、工业和信息化部、财政部等按职责分工负责）
  （十五）提升金融服务水平。鼓励金融机构创新开发适应战略性新兴产业特点的金融产品和服务，加大对产业链核心企业的支持力度，优化产业链上下游企业金融服务，完善内部考核和风险控制机制。鼓励银行探索建立新兴产业金融服务中心或事业部。推动政银企合作。构建保险等中长期资金投资战略性新兴产业的有效机制。制订战略性新兴产业上市公司分类指引，优化发行上市制度，加大科创板等对战略性新兴产业的支持力度。加大战略性新兴产业企业（公司）债券发行力度。支持创业投资、私募基金等投资战略性新兴产业。（责任部门：人民银行、银保监会、证监会、发展改革委等按职责分工负责）
  （十六）推进市场主体投资。依托国有企业主业优势，优化国有经济布局和结构，加大战略性新兴产业投资布局力度。鼓励具备条件的各类所有制企业独立或联合承担国家各类战略性新兴产业研发、创新能力和产业化等建设项目。支持各类所有制企业发挥各自优势，加强在战略性新兴产业领域合作，促进大中小企业融通发展。修订外商投资准入负面清单和鼓励外商投资产业目录，进一步放宽或取消外商投资限制，增加战略性新兴产业条目。（责任部门：发展改革委、工业和信息化部、商务部、国资委等职责分工负责）
  五、优化投资服务环境
  （十七）深化“放管服”改革。全力推动重大项目“物流通、资金通、人员通、政策通”。深化投资审批制度改革，推进战略性新兴产业投资项目承诺制审批，简化、整合项目报建手续，深化投资项目在线审批监管平台应用，加快推进全程网办。全面梳理新产业、新业态、新模式准入和行政许可流程，精简审批环节，缩短办理时限，推行“一网通办”。（责任部门：发展改革委牵头，各部门按职责分工负责）
  （十八）加快要素市场化配置。充分发挥市场在资源配置中的决定性作用，更好发挥政府作用。统筹做好用地、用水、用能、环保等要素配置，将土地林地、建筑用砂、能耗等指标优先保障符合高质量发展要求的重大工程和项目需求。加强工业用地市场化配置，鼓励地方盘活利用存量土地。（责任部门：发展改革委、自然资源部、生态环境部、住房城乡建设部、水利部、商务部等按职责分工负责）
  （十九）完善包容审慎监管。推动建立适应新业态新模式发展特点、以信用为基础的新型监管机制。规范行政执法行为，推进跨部门联合“双随机、一公开”监管和“互联网+监管”，细化量化行政处罚标准。（责任部门：发展改革委牵头，各部门按职责分工负责）
  （二十）营造良好投资氛围。各地区、各部门要积极做好政策咨询和宣传引导工作，以“线上线下”产业招商会、优质项目遴选赛、政银企对接会、高端论坛等形式加强交流合作，增强企业投资意愿，激发社会投资创新动力和发展活力，努力营造全社会敢投资、愿投资、善投资战略性新兴产业发展的良好氛围。（责任部门：发展改革委牵头，各部门按职责分工负责）

国家发展改革委

科  技  部

工业和信息化部

财  政  部

2020年9月8日

**P19**

**Notice on Agreeing to Carry Out Intelligent Construction Pilot Projects**

**住房和城乡建设部办公厅关于同意开展智能建造试点的函**

建办市函〔2021〕55号

上海市、重庆市住房和城乡建设（管）委，广东省住房和城乡建设厅：

　　《关于金地集团上海嘉定新城菊园社区项目申请作为住建部智能建造与建筑工业化协同发展试点项目的函》、《关于推荐佛山顺德凤桐花园等项目为国家智能建造试点项目的函》（粤建市函〔2021〕56号）、《关于推荐重庆美好天赋等三个项目为住房和城乡建设部智能建造试点项目的请示》（渝建函〔2021〕71号）收悉。经研究，现函复如下：

　　一、同意将上海嘉定新城菊园社区JDC1－0402单元05－02地块项目、佛山顺德凤桐花园项目、佛山顺德北滘镇南坪路以西地块之一项目、深圳市长圳公共住房及其附属工程总承包（EPC）项目和重庆美好天赋项目、绿地新里秋月台项目、万科四季花城三期项目列为我部智能建造试点项目。

　　二、开展智能建造试点工作，要深入贯彻落实《住房和城乡建设部等部门关于推动智能建造与建筑工业化协同发展的指导意见》（建市〔2020〕60号），围绕建筑业高质量发展，以数字化、智能化升级为动力，创新突破相关核心技术，加大智能建造在工程建设各环节应用，提升工程质量安全、效益和品质，尽快探索出一套可复制可推广的智能建造发展模式和实施经验。

　　三、你委（厅）要切实加强组织领导，完善工作机制，落实工作责任，督促有关单位按照试点方案明确的试点目标和重点任务，抓紧推进试点工作，及时总结经验做法。试点中有关情况和问题，请及时与我部建筑市场监管司联系。

　　　　　　　　　　　　　　　　　　　　　　　　　　　　　　　　　　住房和城乡建设部办公厅
2021年2月2日

**P20**

**Green Construction Technology Guidelines (Trial)**

**绿色建造技术导则（试行）**

1 总则

1.0.1 为贯彻落实绿色发展理念，推进绿色建造，节约资 源，保护环境，减少排放，提升建筑工程品质，推动建筑业

高质量发展，制定本导则。

1.0.2 本导则适用于新建民用建筑、工业建筑及其相关附

属设施的绿色建造，既有建筑的改建或扩建可参照执行。

1.0.3 绿色建造应将绿色发展理念融入工程策划、设计、 施工、交付的建造全过程，充分体现绿色化、工业化、信息

化、集约化和产业化的总体特征。

1.0.4 绿色建造除应符合本导则的规定外， 尚应符合国家

现行有关标准的规定。

2 术语

2.0.1 绿色建造 green construction

按照绿色发展的要求，通过科学管理和技术创新，采用 有利于节约资源、保护环境、减少排放、提高效率、保障品

质的建造方式，实现人与自然和谐共生的工程建造活动。

2.0.2 绿色策划 green planning

因地制宜对建造全过程、全要素进行统筹，科学确定绿

色建造目标及实施路径的工程策划活动。

2.0.3 绿色设计 green design

贯彻绿色建造理念，落实绿色策划目标的工程设计活

动。

2.0.4 绿色建材 green building material

在全寿命期内可减少对资源的消耗和对生态环境的影 响，具有节能、减排、安全、健康、便利、可循环等特征的

建材产品。

2.0.5 绿色施工 green construction operation

在保证工程质量、施工安全等基本要求的前提下，以人 为本，因地制宜，通过科学管理和技术进步，最大限度地节

约资源，减少对环境负面影响的施工及生产活动。

2.0.6 智慧工地 smart construction site

综合采用各类信息技术， 围绕人员、机械设备、材料、

方法、环境等施工现场关键要素，具备信息实时采集、互通

共享、工作协同、智能决策分析、风险预控等功能的数字化

施工管理模式。

2.0.7 绿色交付 green delivery

在综合效能调适、绿色建造效果评估的基础上，制定交 付策略、交付标准、交付方案，采用实体与数字化同步交付

的方式，进行工程移交和验收的活动。

2.0.8 建筑信息模型 building information model

在建筑工程及设施全寿命期内，对其物理和功能特性进 行数字化表达，并依此设计、施工、运营的过程和结果的总

称，简称模型或 BIM。

3 基本规定

3.0.1 绿色建造应统筹考虑建筑工程质量、安全、效率、 环保、生态等要素，实现工程策划、设计、施工、交付全过

程一体化，提高建造水平和建筑品质。

3.0.2 绿色建造应全面体现绿色要求，有效降低建造全过 程对资源的消耗和对生态环境的影响，减少碳排放，整体提

升建造活动绿色化水平。

3.0.3 绿色建造宜采用系统化集成设计、精益化生产施工、 一体化装修的方式，加强新技术推广应用，整体提升建造方

式工业化水平。

3.0.4 绿色建造宜结合实际需求，有效采用 BIM、物联网、 大数据、云计算、移动通信、区块链、人工智能、机器人等

相关技术，整体提升建造手段信息化水平。

3.0.5 绿色建造宜采用工程总承包、全过程工程咨询等组 织管理方式，促进设计、生产、施工深度协同，整体提升建

造管理集约化水平。

3.0.6 绿色建造宜加强设计、生产、施工、运营全产业链 上下游企业间的沟通合作，强化专业分工和社会协作，优化 资源配置，构建绿色建造产业链，整体提升建造过程产业化

水平。

4 绿色策划

4.1 一般规定

4.1.1 建设单位应在建筑工程立项阶段组织编制项目绿色 策划方案，项目各参与方应遵照执行。

4.1.2 绿色策划方案应明确绿色建造总体目标和资源节 约、环境保护、减少碳排放、品质提升、职业健康安全等分 项目标，应包括绿色设计策划、绿色施工策划、绿色交付策 划等内容。

4.1.3 绿色策划方案应因地制宜对建造全过程、全要素进 行统筹，明确绿色建造实施路径，体现绿色化、工业化、信 息化、集约化和产业化特征。

4.1.4 绿色策划方案应确定项目定位和组织架构，明确各 阶段的主要控制指标，进行综合成本与效益分析，制定主要 工作计划。

4.1.5 绿色策划方案应统筹设计、构件部品部件生产运输、 施工安装和运营维护管理，推进产业链上下游资源共享、系 统集成和联动发展。

4.1.6 绿色策划宜制定合理的减排方案，建立碳排放管理 体系，并应明确建筑垃圾减量化等目标。

4.1.7 绿色策划宜推动全过程数字化、 网络化、智能化技 术应用，积极采用 BIM 技术，利用基于统一数据及接口标准

的信息管理平台，支撑各参与方、各阶段的信息共享与传递。 4.1.8 绿色策划宜结合工程实际情况，综合考虑技术水平、 成本投入与效益产出等因素，确定智能建造、新型建筑工业

化的应用目标和实施路径。

4.2 绿色设计策划

4.2.1 应根据绿色建造目标，结合项目定位，在综合技术 经济可行性分析基础上，确定绿色设计目标与实施路径，明 确主要绿色设计指标和技术措施。

4.2.2 应推进建筑、结构、机电设备、装饰装修等专业的 系统化集成设计。

4.2.3 应以保障性能综合最优为目标，对场地、建筑空间、 室内环境、建筑设备进行全面统筹。

4.2.4 应明确绿色建材选用依据、总体技术性能指标，确 定绿色建材的使用率。

4.2.5 应综合考虑生产、施工的便易性，提出全过程、全 专业、各参与方之间的一体化协同设计要求。

4.3 绿色施工策划

4.3.1 应结合施工现场及周边环境、工程实际情况等进行 影响因素分析和环境风险评估，并依据分析和评估结果进行 绿色施工策划。

4.3.2 应按照现行国家标准《建筑工程绿色施工评价标准》 GB/T 50640 中的优良级别，明确项目绿色施工关键指标。

4.3.3 应对生态环境保护、资源节约与循环利用、碳排放 降低、人力资源节约及职业健康安全等进行总体分析，策划

适宜的绿色施工技术路径与措施。

4.4 绿色交付策划

4.4.1 应根据建筑类型和运营维护需求确定绿色建造项目 的实体交付内容及交付标准。

4.4.2 宜按照城市信息化建设要求和运营维护需求，制定 数字化交付标准和方案，明确各阶段责任主体和交付成果。

4.4.3 应明确综合效能调适及绿色建造效果评估的内容及

方式。

5 绿色设计

5.1 一般规定

5.1.1 应统筹建筑、结构、机电设备、装饰装修、景观园 林等各专业设计，统筹策划、设计、施工、交付等建造全过 程，实现工程全寿命期系统化集成设计。

5.1.2 宜应用 BIM 等数字化设计方式，实现设计协同、设 计优化。

5.1.3 应优先就地取材，并统筹确定各类建材及设备的设 计使用年限。

5.1.4 应强化设计方案技术论证，严格控制设计变更。设 计变更不应降低工程绿色性能，重大变更应组织专家对其是 否影响工程绿色性能进行论证。

5.1.5 应在设计阶段加强建筑垃圾源头管控，按照《住房 和城乡建设部关于推进建筑垃圾减量化的指导意见》（建质 〔2020〕46 号）的有关规定进行设计。

5.2 设计要求

5.2.1 场地设计应有效利用地域自然条件，尊重城市肌理 和地域风貌，实现建筑布局、交通组织、场地环境、场地设 施和管网的合理设计。

5.2.2 应按照“被动式技术优先、主动式技术优化 ”的原 则，优化功能空间布局，充分发掘场地空间、建筑本体与设

备在节约资源方面的潜力。

5.2.3 应综合考虑安全耐久、节能减排、易于建造等因素， 择优选择建筑形体和结构体系。

5.2.4 应根据建筑规模、用途、能源条件以及国家和地区 节能环保政策对冷热源方案进行综合论证，合理利用浅层地 能、太阳能、风能等可再生能源以及余热资源。

5.2.5 应体现海绵城市建设理念，采用“渗、滞、蓄、净、 用、排 ”等措施对施工期间及建筑竣工后的场地雨水进行有 效统筹控制，溢流排放应与城市雨水排放系统衔接。

5.2.6 应优先采用管线分离、一体化装修技术，对建筑围 护结构和内外装饰装修构造节点进行精细设计。

5.2.7 宜采用标准化构件和部件，使用集成化模块化建筑 部品，提高工程品质，降低运行维护成本。

5.3 协同设计

5.3.1 应建立涵盖设计、生产、施工等不同阶段的协同设 计机制，实现生产、施工、运营维护各方的前置参与，统筹 管理项目方案设计、初步设计、施工图设计。

5.3.2 宜采用协同设计平台，集成技术措施、产品性能清 单、成本数据库等，实现全过程、全专业、各参与方的协同 设计。

5.3.3 应按照标准化、模块化原则对空间、构件和部品进 行协同深化设计，实现建筑构配件与设备和部品之间模数协

调统一。

5.3.4 宜实现部品部件、 内外装饰装修、 围护结构和机电

管线等一体化集成。

5.4 数字设计

5.4.1 宜采用 BIM 正向设计，优化设计流程，支撑不同专 业间以及设计与生产、施工的数据交换和信息共享。

5.4.2 宜集成应用 BIM、地理信息系统（GIS）、三维测量 等信息技术及模拟分析软件，进行性能模拟分析、设计优化 和阶段成果交付。

5.4.3 应统一设计过程中 BIM 组织方式、工作界面、模型 细度和样板文件。

5.4.4 宜采用 BIM 信息平台，支撑 BIM 模型存储与集成、 版本控制，保障数据安全。

5.4.5 应在设计过程中积累可重复利用及标准化部品构 件，丰富和完善 BIM 构件库资源。

5.4.6 宜推进 BIM 与项目、企业管理信息系统的集成应用， 推动 BIM 与城市信息模型（CIM）平台以及建筑产业互联网 的融通联动。

5.5 材料选用

5.5.1 建筑材料的选用应符合下列规定：

1 应符合国家和地方相关标准规范环保要求；

2 宜优先选用获得绿色建材评价认证标识的建筑材料

和产品；

3 宜优先采用高强、高性能材料；

4 宜选择地方性建筑材料和当地推广使用的建筑材

料。

5.5.2 建筑结构材料应优先选用高耐久性混凝土、耐候和 耐火结构钢、耐久木材等。

5.5.3 外饰面材料、室内装饰装修材料、防水和密封材料 等应选用耐久性好、易维护的材料。

5.5.4 应合理选用可再循环材料、可再利用材料，宜选用 以废弃物为原料生产的利废建材。

5.5.5 建筑门窗、幕墙、 围栏及其配件的力学性能、热工 性能和耐久性等应符合相应产品标准规定，并应满足设计使 用年限要求。

5.5.6 管材、管线、管件应选用耐腐蚀、抗老化、耐久性 能好的材料，活动配件应选用长寿命产品，并应考虑部品之 间合理的寿命匹配性。不同使用寿命的部品组合时，构造宜 便于分别拆换、更新和升级。

5.5.7 建筑装修宜优先采用装配式装修，选用集成厨卫等 工业化内装部品。

6 绿色施工

6.1 一般规定

6.1.1 绿色施工应符合现行国家标准《建筑工程绿色施工 规范》GB/T 50905 和《建筑工程绿色施工评价标准》GB/T 50640 的要求。

6.1.2 应根据绿色施工策划进行绿色施工组织设计、绿色 施工方案编制。

6.1.3 应建立与设计、生产、运营维护联动的协同管理机

制。

6.1.4 应积极采用工业化、智能化建造方式，实现工程建 设低消耗、低排放、高质量和高效益。

6.1.5 宜积极运用 BIM、大数据、云计算、物联网以及移动 通讯等信息化技术组织绿色施工，提高施工管理的信息化和 精细化水平。

6.1.6 应建立完善的绿色建材供应链，采用绿色建筑材料、 部品部件等。

6.1.7 应编制施工现场建筑垃圾减量化专项方案，实现建 筑垃圾源头减量、过程控制、循环利用。

6.1.8 鼓励对传统施工工艺进行绿色化升级革新。

6.1.9 应加强绿色施工新技术、新材料、新工艺、新设备 应用，优先采用“建筑业 10 项新技术 ”。

6.1.10 部品部件生产应采用环保生产工艺和设备设施，并 应严格执行质量管理体系、环境管理体系和职业健康安全管 理体系。

6.1.11 部品部件生产应提高数字化、智能化水平，逐步实 现精益生产、智能制造。

6.1.12 应制定消防疏散、卫生防疫、职业健康安全等管理 制度和突发事件应急措施，保障人员身心健康。

6.2 协同与优化

6.2.1 应在项目前期进行设计与施工协同，根据工程实际 情况及施工能力优化设计方案，提高施工机械化、工业化、 信息化水平。

6.2.2 应进行多层级交底，明确绿色设计重点内容、绿色 建材产品使用要求。

6.2.3 应结合加工、运输、安装方案和施工工艺要求，对 工程重点、难点部位和复杂节点等进行深化设计。

6.2.4 在满足设计要求的前提下，应充分考虑施工临时设 施与永久性设施的结合利用，实现永临结合。

6.2.5 部品部件生产应与设计、物流、现场施工进行有效 协同与联动。

6.3 环境保护

6.3.1 应通过信息化手段监测并分析施工现场扬尘、噪声、

光、污水、有害气体、固体废弃物等各类污染物。

6.3.2 应采取措施减少扬尘排放，PM10 和 PM2.5 不得超过当地 生态环境部门或住房和城乡建设主管部门要求的限值。

6.3.3 现场有害气体应经净化处理后排放，排放标准应符 合现行国家标准《环境空气质量标准》GB 3095 和《民用建 筑工程室内环境污染控制标准》GB 50325 的规定。

6.3.4 应采取措施控制噪声和振动污染，噪声限值应满足 现行国家标准《建筑施工场界环境噪声排放标准》GB 12523 的规定，振动限值应符合现行国家标准《城市区域环境振动 标准》GB 10070 的规定。

6.3.5 应采取措施保护施工现场及周边水环境，减少地下 水抽取，避免施工场地的水土污染。

6.3.6 应采取措施减少污水排放。排入城市污水管网的施 工污水应符合现行国家标准《污水排入城镇下水道水质标 准》GB/T 31962 的规定。没有纳管条件的，应处理达到相关 排放标准或收纳水体要求后，方可排放。

6.3.7 应采取措施减少光污染，光污染限值应满足现行行 业标准《城市夜景照明设计规范》JGJ/T 163 的规定。

6.3.8 宜采用装配化施工工艺，建筑内外装修优先采用装 配式装修等干式工法施工工艺及集成厨卫等模块化部品部 件，减少现场切割及湿作业。

6.3.9 应采用先进施工工艺与方法，从源头减少有毒有害 废弃物的产生。对产生的有毒有害废弃物应 100%分类回收、

合规处理。

6.3.10 拆除施工应制定环境保护计划，选择对环境影响小 的拆除工艺。对拆除过程中产生的废水、噪声、扬尘等应采

取针对性防治措施，并制定拆除垃圾处理方案。

6.4 资源节约

6.4.1 应采用精益化施工组织方式，统筹管理施工相关要 素和环节，提升施工现场精细化管理水平，减少资源消耗与 浪费。

6.4.2 应推广使用新型模架体系，提高施工临时设施和周 转材料的工业化程度和周转次数。

6.4.3 部品部件安装应采用与其相匹配的工具化、标准化 工装系统，采用适用的安装工法，制定合理的安装工序，减 少现场支模和脚手架搭建。

6.4.4 应积极推广材料工厂化加工，实现精准下料、精细 管理，降低建筑材料损耗率。

6.4.5 应加强施工设备的进场、安装、使用、维护保养、 拆除及退场管理，减少过程中设备损耗。

6.4.6 应采用节能型设备，监控重点能耗设备的耗能，对 多台同类设备实施群控管理。

6.4.7 应结合工程所在地地域特征，积极利用适宜的可再 生能源。

6.4.8 应因地制宜对施工现场雨水、 中水进行科学收集和

合理利用。

6.4.9 应科学布置施工现场，合理规划临时用地，减少地 面硬化。宜利用再生材料或可周转材料进行临时场地硬化。 6.4.10 应采取措施减少固体废弃物产生，建筑垃圾产生量 应控制在现浇钢筋混凝土结构每万平方米不大于 300 吨，装 配式建筑每万平方米不大于 200 吨（不包括工程渣土、工程

泥浆）。

6.5 信息技术应用

6.5.1 应通过信息技术促进设计、生产、施工、运营维护 等产业链联动，支持项目多参与方协同工作，实现建造全过 程统筹管理。

6.5.2 宜基于 BIM 设计信息，推进工厂生产全流程自动化、 信息化、智能化。

6.5.3 宜采用 BIM 等信息技术进行深化设计和专业协调， 避免“错漏碰缺 ”等问题。对危险性较大和工序复杂的方案 应进行三维模拟和可视化交底。

6.5.4 应根据项目需求和参建单位情况，采用智慧工地管 理系统，实现信息互通共享、工作协同、智能决策分析、风 险预控。

6.5.5 应采用信息通信技术对施工设备的基础信息、进出 场信息和安装信息等进行管理，对塔式起重机、施工升降机 等危险性较大设备的运行数据进行实时采集和监控。

6.5.6 宜采用自动化施工器械、智能移动终端等相关设备， 提升施工质量和效率，降低安全风险。积极推广使用建筑机

器人进行材料搬运、打磨、铺墙地砖、钢筋加工、喷涂、高

空焊接等工作。

7 绿色交付

7.1 一般规定

7.1.1 项目交付前应进行绿色建造的效果评估。

7.1.2 项目交付前应完成绿色建筑相关检测，提交建筑使 用说明书。

7.1.3 应核定绿色建材实际使用率，提交核定计算书。

7.1.4 应将建筑各分部分项工程的设计、施工、检测等技 术资料整合和校验，并按相关标准移交建设单位和运营单 位。

7.1.5 应制定建筑物各子系统（机电设备系统、消防系统 等）运行操作规程和维护保养手册。

7.1.6 应按照绿色交付标准及成果要求提供实体交付及数 字化交付成果。数字化交付成果应保证与实体交付成果信息 的一致性和准确性，建设单位可在交付前组织成果验收。

7.2 交付要求

7.2.1 应对建筑开展综合效能调适，包括夏季工况、冬季 工况及过渡季节工况的调适和性能验证，使建筑机电系统满 足绿色建造目标和实际使用等要求。

7.2.2 应组织相关各方建立综合效能调适团队，明确各方 职责，编制调适方案，制定调适计划。

7.2.3 综合效能调适的内容和要求应符合现行行业标准

《绿色建筑运行维护技术规范》JGJ/T 391 的规定。综合效

能调适完成后，应将相关技术文件存档。

7.2.4 数字化交付的内容及标准应执行工程所在地的相关 规定。当所在地区未规定时，可由建设单位牵头确定，各参 建单位遵照执行。

7.2.5 数字化交付内容应包含数字化工程质量验收文件、 施工影像资料、建筑信息模型等。应编制说明书，详细说明 交付的范围与内容。

7.2.6 建筑信息模型应按单位工程进行划分组建，每个单 位工程包含建筑、结构、给排水、电气、暖通等分专业模型 以及综合模型文件。

7.2.7 应基于构件维护、保养、更换、质量追溯等需求， 为建筑信息模型构件建立编码，并确保构件编码的唯一性。

7.2.8 服务数字化运营维护的建筑信息模型应包含供应商 和维护保养等信息。

7.2.9 数字化交付过程中数据传递应遵守相关保密规定。

7.3 效果评估

7.3.1 应对绿色建造节约资源和保护环境的效果进行评 估，并形成效果评估报告。可采用内部自评的形式，或委托 具备评估能力的技术服务单位进行评估。效果评估应包含但 不限于绿色施工、减排、海绵城市建设等内容。

7.3.2 效果评估的具体内容、参考标准、评估结果以及证

明材料等应进行汇总，形成绿色建造效果评估表。7.3.3 证明材料应包括但不限于设计文件、专项报告、分 析计算报告、现场检测报告等。

7.3.4 进行绿色施工效果评估时，证明材料应包括绿色施 工评价定级报告，评价定级方法应按照现行国家标准《建筑 工程绿色施工评价标准》GB/T 50640 执行。

7.3.5 进行减排效果评估时，证明材料应包括碳排放计算 报告，计算方法应按照现行国家标准《建筑碳排放计算标准》 GB/T 51366 执行。

7.3.6 场地和地块海绵城市建设效果评估，应按照现行国 家标准《海绵城市建设评价标准》GB/T 51345 执行。

住房和城乡建设部

2021 年 3 月

**P21**

**Notice on Issuing a List of Replicable Practices for the Coordinated Development of Intelligent Construction and New Building Industrialization (First Batch)**

**住房和城乡建设部办公厅关于印发智能建造与新型建筑工业化协同发展可复制经验做法清单（第一批）的通知**

建办市函〔2021〕316号

各省、自治区住房和城乡建设厅，直辖市住房和城乡建设（管）委，北京市规划和自然资源委，新疆生产建设兵团住房和城乡建设局：

　　按照《住房和城乡建设部等部门关于推动智能建造与建筑工业化协同发展的指导意见》（建市〔2020〕60号）要求，各地围绕数字设计、智能生产、智能施工等方面积极探索，推动智能建造与新型建筑工业化协同发展取得较大进展。我部总结各地经验做法形成《智能建造与新型建筑工业化协同发展可复制经验做法清单（第一批）》，现印发给你们，请结合实际学习借鉴。

住房和城乡建设部办公厅
2021年7月28日

**P22**

**Three-Year Action Plan for the Construction of New Infrastructure for the Internet of Things (2021-2023)**

**物联网新型基础设施建设三年行动计划（2021-2023年）**

工信部联科〔2021〕130号

物联网是以感知技术和网络通信技术为主要手段，实现人、机、物的泛在连接，提供信息感知、信息传输、信息处理等服务的基础设施。随着经济社会数字化转型和智能升级步伐加快，物联网已经成为新型基础设施的重要组成部分。为贯彻落实《中华人民共和国国民经济和社会发展第十四个五年规划和2035年远景目标纲要》，打造系统完备、高效实用、智能绿色、安全可靠的现代化基础设施体系，推进物联网新型基础设施建设，充分发挥物联网在推动数字经济发展、赋能传统产业转型升级方面的重要作用，制定本计划。

一、总体要求

（一）指导思想

以习近平新时代中国特色社会主义思想为指导，全面贯彻党的十九大和十九届二中、三中、四中、五中全会精神，立足新发展阶段，完整、准确、全面贯彻新发展理念，构建新发展格局，坚持问题导向和需求导向，打造支持固移融合、宽窄结合的物联网接入能力，加速推进全面感知、泛在连接、安全可信的物联网新型基础设施建设，加快技术创新，壮大产业生态，深化重点领域应用，推动物联网全面发展，不断培育经济新增长点，有力支撑制造强国和网络强国建设。

（二）基本原则

聚焦重点，精准突破。聚焦感知、传输、处理、存储、安全等重点环节，加快关键核心技术攻关，提升技术的有效供给；聚焦发展基础好、转型意愿强的重点行业和地区，加快物联网新型基础设施部署，提高物联网应用水平。

需求牵引，强化赋能。以社会治理现代化需求为导向，积极拓展应用场景，提升社会治理与公共服务水平；以产业转型需求为导向，推进物联网与传统产业深度融合，促进产业提质增效；以消费升级需求为导向，推动智能产品的研发与应用，丰富数字生活体验。

统筹协同，汇聚合力。充分发挥地方政府在新型基础设施建设规划、投资布局中的统筹引导作用，形成政策合力；充分发挥科研院所、高校、企业在技术攻关、成果转化中的创新主体作用，形成技术合力；充分发挥市场在资源配置中的决定性作用，调动各方积极性，形成产业合力。

自主创新，安全可靠。统筹发展和安全，提升关键核心技术自主可控水平，加强物联网技术、网络、终端、应用等安全防护能力建设，增强产业链供应链韧性，加强数据安全保护，提升安全可靠运行能力，有效防范化解安全风险隐患。

（三）行动目标

到2023年底，在国内主要城市初步建成物联网新型基础设施，社会现代化治理、产业数字化转型和民生消费升级的基础更加稳固。突破一批制约物联网发展的关键共性技术，培育一批示范带动作用强的物联网建设主体和运营主体，催生一批可复制、可推广、可持续的运营服务模式，导出一批赋能作用显著、综合效益优良的行业应用，构建一套健全完善的物联网标准和安全保障体系。

——创新能力有所突破。高端传感器、物联网芯片、物联网操作系统、新型短距离通信等关键技术水平和市场竞争力显著提升；物联网与5G、人工智能、区块链、大数据、IPv6等技术深度融合应用取得产业化突破；物联网新技术、新产品、新模式不断涌现。

——产业生态不断完善。推动10家物联网企业成长为产值过百亿、能带动中小企业融通发展的龙头企业；支持发展一批专精特新“小巨人”企业；培育若干国家物联网新型工业化产业示范基地，带动物联网产业加速向规模化、集约化、高价值发展。

——应用规模持续扩大。在智慧城市、数字乡村、智能交通、智慧农业、智能制造、智能建造、智慧家居等重点领域，加快部署感知终端、网络和平台，形成一批基于自主创新技术产品、具有大规模推广价值的行业解决方案，有力支撑新型基础设施建设；推进IPv6在物联网领域的大规模应用；物联网连接数突破20亿。

——支撑体系更加健全。完善物联网标准体系，完成40项以上国家标准或行业标准制修订；建立面向网络安全、数据安全、个人信息保护的物联网安全保障体系；建设试验检测、知识产权服务、科技成果转化、人才培养等公共服务平台。

二、重点任务

（一）创新能力提升行动

1.突破关键核心技术。贯通“云、网、端”，围绕信息感知、信息传输、信息处理等产业链关键环节，体系化部署创新链。实施“揭榜挂帅”制度，鼓励和支持骨干企业加大关键核心技术攻关力度，突破智能感知、新型短距离通信、高精度定位等关键共性技术，补齐高端传感器、物联网芯片等产业短板，进一步提升高性能、通用化的物联网感知终端供给能力。

2.推动技术融合创新。面向“5G+物联网”，充分利用5G网络的高可靠、低时延、大连接特点，丰富通信技术供给，拓展物联网应用场景；面向“大数据+物联网”，实现数据高效协同处理，深度挖掘物理世界数据价值；面向“人工智能+物联网”，建立“感知终端+平台+场景”的智能化服务；面向“区块链+物联网”，建立感知终端的信用体系，保障数据确权和价值流通。

3.构建协同创新机制。鼓励地方联合龙头企业、科研院所、高校建立一批物联网技术孵化创新中心，打通科技成果转化链条，推进科技成果中试熟化和工程化应用。鼓励龙头企业联合上下游企业组建物联网产业技术联盟，探索“专利+标准+开源社区”发展模式，激发创新活力。依托基金会、开源社区，聚集开发者和用户资源，共同打造成熟的开源产品和应用解决方案，形成具有国际竞争力协同创新生态。


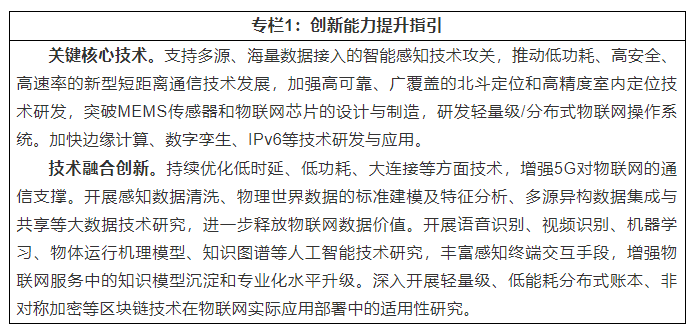


（二）产业生态培育行动

4.培育多元化市场主体。培育一批技术领先、资源整合能力强的龙头企业，深化产学研联合创新，促进创新链，产业链、资金链高效配置，推动感知终端、平台、网络设施的规模化部署。培育一批物联网领域专精特新“小巨人”企业，面向特定场景和细分领域，成为先进技术产品和适用性解决方案供应方。培育一批物联网运营服务商，开展方案设计、集成实施、网络运维、经营管理、网络信息安全防护等服务。

5.加强产业集聚发展。支持产业特色鲜明、基础条件好、应用示范效果突出的地区建设物联网新型工业化产业示范基地，持续发挥现有示范基地的品牌知名度和影响力。加快推动产业集聚发展，做好新产品、新服务、新模式的先行先试，优化政策、人才、技术、资金等资源要素配置。


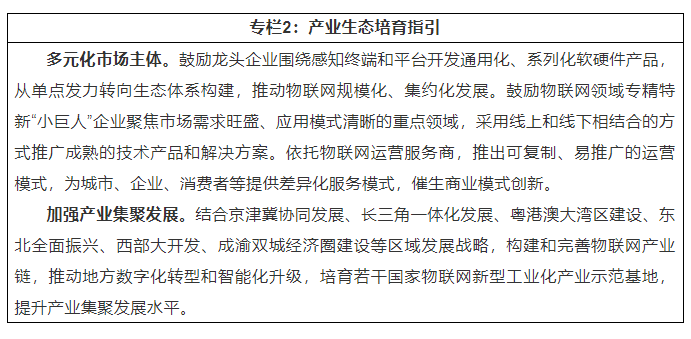


（三）融合应用发展行动

6.社会治理领域。推动交通、能源、市政、卫生健康等传统基础设施的改造升级，将感知终端纳入公共基础设施统一规划建设，打造固移融合、宽窄结合的物联接入能力，搭建综合管理和数据共享平台，充分挖掘多源异构数据价值，推动智慧城市和数字乡村建设，提升社会管理与公共服务的智能化水平。


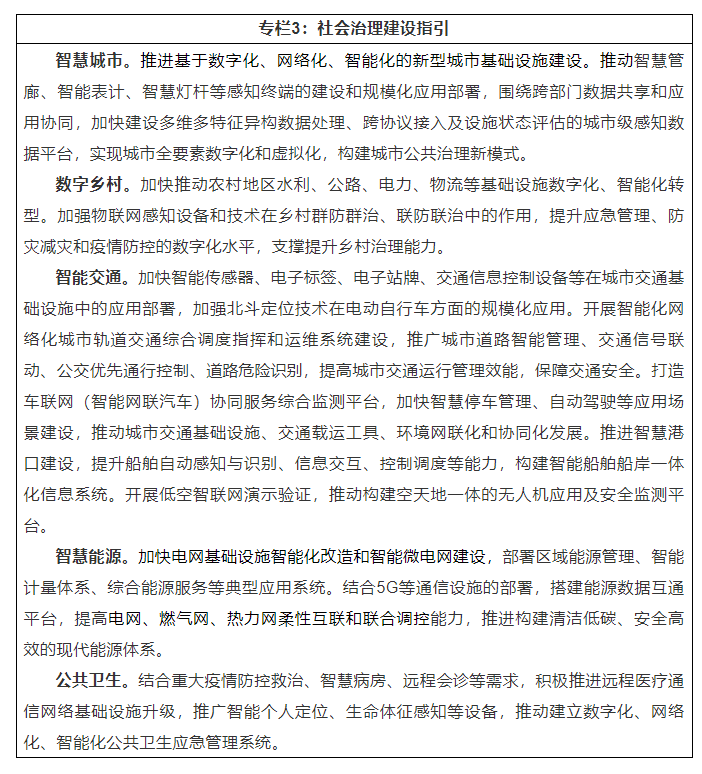


7.行业应用领域。以农业、制造业、建筑业、生态环境、文旅等数字化转型、智能化升级为驱动力，加快数据采集终端、表计、控制器等感知终端应用部署，支持运用新型网络技术改造企业内网和行业专网，建设提供环境监测、信息追溯、状态预警、标识解析等服务的平台，打造一批与行业适配度高的解决方案和应用标杆。


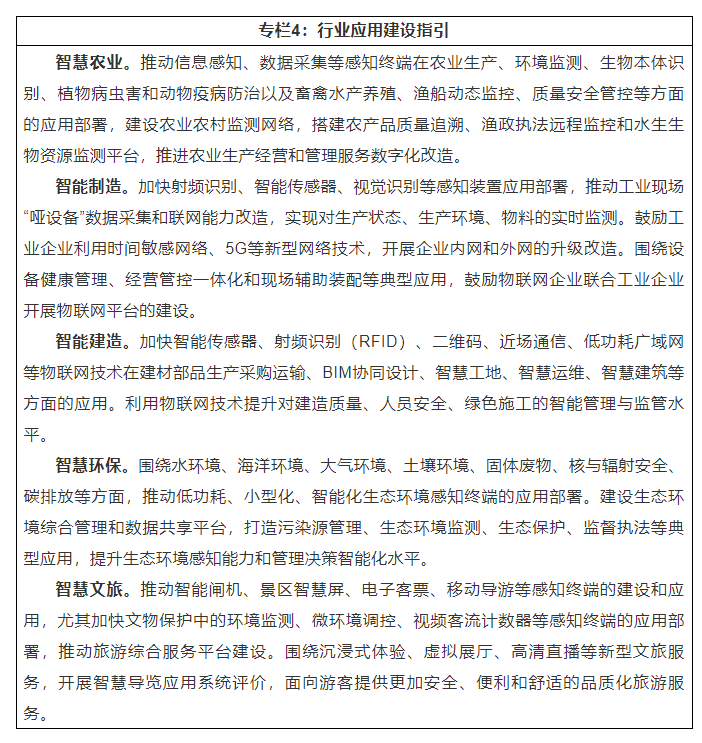


8.民生消费领域。推动感知终端和智能产品在家庭、楼宇、社区的应用部署。打造异构产品互联、集中控制的智慧家庭，建设低碳环保、安全舒适的智慧楼宇和新型社区。鼓励物联网企业与运动器械制造商、康复辅具生产商、养老机构、运动场馆等跨界合作，加快推动可穿戴设备、智能医疗健康产品、智能体育装备等应用普及。


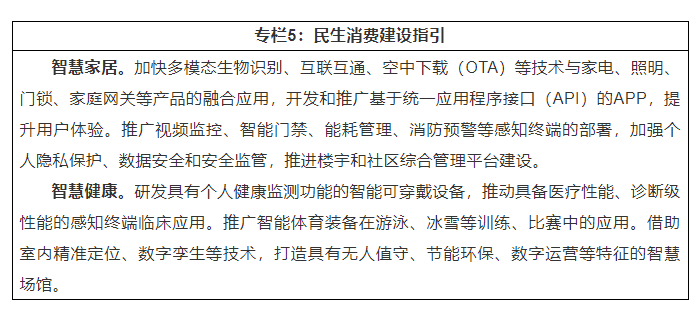


（四）支撑体系优化行动

9.推进IPv6规模应用。完善物联网终端入网检测技术标准与规范，明确IPv6网络接入要求。推进面向公众网络的物联网平台、终端、网关设备等进行IPv6升级改造和使用，推动新产品默认支持并开启IPv6功能。引导和鼓励企业面向行业应用采用基于IPv6的应用解决方案，推广支持IPv6的物联网终端和模组的应用。


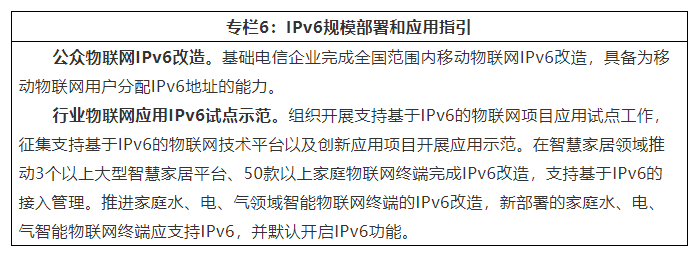


10.加强标准体系建设。优化完善物联网标准体系，建立物联网全产业链标准图谱，加快新技术产品、基础设施建设、行业应用等国家和行业标准制修订，鼓励团体标准先行先试。持续深度参与国际标准化组织（ISO）、国际电信联盟（ITU）、国际电工委员会（IEC）等国际标准化工作，提升我国在国际标准化活动中的贡献度。加强重点标准的实施和评估。

11.完善公共服务体系。支持专业服务机构创新工作思维理念，提升知识产权、科技成果转化、人才培训、投融资等服务能力。搭建技术与标准公共服务平台，开展新技术验证、测试认证、产品质量分级和系统评价等服务。引导地方行业协会、产业园区、科研院所、龙头企业等共同建立资源开放共享平台，开放科研仪器、检测设备、研发能力等资源。

12.强化安全支撑保障。加快围绕感知、接入、传输、数据、应用等安全技术的研究。加快物联网安全监测、预警分析和应对处置技术手段建设，提升感知终端、网络、数据及系统的安全保障水平。加强物联网卡安全管理，推动形成售前风险评估、售时分类登记、售后使用监测的物联网卡全生命周期管理制度。加快物联网领域商用密码技术和产品的应用推广，建设面向物联网领域的密码应用检测平台，提升物联网领域商用密码安全性和应用水平。强化物联网应用场景与频谱资源使用的适配性，保障物联网频率使用安全。依托联盟协会，开展物联网基础安全“百企千款”产品培育计划，建设安全公共服务平台，开展安全能力评估，打造“物联网安心产品”。

三、保障措施

（一）优化协同治理机制。加强部门协同，推动重点任务有效落实。鼓励地方政府结合实际制定针对性强、可操作的政策措施，因地制宜推动物联网产业发展和新型基础设施规划建设。发挥物联网行业协会、产业联盟作用，有效推动产业链上下游需求对接、资源共享。

（二）健全统计和评估机制。完善物联网产业统计体系，为评估考核行动计划成效提供科学依据。持续加强物联网新型工业化产业示范基地质量评价工作，规范“揭榜挂帅”等重点行动的过程管理和第三方评估。

（三）完善人才培养体系。支持和引导普通高等院校、职业院校加大物联网相关学科专业人才培养力度，补齐人才缺口。鼓励企业与院校、科研机构共建实验室和实训基地，增强创新型、应用型、复合型物联网人才供给。推动健全完善物联网人才职业技术技能标准体系。

（四）加大财税金融支持。发挥财政资金的引领推动作用，鼓励地方政府设立物联网专项基金，引导金融机构参与物联网新型基础设施建设。落实研发费用加计扣除等税收优惠政策，推动企业加大研发投入。促进社会资本与中小企业对接，推动解决物联网融资问题。

（五）深化国际交流与合作。依托“一带一路”倡议，充分利用区域全面经济伙伴关系协定（RCEP）等合作机制，促进技术研发、产业化推广、基础设施建设、人才培养等方面的交流与合作。支持国内物联网企业在海外设立分支机构，积极拓展国际合作渠道，提升国际化发展层次。

工业和信息化部

中央网络安全和信息化委员会办公室

科学技术部

生态环境部

住房和城乡建设部

农业农村部

国家卫生健康委员会

国家能源局

2021年9月10日

**P23**

**National Outline for Standardization Development**

**国家标准化发展纲要**

新华社北京10月10日电 近日，中共中央、国务院印发了《国家标准化发展纲要》，并发出通知，要求各地区各部门结合实际认真贯彻落实。

《国家标准化发展纲要》主要内容如下。

标准是经济活动和社会发展的技术支撑，是国家基础性制度的重要方面。标准化在推进国家治理体系和治理能力现代化中发挥着基础性、引领性作用。新时代推动高质量发展、全面建设社会主义现代化国家，迫切需要进一步加强标准化工作。为统筹推进标准化发展，制定本纲要。

一、总体要求

（一）指导思想。以习近平新时代中国特色社会主义思想为指导，深入贯彻党的十九大和十九届二中、三中、四中、五中全会精神，按照统筹推进“五位一体”总体布局和协调推进“四个全面”战略布局要求，坚持以人民为中心的发展思想，立足新发展阶段、贯彻新发展理念、构建新发展格局，优化标准化治理结构，增强标准化治理效能，提升标准国际化水平，加快构建推动高质量发展的标准体系，助力高技术创新，促进高水平开放，引领高质量发展，为全面建成社会主义现代化强国、实现中华民族伟大复兴的中国梦提供有力支撑。

（二）发展目标

到2025年，实现标准供给由政府主导向政府与市场并重转变，标准运用由产业与贸易为主向经济社会全域转变，标准化工作由国内驱动向国内国际相互促进转变，标准化发展由数量规模型向质量效益型转变。标准化更加有效推动国家综合竞争力提升，促进经济社会高质量发展，在构建新发展格局中发挥更大作用。

——全域标准化深度发展。农业、工业、服务业和社会事业等领域标准全覆盖，新兴产业标准地位凸显，健康、安全、环境标准支撑有力，农业标准化生产普及率稳步提升，推动高质量发展的标准体系基本建成。

——标准化水平大幅提升。共性关键技术和应用类科技计划项目形成标准研究成果的比率达到50%以上，政府颁布标准与市场自主制定标准结构更加优化，国家标准平均制定周期缩短至18个月以内，标准数字化程度不断提高，标准化的经济效益、社会效益、质量效益、生态效益充分显现。

——标准化开放程度显著增强。标准化国际合作深入拓展，互利共赢的国际标准化合作伙伴关系更加密切，标准化人员往来和技术合作日益加强，标准信息更大范围实现互联共享，我国标准制定透明度和国际化环境持续优化，国家标准与国际标准关键技术指标的一致性程度大幅提升，国际标准转化率达到85%以上。

——标准化发展基础更加牢固。建成一批国际一流的综合性、专业性标准化研究机构，若干国家级质量标准实验室，50个以上国家技术标准创新基地，形成标准、计量、认证认可、检验检测一体化运行的国家质量基础设施体系，标准化服务业基本适应经济社会发展需要。

到2035年，结构优化、先进合理、国际兼容的标准体系更加健全，具有中国特色的标准化管理体制更加完善，市场驱动、政府引导、企业为主、社会参与、开放融合的标准化工作格局全面形成。

二、推动标准化与科技创新互动发展

（三）加强关键技术领域标准研究。在人工智能、量子信息、生物技术等领域，开展标准化研究。在两化融合、新一代信息技术、大数据、区块链、卫生健康、新能源、新材料等应用前景广阔的技术领域，同步部署技术研发、标准研制与产业推广，加快新技术产业化步伐。研究制定智能船舶、高铁、新能源汽车、智能网联汽车和机器人等领域关键技术标准，推动产业变革。适时制定和完善生物医学研究、分子育种、无人驾驶等领域技术安全相关标准，提升技术领域安全风险管理水平。

（四）以科技创新提升标准水平。建立重大科技项目与标准化工作联动机制，将标准作为科技计划的重要产出，强化标准核心技术指标研究，重点支持基础通用、产业共性、新兴产业和融合技术等领域标准研制。及时将先进适用科技创新成果融入标准，提升标准水平。对符合条件的重要技术标准按规定给予奖励，激发全社会标准化创新活力。

（五）健全科技成果转化为标准的机制。完善科技成果转化为标准的评价机制和服务体系，推进技术经理人、科技成果评价服务等标准化工作。完善标准必要专利制度，加强标准制定过程中的知识产权保护，促进创新成果产业化应用。完善国家标准化技术文件制度，拓宽科技成果标准化渠道。将标准研制融入共性技术平台建设，缩短新技术、新工艺、新材料、新方法标准研制周期，加快成果转化应用步伐。

三、提升产业标准化水平

（六）筑牢产业发展基础。加强核心基础零部件（元器件）、先进基础工艺、关键基础材料与产业技术基础标准建设，加大基础通用标准研制应用力度。开展数据库等方面标准攻关，提升标准设计水平，制定安全可靠、国际先进的通用技术标准。

（七）推进产业优化升级。实施高端装备制造标准化强基工程，健全智能制造、绿色制造、服务型制造标准，形成产业优化升级的标准群，部分领域关键标准适度领先于产业发展平均水平。完善扩大内需方面的标准，不断提升消费品标准和质量水平，全面促进消费。推进服务业标准化、品牌化建设，健全服务业标准，重点加强食品冷链、现代物流、电子商务、物品编码、批发零售、房地产服务等领域标准化。健全和推广金融领域科技、产品、服务与基础设施等标准，有效防范化解金融风险。加快先进制造业和现代服务业融合发展标准化建设，推行跨行业跨领域综合标准化。建立健全大数据与产业融合标准，推进数字产业化和产业数字化。

（八）引领新产品新业态新模式快速健康发展。实施新产业标准化领航工程，开展新兴产业、未来产业标准化研究，制定一批应用带动的新标准，培育发展新业态新模式。围绕食品、医疗、应急、交通、水利、能源、金融等领域智慧化转型需求，加快完善相关标准。建立数据资源产权、交易流通、跨境传输和安全保护等标准规范，推动平台经济、共享经济标准化建设，支撑数字经济发展。健全依据标准实施科学有效监管机制，鼓励社会组织应用标准化手段加强自律、维护市场秩序。

（九）增强产业链供应链稳定性和产业综合竞争力。围绕生产、分配、流通、消费，加快关键环节、关键领域、关键产品的技术攻关和标准研制应用，提升产业核心竞争力。发挥关键技术标准在产业协同、技术协作中的纽带和驱动作用，实施标准化助力重点产业稳链工程，促进产业链上下游标准有效衔接，提升产业链供应链现代化水平。

（十）助推新型基础设施提质增效。实施新型基础设施标准化专项行动，加快推进通信网络基础设施、新技术基础设施、算力基础设施等信息基础设施系列标准研制，协同推进融合基础设施标准研制，建立工业互联网标准，制定支撑科学研究、技术研发、产品研制的创新基础设施标准，促进传统基础设施转型升级。

四、完善绿色发展标准化保障

（十一）建立健全碳达峰、碳中和标准。加快节能标准更新升级，抓紧修订一批能耗限额、产品设备能效强制性国家标准，提升重点产品能耗限额要求，扩大能耗限额标准覆盖范围，完善能源核算、检测认证、评估、审计等配套标准。加快完善地区、行业、企业、产品等碳排放核查核算标准。制定重点行业和产品温室气体排放标准，完善低碳产品标准标识制度。完善可再生能源标准，研究制定生态碳汇、碳捕集利用与封存标准。实施碳达峰、碳中和标准化提升工程。

（十二）持续优化生态系统建设和保护标准。不断完善生态环境质量和生态环境风险管控标准，持续改善生态环境质量。进一步完善污染防治标准，健全污染物排放、监管及防治标准，筑牢污染排放控制底线。统筹完善应对气候变化标准，制定修订应对气候变化减缓、适应、监测评估等标准。制定山水林田湖草沙多生态系统质量与经营利用标准，加快研究制定水土流失综合防治、生态保护修复、生态系统服务与评价、生态承载力评估、生态资源评价与监测、生物多样性保护及生态效益评估与生态产品价值实现等标准，增加优质生态产品供给，保障生态安全。

（十三）推进自然资源节约集约利用。构建自然资源统一调查、登记、评价、评估、监测等系列标准，研究制定土地、矿产资源等自然资源节约集约开发利用标准，推进能源资源绿色勘查与开发标准化。以自然资源资产清查统计和资产核算为重点，推动自然资源资产管理体系标准化。制定统一的国土空间规划技术标准，完善资源环境承载能力和国土空间开发适宜性评价机制。制定海洋资源开发保护标准，发展海洋经济，服务陆海统筹。

（十四）筑牢绿色生产标准基础。建立健全土壤质量及监测评价、农业投入品质量、适度规模养殖、循环型生态农业、农产品食品安全、监测预警等绿色农业发展标准。建立健全清洁生产标准，不断完善资源循环利用、产品绿色设计、绿色包装和绿色供应链、产业废弃物综合利用等标准。建立健全绿色金融、生态旅游等绿色发展标准。建立绿色建造标准，完善绿色建筑设计、施工、运维、管理标准。建立覆盖各类绿色生活设施的绿色社区、村庄建设标准。

（十五）强化绿色消费标准引领。完善绿色产品标准，建立绿色产品分类和评价标准，规范绿色产品、有机产品标识。构建节能节水、绿色采购、垃圾分类、制止餐饮浪费、绿色出行、绿色居住等绿色生活标准。分类建立绿色公共机构评价标准，合理制定消耗定额和垃圾排放指标。

五、加快城乡建设和社会建设标准化进程

（十六）推进乡村振兴标准化建设。强化标准引领，实施乡村振兴标准化行动。加强高标准农田建设，加快智慧农业标准研制，加快健全现代农业全产业链标准，加强数字乡村标准化建设，建立农业农村标准化服务与推广平台，推进地方特色产业标准化。完善乡村建设及评价标准，以农村环境监测与评价、村容村貌提升、农房建设、农村生活垃圾与污水治理、农村卫生厕所建设改造、公共基础设施建设等为重点，加快推进农村人居环境改善标准化工作。推进度假休闲、乡村旅游、民宿经济、传统村落保护利用等标准化建设，促进农村一二三产业融合发展。

（十七）推动新型城镇化标准化建设。研究制定公共资源配置标准，建立县城建设标准、小城镇公共设施建设标准。研究制定城市体检评估标准，健全城镇人居环境建设与质量评价标准。完善城市生态修复与功能完善、城市信息模型平台、建设工程防灾、更新改造及海绵城市建设等标准。推进城市设计、城市历史文化保护传承与风貌塑造、老旧小区改造等标准化建设，健全街区和公共设施配建标准。建立智能化城市基础设施建设、运行、管理、服务等系列标准，制定城市休闲慢行系统和综合管理服务等标准，研究制定新一代信息技术在城市基础设施规划建设、城市管理、应急处置等方面的应用标准。健全住房标准，完善房地产信息数据、物业服务等标准。推动智能建造标准化，完善建筑信息模型技术、施工现场监控等标准。开展城市标准化行动，健全智慧城市标准，推进城市可持续发展。

（十八）推动行政管理和社会治理标准化建设。探索开展行政管理标准建设和应用试点，重点推进行政审批、政务服务、政务公开、财政支出、智慧监管、法庭科学、审判执行、法律服务、公共资源交易等标准制定与推广，加快数字社会、数字政府、营商环境标准化建设，完善市场要素交易标准，促进高标准市场体系建设。强化信用信息采集与使用、数据安全和个人信息保护、网络安全保障体系和能力建设等领域标准的制定实施。围绕乡村治理、综治中心、网格化管理，开展社会治理标准化行动，推动社会治理标准化创新。

（十九）加强公共安全标准化工作。坚持人民至上、生命至上，实施公共安全标准化筑底工程，完善社会治安、刑事执法、反恐处突、交通运输、安全生产、应急管理、防灾减灾救灾标准，织密筑牢食品、药品、农药、粮食能源、水资源、生物、物资储备、产品质量、特种设备、劳动防护、消防、矿山、建筑、网络等领域安全标准网，提升洪涝干旱、森林草原火灾、地质灾害、地震等自然灾害防御工程标准，加强重大工程和各类基础设施的数据共享标准建设，提高保障人民群众生命财产安全水平。加快推进重大疫情防控救治、国家应急救援等领域标准建设，抓紧完善国家重大安全风险应急保障标准。构建多部门多区域多系统快速联动、统一高效的公共安全标准化协同机制，推进重大标准制定实施。

（二十）推进基本公共服务标准化建设。围绕幼有所育、学有所教、劳有所得、病有所医、老有所养、住有所居、弱有所扶等方面，实施基本公共服务标准体系建设工程，重点健全和推广全国统一的社会保险经办服务、劳动用工指导和就业创业服务、社会工作、养老服务、儿童福利、残疾人服务、社会救助、殡葬公共服务以及公共教育、公共文化体育、住房保障等领域技术标准，使发展成果更多更公平惠及全体人民。

（二十一）提升保障生活品质的标准水平。围绕普及健康生活、优化健康服务、倡导健康饮食、完善健康保障、建设健康环境、发展健康产业等方面，建立广覆盖、全方位的健康标准。制定公共体育设施、全民健身、训练竞赛、健身指导、线上和智能赛事等标准，建立科学完备、门类齐全的体育标准。开展养老和家政服务标准化专项行动，完善职业教育、智慧社区、社区服务等标准，加强慈善领域标准化建设。加快广播电视和网络视听内容融合生产、网络智慧传播、终端智能接收、安全智慧保障等标准化建设，建立全媒体传播标准。提高文化旅游产品与服务、消费保障、公园建设、景区管理等标准化水平。

六、提升标准化对外开放水平

（二十二）深化标准化交流合作。履行国际标准组织成员国责任义务，积极参与国际标准化活动。积极推进与共建“一带一路”国家在标准领域的对接合作，加强金砖国家、亚太经合组织等标准化对话，深化东北亚、亚太、泛美、欧洲、非洲等区域标准化合作，推进标准信息共享与服务，发展互利共赢的标准化合作伙伴关系。联合国际标准组织成员，推动气候变化、可持续城市和社区、清洁饮水与卫生设施、动植物卫生、绿色金融、数字领域等国际标准制定，分享我国标准化经验，积极参与民生福祉、性别平等、优质教育等国际标准化活动，助力联合国可持续发展目标实现。支持发展中国家提升利用标准化实现可持续发展的能力。

（二十三）强化贸易便利化标准支撑。持续开展重点领域标准比对分析，积极采用国际标准，大力推进中外标准互认，提高我国标准与国际标准的一致性程度。推出中国标准多语种版本，加快大宗贸易商品、对外承包工程等中国标准外文版编译。研究制定服务贸易标准，完善数字金融、国际贸易单一窗口等标准。促进内外贸质量标准、检验检疫、认证认可等相衔接，推进同线同标同质。创新标准化工作机制，支撑构建面向全球的高标准自由贸易区网络。

（二十四）推动国内国际标准化协同发展。统筹推进标准化与科技、产业、金融对外交流合作，促进政策、规则、标准联通。建立政府引导、企业主体、产学研联动的国际标准化工作机制。实施标准国际化跃升工程，推进中国标准与国际标准体系兼容。推动标准制度型开放，保障外商投资企业依法参与标准制定。支持企业、社会团体、科研机构等积极参与各类国际性专业标准组织。支持国际性专业标准组织来华落驻。

七、推动标准化改革创新

（二十五）优化标准供给结构。充分释放市场主体标准化活力，优化政府颁布标准与市场自主制定标准二元结构，大幅提升市场自主制定标准的比重。大力发展团体标准，实施团体标准培优计划，推进团体标准应用示范，充分发挥技术优势企业作用，引导社会团体制定原创性、高质量标准。加快建设协调统一的强制性国家标准，筑牢保障人身健康和生命财产安全、生态环境安全的底线。同步推进推荐性国家标准、行业标准和地方标准改革，强化推荐性标准的协调配套，防止地方保护和行业垄断。建立健全政府颁布标准采信市场自主制定标准的机制。

（二十六）深化标准化运行机制创新。建立标准创新型企业制度和标准融资增信制度，鼓励企业构建技术、专利、标准联动创新体系，支持领军企业联合科研机构、中小企业等建立标准合作机制，实施企业标准领跑者制度。建立国家统筹的区域标准化工作机制，将区域发展标准需求纳入国家标准体系建设，实现区域内标准发展规划、技术规则相互协同，服务国家重大区域战略实施。持续优化标准制定流程和平台、工具，健全企业、消费者等相关方参与标准制定修订的机制，加快标准升级迭代，提高标准质量水平。

（二十七）促进标准与国家质量基础设施融合发展。以标准为牵引，统筹布局国家质量基础设施资源，推进国家质量基础设施统一建设、统一管理，健全国家质量基础设施一体化发展体制机制。强化标准在计量量子化、检验检测智能化、认证市场化、认可全球化中的作用，通过人工智能、大数据、区块链等新一代信息技术的综合应用，完善质量治理，促进质量提升。强化国家质量基础设施全链条技术方案提供，运用标准化手段推动国家质量基础设施集成服务与产业价值链深度融合。

（二十八）强化标准实施应用。建立法规引用标准制度、政策实施配套标准制度，在法规和政策文件制定时积极应用标准。完善认证认可、检验检测、政府采购、招投标等活动中应用先进标准机制，推进以标准为依据开展宏观调控、产业推进、行业管理、市场准入和质量监管。健全基于标准或标准条款订立、履行合同的机制。建立标准版权制度、呈缴制度和市场自主制定标准交易制度，加大标准版权保护力度。按照国家有关规定，开展标准化试点示范工作，完善对标达标工作机制，推动企业提升执行标准能力，瞄准国际先进标准提高水平。

（二十九）加强标准制定和实施的监督。健全覆盖政府颁布标准制定实施全过程的追溯、监督和纠错机制，实现标准研制、实施和信息反馈闭环管理。开展标准质量和标准实施第三方评估，加强标准复审和维护更新。健全团体标准化良好行为评价机制。强化行业自律和社会监督，发挥市场对团体标准的优胜劣汰作用。有效实施企业标准自我声明公开和监督制度，将企业产品和服务符合标准情况纳入社会信用体系建设。建立标准实施举报、投诉机制，鼓励社会公众对标准实施情况进行监督。

八、夯实标准化发展基础

（三十）提升标准化技术支撑水平。加强标准化理论和应用研究，构建以国家级综合标准化研究机构为龙头，行业、区域和地方标准化研究机构为骨干的标准化科技体系。发挥优势企业在标准化科技体系中的作用。完善专业标准化技术组织体系，健全跨领域工作机制，提升开放性和透明度。建设若干国家级质量标准实验室、国家标准验证点和国家产品质量检验检测中心。有效整合标准技术、检测认证、知识产权、标准样品等资源，推进国家技术标准创新基地建设。建设国家数字标准馆和全国统一协调、分工负责的标准化公共服务平台。发展机器可读标准、开源标准，推动标准化工作向数字化、网络化、智能化转型。

（三十一）大力发展标准化服务业。完善促进标准、计量、认证认可、检验检测等标准化相关高技术服务业发展的政策措施，培育壮大标准化服务业市场主体，鼓励有条件地区探索建立标准化服务业产业集聚区，健全标准化服务评价机制和标准化服务业统计分析报告制度。鼓励标准化服务机构面向中小微企业实际需求，整合上下游资源，提供标准化整体解决方案。大力发展新型标准化服务工具和模式，提升服务专业化水平。

（三十二）加强标准化人才队伍建设。将标准化纳入普通高等教育、职业教育和继续教育，开展专业与标准化教育融合试点。构建多层次从业人员培养培训体系，开展标准化专业人才培养培训和国家质量基础设施综合教育。建立健全标准化领域人才的职业能力评价和激励机制。造就一支熟练掌握国际规则、精通专业技术的职业化人才队伍。提升科研人员标准化能力，充分发挥标准化专家在国家科技决策咨询中的作用，建设国家标准化高端智库。加强基层标准化管理人员队伍建设，支持西部地区标准化专业人才队伍建设。

（三十三）营造标准化良好社会环境。充分利用世界标准日等主题活动，宣传标准化作用，普及标准化理念、知识和方法，提升全社会标准化意识，推动标准化成为政府管理、社会治理、法人治理的重要工具。充分发挥标准化社会团体的桥梁和纽带作用，全方位、多渠道开展标准化宣传，讲好标准化故事。大力培育发展标准化文化。

九、组织实施

（三十四）加强组织领导。坚持党对标准化工作的全面领导。进一步完善国务院标准化协调推进部际联席会议制度，健全统一、权威、高效的管理体制和工作机制，强化部门协同、上下联动。各省（自治区、直辖市）要建立健全标准化工作协调推进领导机制，将标准化工作纳入政府绩效评价和政绩考核。各地区各有关部门要将本纲要主要任务与国民经济和社会发展规划有效衔接、同步推进，确保各项任务落到实处。

（三十五）完善配套政策。各地区各有关部门要强化金融、信用、人才等政策支持，促进科技、产业、贸易等政策协同。按照有关规定开展表彰奖励。发挥财政资金引导作用，积极引导社会资本投入标准化工作。完善标准化统计调查制度，开展标准化发展评价，将相关指标纳入国民经济和社会发展统计。建立本纲要实施评估机制，把相关结果作为改进标准化工作的重要依据。重大事项及时向党中央、国务院请示报告。

中共中央 国务院

2021年10月10日

**P24**

**Opinions on Promoting Green Development in Urban and Rural Construction**

**关于推动城乡建设绿色发展的意见**

新华社北京10月21日电 近日，中共中央办公厅、国务院办公厅印发了《关于推动城乡建设绿色发展的意见》，并发出通知，要求各地区各部门结合实际认真贯彻落实。

《关于推动城乡建设绿色发展的意见》主要内容如下。

城乡建设是推动绿色发展、建设美丽中国的重要载体。党的十八大以来，我国人居环境持续改善，住房水平显著提高，同时仍存在整体性缺乏、系统性不足、宜居性不高、包容性不够等问题，大量建设、大量消耗、大量排放的建设方式尚未根本扭转。为推动城乡建设绿色发展，现提出如下意见。

一、总体要求

（一）指导思想。以习近平新时代中国特色社会主义思想为指导，深入贯彻党的十九大和十九届二中、三中、四中、五中全会精神，践行习近平生态文明思想，按照党中央、国务院决策部署，立足新发展阶段、贯彻新发展理念、构建新发展格局，坚持以人民为中心，坚持生态优先、节约优先、保护优先，坚持系统观念，统筹发展和安全，同步推进物质文明建设与生态文明建设，落实碳达峰、碳中和目标任务，推进城市更新行动、乡村建设行动，加快转变城乡建设方式，促进经济社会发展全面绿色转型，为全面建设社会主义现代化国家奠定坚实基础。

（二）工作原则。坚持人与自然和谐共生，尊重自然、顺应自然、保护自然，推动构建人与自然生命共同体。坚持整体与局部相协调，统筹规划、建设、管理三大环节，统筹城镇和乡村建设。坚持效率与均衡并重，促进城乡资源能源节约集约利用，实现人口、经济发展与生态资源协调。坚持公平与包容相融合，完善城乡基础设施，推进基本公共服务均等化。坚持保护与发展相统一，传承中华优秀传统文化，推动创造性转化、创新性发展。坚持党建引领与群众共建共治共享相结合，完善群众参与机制，共同创造美好环境。

（三）总体目标

到2025年，城乡建设绿色发展体制机制和政策体系基本建立，建设方式绿色转型成效显著，碳减排扎实推进，城市整体性、系统性、生长性增强，“城市病”问题缓解，城乡生态环境质量整体改善，城乡发展质量和资源环境承载能力明显提升，综合治理能力显著提高，绿色生活方式普遍推广。

到2035年，城乡建设全面实现绿色发展，碳减排水平快速提升，城市和乡村品质全面提升，人居环境更加美好，城乡建设领域治理体系和治理能力基本实现现代化，美丽中国建设目标基本实现。

二、推进城乡建设一体化发展

（一）促进区域和城市群绿色发展。建立健全区域和城市群绿色发展协调机制，充分发挥各城市比较优势，促进资源有效配置。在国土空间规划中统筹划定生态保护红线、永久基本农田、城镇开发边界等管控边界，统筹生产、生活、生态空间，实施最严格的耕地保护制度，建立水资源刚性约束制度，建设与资源环境承载能力相匹配、重大风险防控相结合的空间格局。统筹区域、城市群和都市圈内大中小城市住房建设，与人口构成、产业结构相适应。协同建设区域生态网络和绿道体系，衔接生态保护红线、环境质量底线、资源利用上线和生态环境准入清单，改善区域生态环境。推进区域重大基础设施和公共服务设施共建共享，建立功能完善、衔接紧密、保障有力的城市群综合立体交通等现代化设施网络体系。

（二）建设人与自然和谐共生的美丽城市。建立分层次、分区域协调管控机制，以自然资源承载能力和生态环境容量为基础，合理确定城市人口、用水、用地规模，合理确定开发建设密度和强度。提高中心城市综合承载能力，建设一批产城融合、职住平衡、生态宜居、交通便利的郊区新城，推动多中心、组团式发展。落实规划环评要求和防噪声距离。大力推进城市节水，提高水资源集约节约利用水平。实施海绵城市建设，完善城市防洪排涝体系，提高城市防灾减灾能力，增强城市韧性。实施城市生态修复工程，保护城市山体自然风貌，修复江河、湖泊、湿地，加强城市公园和绿地建设，推进立体绿化，构建连续完整的生态基础设施体系。实施城市功能完善工程，加强婴幼儿照护机构、幼儿园、中小学校、医疗卫生机构、养老服务机构、儿童福利机构、未成年人救助保护机构、社区足球场地等设施建设，增加公共活动空间，建设体育公园，完善文化和旅游消费场所设施，推动发展城市新业态、新功能。建立健全推进城市生态修复、功能完善工程标准规范和工作体系。推动绿色城市、森林城市、“无废城市”建设，深入开展绿色社区创建行动。推进以县城为重要载体的城镇化建设，加强县城绿色低碳建设，大力提升县城公共设施和服务水平。

（三）打造绿色生态宜居的美丽乡村。按照产业兴旺、生态宜居、乡风文明、治理有效、生活富裕的总要求，以持续改善农村人居环境为目标，建立乡村建设评价机制，探索县域乡村发展路径。提高农房设计和建造水平，建设满足乡村生产生活实际需要的新型农房，完善水、电、气、厕配套附属设施，加强既有农房节能改造。保护塑造乡村风貌，延续乡村历史文脉，严格落实有关规定，不破坏地形地貌、不拆传统民居、不砍老树、不盖高楼。统筹布局县城、中心镇、行政村基础设施和公共服务设施，促进城乡设施联动发展。提高镇村设施建设水平，持续推进农村生活垃圾、污水、厕所粪污、畜禽养殖粪污治理，实施农村水系综合整治，推进生态清洁流域建设，加强水土流失综合治理，加强农村防灾减灾能力建设。立足资源优势打造各具特色的农业全产业链，发展多种形式适度规模经营，支持以“公司+农户”等模式对接市场，培育乡村文化、旅游、休闲、民宿、健康养老、传统手工艺等新业态，强化农产品及其加工副产物综合利用，拓宽农民增收渠道，促进产镇融合、产村融合，推动农村一二三产业融合发展。

三、转变城乡建设发展方式

（一）建设高品质绿色建筑。实施建筑领域碳达峰、碳中和行动。规范绿色建筑设计、施工、运行、管理，鼓励建设绿色农房。推进既有建筑绿色化改造，鼓励与城镇老旧小区改造、农村危房改造、抗震加固等同步实施。开展绿色建筑、节约型机关、绿色学校、绿色医院创建行动。加强财政、金融、规划、建设等政策支持，推动高质量绿色建筑规模化发展，大力推广超低能耗、近零能耗建筑，发展零碳建筑。实施绿色建筑统一标识制度。建立城市建筑用水、用电、用气、用热等数据共享机制，提升建筑能耗监测能力。推动区域建筑能效提升，推广合同能源管理、合同节水管理服务模式，降低建筑运行能耗、水耗，大力推动可再生能源应用，鼓励智能光伏与绿色建筑融合创新发展。

（二）提高城乡基础设施体系化水平。建立健全基础设施建档制度，普查现有基础设施，统筹地下空间综合利用。推进城乡基础设施补短板和更新改造专项行动以及体系化建设，提高基础设施绿色、智能、协同、安全水平。加强公交优先、绿色出行的城市街区建设，合理布局和建设城市公交专用道、公交场站、车船用加气加注站、电动汽车充换电站，加快发展智能网联汽车、新能源汽车、智慧停车及无障碍基础设施，强化城市轨道交通与其他交通方式衔接。加强交通噪声管控，落实城市交通设计、规划、建设和运行噪声技术要求。加强城市高层建筑、大型商业综合体等重点场所消防安全管理，打通消防生命通道，推进城乡应急避难场所建设。持续推动城镇污水处理提质增效，完善再生水、集蓄雨水等非常规水源利用系统，推进城镇污水管网全覆盖，建立污水处理系统运营管理长效机制。因地制宜加快连接港区管网建设，做好船舶生活污水收集处理。统筹推进煤改电、煤改气及集中供热替代等，加快农村电网、天然气管网、热力管网等建设改造。

（三）加强城乡历史文化保护传承。建立完善城乡历史文化保护传承体系，健全管理监督机制，完善保护标准和政策法规，严格落实责任，依法问责处罚。开展历史文化资源普查，做好测绘、建档、挂牌工作。建立历史文化名城、名镇、名村及传统村落保护制度，加大保护力度，不拆除历史建筑，不拆真遗存，不建假古董，做到按级施保、应保尽保。完善项目审批、财政支持、社会参与等制度机制，推动历史建筑绿色化更新改造、合理利用。建立保护项目维护修缮机制，保护和培养传统工匠队伍，传承传统建筑绿色营造方式。

（四）实现工程建设全过程绿色建造。开展绿色建造示范工程创建行动，推广绿色化、工业化、信息化、集约化、产业化建造方式，加强技术创新和集成，利用新技术实现精细化设计和施工。大力发展装配式建筑，重点推动钢结构装配式住宅建设，不断提升构件标准化水平，推动形成完整产业链，推动智能建造和建筑工业化协同发展。完善绿色建材产品认证制度，开展绿色建材应用示范工程建设，鼓励使用综合利用产品。加强建筑材料循环利用，促进建筑垃圾减量化，严格施工扬尘管控，采取综合降噪措施管控施工噪声。推动传统建筑业转型升级，完善工程建设组织模式，加快推行工程总承包，推广全过程工程咨询，推进民用建筑工程建筑师负责制。加快推进工程造价改革。改革建筑劳动用工制度，大力发展专业作业企业，培育职业化、专业化、技能化建筑产业工人队伍。

（五）推动形成绿色生活方式。推广节能低碳节水用品，推动太阳能、再生水等应用，鼓励使用环保再生产品和绿色设计产品，减少一次性消费品和包装用材消耗。倡导绿色装修，鼓励选用绿色建材、家具、家电。持续推进垃圾分类和减量化、资源化，推动生活垃圾源头减量，建立健全生活垃圾分类投放、分类收集、分类转运、分类处理系统。加强危险废物、医疗废物收集处理，建立完善应急处置机制。科学制定城市慢行系统规划，因地制宜建设自行车专用道和绿道，全面开展人行道净化行动，改造提升重点城市步行街。深入开展绿色出行创建行动，优化交通出行结构，鼓励公众选择公共交通、自行车和步行等出行方式。

四、创新工作方法

（一）统筹城乡规划建设管理。坚持总体国家安全观，以城乡建设绿色发展为目标，加强顶层设计，编制相关规划，建立规划、建设、管理三大环节统筹机制，统筹城市布局的经济需要、生活需要、生态需要、安全需要，统筹地上地下空间综合利用，统筹各类基础设施建设，系统推进重大工程项目。创新城乡建设管控和引导机制，完善城市形态，提升建筑品质，塑造时代特色风貌。完善城乡规划、建设、管理制度，动态管控建设进程，确保一张蓝图实施不走样、不变形。

（二）建立城市体检评估制度。建立健全“一年一体检、五年一评估”的城市体检评估制度，强化对相关规划实施情况和历史文化保护传承、基础设施效率、生态建设、污染防治等的评估。制定城市体检评估标准，将绿色发展纳入评估指标体系。城市政府作为城市体检评估工作主体，要定期开展体检评估，制定年度建设和整治行动计划，依法依规向社会公开体检评估结果。加强对相关规划实施的监督，维护规划的严肃性权威性。

（三）加大科技创新力度。完善以市场为导向的城乡建设绿色技术创新体系，培育壮大一批绿色低碳技术创新企业，充分发挥国家工程研究中心、国家技术创新中心、国家企业技术中心、国家重点实验室等创新平台对绿色低碳技术的支撑作用。加强国家科技计划研究，系统布局一批支撑城乡建设绿色发展的研发项目，组织开展重大科技攻关，加大科技成果集成创新力度。建立科技项目成果库和公开制度，鼓励科研院所、企业等主体融通创新、利益共享，促进科技成果转化。建设国际化工程建设标准体系，完善相关标准。

（四）推动城市智慧化建设。建立完善智慧城市建设标准和政策法规，加快推进信息技术与城市建设技术、业务、数据融合。开展城市信息模型平台建设，推动建筑信息模型深化应用，推进工程建设项目智能化管理，促进城市建设及运营模式变革。搭建城市运行管理服务平台，加强对市政基础设施、城市环境、城市交通、城市防灾的智慧化管理，推动城市地下空间信息化、智能化管控，提升城市安全风险监测预警水平。完善工程建设项目审批管理系统，逐步实现智能化全程网上办理，推进与投资项目在线审批监管平台等互联互通。搭建智慧物业管理服务平台，加强社区智慧化建设管理，为群众提供便捷服务。

（五）推动美好环境共建共治共享。建立党组织统一领导、政府依法履责、各类组织积极协同、群众广泛参与，自治、法治、德治相结合的基层治理体系，推动形成建设美好人居环境的合力，实现决策共谋、发展共建、建设共管、效果共评、成果共享。下沉公共服务和社会管理资源，按照有关规定探索适宜城乡社区治理的项目招投标、奖励等机制，解决群众身边、房前屋后的实事小事。以城镇老旧小区改造、历史文化街区保护与利用、美丽乡村建设、生活垃圾分类等为抓手和载体，构建社区生活圈，广泛发动组织群众参与城乡社区治理，共同建设美好家园。

五、加强组织实施

（一）加强党的全面领导。把党的全面领导贯穿城乡建设绿色发展各方面各环节，不折不扣贯彻落实中央决策部署。建立省负总责、市县具体负责的工作机制，地方各级党委和政府要充分认识推动城乡建设绿色发展的重要意义，加快形成党委统一领导、党政齐抓共管的工作格局。各省（自治区、直辖市）要根据本意见确定本地区推动城乡建设绿色发展的工作目标和重点任务，加强统筹协调，推进解决重点难点问题。市、县作为工作责任主体，要制定具体措施，切实抓好组织落实。

（二）完善工作机制。加强部门统筹协调，住房城乡建设、发展改革、工业和信息化、民政、财政、自然资源、生态环境、交通运输、水利、农业农村、文化和旅游、金融、市场监管等部门要按照各自职责完善有关支持政策，推动落实重点任务。加大财政、金融支持力度，完善绿色金融体系，支持城乡建设绿色发展重大项目和重点任务。各地要结合实际建立相关工作机制，确保各项任务落实落地。

（三）健全支撑体系。建立完善推动城乡建设绿色发展的体制机制和制度，推进城乡建设领域治理体系和治理能力现代化。制定修订城乡建设和历史文化保护传承等法律法规，为城乡建设绿色发展提供法治保障。深化城市管理和执法体制改革，加强队伍建设，推进严格规范公正文明执法，提高城市管理和执法能力水平。健全社会公众满意度评价和第三方考评机制，由群众评判城乡建设绿色发展成效。加快管理、技术和机制创新，培育绿色发展新动能，实现动力变革。

（四）加强培训宣传。中央组织部、住房城乡建设部要会同国家发展改革委、自然资源部、生态环境部加强培训，不断提高党政主要负责同志推动城乡建设绿色发展的能力和水平。在各级党校（行政学院）、干部学院增加相关培训课程，编辑出版系列教材，教育引导各级领导干部和广大专业技术人员尊重城乡发展规律，尊重自然生态环境，尊重历史文化传承，重视和回应群众诉求。加强国际交流合作，广泛吸收借鉴先进经验。采取多种形式加强教育宣传和舆论引导，普及城乡建设绿色发展法律法规和科学知识。

中共中央办公厅 国务院办公厅

2011年10月21日

**P25**

**Notice on the Issuance of the Action Plan for Carbon Peaking Before 2030**

**2030年前碳达峰行动方案**

国发〔2021〕23号

为深入贯彻落实党中央、国务院关于碳达峰、碳中和的重大战略决策，扎实推进碳达峰行动，制定本方案。

一、总体要求

（一）指导思想。以习近平新时代中国特色社会主义思想为指导，全面贯彻党的十九大和十九届二中、三中、四中、五中全会精神，深入贯彻习近平生态文明思想，立足新发展阶段，完整、准确、全面贯彻新发展理念，构建新发展格局，坚持系统观念，处理好发展和减排、整体和局部、短期和中长期的关系，统筹稳增长和调结构，把碳达峰、碳中和纳入经济社会发展全局，坚持“全国统筹、节约优先、双轮驱动、内外畅通、防范风险”的总方针，有力有序有效做好碳达峰工作，明确各地区、各领域、各行业目标任务，加快实现生产生活方式绿色变革，推动经济社会发展建立在资源高效利用和绿色低碳发展的基础之上，确保如期实现2030年前碳达峰目标。

（二）工作原则。

——总体部署、分类施策。坚持全国一盘棋，强化顶层设计和各方统筹。各地区、各领域、各行业因地制宜、分类施策，明确既符合自身实际又满足总体要求的目标任务。

——系统推进、重点突破。全面准确认识碳达峰行动对经济社会发展的深远影响，加强政策的系统性、协同性。抓住主要矛盾和矛盾的主要方面，推动重点领域、重点行业和有条件的地方率先达峰。

——双轮驱动、两手发力。更好发挥政府作用，构建新型举国体制，充分发挥市场机制作用，大力推进绿色低碳科技创新，深化能源和相关领域改革，形成有效激励约束机制。

——稳妥有序、安全降碳。立足我国富煤贫油少气的能源资源禀赋，坚持先立后破，稳住存量，拓展增量，以保障国家能源安全和经济发展为底线，争取时间实现新能源的逐渐替代，推动能源低碳转型平稳过渡，切实保障国家能源安全、产业链供应链安全、粮食安全和群众正常生产生活，着力化解各类风险隐患，防止过度反应，稳妥有序、循序渐进推进碳达峰行动，确保安全降碳。

二、主要目标

“十四五”期间，产业结构和能源结构调整优化取得明显进展，重点行业能源利用效率大幅提升，煤炭消费增长得到严格控制，新型电力系统加快构建，绿色低碳技术研发和推广应用取得新进展，绿色生产生活方式得到普遍推行，有利于绿色低碳循环发展的政策体系进一步完善。到2025年，非化石能源消费比重达到20%左右，单位国内生产总值能源消耗比2020年下降13.5%，单位国内生产总值二氧化碳排放比2020年下降18%，为实现碳达峰奠定坚实基础。

“十五五”期间，产业结构调整取得重大进展，清洁低碳安全高效的能源体系初步建立，重点领域低碳发展模式基本形成，重点耗能行业能源利用效率达到国际先进水平，非化石能源消费比重进一步提高，煤炭消费逐步减少，绿色低碳技术取得关键突破，绿色生活方式成为公众自觉选择，绿色低碳循环发展政策体系基本健全。到2030年，非化石能源消费比重达到25%左右，单位国内生产总值二氧化碳排放比2005年下降65%以上，顺利实现2030年前碳达峰目标。

三、重点任务

将碳达峰贯穿于经济社会发展全过程和各方面，重点实施能源绿色低碳转型行动、节能降碳增效行动、工业领域碳达峰行动、城乡建设碳达峰行动、交通运输绿色低碳行动、循环经济助力降碳行动、绿色低碳科技创新行动、碳汇能力巩固提升行动、绿色低碳全民行动、各地区梯次有序碳达峰行动等“碳达峰十大行动”。

（一）能源绿色低碳转型行动。

能源是经济社会发展的重要物质基础，也是碳排放的最主要来源。要坚持安全降碳，在保障能源安全的前提下，大力实施可再生能源替代，加快构建清洁低碳安全高效的能源体系。

1．推进煤炭消费替代和转型升级。加快煤炭减量步伐，“十四五”时期严格合理控制煤炭消费增长，“十五五”时期逐步减少。严格控制新增煤电项目，新建机组煤耗标准达到国际先进水平，有序淘汰煤电落后产能，加快现役机组节能升级和灵活性改造，积极推进供热改造，推动煤电向基础保障性和系统调节性电源并重转型。严控跨区外送可再生能源电力配套煤电规模，新建通道可再生能源电量比例原则上不低于50%。推动重点用煤行业减煤限煤。大力推动煤炭清洁利用，合理划定禁止散烧区域，多措并举、积极有序推进散煤替代，逐步减少直至禁止煤炭散烧。

2．大力发展新能源。全面推进风电、太阳能发电大规模开发和高质量发展，坚持集中式与分布式并举，加快建设风电和光伏发电基地。加快智能光伏产业创新升级和特色应用，创新“光伏+”模式，推进光伏发电多元布局。坚持陆海并重，推动风电协调快速发展，完善海上风电产业链，鼓励建设海上风电基地。积极发展太阳能光热发电，推动建立光热发电与光伏发电、风电互补调节的风光热综合可再生能源发电基地。因地制宜发展生物质发电、生物质能清洁供暖和生物天然气。探索深化地热能以及波浪能、潮流能、温差能等海洋新能源开发利用。进一步完善可再生能源电力消纳保障机制。到2030年，风电、太阳能发电总装机容量达到12亿千瓦以上。

3．因地制宜开发水电。积极推进水电基地建设，推动金沙江上游、澜沧江上游、雅砻江中游、黄河上游等已纳入规划、符合生态保护要求的水电项目开工建设，推进雅鲁藏布江下游水电开发，推动小水电绿色发展。推动西南地区水电与风电、太阳能发电协同互补。统筹水电开发和生态保护，探索建立水能资源开发生态保护补偿机制。“十四五”、“十五五”期间分别新增水电装机容量4000万千瓦左右，西南地区以水电为主的可再生能源体系基本建立。

4．积极安全有序发展核电。合理确定核电站布局和开发时序，在确保安全的前提下有序发展核电，保持平稳建设节奏。积极推动高温气冷堆、快堆、模块化小型堆、海上浮动堆等先进堆型示范工程，开展核能综合利用示范。加大核电标准化、自主化力度，加快关键技术装备攻关，培育高端核电装备制造产业集群。实行最严格的安全标准和最严格的监管，持续提升核安全监管能力。

5．合理调控油气消费。保持石油消费处于合理区间，逐步调整汽油消费规模，大力推进先进生物液体燃料、可持续航空燃料等替代传统燃油，提升终端燃油产品能效。加快推进页岩气、煤层气、致密油（气）等非常规油气资源规模化开发。有序引导天然气消费，优化利用结构，优先保障民生用气，大力推动天然气与多种能源融合发展，因地制宜建设天然气调峰电站，合理引导工业用气和化工原料用气。支持车船使用液化天然气作为燃料。

6．加快建设新型电力系统。构建新能源占比逐渐提高的新型电力系统，推动清洁电力资源大范围优化配置。大力提升电力系统综合调节能力，加快灵活调节电源建设，引导自备电厂、传统高载能工业负荷、工商业可中断负荷、电动汽车充电网络、虚拟电厂等参与系统调节，建设坚强智能电网，提升电网安全保障水平。积极发展“新能源+储能”、源网荷储一体化和多能互补，支持分布式新能源合理配置储能系统。制定新一轮抽水蓄能电站中长期发展规划，完善促进抽水蓄能发展的政策机制。加快新型储能示范推广应用。深化电力体制改革，加快构建全国统一电力市场体系。到2025年，新型储能装机容量达到3000万千瓦以上。到2030年，抽水蓄能电站装机容量达到1.2亿千瓦左右，省级电网基本具备5%以上的尖峰负荷响应能力。

（二）节能降碳增效行动。

落实节约优先方针，完善能源消费强度和总量双控制度，严格控制能耗强度，合理控制能源消费总量，推动能源消费革命，建设能源节约型社会。

1．全面提升节能管理能力。推行用能预算管理，强化固定资产投资项目节能审查，对项目用能和碳排放情况进行综合评价，从源头推进节能降碳。提高节能管理信息化水平，完善重点用能单位能耗在线监测系统，建立全国性、行业性节能技术推广服务平台，推动高耗能企业建立能源管理中心。完善能源计量体系，鼓励采用认证手段提升节能管理水平。加强节能监察能力建设，健全省、市、县三级节能监察体系，建立跨部门联动机制，综合运用行政处罚、信用监管、绿色电价等手段，增强节能监察约束力。

2．实施节能降碳重点工程。实施城市节能降碳工程，开展建筑、交通、照明、供热等基础设施节能升级改造，推进先进绿色建筑技术示范应用，推动城市综合能效提升。实施园区节能降碳工程，以高耗能高排放项目（以下称“两高”项目）集聚度高的园区为重点，推动能源系统优化和梯级利用，打造一批达到国际先进水平的节能低碳园区。实施重点行业节能降碳工程，推动电力、钢铁、有色金属、建材、石化化工等行业开展节能降碳改造，提升能源资源利用效率。实施重大节能降碳技术示范工程，支持已取得突破的绿色低碳关键技术开展产业化示范应用。

3．推进重点用能设备节能增效。以电机、风机、泵、压缩机、变压器、换热器、工业锅炉等设备为重点，全面提升能效标准。建立以能效为导向的激励约束机制，推广先进高效产品设备，加快淘汰落后低效设备。加强重点用能设备节能审查和日常监管，强化生产、经营、销售、使用、报废全链条管理，严厉打击违法违规行为，确保能效标准和节能要求全面落实。

4．加强新型基础设施节能降碳。优化新型基础设施空间布局，统筹谋划、科学配置数据中心等新型基础设施，避免低水平重复建设。优化新型基础设施用能结构，采用直流供电、分布式储能、“光伏+储能”等模式，探索多样化能源供应，提高非化石能源消费比重。对标国际先进水平，加快完善通信、运算、存储、传输等设备能效标准，提升准入门槛，淘汰落后设备和技术。加强新型基础设施用能管理，将年综合能耗超过1万吨标准煤的数据中心全部纳入重点用能单位能耗在线监测系统，开展能源计量审查。推动既有设施绿色升级改造，积极推广使用高效制冷、先进通风、余热利用、智能化用能控制等技术，提高设施能效水平。

（三）工业领域碳达峰行动。

工业是产生碳排放的主要领域之一，对全国整体实现碳达峰具有重要影响。工业领域要加快绿色低碳转型和高质量发展，力争率先实现碳达峰。

1．推动工业领域绿色低碳发展。优化产业结构，加快退出落后产能，大力发展战略性新兴产业，加快传统产业绿色低碳改造。促进工业能源消费低碳化，推动化石能源清洁高效利用，提高可再生能源应用比重，加强电力需求侧管理，提升工业电气化水平。深入实施绿色制造工程，大力推行绿色设计，完善绿色制造体系，建设绿色工厂和绿色工业园区。推进工业领域数字化智能化绿色化融合发展，加强重点行业和领域技术改造。

2．推动钢铁行业碳达峰。深化钢铁行业供给侧结构性改革，严格执行产能置换，严禁新增产能，推进存量优化，淘汰落后产能。推进钢铁企业跨地区、跨所有制兼并重组，提高行业集中度。优化生产力布局，以京津冀及周边地区为重点，继续压减钢铁产能。促进钢铁行业结构优化和清洁能源替代，大力推进非高炉炼铁技术示范，提升废钢资源回收利用水平，推行全废钢电炉工艺。推广先进适用技术，深挖节能降碳潜力，鼓励钢化联产，探索开展氢冶金、二氧化碳捕集利用一体化等试点示范，推动低品位余热供暖发展。

3．推动有色金属行业碳达峰。巩固化解电解铝过剩产能成果，严格执行产能置换，严控新增产能。推进清洁能源替代，提高水电、风电、太阳能发电等应用比重。加快再生有色金属产业发展，完善废弃有色金属资源回收、分选和加工网络，提高再生有色金属产量。加快推广应用先进适用绿色低碳技术，提升有色金属生产过程余热回收水平，推动单位产品能耗持续下降。

4．推动建材行业碳达峰。加强产能置换监管，加快低效产能退出，严禁新增水泥熟料、平板玻璃产能，引导建材行业向轻型化、集约化、制品化转型。推动水泥错峰生产常态化，合理缩短水泥熟料装置运转时间。因地制宜利用风能、太阳能等可再生能源，逐步提高电力、天然气应用比重。鼓励建材企业使用粉煤灰、工业废渣、尾矿渣等作为原料或水泥混合材。加快推进绿色建材产品认证和应用推广，加强新型胶凝材料、低碳混凝土、木竹建材等低碳建材产品研发应用。推广节能技术设备，开展能源管理体系建设，实现节能增效。

5．推动石化化工行业碳达峰。优化产能规模和布局，加大落后产能淘汰力度，有效化解结构性过剩矛盾。严格项目准入，合理安排建设时序，严控新增炼油和传统煤化工生产能力，稳妥有序发展现代煤化工。引导企业转变用能方式，鼓励以电力、天然气等替代煤炭。调整原料结构，控制新增原料用煤，拓展富氢原料进口来源，推动石化化工原料轻质化。优化产品结构，促进石化化工与煤炭开采、冶金、建材、化纤等产业协同发展，加强炼厂干气、液化气等副产气体高效利用。鼓励企业节能升级改造，推动能量梯级利用、物料循环利用。到2025年，国内原油一次加工能力控制在10亿吨以内，主要产品产能利用率提升至80%以上。

6．坚决遏制“两高”项目盲目发展。采取强有力措施，对“两高”项目实行清单管理、分类处置、动态监控。全面排查在建项目，对能效水平低于本行业能耗限额准入值的，按有关规定停工整改，推动能效水平应提尽提，力争全面达到国内乃至国际先进水平。科学评估拟建项目，对产能已饱和的行业，按照“减量替代”原则压减产能；对产能尚未饱和的行业，按照国家布局和审批备案等要求，对标国际先进水平提高准入门槛；对能耗量较大的新兴产业，支持引导企业应用绿色低碳技术，提高能效水平。深入挖潜存量项目，加快淘汰落后产能，通过改造升级挖掘节能减排潜力。强化常态化监管，坚决拿下不符合要求的“两高”项目。

（四）城乡建设碳达峰行动。

加快推进城乡建设绿色低碳发展，城市更新和乡村振兴都要落实绿色低碳要求。

1．推进城乡建设绿色低碳转型。推动城市组团式发展，科学确定建设规模，控制新增建设用地过快增长。倡导绿色低碳规划设计理念，增强城乡气候韧性，建设海绵城市。推广绿色低碳建材和绿色建造方式，加快推进新型建筑工业化，大力发展装配式建筑，推广钢结构住宅，推动建材循环利用，强化绿色设计和绿色施工管理。加强县城绿色低碳建设。推动建立以绿色低碳为导向的城乡规划建设管理机制，制定建筑拆除管理办法，杜绝大拆大建。建设绿色城镇、绿色社区。

2．加快提升建筑能效水平。加快更新建筑节能、市政基础设施等标准，提高节能降碳要求。加强适用于不同气候区、不同建筑类型的节能低碳技术研发和推广，推动超低能耗建筑、低碳建筑规模化发展。加快推进居住建筑和公共建筑节能改造，持续推动老旧供热管网等市政基础设施节能降碳改造。提升城镇建筑和基础设施运行管理智能化水平，加快推广供热计量收费和合同能源管理，逐步开展公共建筑能耗限额管理。到2025年，城镇新建建筑全面执行绿色建筑标准。

3．加快优化建筑用能结构。深化可再生能源建筑应用，推广光伏发电与建筑一体化应用。积极推动严寒、寒冷地区清洁取暖，推进热电联产集中供暖，加快工业余热供暖规模化应用，积极稳妥开展核能供热示范，因地制宜推行热泵、生物质能、地热能、太阳能等清洁低碳供暖。引导夏热冬冷地区科学取暖，因地制宜采用清洁高效取暖方式。提高建筑终端电气化水平，建设集光伏发电、储能、直流配电、柔性用电于一体的“光储直柔”建筑。到2025年，城镇建筑可再生能源替代率达到8%，新建公共机构建筑、新建厂房屋顶光伏覆盖率力争达到50%。

4．推进农村建设和用能低碳转型。推进绿色农房建设，加快农房节能改造。持续推进农村地区清洁取暖，因地制宜选择适宜取暖方式。发展节能低碳农业大棚。推广节能环保灶具、电动农用车辆、节能环保农机和渔船。加快生物质能、太阳能等可再生能源在农业生产和农村生活中的应用。加强农村电网建设，提升农村用能电气化水平。

（五）交通运输绿色低碳行动。

加快形成绿色低碳运输方式，确保交通运输领域碳排放增长保持在合理区间。

1．推动运输工具装备低碳转型。积极扩大电力、氢能、天然气、先进生物液体燃料等新能源、清洁能源在交通运输领域应用。大力推广新能源汽车，逐步降低传统燃油汽车在新车产销和汽车保有量中的占比，推动城市公共服务车辆电动化替代，推广电力、氢燃料、液化天然气动力重型货运车辆。提升铁路系统电气化水平。加快老旧船舶更新改造，发展电动、液化天然气动力船舶，深入推进船舶靠港使用岸电，因地制宜开展沿海、内河绿色智能船舶示范应用。提升机场运行电动化智能化水平，发展新能源航空器。到2030年，当年新增新能源、清洁能源动力的交通工具比例达到40%左右，营运交通工具单位换算周转量碳排放强度比2020年下降9.5%左右，国家铁路单位换算周转量综合能耗比2020年下降10%。陆路交通运输石油消费力争2030年前达到峰值。

2．构建绿色高效交通运输体系。发展智能交通，推动不同运输方式合理分工、有效衔接，降低空载率和不合理客货运周转量。大力发展以铁路、水路为骨干的多式联运，推进工矿企业、港口、物流园区等铁路专用线建设，加快内河高等级航道网建设，加快大宗货物和中长距离货物运输“公转铁”、“公转水”。加快先进适用技术应用，提升民航运行管理效率，引导航空企业加强智慧运行，实现系统化节能降碳。加快城乡物流配送体系建设，创新绿色低碳、集约高效的配送模式。打造高效衔接、快捷舒适的公共交通服务体系，积极引导公众选择绿色低碳交通方式。“十四五”期间，集装箱铁水联运量年均增长15%以上。到2030年，城区常住人口100万以上的城市绿色出行比例不低于70%。

3．加快绿色交通基础设施建设。将绿色低碳理念贯穿于交通基础设施规划、建设、运营和维护全过程，降低全生命周期能耗和碳排放。开展交通基础设施绿色化提升改造，统筹利用综合运输通道线位、土地、空域等资源，加大岸线、锚地等资源整合力度，提高利用效率。有序推进充电桩、配套电网、加注（气）站、加氢站等基础设施建设，提升城市公共交通基础设施水平。到2030年，民用运输机场场内车辆装备等力争全面实现电动化。

（六）循环经济助力降碳行动。

抓住资源利用这个源头，大力发展循环经济，全面提高资源利用效率，充分发挥减少资源消耗和降碳的协同作用。

1．推进产业园区循环化发展。以提升资源产出率和循环利用率为目标，优化园区空间布局，开展园区循环化改造。推动园区企业循环式生产、产业循环式组合，组织企业实施清洁生产改造，促进废物综合利用、能量梯级利用、水资源循环利用，推进工业余压余热、废气废液废渣资源化利用，积极推广集中供气供热。搭建基础设施和公共服务共享平台，加强园区物质流管理。到2030年，省级以上重点产业园区全部实施循环化改造。

2．加强大宗固废综合利用。提高矿产资源综合开发利用水平和综合利用率，以煤矸石、粉煤灰、尾矿、共伴生矿、冶炼渣、工业副产石膏、建筑垃圾、农作物秸秆等大宗固废为重点，支持大掺量、规模化、高值化利用，鼓励应用于替代原生非金属矿、砂石等资源。在确保安全环保前提下，探索将磷石膏应用于土壤改良、井下充填、路基修筑等。推动建筑垃圾资源化利用，推广废弃路面材料原地再生利用。加快推进秸秆高值化利用，完善收储运体系，严格禁烧管控。加快大宗固废综合利用示范建设。到2025年，大宗固废年利用量达到40亿吨左右；到2030年，年利用量达到45亿吨左右。

3．健全资源循环利用体系。完善废旧物资回收网络，推行“互联网+”回收模式，实现再生资源应收尽收。加强再生资源综合利用行业规范管理，促进产业集聚发展。高水平建设现代化“城市矿产”基地，推动再生资源规范化、规模化、清洁化利用。推进退役动力电池、光伏组件、风电机组叶片等新兴产业废物循环利用。促进汽车零部件、工程机械、文办设备等再制造产业高质量发展。加强资源再生产品和再制造产品推广应用。到2025年，废钢铁、废铜、废铝、废铅、废锌、废纸、废塑料、废橡胶、废玻璃等9种主要再生资源循环利用量达到4.5亿吨，到2030年达到5.1亿吨。

4．大力推进生活垃圾减量化资源化。扎实推进生活垃圾分类，加快建立覆盖全社会的生活垃圾收运处置体系，全面实现分类投放、分类收集、分类运输、分类处理。加强塑料污染全链条治理，整治过度包装，推动生活垃圾源头减量。推进生活垃圾焚烧处理，降低填埋比例，探索适合我国厨余垃圾特性的资源化利用技术。推进污水资源化利用。到2025年，城市生活垃圾分类体系基本健全，生活垃圾资源化利用比例提升至60%左右。到2030年，城市生活垃圾分类实现全覆盖，生活垃圾资源化利用比例提升至65%。

（七）绿色低碳科技创新行动。

发挥科技创新的支撑引领作用，完善科技创新体制机制，强化创新能力，加快绿色低碳科技革命。

1．完善创新体制机制。制定科技支撑碳达峰碳中和行动方案，在国家重点研发计划中设立碳达峰碳中和关键技术研究与示范等重点专项，采取“揭榜挂帅”机制，开展低碳零碳负碳关键核心技术攻关。将绿色低碳技术创新成果纳入高等学校、科研单位、国有企业有关绩效考核。强化企业创新主体地位，支持企业承担国家绿色低碳重大科技项目，鼓励设施、数据等资源开放共享。推进国家绿色技术交易中心建设，加快创新成果转化。加强绿色低碳技术和产品知识产权保护。完善绿色低碳技术和产品检测、评估、认证体系。

2．加强创新能力建设和人才培养。组建碳达峰碳中和相关国家实验室、国家重点实验室和国家技术创新中心，适度超前布局国家重大科技基础设施，引导企业、高等学校、科研单位共建一批国家绿色低碳产业创新中心。创新人才培养模式，鼓励高等学校加快新能源、储能、氢能、碳减排、碳汇、碳排放权交易等学科建设和人才培养，建设一批绿色低碳领域未来技术学院、现代产业学院和示范性能源学院。深化产教融合，鼓励校企联合开展产学合作协同育人项目，组建碳达峰碳中和产教融合发展联盟，建设一批国家储能技术产教融合创新平台。

3．强化应用基础研究。实施一批具有前瞻性、战略性的国家重大前沿科技项目，推动低碳零碳负碳技术装备研发取得突破性进展。聚焦化石能源绿色智能开发和清洁低碳利用、可再生能源大规模利用、新型电力系统、节能、氢能、储能、动力电池、二氧化碳捕集利用与封存等重点，深化应用基础研究。积极研发先进核电技术，加强可控核聚变等前沿颠覆性技术研究。

4．加快先进适用技术研发和推广应用。集中力量开展复杂大电网安全稳定运行和控制、大容量风电、高效光伏、大功率液化天然气发动机、大容量储能、低成本可再生能源制氢、低成本二氧化碳捕集利用与封存等技术创新，加快碳纤维、气凝胶、特种钢材等基础材料研发，补齐关键零部件、元器件、软件等短板。推广先进成熟绿色低碳技术，开展示范应用。建设全流程、集成化、规模化二氧化碳捕集利用与封存示范项目。推进熔盐储能供热和发电示范应用。加快氢能技术研发和示范应用，探索在工业、交通运输、建筑等领域规模化应用。

（八）碳汇能力巩固提升行动。

坚持系统观念，推进山水林田湖草沙一体化保护和修复，提高生态系统质量和稳定性，提升生态系统碳汇增量。

1．巩固生态系统固碳作用。结合国土空间规划编制和实施，构建有利于碳达峰、碳中和的国土空间开发保护格局。严守生态保护红线，严控生态空间占用，建立以国家公园为主体的自然保护地体系，稳定现有森林、草原、湿地、海洋、土壤、冻土、岩溶等固碳作用。严格执行土地使用标准，加强节约集约用地评价，推广节地技术和节地模式。

2．提升生态系统碳汇能力。实施生态保护修复重大工程。深入推进大规模国土绿化行动，巩固退耕还林还草成果，扩大林草资源总量。强化森林资源保护，实施森林质量精准提升工程，提高森林质量和稳定性。加强草原生态保护修复，提高草原综合植被盖度。加强河湖、湿地保护修复。整体推进海洋生态系统保护和修复，提升红树林、海草床、盐沼等固碳能力。加强退化土地修复治理，开展荒漠化、石漠化、水土流失综合治理，实施历史遗留矿山生态修复工程。到2030年，全国森林覆盖率达到25%左右，森林蓄积量达到190亿立方米。

3．加强生态系统碳汇基础支撑。依托和拓展自然资源调查监测体系，利用好国家林草生态综合监测评价成果，建立生态系统碳汇监测核算体系，开展森林、草原、湿地、海洋、土壤、冻土、岩溶等碳汇本底调查、碳储量评估、潜力分析，实施生态保护修复碳汇成效监测评估。加强陆地和海洋生态系统碳汇基础理论、基础方法、前沿颠覆性技术研究。建立健全能够体现碳汇价值的生态保护补偿机制，研究制定碳汇项目参与全国碳排放权交易相关规则。

4．推进农业农村减排固碳。大力发展绿色低碳循环农业，推进农光互补、“光伏+设施农业”、“海上风电+海洋牧场”等低碳农业模式。研发应用增汇型农业技术。开展耕地质量提升行动，实施国家黑土地保护工程，提升土壤有机碳储量。合理控制化肥、农药、地膜使用量，实施化肥农药减量替代计划，加强农作物秸秆综合利用和畜禽粪污资源化利用。

（九）绿色低碳全民行动。

增强全民节约意识、环保意识、生态意识，倡导简约适度、绿色低碳、文明健康的生活方式，把绿色理念转化为全体人民的自觉行动。

1．加强生态文明宣传教育。将生态文明教育纳入国民教育体系，开展多种形式的资源环境国情教育，普及碳达峰、碳中和基础知识。加强对公众的生态文明科普教育，将绿色低碳理念有机融入文艺作品，制作文创产品和公益广告，持续开展世界地球日、世界环境日、全国节能宣传周、全国低碳日等主题宣传活动，增强社会公众绿色低碳意识，推动生态文明理念更加深入人心。

2．推广绿色低碳生活方式。坚决遏制奢侈浪费和不合理消费，着力破除奢靡铺张的歪风陋习，坚决制止餐饮浪费行为。在全社会倡导节约用能，开展绿色低碳社会行动示范创建，深入推进绿色生活创建行动，评选宣传一批优秀示范典型，营造绿色低碳生活新风尚。大力发展绿色消费，推广绿色低碳产品，完善绿色产品认证与标识制度。提升绿色产品在政府采购中的比例。

3．引导企业履行社会责任。引导企业主动适应绿色低碳发展要求，强化环境责任意识，加强能源资源节约，提升绿色创新水平。重点领域国有企业特别是中央企业要制定实施企业碳达峰行动方案，发挥示范引领作用。重点用能单位要梳理核算自身碳排放情况，深入研究碳减排路径，“一企一策”制定专项工作方案，推进节能降碳。相关上市公司和发债企业要按照环境信息依法披露要求，定期公布企业碳排放信息。充分发挥行业协会等社会团体作用，督促企业自觉履行社会责任。

4．强化领导干部培训。将学习贯彻习近平生态文明思想作为干部教育培训的重要内容，各级党校（行政学院）要把碳达峰、碳中和相关内容列入教学计划，分阶段、多层次对各级领导干部开展培训，普及科学知识，宣讲政策要点，强化法治意识，深化各级领导干部对碳达峰、碳中和工作重要性、紧迫性、科学性、系统性的认识。从事绿色低碳发展相关工作的领导干部要尽快提升专业素养和业务能力，切实增强推动绿色低碳发展的本领。

（十）各地区梯次有序碳达峰行动。

各地区要准确把握自身发展定位，结合本地区经济社会发展实际和资源环境禀赋，坚持分类施策、因地制宜、上下联动，梯次有序推进碳达峰。

1．科学合理确定有序达峰目标。碳排放已经基本稳定的地区要巩固减排成果，在率先实现碳达峰的基础上进一步降低碳排放。产业结构较轻、能源结构较优的地区要坚持绿色低碳发展，坚决不走依靠“两高”项目拉动经济增长的老路，力争率先实现碳达峰。产业结构偏重、能源结构偏煤的地区和资源型地区要把节能降碳摆在突出位置，大力优化调整产业结构和能源结构，逐步实现碳排放增长与经济增长脱钩，力争与全国同步实现碳达峰。

2．因地制宜推进绿色低碳发展。各地区要结合区域重大战略、区域协调发展战略和主体功能区战略，从实际出发推进本地区绿色低碳发展。京津冀、长三角、粤港澳大湾区等区域要发挥高质量发展动力源和增长极作用，率先推动经济社会发展全面绿色转型。长江经济带、黄河流域和国家生态文明试验区要严格落实生态优先、绿色发展战略导向，在绿色低碳发展方面走在全国前列。中西部和东北地区要着力优化能源结构，按照产业政策和能耗双控要求，有序推动高耗能行业向清洁能源优势地区集中，积极培育绿色发展动能。

3．上下联动制定地方达峰方案。各省、自治区、直辖市人民政府要按照国家总体部署，结合本地区资源环境禀赋、产业布局、发展阶段等，坚持全国一盘棋，不抢跑，科学制定本地区碳达峰行动方案，提出符合实际、切实可行的碳达峰时间表、路线图、施工图，避免“一刀切”限电限产或运动式“减碳”。各地区碳达峰行动方案经碳达峰碳中和工作领导小组综合平衡、审核通过后，由地方自行印发实施。

4．组织开展碳达峰试点建设。加大中央对地方推进碳达峰的支持力度，选择100个具有典型代表性的城市和园区开展碳达峰试点建设，在政策、资金、技术等方面对试点城市和园区给予支持，加快实现绿色低碳转型，为全国提供可操作、可复制、可推广的经验做法。

四、国际合作

（一）深度参与全球气候治理。大力宣传习近平生态文明思想，分享中国生态文明、绿色发展理念与实践经验，为建设清洁美丽世界贡献中国智慧、中国方案、中国力量，共同构建人与自然生命共同体。主动参与全球绿色治理体系建设，坚持共同但有区别的责任原则、公平原则和各自能力原则，坚持多边主义，维护以联合国为核心的国际体系，推动各方全面履行《联合国气候变化框架公约》及其《巴黎协定》。积极参与国际航运、航空减排谈判。

（二）开展绿色经贸、技术与金融合作。优化贸易结构，大力发展高质量、高技术、高附加值绿色产品贸易。加强绿色标准国际合作，推动落实合格评定合作和互认机制，做好绿色贸易规则与进出口政策的衔接。加强节能环保产品和服务进出口。加大绿色技术合作力度，推动开展可再生能源、储能、氢能、二氧化碳捕集利用与封存等领域科研合作和技术交流，积极参与国际热核聚变实验堆计划等国际大科学工程。深化绿色金融国际合作，积极参与碳定价机制和绿色金融标准体系国际宏观协调，与有关各方共同推动绿色低碳转型。

（三）推进绿色“一带一路”建设。秉持共商共建共享原则，弘扬开放、绿色、廉洁理念，加强与共建“一带一路”国家的绿色基建、绿色能源、绿色金融等领域合作，提高境外项目环境可持续性，打造绿色、包容的“一带一路”能源合作伙伴关系，扩大新能源技术和产品出口。发挥“一带一路”绿色发展国际联盟等合作平台作用，推动实施《“一带一路”绿色投资原则》，推进“一带一路”应对气候变化南南合作计划和“一带一路”科技创新行动计划。

五、政策保障

（一）建立统一规范的碳排放统计核算体系。加强碳排放统计核算能力建设，深化核算方法研究，加快建立统一规范的碳排放统计核算体系。支持行业、企业依据自身特点开展碳排放核算方法学研究，建立健全碳排放计量体系。推进碳排放实测技术发展，加快遥感测量、大数据、云计算等新兴技术在碳排放实测技术领域的应用，提高统计核算水平。积极参与国际碳排放核算方法研究，推动建立更为公平合理的碳排放核算方法体系。

（二）健全法律法规标准。构建有利于绿色低碳发展的法律体系，推动能源法、节约能源法、电力法、煤炭法、可再生能源法、循环经济促进法、清洁生产促进法等制定修订。加快节能标准更新，修订一批能耗限额、产品设备能效强制性国家标准和工程建设标准，提高节能降碳要求。健全可再生能源标准体系，加快相关领域标准制定修订。建立健全氢制、储、输、用标准。完善工业绿色低碳标准体系。建立重点企业碳排放核算、报告、核查等标准，探索建立重点产品全生命周期碳足迹标准。积极参与国际能效、低碳等标准制定修订，加强国际标准协调。

（三）完善经济政策。各级人民政府要加大对碳达峰、碳中和工作的支持力度。建立健全有利于绿色低碳发展的税收政策体系，落实和完善节能节水、资源综合利用等税收优惠政策，更好发挥税收对市场主体绿色低碳发展的促进作用。完善绿色电价政策，健全居民阶梯电价制度和分时电价政策，探索建立分时电价动态调整机制。完善绿色金融评价机制，建立健全绿色金融标准体系。大力发展绿色贷款、绿色股权、绿色债券、绿色保险、绿色基金等金融工具，设立碳减排支持工具，引导金融机构为绿色低碳项目提供长期限、低成本资金，鼓励开发性政策性金融机构按照市场化法治化原则为碳达峰行动提供长期稳定融资支持。拓展绿色债券市场的深度和广度，支持符合条件的绿色企业上市融资、挂牌融资和再融资。研究设立国家低碳转型基金，支持传统产业和资源富集地区绿色转型。鼓励社会资本以市场化方式设立绿色低碳产业投资基金。

（四）建立健全市场化机制。发挥全国碳排放权交易市场作用，进一步完善配套制度，逐步扩大交易行业范围。建设全国用能权交易市场，完善用能权有偿使用和交易制度，做好与能耗双控制度的衔接。统筹推进碳排放权、用能权、电力交易等市场建设，加强市场机制间的衔接与协调，将碳排放权、用能权交易纳入公共资源交易平台。积极推行合同能源管理，推广节能咨询、诊断、设计、融资、改造、托管等“一站式”综合服务模式。

六、组织实施

（一）加强统筹协调。加强党中央对碳达峰、碳中和工作的集中统一领导，碳达峰碳中和工作领导小组对碳达峰相关工作进行整体部署和系统推进，统筹研究重要事项、制定重大政策。碳达峰碳中和工作领导小组成员单位要按照党中央、国务院决策部署和领导小组工作要求，扎实推进相关工作。碳达峰碳中和工作领导小组办公室要加强统筹协调，定期对各地区和重点领域、重点行业工作进展情况进行调度，科学提出碳达峰分步骤的时间表、路线图，督促将各项目标任务落实落细。

（二）强化责任落实。各地区各有关部门要深刻认识碳达峰、碳中和工作的重要性、紧迫性、复杂性，切实扛起责任，按照《中共中央　国务院关于完整准确全面贯彻新发展理念做好碳达峰碳中和工作的意见》和本方案确定的主要目标和重点任务，着力抓好各项任务落实，确保政策到位、措施到位、成效到位，落实情况纳入中央和省级生态环境保护督察。各相关单位、人民团体、社会组织要按照国家有关部署，积极发挥自身作用，推进绿色低碳发展。

（三）严格监督考核。实施以碳强度控制为主、碳排放总量控制为辅的制度，对能源消费和碳排放指标实行协同管理、协同分解、协同考核，逐步建立系统完善的碳达峰碳中和综合评价考核制度。加强监督考核结果应用，对碳达峰工作成效突出的地区、单位和个人按规定给予表彰奖励，对未完成目标任务的地区、部门依规依法实行通报批评和约谈问责。各省、自治区、直辖市人民政府要组织开展碳达峰目标任务年度评估，有关工作进展和重大问题要及时向碳达峰碳中和工作领导小组报告。

国务院

2021年10月24日

**P26**

**Notice on Publishing Typical Cases of New Technologies and Products for Intelligent Construction (First Batch)**

**住房和城乡建设部办公厅关于发布智能建造新技术新产品创新服务典型案例（第一批）的通知**

建办市函〔2021〕482号

各省、自治区住房和城乡建设厅，直辖市住房和城乡建设（管）委，北京市规划和自然资源委，新疆生产建设兵团住房和城乡建设局：

　　按照《住房和城乡建设部等部门关于推动智能建造与建筑工业化协同发展的指导意见》（建市〔2020〕60号）要求，为总结推广智能建造可复制经验做法，指导各地住房和城乡建设主管部门和企业全面了解、科学选用智能建造技术和产品，经企业申报、地方推荐、专家评审，确定124个案例为第一批智能建造新技术新产品创新服务典型案例（案例集可在住房和城乡建设部门户网站上查询）。现予以发布，请结合实际学习借鉴。

　　　　　　　　　　　　　　　　　　　　　　　　　　　　　　　　　　　　　　　　　　　　　　　　　住房和城乡建设部办公厅　　　　　　　　　　　　　　　　　　　　　　　 2021年11月22日

　　（此件主动公开）

**P27**

**"14th Five-Year" Development Plan for the Construction Industry**

**“十四五”建筑业发展规划**

建市〔2022〕11号

本规划根据《中华人民共和国国民经济和社会发展第十 四个五年规划和 2035 年远景目标纲要》编制，主要阐明“十 四五” 时期建筑业发展的战略方向，明确发展目标和主要任务，是行业发展的指导性文件。

一、总体要求

（ 一）规划背景。

“十三五”期间，我国建筑业改革发展成效显著，全国 建筑业增加值年均增长 5.1%， 占国内生产总值比重保持在 6.9%以上，建筑企业签订合同额年均增长 12.5%，勘察设计 企业营业收入年均增长 24.1%，工程监理、造价咨询、招标 代理等工程咨询服务企业营业收入年均增长均超过 15%。 2020 年，全国建筑业总产值达 26.39 万亿元，实现增加值 7.2 万亿元， 占国内生产总值比重达到 7.1%，房屋施工面积 149.47 亿平方米，建筑业从业人数 5366 万人。建筑业作为 国民经济支柱产业的作用不断增强，为促进经济增长、缓解 社会就业压力、推进新型城镇化建设、保障和改善人民生活、决胜全面建成小康社会作出了重要贡献。在取得成绩的同时，建筑业依然存在发展质量和效益不 高的问题，集中表现为发展方式粗放、劳动生产率低、高耗 能高排放、市场秩序不规范、建筑品质总体不高、工程质量 安全事故时有发生等，与人民群众日益增长的美好生活需要相比仍有一定差距。

“十四五” 时期是新发展阶段的开局起步期，是实施城 市更新行动、推进新型城镇化建设的机遇期，也是加快建筑 业转型发展的关键期。一方面，建筑市场作为我国超大规模 市场的重要组成部分，是构建新发展格局的重要阵地，在与 先进制造业、新一代信息技术深度融合发展方面有着巨大的 潜力和发展空间。另一方面，我国城市发展由大规模增量建 设转为存量提质改造和增量结构调整并重，人民群众对住房 的要求从有没有转向追求好不好，将为建筑业提供难得的转 型发展机遇。建筑业迫切需要树立新发展思路，将扩大内需 与转变发展方式有机结合起来，同步推进，从追求高速增长 转向追求高质量发展，从 “量”的扩张转向 “质”的提升，走出一条内涵集约式发展新路。

（ 二）指导思想。

以习近平新时代中国特色社会主义思想为指导，深入贯 彻党的十九大和十九届历次全会精神，立足新发展阶段，完 整、准确、全面贯彻新发展理念，构建新发展格局，坚持稳 中求进工作总基调， 以推动建筑业高质量发展为主题， 以深 化供给侧结构性改革为主线，以推动智能建造与新型建筑工 业化协同发展为动力，加快建筑业转型升级，实现绿色低碳 发展，切实提高发展质量和效益，不断满足人民群众对美好 生活的需要，为开启全面建设社会主义现代化国家新征程奠定坚实基础。

（三）基本原则。

——坚持统筹谋划，系统推进。坚持问题导向、 目标导 向和结果导向，对标“十四五”时期经济社会发展目标和 2035 年远景目标，落实碳达峰、碳中和目标任务，加强前瞻性研 究、全局性谋划和战略性布局，明确建筑业改革发展方向和 目标任务，坚持整体推进与重点突破相结合，着力构建行业发展新格局。

——坚持市场主导，政府引导。持续完善建筑业管理体 制机制，建设高标准建筑市场体系，深入推进 “放管服” 改 革，进一步优化营商环境，充分发挥市场在资源配置中的决 定性作用，更好发挥政府作用，有效激发建筑市场各方主体活力。

——坚持创新驱动，绿色发展。推广绿色化、工业化、 信息化、集约化、产业化建造方式，推动新一代信息技术与 建筑业深度融合，积极培育新产品、新业态、新模式，减少 材料和能源消耗，降低建造过程碳排放量，实现更高质量、更有效率、更加公平、更可持续的发展。

——坚持质量第一，安全为本。统筹发展与安全，坚持 人民至上、生命至上，坚决把质量安全作为行业发展的生命 线， 以数字化赋能为支撑， 以信用管理为抓手，健全工程质 量安全管理机制，强化政府监管作用，防范化解重大质量安全风险，着力提升建筑品质，不断增强人民群众获得感。

二、发展目标

（一）2035 年远景目标。

以建设世界建造强国为目标，着力构建市场机制有效、 质量安全可控、标准支撑有力、市场主体有活力的现代化建 筑业发展体系。到 2035 年，建筑业发展质量和效益大幅提 升，建筑工业化全面实现，建筑品质显著提升，企业创新能 力大幅提高，高素质人才队伍全面建立，产业整体优势明显 增强， “ 中国建造”核心竞争力世界领先，迈入智能建造世

界强国行列，全面服务社会主义现代化强国建设。

（二） “十四五”时期发展目标。

对标 2035 年远景目标，初步形成建筑业高质量发展体 系框架，建筑市场运行机制更加完善，营商环境和产业结构 不断优化，建筑市场秩序明显改善，工程质量安全保障体系 基本健全，建筑工业化、数字化、智能化水平大幅提升，建 造方式绿色转型成效显著，加速建筑业由大向强转变，为形成强大国内市场、构建新发展格局提供有力支撑。

—— 国民经济支柱产业地位更加稳固。高质量完成全社 会固定资产投资建设任务，全国建筑业总产值年均增长率保 持在合理区间，建筑业增加值占国内生产总值的比重保持在 6%左右。新一代信息技术与建筑业实现深度融合，催生一批新产品新业态新模式，壮大经济发展新引擎。

——产业链现代化水平明显提高。智能建造与新型建筑

工业化协同发展的政策体系和产业体系基本建立，装配式建 筑占新建建筑的比例达到 30%以上，打造一批建筑产业互联 网平台，形成一批建筑机器人标志性产品，培育一批智能建造和装配式建筑产业基地。

——绿色低碳生产方式初步形成。绿色建造政策、技术、 实施体系初步建立，绿色建造方式加快推行，工程建设集约 化水平不断提高，新建建筑施工现场建筑垃圾排放量控制在 每万平方米 300 吨以下，建筑废弃物处理和再利用的市场机制初步形成，建设一批绿色建造示范工程。

——建筑市场体系更加完善。建筑法修订加快推进，法 律法规体系更加完善。企业资质管理制度进一步完善，个人 执业资格管理进一步强化，工程担保和信用管理制度不断健 全，工程造价市场化机制初步形成。工程建设组织模式持续 优化，工程总承包和全过程工程咨询广泛推行。符合建筑业 特点的用工方式基本建立，建筑工人实现公司化、专业化管理， 中级工以上建筑工人达 1000 万人以上。

——工程质量安全水平稳步提升。建筑品质和使用功能 不断提高，建筑施工安全生产形势持续稳定向好，重特大安 全生产事故得到有效遏制。建设工程消防设计审查和验收平 稳有序开展。城市轨道交通工程智慧化建设初具成效。工程 抗震防灾能力稳步提升。质量安全技术创新和应用水平不断提高。

三、主要任务

（ 一）加快智能建造与新型建筑工业化协同发展。

1.完善智能建造政策和产业体系。

实施智能建造试点示范创建行动，发展一批试点城市， 建设一批示范项目，总结推广可复制政策机制。加强基础共 性和关键核心技术研发，构建先进适用的智能建造标准体 系。发布智能建造新技术新产品创新服务典型案例，编制智 能建造白皮书，推广数字设计、智能生产和智能施工。培育 智能建造产业基地，加快人才队伍建设，形成涵盖科研、设 计、生产加工、施工装配、运营等全产业链融合一体的智能建造产业体系。

2.夯实标准化和数字化基础。

完善模数协调、构件选型等标准，建立标准化部品部件 库，推进建筑平面、立面、部品部件、接口标准化，推广少 规格、多组合设计方法，实现标准化和多样化的统一。加快 推进建筑信息模型（BIM）技术在工程全寿命期的集成应用， 健全数据交互和安全标准，强化设计、生产、施工各环节数字化协同，推动工程建设全过程数字化成果交付和应用。

| 专栏 1 BIM 技术集成应用 |
| --- |
| 2025 年，基本形成 BIM 技术框架和标准体系。  1.推进自主可控 BIM 软件研发。积极引导培育一批 BIM 软件开发  骨干企业和专业人才，保障信息安全。  2.完善 BIM 标准体系。加快编制数据接口、信息交换等标准，推 进 BIM 与生产管理系统、工程管理信息系统、建筑产业互联网平台的  一体化应用。  3.引导企业建立 BIM 云服务平台。推动信息传递云端化，实现设  计、生产、施工环节数据共享。  4.建立基于 BIM 的区域管理体系。研究利用 BIM 技术进行区域管 理的标准、导则和平台建设要求，建立应用场景，在新建区域探索建 立单个项目建设与区域管理融合的新模式，在既有建筑区域探索基于  现状的快速建模技术。  5.开展 BIM 报建审批试点。完善 BIM 报建审批标准，建立 BIM 辅 助审查审批的信息系统，推进 BIM 与城市信息模型（CIM）平台融通  联动，提高信息化监管能力。 |
|  |

3.推广数字化协同设计。

应用数字化手段丰富方案创作方法，提高建筑设计方案 创作水平。鼓励大型设计企业建立数字化协同设计平台，推 进建筑、结构、设备管线、装修等一体化集成设计，提高各 专业协同设计能力。完善施工图设计文件编制深度要求，提 升精细化设计水平，为后续精细化生产和施工提供基础。研 发利用参数化、生成式设计软件，探索人工智能技术在设计 中应用。研究应用岩土工程勘测信息挖掘、集成技术和方法，推进勘测过程数字化。

4.大力发展装配式建筑。

构建装配式建筑标准化设计和生产体系，推动生产和施工智能化升级，扩大标准化构件和部品部件使用规模，提高装配式建筑综合效益。完善适用不同建筑类型装配式混凝土 建筑结构体系，加大高性能混凝土、高强钢筋和消能减震、 预应力技术集成应用。完善钢结构建筑标准体系，推动建立 钢结构住宅通用技术体系，健全钢结构建筑工程计价依据， 以标准化为主线引导上下游产业链协同发展。积极推进装配 化装修方式在商品住房项目中的应用，推广管线分离、一体 化装修技术，推广集成化模块化建筑部品，促进装配化装修 与装配式建筑深度融合。大力推广应用装配式建筑，积极推 进高品质钢结构住宅建设，鼓励学校、医院等公共建筑优先采用钢结构。培育一批装配式建筑生产基地。

5.打造建筑产业互联网平台。

加大建筑产业互联网平台基础共性技术攻关力度，编制 关键技术标准、发展指南和白皮书。开展建筑产业互联网平 台建设试点，探索适合不同应用场景的系统解决方案，培育 一批行业级、企业级、项目级建筑产业互联网平台，建设政 府监管平台。鼓励建筑企业、互联网企业和科研院所等开展 合作，加强物联网、大数据、云计算、人工智能、 区块链等新一代信息技术在建筑领域中的融合应用。

| 专栏 2 建筑产业互联网平台建设 |
| --- |
| 2025 年，建筑产业互联网平台体系初步形成，培育一批行业级、企业级、项目级平台和政府监管平台。  1.加快建设行业级平台。围绕部品部件生产采购配送、工程机械设备租赁、建筑劳务用工、装饰装修等重点领域推进行业级建筑产业互联网平台建设，提高供应链协同水平，推动资源高效配置。 |

| 2.积极培育企业级平台。发挥龙头企业示范引领作用，以企业资 源计划（ERP）平台为基础，建设企业级建筑产业互联网平台，实现  企业资源集约调配和智能决策，提升企业运营管理效益。  3.研发应用项目级平台。以智慧工地建设为载体推广项目级建筑 产业互联网平台，运用信息化手段解决施工现场实际问题，强化关键  环节质量安全管控，提升工程项目建设管理水平。  4.探索建设政府监管平台。完善全国建筑市场监管公共服务平 台，推动各地研发基于建筑产业互联网平台的政府监管平台，汇聚整 合建筑业大数据资源，支撑市场监测和数据分析功能，探索建立大数  据辅助科学决策和市场监管的机制。 |
| --- |

6.加快建筑机器人研发和应用。

加强新型传感、智能控制和优化、多机协同、人机协作 等建筑机器人核心技术研究，研究编制关键技术标准，形成 一批建筑机器人标志性产品。积极推进建筑机器人在生产、 施工、维保等环节的典型应用，重点推进与装配式建筑相配 套的建筑机器人应用,辅助和替代 “危、繁、脏、重”施工 作业。推广智能塔吊、智能混凝土泵送设备等智能化工程设备，提高工程建设机械化、智能化水平。

| 专栏 3 建筑机器人研发应用 |
| --- |
| 2025 年，形成一批建筑机器人标志性产品，实现部分领域批量化应用。  1.推广部品部件生产机器人。以混凝土预制构件制作、钢构件下料焊接、隔墙板和集成厨卫生产等工厂生产关键工艺环节为重点，推进建筑机器人创新应用。 |

| 2.加快研发施工机器人。以测量、材料配送、钢筋加工、混凝土 浇筑、构部件安装、楼面墙面装饰装修、高空焊接、深基坑施工等现  场施工环节为重点，加快建筑机器人研发应用。  3.积极探索运维机器人。在建筑安全监测、安防巡检、高层建筑  清洁等运维环节，加强建筑机器人应用场景探索。 |
| --- |

7.推广绿色建造方式。

持续深化绿色建造试点工作，提炼可复制推广经验。开 展绿色建造示范工程创建行动，提升工程建设集约化水平， 实现精细化设计和施工。培育绿色建造创新中心，加快推进 关键核心技术攻关及产业化应用。研究建立绿色建造政策、 技术、实施体系， 出台绿色建造技术导则和计价依据，构建 覆盖工程建设全过程的绿色建造标准体系。在政府投资工程 和大型公共建筑中全面推行绿色建造。积极推进施工现场建 筑垃圾减量化，推动建筑废弃物的高效处理与再利用，探索 建立研发、设计、建材和部品部件生产、施工、资源回收再利用等一体化协同的绿色建造产业链。

| 专栏 4 建筑垃圾减量化 |
| --- |
| 2025 年，各地区建筑垃圾减量化工作机制进一步完善，实现新 建建筑施工现场建筑垃圾（不包括工程渣土、工程泥浆）排放量每万平方米不高于 300 吨，其中装配式建筑排放量不高于 200 吨。  1.完善制度和标准体系。构建依法治废、源头减量、资源利用制度体系和建筑垃圾分类、收集、统计、处置及再生利用标准体系。探索建立施工现场建筑垃圾排放量公示制度，研究建筑垃圾资源化产品准入与保障机制。 |

| 2.推动技术和管理创新。支持开展建筑垃圾减量化技术和管理创 新研究，打造一批技术转化平台，形成基础研究、技术攻关、成果产业化的建筑垃圾治理全过程创新生态链。  3.提升建筑垃圾信息化管理水平。引导和推广建立建筑垃圾管理 平台。构建全程覆盖、精细高效的监管体系，实现建筑垃圾可量化、可追踪的全过程闭合管理。 |
| --- |

（ 二）健全建筑市场运行机制。

1.加强建筑市场信用体系建设。

完善建筑市场信用管理政策体系，构建以信用为基础的 新型建筑市场监管机制。完善全国建筑市场监管公共服务平 台，加强对行政许可、行政处罚、工程业绩、质量安全事故、 监督检查、评奖评优等信息的归集和共享，全面记录建筑市 场各方主体信用行为。推进部门间信用信息共享，鼓励社会 组织及第三方机构参与信用信息归集，丰富和完善建筑市场 主体信用档案。实行信用信息分级分类管理，加强信用信息 在政府采购、招标投标、行政审批、市场准入等事项中应用， 根据市场主体信用情况实施差异化监管。加大对违法发包、 转包、违法分包、资质资格挂靠等违法违规行为的查处力度， 完善和实施建筑市场主体 “黑名单”制度，开展失信惩戒，持续规范建筑市场秩序。

| 专栏 5 全国建筑市场监管公共服务平台建设  2025 年，基本形成覆盖建筑业的 “互联网+政务服务”和 “互联网+监管”体系，对接支撑建筑产业互联网平台。  1.推进行业数据互联共享。统一数据标准，打通数据壁垒。积极 应用 BIM、物联网、区块链等先进信息技术，加强政府监管数据和市场主体行为数据的归集共享，基本建成建筑业基础数据库。 |
| --- |
| 2.提升政务服务质量。全面推行施工许可电子证照、消防设计审 查验收电子证照，加快推广应用企业资质证书、人员注册执业证书电 子证照。在全国范围内推进各类电子证照信息的归集共享，为相关政务服务事项提供技术支撑，推动实现 “数据多跑路、群众少跑腿”。  3.创新信用监管模式。完善建筑市场信用信息数据库，加强对行 政许可、行政处罚、工程业绩、评奖评优等信息的归集共享，建立完 善建筑市场主体信用档案。实行信用信息分级分类管理，加大信用信 息公开力度，推进信用信息科学规范应用。探索建立大数据辅助监管和决策的机制，提升政府数字化监管能力。 |

2.深化招标投标制度改革。

完善招标投标制度体系，进一步扩大招标人自主权，强 化招标人首要责任。鼓励有条件的地区政府投资工程按照建 设、使用分离的原则，实施相对集中专业化管理。优化评标 方法，将投标人信用情况和工程质量安全情况作为评标重要 指标，优先选择符合绿色发展要求的投标方案。积极推行采 用 “评定分离”方法确定中标人。完善设计咨询服务委托和 计费模式，推广采用团队招标方式选择设计单位，探索设计服务市场化人工时计价模式，根据设计服务内容、深度和质量合理确定设计服务价格，推动实现“按质择优、优质优价”。 全面推行招标投标交易全过程电子化和异地远程评标，加大 招标投标活动信息公开力度，加快推动交易、监管数据互联 共享。规范招标投标异议投诉处理工作，强化事中事后监管， 依法严肃查处规避招标、 串通投标、弄虚作假等违法违规行 为，及时纠正通过设立不合理条件限制或排斥外地企业承揽业务的做法，形成统一开放、竞争有序的市场环境。

3.完善企业资质管理制度。

深化建设工程企业资质管理制度改革，修订出台企业资 质管理规定和标准，大幅压减企业资质类别和等级，放宽建 筑市场准入限制。下放企业资质审批权限，推行企业资质审 批告知承诺制和企业资质证书电子证照，简化各类证明事 项，实现企业资质审批 “ 一网通办”。加强企业资质与质量 安全的联动管理，实行 “ 一票否决”制，对发生质量安全事 故的企业依法从严处罚，并在一定期限内不批准其资质申 请。充分利用信息化手段加强资质审批后动态监管，将违法 违规行为、质量安全问题多发或存在重大质量安全隐患的企业列为重点核查对象，不符合资质标准要求的依法撤回。

4.强化个人执业资格管理。

完善注册建筑师、勘察设计注册工程师、注册建造师、 注册监理工程师和注册造价工程师管理制度，进一步明确注册人员权利、义务和责任。推进职业资格考试、注册、执业、继续教育等制度改革，推行注册执业证书电子证照。提高注 册人员执业实践能力，严格执行执业签字制度，探索建立个 人执业保险制度，规范执业行为。在部分地区探索实行注册 人员执业行为扣分制，扣分达到一定数量后限制执业并接受 继续教育。 弘扬职业精神，提升注册人员的专业素养和社会责任感。

5.推行工程担保制度。

加快推行投标担保、履约担保、工程质量保证担保和农 民工工资支付担保，提升各类保证金的保函替代率。加快推 行银行保函制度，探索工程担保公司保函和工程保证保险。 落实建设单位工程款支付担保制度。大力推行电子保函，研究制定保函示范文本和电子保函数据标准，加大保函信息公开力度。

6.完善工程监理制度。

进一步夯实监理责任，明确职责范围，提高监理能力， 整顿规范监理市场，优化市场环境。鼓励监理企业参与城市 更新行动、新型城镇化建设、高品质绿色建筑建设。鼓励监 理企业通过政府购买服务方式参与工程质量安全监督检查， 强化工程监理在质量安全管理方面的作用。在铁路工程等领 域推广重大工程建设项目监理向政府报告工作制度。推进监 理行业标准化、信息化建设，组织行业协会、监理企业研究制定工程监理相关团体标准、企业标准和示范文本，推进 BIM技术、物联网、人工智能等现代信息技术在工程监理中的融合应用。

7.深化工程造价改革。

完善工程计价依据体系，从国情出发，借鉴国际做法， 改进工程计量和计价规则，优化计价依据编制、发布和动态 管理机制，更加适应市场化需要。搭建市场价格信息发布平 台，鼓励企事业单位和行业协会通过平台发布人工、材料、 机械等市场价格信息，进一步完善工程造价市场形成机制。 加快建立国有资金投资工程造价数据库，加强工程造价数据 积累，为相关工程概预算编制提供依据。强化建设单位造价 管控责任，严格施工合同履约管理，全面推行施工过程价款 结算和支付。完善造价咨询行业监管制度，构建政府主导、企业自治、行业自律、社会监督的协同监管新格局。

（三）完善工程建设组织模式。

1.推广工程总承包模式。

加快完善工程总承包相关的招标投标、工程计价、合同 管理等制度规定，落实工程总承包单位工程设计、施工主体 责任。 以装配式建筑为重点，鼓励和引导建设内容明确、技 术方案成熟的工程项目优先采用工程总承包模式。支持工程 总承包单位做优做强、专业承包单位做精做专，提高工程总 承包单位项目管理、资源配置、风险管控等综合服务能力，进一步延伸融资、运行维护服务。在工程总承包项目中推进全过程 BIM 技术应用，促进技术与管理、设计与施工深度融 合。鼓励建设单位根据实施效益对工程总承包单位给予奖励。

2.发展全过程工程咨询服务。

加快建立全过程工程咨询服务交付标准、工作流程、合 同体系和管理体系，明确权责关系，完善服务酬金计取方式。 发展涵盖投资决策、工程建设、运营等环节的全过程工程咨 询服务模式，鼓励政府投资项目和国有企业投资项目带头推 行。培养一批具有国际竞争力的全过程工程咨询企业和领军人才。

3.推行建筑师负责制。

在民用建筑工程项目中推行建筑师负责制，在统筹协调 设计阶段各专业和环节基础上，推行建筑师负责工程建设全 过程管理和服务。 出台推行建筑师负责制指导意见，完善委 托发包方式、服务标准、合同示范文本以及个人执业保险等 配套制度。依据合同赋予建筑师代表建设单位签发指令和认 可工程的权利，明确建筑师相应的设计主体责任和咨询管理 责任，更好发挥建筑师对建筑品质管控作用。拓展设计咨询 服务链条，促进工程设计咨询服务向专业化和价值链高端延 伸。探索建立建筑前策划、后评估制度，优化项目前期技术 策划，对已使用建筑的功能、效益、环境影响等进行综合评估，强化设计引领作用。

（四）培育建筑产业工人队伍。

1.改革建筑劳务用工制度。

鼓励建筑企业通过培育自有建筑工人、吸纳高技能技术 工人和职业院校毕业生等方式，建立相对稳定的核心技术工 人队伍。 引导小微型劳务企业向专业作业企业转型发展，进 一步做专做精。制定建筑工人职业技能标准和评价规范，推行终身职业技能培训制度。推动大型建筑业央企与高职院校 合作办学，建设建筑产业工人培育基地，加强技能培训。推 动各地制定施工现场技能工人基本配备标准，推行装配式建 筑灌浆工、构件装配工、钢结构吊装工等特殊工种持证上岗。 完善建筑职业（工种）人工价格市场化信息发布机制，引导 建筑企业将建筑工人薪酬与技能等级挂钩。全面落实建筑工人劳动合同制度。

2.加强建筑工人实名制管理。

完善全国建筑工人管理服务信息平台，充分运用物联 网、生物识别、 区块链等新一代信息技术，实现建筑工人实 名制、劳动合同、培训记录与考核评价、作业绩效与评价等 方面的信息化管理。制定统一数据标准，加强各系统平台间 数据对接互认，实现全国数据互联共享。将建筑工人管理数 据与日常监管相结合，加强数据分析应用，提升监管效能。 在建筑工人实名制管理的基础上，加强管理人员到岗履职监管，严格实行特种作业人员实名上岗，压实现场管理和技术人员责任。

3.保障建筑工人合法权益。

健全保障建筑工人薪酬支付的长效机制，落实工资保证 金、工资专用账户管理等制度，推行分包单位农民工工资委 托施工总承包单位代发制度。完善建筑工人社会保险缴费机 制，保障职业安全和健康权益。落实施工现场生活环境、劳 动保护和作业环境基本配置，持续改善建筑工人生产生活环 境。鼓励有条件的企业按照国家规定进行上岗前、在岗期间和离岗时的职业健康检查。

（五）完善工程质量安全保障体系。

1.提升工程建设标准水平。

完善建筑工程质量标准体系，提高安全标准，强化工程 质量保障的标准化措施。进一步完善建筑性能标准，合理确 定节能、室内外环境质量、无障碍、适老化等建筑品质指标。 研究制订绿色建筑设计、施工、运行维护标准体系，完善既有建筑绿色改造技术及评价标准，编制超低能耗、近零能耗建筑相关标准。

2.落实工程质量安全责任。

全面落实工程建设各方主体及项目负责人质量安全责 任，进一步明确责任边界，构建以建设单位为首要责任的质 量安全主体责任体系。完善责任追溯机制，加大质量安全责任追究力度，依法依规严肃事故查处。严格执行工程质量终

身责任制，落实法定代表人授权书、质量终身责任承诺书和 永久性标牌制度。研究制定施工安全风险防控和重大隐患排 查治理标准，建立健全双重预防工作机制。完善安全生产许可证制度，探索推行 “全国一证、分省管理”方式。

3.全面提高工程质量安全监管水平。

健全工程质量安全监督机制，完善省、市、县三级监管 体系，厘清层级监管职责，严格落实监管责任。依托全国工 程质量安全监管平台和地方各级监管平台，大力推进 “互联 网+监管”，充分运用大数据、云计算等信息化手段和差异 化监督方式，实现 “智慧”监督。完善质量安全监管和执法 衔接机制，提高精准执法和服务水平。加强工程质量安全监 督队伍建设，加大专业人员培训力度，强化层级监督考核机 制，提升监督队伍标准化、专业化水平。组织开展全国工程 质量检测行业专项治理行动，规范检测市场秩序，依法严厉打击弄虚作假等违法违规行为。

| 专栏 6 预拌混凝土质量专项治理  2025 年，预拌混凝土管理法规制度更加完备，预拌混凝土质量总体可控、稳中有升。 |
| --- |
| 1.组织开展预拌混凝土质量专项抽查。依法严厉查处预拌混凝土质量不合格、违规使用或检测数据造假等违法违规行为。  2.完善预拌混凝土管理制度。健全预拌混凝土生产、运输和使用环节质量管理机制。  3.完善预拌混凝土相关标准。研究制定混凝土结构通用规范和机制砂混凝土应用技术规范，修订预拌混凝土产品标准。 |

| 4.组织开展违规海砂排查整治行动。指导地方严厉打击违规使用海砂等行为。 |
| --- |

| 专栏 7 危险性较大的分部分项工程专项治理 |
| --- |
| 2025 年，重大安全风险管控和隐患排查治理机制更加健全，安 全生产责任体系更加完善，安全科技支撑能力显著增强，施工安全事故得到有效遏制。  1.制定《危险性较大的分部分项工程专项施工方案编制指南》， 加强专项施工方案编制、审核、论证、实施环节突出问题整治，严厉打击可能导致群死群伤事故的严重违法违规行为。  2.鼓励推行建筑起重机械租赁、安拆、使用、维护一体化管理模式，进一步压实建筑起重机械各环节安全生产责任。  3.加大危险性较大的分部分项工程领域安全技术和信息化技术研发推广，实施 “机械化换人、 自动化减人”，消除重大隐患。 |

4.构建工程质量安全治理新局面。

加快工程质量安全信用体系建设，进一步健全质量安全

信用信息归集、公开制度，加大守信激励和失信惩戒力度。

完善安全生产处罚机制，严格落实安全生产事故“一票否决” 制度。大力发展工程质量保险，积极开展质量保险顶层设计研究， 以城市为单位启动新一轮质量保险试点，加快推动全国工程质量保险信息系统建设。制定建筑施工安全生产责任 保险实施办法，建立健全投保理赔事故预防机制。推动建立建筑工程质量评价制度，形成可量化的评价指标和评价机制，鼓励通过政府购买服务，委托具备条件的第三方机构独立开展质量评价。推进实施住宅工程质量信息公示制度，充分发挥社会监督约束作用。推动建设工程消防技术服务市场化，规范技术服务行为。

5.强化勘察设计质量管理。

健全完善勘察设计质量管理制度，修订勘察质量管理办 法，制定设计质量管理办法。强化施工图审查作用，全面推 广数字化审查，探索推进 BIM 审查和人工智能审查。推动建 立勘察设计质量监管信息系统，加强勘察设计质量全过程信 息化监管，加大对违反法律法规和工程建设强制性标准问题 的查处力度，建立施工图审查关键信息公开制度。加强和改 进消防设计审查管理，探索推进技术审查与行政审批分离， 推动消防设计技术审查第三方服务发展。推动将消防设计技术审查和施工图审查同步开展，提高审查质量和效率。

6.优化工程竣工验收制度。

完善住宅分户验收制度，鼓励购房者参与分户验收，按 户留存影像资料，作为住宅交付档案。细化《住宅质量保证 书》《住宅使用说明书》，制定发布示范文本，明确“两书” 规范格式和基本内容。试行建设单位按套出具住宅质量合格证明文件。规范消防验收管理，推动消防验收纳入竣工联合验收，统一出具验收意见。

7.推进工程质量安全管理标准化和信息化。

全面推行工程质量安全手册制度，加快健全手册体系， 完善建筑施工企业和工程项目安全生产标准化考评制度。研 究制定装配式建筑质量安全管理制度，运用信息化手段，实 现部品部件生产质量可追溯管理，加强竖向节点连接等施工 关键环节质量安全管控。深化施工安全领域 “证照分离” 改 革，推进涉企、涉人证照电子化，实现建筑施工特种作业操 作资格证书信息联网和一站式查询。制定建筑工程材料、工 艺、设备鼓励应用和限制淘汰名录，推广安全先进适用的建造技术，限制淘汰落后工艺。

| 专栏 8 智慧城市轨道交通工程建设 |
| --- |
| 2025 年，城市轨道交通工程质量安全责任体系、风险防控体系更加健全，标准化、信息化、智能化水平明显提升。  1.推进智慧工地建设。强化建设单位质量安全首要责任，完善多 阶段验收管理对策措施。推进城市轨道交通工程质量安全管理信息平台建设运用，提高风险隐患智能管控能力。  2.提升第三方监测智慧化水平。完善第三方监测数据采集技术手段，推进施工现场风险动态监测、 自动分析和智能预警。  3.完善风险防控技术措施。对全国城市轨道交通建设工程相关的 基坑、隧道坍塌事故典型案例和盾构施工风险防控等进行调查研究，完善关键技术措施，强化重大风险管控。 |

（六）稳步提升工程抗震防灾能力。

1.健全工程抗震防灾制度和标准体系。

落实《建设工程抗震管理条例》有关规定，全面梳理现行制度体系，加快制修订配套规章制度。不断完善工程抗震防灾技术标准体系，加大标准前期研究力度，加快制定工程 抗震鉴定和加固标准，制修订工程减震隔震等抗震新技术应用标准，为提升工程抗震防灾水平提供支撑。

2.严格建设工程抗震设防监管。

加强建设工程抗震标准实施监督和抗震设防质量监管， 建立重点地区重大建设工程抗震设防专篇编制制度，完善超限高层建筑工程抗震设防审批、市政工程抗震设防专项论证 制度。全面落实位于高烈度设防地区、地震重点监视防御区 建筑抗震设防要求，保障新建学校、幼儿园、 医院、养老机 构、儿童福利机构、应急指挥中心、应急避难场所、广播电 视等建筑满足设防地震下正常使用要求。落实工程抗震责任企业及从业人员信用记录制度，加大信用信息公开力度。

3.推动工程抗震防灾产业和技术发展。

推动工程抗震防灾产业发展，支持新型经济快速抗震加 固、新型减隔震、结构主被动一体化等技术成果转化。建立 隔震减震装置质量信息全过程追溯管理机制，探索隔震减震 装置质量信息公示制度，发挥社会监督约束作用，保障产业 健康发展。加强抗震防灾基础理论和应用研究，逐步实现工程抗震计算软件和大型设备等关键核心技术基本自主可控。

4.提升抗震防灾管理水平和工程抗震能力。

全面完成第一次全国自然灾害综合风险普查房屋建筑和市政设施调查，建立全国统一的房屋建筑和市政基础设施工程抗震防灾基础数据库，利用信息化手段提高工程抗震防 灾管理的现代化水平，为城市信息模型（CIM）平台建设和 工程建设数字化监管提供基础数据。加强房屋建筑和市政基 础设施抗震性能鉴定工作，推进实施地震易发区房屋设施加固工程，提升既有建筑抗震能力。

（七）加快建筑业 “走出去”步伐。

1.推进工程建设标准国际化。

加强与有关国际标准化组织的交流合作，参与国际标准 化战略、政策和规则制定。主动参与国际标准编制和管理工 作，积极主导国际标准制定。加快我国工程建设标准外文版 编译，鼓励重要标准制修订同步翻译。加强与 “ 一带一路” 沿线国家及地区的多边双边工程建设标准交流与合作，推动 我国标准转化为国际或区域标准。加强我国标准在援外工程、 “ 一带一路”建设工程中的推广应用。

2.提高企业对外承包能力。

鼓励我国建筑企业、工程设计等咨询服务企业参与共建 “一带一路”，积极开展国际工程承包和劳务合作。支持企 业开展工程总承包和全过程工程咨询业务，推动对外承包业 务向项目融资、设计咨询、运营维护管理等高附加值领域拓 展，逐步提高我国企业在国际市场上的话语权和竞争力。加强对外承包工程监督管理，规范企业海外经营行为。

3.加强国际交流与合作。

加快推动与 “ 一带一路”沿线国家及地区签订双边工程 建设合作备忘录，加强政府主管部门沟通协调和信息共享， 共同推动建筑企业 “走出去”。推进注册建筑师等工程建设 领域执业资格国际互认，拓展青年人才交流合作渠道，加快培养熟悉国际规则的复合型人才。

四、保障措施

（ 一）强化规划实施。

各地要加大统筹、协调和支持力度，建立协同推进机制， 明确任务分工，加强动态跟踪，确保规划各项目标任务落到 实处。鼓励行业协会积极向政府部门反馈规划实施情况和政策建议，发挥好行业自律作用，提升服务行业和企业的能力。

（ 二）开展评估考核。

加强对规划实施情况的统计监测和绩效评估，根据任务 进展情况、阶段目标完成情况、技术发展新动向等对规划进 行动态调整。完善监督考核机制，对规划实施效果显著的地区予以通报表扬，督促规划组织实施不到位的地区加大工作力度。

（三）加强宣传引导。

各地要及时总结可复制可推广的实践经验，广泛宣传规划实施的新进展和新成效，调动社会各界支持建筑业高质量发展的积极性，营造良好的发展环境。

住房和城乡建设部
2022年1月19日

**P28**

**Notice on Expanding the Scope of Government Procurement Policies to Support Green Building Materials and Improve Building Quality**

**关于扩大政府采购支持绿色建材促进建筑品质提升政策实施范围的通知**

财库〔2022〕35号

各省、自治区、直辖市、计划单列市财政厅（局）、住房和城乡建设厅（委、管委、局）、工业和信息化主管部门，新疆生产建设兵团财政局、住房和城乡建设局、工业和信息化局：

为落实《中共中央 国务院关于完整准确全面贯彻新发展理念做好碳达峰碳中和工作的意见》，加大绿色低碳产品采购力度，全面推广绿色建筑和绿色建材，在南京、杭州、绍兴、湖州、青岛、佛山等6个城市试点的基础上，财政部、住房城乡建设部、工业和信息化部决定进一步扩大政府采购支持绿色建材促进建筑品质提升政策实施范围。现将有关事项通知如下：

一、实施范围

自2022年11月起，在北京市朝阳区等48个市（市辖区）实施政府采购支持绿色建材促进建筑品质提升政策（含此前6个试点城市，具体城市名单见附件1）。纳入政策实施范围的项目包括医院、学校、办公楼、综合体、展览馆、会展中心、体育馆、保障房等政府采购工程项目，含适用招标投标法的政府采购工程项目。各有关城市可选择部分项目先行实施，在总结经验的基础上逐步扩大范围，到2025年实现政府采购工程项目政策实施的全覆盖。鼓励将其他政府投资项目纳入实施范围。

二、主要任务

各有关城市要深入贯彻习近平生态文明思想，运用政府采购政策积极推广应用绿色建筑和绿色建材，大力发展装配式、智能化等新型建筑工业化建造方式，全面建设二星级以上绿色建筑，形成支持建筑领域绿色低碳转型的长效机制，引领建材和建筑产业高质量发展，着力打造宜居、绿色、低碳城市。

（一）落实政府采购政策要求。各有关城市要严格执行财政部、住房城乡建设部、工业和信息化部制定的《绿色建筑和绿色建材政府采购需求标准》（以下简称《需求标准》，见附件2）。项目立项阶段，要将《需求标准》有关要求嵌入项目建议书和可行性研究报告中；招标采购阶段，要将《需求标准》有关要求作为工程招标文件或采购文件以及合同文本的实质性要求，要求承包单位按合同约定进行设计、施工，并采购或使用符合要求的绿色建材；施工阶段，要强化施工现场监管，确保施工单位落实绿色建筑要求，使用符合《需求标准》的绿色建材；履约验收阶段，要根据《需求标准》制定相应的履约验收标准，并与现行验收程序有效融合。鼓励通过验收的项目申报绿色建筑标识，充分发挥政府采购工程项目的示范作用。

（二）加强绿色建材采购管理。纳入政策实施范围的政府采购工程涉及使用《需求标准》中的绿色建材的，应当全部采购和使用符合相关标准的建材。各有关城市要探索实施对通用类绿色建材的批量集中采购，由政府集中采购机构或部门集中采购机构定期归集采购人的绿色建材采购计划，开展集中带量采购。要积极推进绿色建材电子化采购交易，所有符合条件的绿色建材产品均可进入电子平台交易，提高绿色建材采购效率和透明度。绿色建材供应商在供货时应当出具所提供建材产品符合需求标准的证明性文件，包括国家统一推行的绿色建材产品认证证书，或符合需求标准的有效检测报告等。

（三）完善绿色建筑和绿色建材政府采购需求标准。各有关城市可结合本地区特点和实际需求，提出优化完善《需求标准》有关内容的建议，包括调整《需求标准》中已包含的建材产品指标要求，增加未包含的建材产品需求标准，或者细化不同建筑类型如学校、医院等的需求标准等，报财政部、住房城乡建设部、工业和信息化部。财政部、住房城乡建设部、工业和信息化部将根据有关城市建议和政策执行情况，动态调整《需求标准》。

（四）优先开展工程价款结算。纳入政策实施范围的工程，要提高工程价款结算比例，工程进度款支付比例不低于已完工程价款的80%。推行施工过程结算，发承包双方通过合同约定，将施工过程按时间或进度节点划分施工周期，对周期内已完成且无争议的工程进行价款计算、确认和支付。经双方确认的过程结算文件作为竣工结算文件的组成部分，竣工后原则上不再重复审核。

三、工作要求

（一）明确部门职责。有关城市财政、住房和城乡建设、工业和信息化部门要各司其职，加强协调配合，形成政策合力。财政部门要组织采购人落实《需求标准》，指导集中采购机构开展绿色建材批量集中采购工作，加强对采购活动的监督管理。住房和城乡建设部门要加强对纳入政策实施范围的工程项目的监管，培育绿色建材应用示范工程和高品质绿色建筑项目。工业和信息化部门要结合区域特点，因地制宜发展绿色建材产业，培育绿色建材骨干企业和重点产品。

（二）精心组织实施。有关城市所在省级财政、住房和城乡建设、工业和信息化部门收到本通知后要及时转发至纳入政策实施范围城市的财政、住房和城乡建设、工业和信息化部门，切实加强对有关城市工作开展的指导。有关城市要根据政策要求，研究制定本地区实施方案，明确各有关部门的责任分工，完善组织协调机制，对实践中出现的问题要及时研究和妥善处理，确保扩大实施范围工作顺利推进，取得扎实成效。要积极总结工作经验，提炼可复制、可推广的先进经验和典型做法。

（三）加强宣传培训。各有关地方和部门要依据各自职责加强政策解读和宣传，及时回应社会关切，营造良好的工作氛围。要加强对建设单位、设计单位、建材企业、施工单位的政策解读和培训，调动相关各方的积极性。

财政部 住房城乡建设部 工业和信息化部
2022年10月12日

**P29**

**Notice on Announcing Intelligent Construction Pilot Cities**

**住房和城乡建设部关于公布智能建造试点城市的通知**

建市函〔2022〕82号

各省、自治区住房和城乡建设厅，直辖市住房和城乡建设（管）委，新疆生产建设兵团住房和城乡建设局：

为贯彻落实党中央、国务院决策部署，大力发展智能建造，以科技创新推动建筑业转型发展，经城市自愿申报、省级住房和城乡建设主管部门审核推荐和专家评审，我部决定将北京市等24个城市列为智能建造试点城市（名单见附件），试点自公布之日开始，为期3年。

试点城市要严格落实试点实施方案，建立健全统筹协调机制，加大政策支持力度，有序推进各项试点任务，确保试点工作取得实效。要及时总结工作经验，形成可感知、可量化、可评价的试点成果，每季度末向我部报送试点工作进展情况，每年年底前报送试点年度报告。有关省级住房和城乡建设主管部门要加大对试点城市的指导支持力度，宣传推广可复制经验做法，推动解决问题困难。我部将定期组织对各试点城市的工作实施进度、科技创新成果、经济社会效益等开展评估，对真抓实干、成效显著的试点城市予以通报表扬，对工作进度滞后的试点城市加强调度督导。

请试点城市于2022年11月底前将完善后的试点实施方案以及1名工作联系人报我部建筑市场监管司。试点工作中的有关情况和问题，请及时沟通联系。

住房和城乡建设部
2022年10月25日

**P30**

**"14th Five-Year" Special Plan for Urbanization and Urban Development Technological Innovation**

**"十四五"城镇化与城市发展科技创新专项规划**

国科发社〔2022〕320号

为明确“十四五”时期城镇化与城市发展领域科技创新的总体思路、发展目标和重点任务，根据《中华人民共和国国民经济和社会发展第十四个五年规划和2035年远景目标纲要》，制定本规划。

一、形势与需求

（一）我国城镇化与城市发展科技创新现状。

党的十八大以来，我国在城镇区域规划、绿色建筑、城市基础设施和生命线工程、城市功能提升、生态居住环境改善、城市信息化管理、城市文化遗产保护与价值挖掘等方面的科技创新取得了长足进展。超高层建筑、大跨度空间结构、跨江跨海超长桥隧等特种结构工程建造技术居于世界领先水平，建筑节能技术达到世界先进水平，新型建筑结构突破技术瓶颈，工程设计实现自主研发。但是与世界领先水平相比，我国城镇化领域大部分技术仍处在跟跑或并跑阶段，城镇基础设施建设相关材料、装备及工程专业软件等领域的应用基础研究仍然不足。同时，城市信息化水平尚不能满足现代化治理的需求，实现城乡建设领域碳减排目标还需要更多绿色低碳技术支撑。

（二）国际城镇化与城市发展科技创新发展趋势。

近10年来，以城市群和都市圈为代表的巨型城市区域成为国际研究热点，在巨型城市区域落实《巴黎气候协定》《生物多样性公约》等逐渐成为焦点，包括基于自然的规划措施、资源优化配置和动态调整、完善公共交通和城市基础设施等。一些城镇化率较高的国家在城镇化与城市发展领域科技部署时，更加关注绿色建筑、低碳城区、适老化社会建设和既有城区建筑改造升级，更加注重信息技术在国土空间优化和城市（群）建设规划、城市基础设施运维、城市功能和空间效率提升等方面的研究和应用。

（三）我国城镇化与城市发展科技创新战略需求。

“十四五”期间，我国城市发展将从经济主导更多转向生产生活生态多元导向，城市建设方式将由增量扩张转向存量挖潜，城市生产生活方式将加快绿色低碳转型。城镇化与城市发展科技创新要紧密结合我国城镇化进程需求，以满足人民日益增长的美好生活需要为根本目的，提高城镇规划建设科学化水平与城市运行智慧化水平，引领住房城乡建设低碳转型，促进城镇可持续发展，全面支撑建设宜居、创新、智慧、绿色、人文、韧性城市。

二、指导思想和基本原则

（一）指导思想。

坚持以习近平新时代中国特色社会主义思想为指导，深入贯彻党的二十大精神，完整、准确、全面贯彻新发展理念，坚持创新驱动发展，推动高质量发展，面向世界科技前沿、面向经济主战场、面向国家重大需求、面向人民生命健康，以体系设计为总领、以目标导向为主线、以技术突破为重点、以场景应用为驱动，进一步整合科技资源、加强统筹协调，着力提升城镇化与城市发展领域的科技支撑能力，破解城镇化发展难题，构建中国特色新型城镇化范式，开创城镇化与城市发展领域科技创新工作新局面。

（二）基本原则。

坚持以人民为中心。以保障民生、增进人民福祉为出发点，解决城镇化进程中人民群众最关心、最直接、最迫切的问题，不断满足人民群众对城市和建筑舒适性、健康性、功能性需求，提升建筑宜居水平，丰富城市文化内涵。

坚持绿色低碳可持续发展。面向碳达峰碳中和目标，狠抓城镇化领域绿色低碳技术攻关，全方位全过程推行绿色规划、绿色建造、绿色运维、绿色消纳，有效降低能源消耗与温室气体排放。

坚持系统思维与创新引领。围绕城市建设全生命周期，统筹规划、设计、建设和运维各环节创新主体，推动政产学研用深度融合，加强关键核心技术与装备研发攻关，加快新技术在城镇化领域的典型场景应用，以科技创新驱动城镇可持续发展。

三、发展目标

到2025年，城镇化与城市发展领域科技创新体系更趋完善，基础理论水平与创新能力显著提高，为新型城镇化提供更高质量的技术解决方案，有力支撑城镇低碳可持续发展，推动城市建设与文化旅游等相关产业发展壮大，科技成果更多更好地惠及民生。

应用基础研究水平显著提升。构建国际领先、中国特色的国土空间、城市（群）建设规划理论和方法。在建筑结构体系与工程建造材料应用基础研究方向取得新突破，形成以健康、低碳和高品质为目标的数字设计、建造和运维的新方法和新工具。

关键核心技术装备研发能力显著增强。在城市更新、建筑低碳节能、韧性城市和全龄友好城市建设、智能建造软硬件平台、文旅资源保护利用等方面突破一批关键技术装备。实现建筑与基础设施功能提升、智能建造和智慧运维、公共文旅服务等领域关键核心技术的国际并跑与局部领跑。

领域创新能力体系建设取得新进展。培养一批城镇化领域高端人才和创新团队，推动建设一批国家级科技创新基地和产业技术创新战略联盟，培育一批城镇化领域科技创新骨干企业，进一步优化政产学研用深度融合的创新体系。

科技创新示范引领作用加快凸显。在国家可持续发展议程创新示范区、雄安新区以及京津冀、长三角、粤港澳大湾区、成渝地区等重点区域，完成一批城市生态修复与功能完善、城乡历史文化遗产保护、城镇老旧小区改造创新示范工程，建设一批高品质绿色健康建筑和低碳宜居示范城市。

四、重点任务

（一）加强城市发展规律与城镇空间布局研究。

深入推进以人民为中心的城镇化发展战略，加强城市发展规律与城市体系布局研究，提升规划调控能力，支撑服务国家城市与城市群战略性布局。推进以县城为重要载体的新型城镇化，推动城乡建设高质量发展，构建具有中国特色的城镇空间优化开发、城市（群）及都市圈建设规划设计、城市体检评估等新型城镇化创新理论方法、关键技术体系与应用示范平台。

| 专栏1 城市发展规律与城镇空间布局 |
| --- |
| 1. 城市群和区域可持续发展指标与智能监测技术。研究基于生态本底网络结构的城市群和区域绿色发展的方法论；研究城市群和区域可持续发展的指标与评价体系；研究基于碳中和目标的低碳城市综合评价方法、碳排放核算技术和全生命周期碳代谢模拟技术；研发城市群和区域建成区的实时监测与感知技术；研发基于生态优先的城市群人-地-产耦合评估技术；研究城镇复杂场景的多模态融合感知与场景智能认知技术；开展京津冀、长三角、粤港澳大湾区的试点工作。  2. 城市体检评估技术。研究城市体检评估方法、标准与指标体系；研究面向常态化监测的城市体检体制与机制；研究城市体检多源数据的自动化采集、综合分析处理及标准化诊断技术；依托城市信息模型基础平台，搭建国家-省-市联动的仿真、模拟与智能决策的城市体检信息平台与数据库。  3. 数字化规划设计。研究基于多维空间传输的城市空间数字规划设计方法；研发具有自主知识产权的图形引擎技术；研发城市设计方案智能生成与仿真技术；研究人的环境行为演变及多尺度空间演变模式识别技术，建立中国超大城市中人的环境行为演变模式识别成果库；研究基于实景三维的多尺度时空地理信息数据生产、建模、管理及服务技术，搭建国家-省-市多层级分布式时空地理信息数据库与平台。 |

（二）加强城市更新与品质提升系统技术研究。

面向城市大规模增量建设转为存量提质改造和增量结构调整并重发展的阶段，针对我国城市功能宜居、绿色低碳、智慧人文的发展需求，以城市全生命周期管理和市政设施运维安全高效、智慧智能、集约节约为目标进行关键核心技术研究，全面提升城市品质，提高以人为核心的城市建设水平，支撑完整社区、城镇老旧街区（小区）改造、历史文化街区更新保护、既有建筑和工业园区再利用、地下空间高效利用等新时期城市更新工作，开展规模化工程示范。

| 专栏2 城市更新与品质提升 |
| --- |
| 1. 既有建筑和市政基础设施诊治更新。研究既有工业厂区、历史文化街区、城区人文保护、改造与功能提升技术；研究既有建筑、社区一体化绿色改造、健康改造、适老改造、消防安全改造、垃圾分类投放设施等宜居改造与性能提升技术与装备；研发建筑与基础设施的智能检测、监测技术与装备；研发建筑与基础设施全生命周期性态演变评估与控制技术；研发建筑与基础设施高效修复、加固技术与装备。  2. 地下空间开发与地上空间高效利用。研究地下空间高效开发利用规划、建设和运维基础理论；研究地下空间资源开发适宜性评价与三维规划管控原理；研发地上地下环境约束下的地下空间容积率控制原理、调查规划方法与高效利用技术；研究地下空间防灾规划技术；研发地下空间开发建造技术与设备，包括地下大空间开发装备、深层地下空间开发技术与装备、受限空间增容开发技术与装备、地下空间开发可持续发展技术、智能化地下空间开发及工程建造技术。  3. 全龄友好城市、活力街区和完整社区。研究城区各类建设场景的智慧建造技术，城市无障碍环境建设技术体系，城市噪音控制与城市热岛效应优化技术，智慧停车管理与慢行交通系统建造技术，基于公共交通导向（TOD）的多功能综合体建造技术，全龄友好型城市公共设施、公共环境、居家环境、信息环境评价与建造技术体系，社区居家养老服务技术体系、普惠托育与适婴适童主动健康服务设施建设技术体系，多场景、多业态全龄社区服务设施建设技术。 |

（三）加强智能建造和智慧运维核心技术装备研发。

面向存量巨大的建筑与基础设施高效运维及街道社区精细化运维等城镇社会可持续发展的公共服务需求，以数字化、智能化技术为基础，开展智能建造与智慧运维基础共性技术和关键核心技术研发与转化应用，促进建筑业与信息产业等业态融合，显著提高建筑工业化、数字化、智能化水平，推进市政公用设施的物联网应用和智能化改造，提升建筑与市政公用设施系统协同管控能力、保障设施供给安全，提升城市运维效率。

| 专栏3智能建造与智慧运维 |
| --- |
| 1. 工业化建造与智能建造软件装备。研究非线性几何特征建模与BIM图形引擎，建立具有自主知识产权的BIM三维图形平台并发展相应软件生态；研发部品部件智能生产线；开发面向典型工程、极端工程建造场景的嵌入式智能融合感知终端；研究大型工地施工现场全要素感知自适应组网技术与多模态异构数据的智能融合技术；研究基于工程供应链、产业链和价值链的建筑产业互联网关键技术；研发智能化工程机械、建筑机器人装备以及人机协同作业系统；研究贯通数字设计、智能生产、智能施工等全产业链的技术标准体系。  2. 高性能土木工程材料与结构体系。研究可持续及环境友好型先进土木工程材料，包括先进水泥基材料、金属材料、复合材料、智能材料、可再生与低碳排放材料等；构建基于材料结构一体化的适应复杂需求和严苛环境的新型结构体系；研发基于工业化建造的城市桥梁新体系及其安全运营和韧性提升关键技术；研发适应工业化与智能建造的新型建筑结构体系与关键技术。  3. 智慧运维。研究公共服务数据治理与数字孪生技术；研究基于三维空间单元的城市信息模型（CIM）理论和平台构建关键技术与应用；研究城镇智能体理论、数据与运行安全等技术标准体系；研究建筑、大型交通枢纽与市政公用设施智慧运维关键技术装备，研究城市数据大脑及数字孪生城市建设理论与技术，构建全场景智能监测预警和智慧综合运维服务平台；研发城市道路系统协同运行平台；研究融合智慧社区与智慧家庭构建方法和技术体系，开展智慧城镇综合示范。 |

（四）加强绿色健康韧性建筑与基础设施研究。

为推进绿色建筑与基础设施建设，提升人居环境，提高居民满意度和获得感，通过整合信息化、新能源和新材料技术，在基础理论和设计方法、工程技术标准、新型绿色建材、围护结构系统和部品、高效机电设备、高性能绿色建筑、健康社区与健康建筑、韧性城市等方面实现全链条技术产品创新并进行集成示范。

| 专栏4 绿色建筑与基础设施 |
| --- |
| 1. 高性能绿色建筑。研究基于人工智能与人因工程学的绿色建筑设计新理论新方法，研究多主体、全专业、高效能的绿色建筑设计建造全过程协同平台，编制高质量发展背景下新一代绿色建筑工程技术标准体系；研发性能可调建材与多功能复合、结构功能一体化的新型智能围护结构产品，开发高效能机电设备与系统；研发低增量成本、高性能绿色建筑和超低能耗建筑、近零/零能耗绿色建筑关键技术体系。  2. 健康社区与健康建筑。研究空气、声音等环境要素对人健康的定量影响（包括增强性影响）与相关机理，建立包括多尺度室内外环境参数等在内的数据收集平台；开发社区-建筑室内外环境健康保障和优化提升关键技术，非视觉健康照明和健康睡眠保障技术，健康建筑与健康社区规划设计和性能保障技术体系；研究未来社区规划设计和功能体系，开展健康社区和健康建筑技术集成和示范。  3. 韧性城市。研究面向不同类型灾害风险的韧性城市理论及设计、分类评价技术，城市综合防灾规划理论，韧性城市区域致灾动态模型与多灾害韧性动态评估技术，城市生态空间韧性功能提升技术，建筑抗震、抗风、防火抗爆韧性系统提升技术及韧性结构新体系，城市应急广播技术体系、城市综合风险评估技术体系，城市应急避难场所、防洪排涝等基础设施建设、评价和运维技术，城市群、都市圈空间韧性功能协同提升技术。 |

（五）加强城镇发展低碳转型系统研究。

以建筑领域积极落实碳达峰碳中和目标为导向，面向城镇能源系统发展目标，从单纯追求能源消费侧的节能减量转变为以低碳发展为导向的能源消费侧革命，积极开展城镇低碳发展表征评价方法与监测系统、城市低碳能源系统、光储直柔新型配电系统、市政基础设施低碳减排与提质增效、城市生态修复与功能完善、零碳建筑、绿色消纳等关键技术与装备研究，推进零碳零排放城市示范。

| 专栏5城镇低碳支撑系统 |
| --- |
| 1. 城镇新型低碳清洁能源系统。研发热水联供、热电协同、烟气余热利用与减排一体化、大温差跨季节水热联储等系列技术；研究光储直柔新型供配电系统基础理论、安全保护方法及相关标准，研发新型光伏一体化技术体系，直流供配电关键设备与技术；开展北方城镇地区低品位余热清洁供暖工程示范，以及源网荷储用协同的区域能源系统试点示范。  2. 市政基础设施低碳减排与提质增效。研究供排水设施低碳排放与提质增效协同优化技术，污水收集处理过程温室气体控制与碳捕集技术，生态型水体全要素全生命周期低碳建设与运维技术，降雨径流污染低碳净化技术，可再生能源为核心的多能互补燃气供应、能源梯级利用和运行优化技术，生活垃圾处理设施低碳排放技术与装备，城市园林绿化碳汇增效技术。  3. 生态修复与功能完善。研究城市生态修复与生境重建相关的城市生态基础设施建设关键技术，基于遥感技术的城市河湖岸线生态系统恢复及调控技术，城市水循环体系智慧管控技术，城镇近自然生态环境营建与运维技术，公园城市背景下都市空间绿化关键技术，研究城市光热环境耦合调控治理技术、竖向城市构建与宜居环境营造技术。  4. 绿色消纳。研究建筑与基础设施低环境影响拆解技术，建（构）筑物三维形态及拆解受力特征的快速测绘分析技术，自动化、智能化建（构）筑物拆解专用装备，适用于城市复杂环境的拆解专用器具与防护作业工具，拆解过程有害效应的生成传播机制与精细控制技术，各类工业建（构）筑物高效拆解技术，拆除垃圾与城市垃圾低碳处理可再生综合利用技术。 |

（六）加强文物科技创新与城市历史文化遗产保护研究。

面向包括历史文化名城名镇名村街区、文物史迹、古建筑、古遗址等在内的文化遗产保护和传承利用的重大需求，加强文物保护与认知基础研究和共性关键技术攻关，创新文物知识挖掘和展示传播技术，构建中国特色、中国风格、中国气派的考古学，建立完善文化遗产全周期保护修复和风险预控理论与技术体系，保护和共享城乡历史文化资源，全面支撑基于历史文化遗产的学习、教育和国际交流等。

| 专栏6 文物保护和传承利用 |
| --- |
| 1. 文物保护与认知基础研究。研究夏商时期文明传承、统一多民族国家形成进程，中华文明起源与早起发展的整体脉络和历史规律；研究文物劣化机理与环境作用机制，研究文物典型劣化过程（病害）的等效模拟或实验验证方法；研究文物建筑火灾蔓延机理；研究文物保护材料作用机理、失效机制、环境影响和服役周期预测方法；研究古代工艺逆向重建与文物产地溯源理论与方法，建立主要产地的示踪指标基础数据库。  2. 文物保护与认知共性关键技术。研发文物表层病害无损检测和智能诊断关键技术与装备；研发文物本体稳定化处理关键技术和装备；研发文物保护新型功能性材料；研究基于历史数据和多维特征的文物建筑动态风险智能评估方法、监测预警模型及防控技术装备，研发针对文物建筑火灾早期探测、快速救援处置技术和专用装备，研究古建筑区域防雷、生物风险监测和出土文物应急保护环境控制成套技术和专用装备；研发考古探测、发掘与研究关键技术和装备。  3. 文物知识挖掘与展示传播技术。研究博物馆文物知识智能化深度展示方法与技术，研发馆藏文物数字物纹提取关键技术与智能监管系统；研发面向特殊场景的虚实融合的文物知识展示与传播技术；研发数字空间文物知识展示与传播技术，研发文物超高清数字化与超高真实感绘制、珍贵文物动态历史信息呈现与多模态交互、在线数字孪生博物馆关键技术。 |

（七）加强文化旅游融合与公共文化服务科技创新。

针对我国文化服务领域智能技术应用、信息化技术融合不足，以及在提供安全、便利的旅游服务和精准智能旅游监管等方面的不足，研究文化和旅游科技的基础理论、关键核心技术以及系统集成技术，提升文化和旅游融合发展的科技支撑能力，实现文化和旅游资源保护与管理服务共性关键技术突破，推动中国文化和旅游高端装备形成国际竞争力，以智能服务平台促进文旅行业监管模式变革。

| 专栏7文化与旅游融合 |
| --- |
| 1. 文旅资源保护利用。研发文化资源保护与复原复现关键技术，优化文化数据提取、存储、利用技术；研究传统文化素材数字化挖掘与素材化基础理论方法，研发文化资源数字化与内容挖掘集成技术；研究语言及视听认知表达、跨媒体内容识别与分析、情感分析等智能创作基础理论与方法，研发文化资源内容创作技术与装备；研究新型全感知、自然交互、虚拟现实、全息影像等视听技术，全媒体内容智能认知和生产处理技术；研发旅游资源保护开发技术与装备，开发旅游资源数据服务平台。  2. 公共文旅服务。研发公共文化服务共性关键技术与装备；研发公共文化服务效能大数据分析集成技术；研发物理形态和数字形态文化资源的备灾存储关键技术与系统装备；研究景区和博物馆等智慧文化场馆构建技术，开发国家文化公园、基础文化设施等智能化监测服务等技术和装备，研发具备智慧媒体功能的公共文化信息视听服务装备；研发旅游智慧发展与旅游公共服务集成技术与系统；研发文旅演艺和文旅展演空间共性关键技术与专用装备；研发文化场馆和文化资源旅游应用专用技术与装备。  3. 文化和旅游行业治理能力提升。研究文旅“智慧大脑”，研发文化行业治理与安全保障关键技术与装备，研究文化场所和设施安全保障基础理论与关键技术，制定文化领域安全技术标准，开发文化行业大数据统计与分析集成技术和系统；研究旅游统计调查和征信服务基础理论；研究国家旅游大数据体系理论与关键技术，研发旅游行业治理与安全保障技术与装备，开发旅游区域安全检验检测、大密度人流安全风险监控及处置等技术装备。 |

五、保障措施

（一）完善体制机制，激励自主创新。

完善科研经费管理机制和科研评价体制，坚持分类评价与政策激励相结合，使科研项目和资金配置更好满足城镇化与城市发展领域重大需求。充分发挥科研人员的积极性和创造性，切实提升自主创新能力。注重科研成果的创新性和系统性，坚持科研工作源于工程、服务工程、引领工程，坚持绩效评价导向，完善同行评议和考评机制，简化项目申请程序，优化完善过程管理。

（二）加强政策扶持，创新投入机制，推动产业发展。

充分运用现有政策和资金渠道，着力支持关键技术研发、应用示范、成果产业化、创新能力建设等。鼓励各级地方政府加大财政扶持力度，强化对新型城镇化相关产业投入的引导和带动作用，建立以政府扶持为引导、企业投入为主体、多元社会资金参与的创新投入机制，推动建筑业等相关产业高质量发展。

（三）统筹基地平台建设，促进产学研用深度融合。

面向城镇绿色低碳可持续发展需求，强化国家战略科技力量，统筹推进全国重点实验室、国家技术创新中心、国家野外科学观测研究站的建设，对符合“十四五”国家重点科研平台建设领域和建设方向的行业重点研究平台，加大支持力度，力争培育进入国家重点科研平台序列，不断提升行业重点科研平台的发展水平。

（四）加强人才培养支持力度。

通过各类人才计划，加快培养领域科技领军人才和创新团队。完善人才激励机制和评价体系，以国家科技计划项目为纽带，重点培养中青年科研骨干。重点支持青年科技人才持续发展，建设行业专家智库。支持青年科技人才承担重大科研项目，开展独立性和原创性研究。

（五）推动科技成果示范应用与试点推广。

结合国家可持续发展议程创新示范区建设，在城镇化基础条件好和可持续发展需求迫切的重点领域，围绕智能建造装备、低碳技术集成应用等方面，开展新模式、新技术、新产品试点示范，形成有效的经验和模式，选择标杆企业和产品进行推广。

（六）积极开展国际交流与合作。

在标准制定、知识产权等方面广泛开展国际交流与合作，不断拓展合作领域。支持国内外科研机构、企业及行业组织间开展技术交流与合作，促进人才、资本、技术等科技创新要素的自由流动。鼓励跨国公司、国外机构等在华设立相关领域研发机构。实行更高水平对外开放，推动“一带一路”高质量发展，提升科技支撑能力和国际竞争力。

科技部 住房城乡建设部
2022年11月18日
