# Supplementary material for: Research on quantitative evaluation of China’s Intelligent Construction Policy (CICP) based on the integration of PMC index model and multi-dimensional analytical framework
Source: PLoS One. 2025 Jul 15;20(7):e0326505. doi: 10.1371/journal.pone.0326505 (PMC12262895; doi:10.1371/journal.pone.0326505)
Supplement: Attachment — Submitted filename: Response to Reviewers.docx [file pone.0326505.s002.docx]

**Response to Reviewer Comments**

**Manuscript ID: PONE-D-24-31927**
**Title:** **Research on quantitative evaluation of China's Intelligent Construction Policy (CICP) based on the Integration of PMC index model and multi-dimensional analytical framework**

Dear Reviewer,

We sincerely appreciate your rigorous evaluation and insightful feedback on our manuscript. Your expertise has been invaluable in refining this work, and we are deeply grateful for the time and intellectual rigor you devoted to the review process. In response to your critiques, we formed a dedicated revision team to systematically address each point raised:

**Comment 1:** Lines 42-45, authors used a few examples to introduce the technologies. However, not all of them are trivial or clear to the readers why they are considered "core technologies" and What role they play in construction.

**⦁ Response:** We appreciate your valuable feedback. To address this concern, we have revised the introduction of the core technologies in the manuscript to provide clearer explanations regarding their significance and roles in the construction industry. Specifically, we have elaborated on the relevance of each technology in the context of modern construction practices, highlighting how each one contributes to improving efficiency, sustainability, or safety. Additionally, we have clarified why these particular technologies are considered "core" in the field. These clarifications can be found on **lines 42-51** of the revised manuscript.

**Comment 2:** Another key contribution of the paper that the authors claimed is to fill the gap between existing literatures. Lines 62-64, authors mentioned a lack of quantitative analysis in policy evaluation but doesn’t explain the significance of filling this gap. Please Expand on why this gap matters. For example, explain how a quantitative approach would improve the accuracy of policy assessments or how it could impact future policy decisions.

**⦁ Response:** Thank your for pointing out the need for further elaboration on the significance of addressing this gap in the literature. We agree that a more detailed explanation would strengthen the argument for the importance of this gap. In response, we have expanded the discussion in the revised manuscript to emphasize how the lack of quantitative analysis in policy evaluation limits the ability to assess the true impact of policies. Specifically, we have explained that a quantitative approach can provide more robust and objective insights into policy effectiveness, helping to identify patterns, assess outcomes with greater precision, and inform better decision-making. Furthermore, we have discussed how such an data-driven approach can enhance the credibility of policy recommendations by offering empirical evidence that supports or challenges policy assumptions. We believe this is particularly important in the development process of CICP, where accurate assessments are crucial for long-term success. These clarifications can be found on **lines 66-75** of the revised manuscript.

**Comment 3:** The literature review session, lines 98-124, can be improved. The literature review feels like a collection of studies without a clear thematic grouping. I would suggest that group the studies into clear thematic sub-sections (e.g., technological innovation in construction, policy evolution in construction). This will help readers follow the progression of ideas.

**⦁ Response:** We sincerely appreciate the reviewer’s suggestion to improve the structure of the literature review. We agree that organizing the studies thematically will help to enhance the clarity and flow of ideas. In response to this comment, we have reorganized the literature review into thematic sub-sections as recommended. The new structure includes the following sub-sections:

**Themes of the existing studies,** including thematic areas such as Technology Application and Infrastructure construction, Environment Improvement and Platform Support, Standard-setting and Management Innovation, Talent Development and System Optimization. This classification also points out the main focus on CICP evaluation and optimization in current.

**Gaps in Current Research,** noting that most of the policy recommendations made by existing studies were based on expert experience and lacked empirical evidence, and puts forward the necessity and importance of introducing quantitative analysis method to evaluate CICP scientifically.

**Advances in Quantitative Policy Analysis,** presenting a series of research cases, and to demonstrate the feasibility of scientific and systematic evaluation and optimization of CICP based on multi-dimensional research framework and quantitative analysis methods.

This grouping provides a clearer progression of ideas, making it easier for readers to follow the discussion and better understanding of the progress of existing CICP research and the reasons for quantitative analysis and evaluation of CICP. We believe these changes significantly improve the overall readability of the literature review. These clarifications can be found on **lines 105-142** of the revised manuscript.

**Comment 4:** The authors employed PMC Model in the study and assumed readers are familiar with the PMC index model, making it hard for a general reader to follow. I would suggest the authors to offer a simplified explanation or a step-by-step example of how the PMC index model is applied, somewhere in between lines 204-244, possibly with a small sample policy to illustrate the process. Figures 1 and 2 could be accompanied by a brief walkthrough.

**⦁ Response:** We appreciate the reviewer’s helpful suggestion to make the application of the PMC Model more accessible to a broader audience. We agree that a more detailed explanation will enhance the clarity of the manuscript. In addition, we are aware that the presentation of the research methodology in the original text is complicated, which is not conducive to the reader's understanding. In response, we have reorganized the presentation of methodology and divided it into three parts:

**Methodological Framework:** This section explains that the analytical framework constructed in this paper relies on text mining and data visualization techniques, and covers the objective evaluation, comprehensive discussion, and optimization suggestions of CICP. In our analytical framework, the research work will be carried out according to five parts of advanced work, including the policy evaluation based on PMC Index Model and the dynamic analysis of policy evolution, status, and trend, offering data-driven recommendations for the optimization of CICP. This explanation, which is now included between **lines 220-225**, clearly outlines the process. We also revised **Figures 1** to provide a clearer explanation, helping readers understand the key concepts being visualized.

**Research Methods and Working Procedures**: This section describes how we are excuting the five parts of advanced work in the analytical framework described above,and the methods and tools uesd in the process. At the same time, we also point out the final goals and conclutions pursued by these parts of the research work. This explanation, which is now included between **lines 230-236**, clearly outlines the methods and procedures. We also revised **Figures 2** to provide a clearer explanation. Especially, we introduce the application method of the PMC index model in detail. We have added a simplified, step-by-step walkthrough of the PMC index model’s application, including an illustrative example using a small sample policy. This explanation, which is now included between **lines 239-365**, clearly outlines the process, showing how the model is applied and how the results are interpreted. We believe these additions will make the methodology more comprehensible to readers unfamiliar with the PMC Model.

**Data sources and sample selection**: This section explains where the data was obtained and processed, and details the final sample of the study. We have made no changes to this part.

We believe that the revised formulation will allow readers to understand the research methods used in this article more clearly and directly. All these modifications are included in the revised manuscript on **pages 6-11**.

**Comment 5:** The selection of the variables is the key of this study. The paper listed the variables but doesn’t explain why they are relevant to evaluating intelligent construction policies. Would the author be able to provide the model specification? At least, provide a rationale for selecting specific variables. For instance, explain why certain policy aspects (e.g., effectiveness, macro or micro perspective) are crucial in evaluating intelligent construction policies.

**⦁ Response:** We thank the reviewer for pointing out the need to clarify the rationale behind the selection of variables. We fully agree that explaining the relevance of these variables is essential for understanding their role in evaluating intelligent construction policies. In response, we have added a detailed explanation in the revised manuscript regarding the selection of each variable, focusing on their relevance to the evaluation of CICP. These clarifications can be found on **lines 387-433** of the revised manuscript.

In addition, We further add an explanation of how primary and secondary variables are selected in the methodology section. In general practice, the primary variables are employed to assess the consistency of policies. These variables exhibit minimal variation in their configuration across different studies, allowing researchers to reference classical settings from existing applications of PMC model. Secondary variables, operating within the scope of primary variables, are particularly instrumental in identifying deficiencies and shortcomings in policy formulation. The selection of corresponding variables typically derives from a systematic synthesis of research findings within the subject's domain, complemented by empirical feedback from practical implementation experiences. (see l**ines254-266** of the revised manuscript)

**Comment 6:** Around line 217, the authors mentioned briefly about the limitations, but did not discuss in depth. Please add a more detailed limitations section. For instance, mention how the quantitative nature of the PMC model might miss qualitative nuances, or how relying on textual data might overlook the practical implementation of policies in different regions.

**⦁ Response:** We appreciate the reviewer’s suggestion to provide a more detailed discussion of the limitations in our study. We agree that a thorough consideration of the limitations will offer readers a more balanced view of the methodology and findings. In response, we have expanded the limitations section of the manuscript to address the points raised by the reviewer, as well as additional considerations. Specifically, we now discuss the following limitations in more detail:

**Quantitative Nature of the PMC Model**: While the PMC index model provides a robust quantitative framework for policy evaluation, its reliance on numerical data can overlook certain qualitative nuances, such as the context-specific factors or the subjective experiences of stakeholders. However, methodological diversity characterizes policy evaluation research.There exists no universally applicable criterion for selecting policy evaluation variables across different studies, as variable determination fundamentally depends on specific research contexts, theoretical frameworks, and policy implementation environments. We also acknowledge that the model may not capture the full complexity of policy impacts, particularly those that are difficult to quantify, such as cultural or societal effects.

**Relying on Textual Data**: The study's reliance on textual data to assess policies may overlook the practical, real-world implementation challenges faced by policymakers in different regions. The textual data often reflects the intended goals and objectives of policies, but may not fully account for variations in local conditions, such as regional economic contexts, regulatory environments, or capacity for policy enforcement.

We also address potential limitations related to sample size, data availability, and generalizability of results, and provide suggestions for how future research could mitigate these challenges.

These points have been incorporated into the revised manuscript in **lines 956-986**, and we believe that this expanded discussion offers a more comprehensive understanding of the study's limitations.

**Comment 7:** Lines 333-348, authors summarized the results. The discussion scale was limited, not in depth. Please provide a more deep insight into the results. For example, explain why the shift between phases is significant. For instance, how did policy changes impact construction practices? Provide examples of how policies in Phase 2 (Development and Promotion) improved upon issues from Phase 1 (Cultivation and Exploration). Please also provide more details about the specific factors that led to the policy shift and how these changes addressed earlier issues. Discuss why Phase 2 policies are more effective and what lessons can be learned for future policymaking.

**⦁ Response:** We thank the reviewer for the insightful feedback and for encouraging us to delve deeper into the discussion of the results. In response, we have significantly expanded the discussion to provide more in-depth analysis and insight into the shift between the phases and the specific impacts of policy changes. Below are the key additions:

**Significance of the Shift Between Phases and Impact of Policy Changes on Construction Practices**

Our comprehensive analysis of the transition from the CEP to the DPP yields several critical insights:

Firstly, the evaluation of 30 CICP texts using the PMC model, combined with comparative data analysis, reveals that the introduction of “P15” around 2020 represents a pivotal milestone in CICP's evolution. Statistical evidence demonstrates a marked improvement in policy formulation quality post-2020, indicating the commencement of a new developmental phase in CICP framework.( see l**ines 471-491** of the revised manuscript)

Secondly, beyond quantitative metrics, our qualitative content analysis of these policy texts reveals substantial differences in policy formulation approaches and content characteristics between the two phases. These distinctions enable clear categorization into distinct periods: the initial exploratory cultivation phase and the subsequent development and promotion phase. Our analysis identifies and examines representative policies from each period to illustrate these evolutionary changes. ( see l**ines 491-521** of the revised manuscript)

Thirdly, the comparative assessment of policy ratings between the two phases, supported by industry scale data from 2019 and 2022, provides empirical evidence of DPP's superior effectiveness in accelerating the growth of China's intelligent construction sector. This analysis substantiates the enhanced impact of DPP policies compared to their CEP predecessors. ( see l**ines 523-530** of the revised manuscript)

Finally,the transition from CEP to DPP reflects significant methodological advancements in policy formulation, manifested through three key dimensions ( see l**ines 535-562** of the revised manuscript):

1)The shift from experimental technology promotion to comprehensive industry application

2)Increased emphasis on long-term objectives and sustainable growth

3)Strategic responses to multiple driving factors

**Factors Leading to the Policy Shift, Effectiveness of Phase 2 Policies, and Lessons for Future Policymaking**

Our extensive discussion of policy text types, content themes, intergovernmental relationships, and the coupling of policy goals and tools in the original manuscript was specifically designed to examine the factors influencing policy transition and to identify the strengths and effectiveness of Phase 2 policies. To enhance the clarity and depth of this analysis, we have implemented the following modifications:

Firstly, we emphasize that the absence of any policy receiving a "perfect" rating indicates substantial room for optimization within CICP. Prior to proposing targeted improvement recommendations, it is essential to conduct an in-depth review and discussion of CICP's evolutionary trajectory, current status, and development trends from a historical perspective. Consequently, we have established a dynamic analytical framework that enables comparative examination of CEP and DPP policy characteristics across multiple dimensions, including policy types, formulation entities, and policy objectives and tools. These analytical dimensions maintain conceptual consistency with the evaluation variables of the PMC model: text type analysis corresponds to "policy nature," intergovernmental relationship examination aligns with "issuing institutions" and "policy recipients," content theme analysis relates to "policy fields," and goal-tool analysis connects with "policy functions" and "Incentives and Constraints." By leveraging the analytical outcomes derived from the PMC model, this systematic investigation will culminate in scientifically grounded recommendations for policy refinement. ( see l**ines563-577** of the revised manuscript)

Secondly, we have synthesized the key findings and conclusions from our analysis. This synthesis encompasses the distinctive features of CICP evolution and the characteristic patterns of CICP's current status, while identifying the primary factors influencing policy shifts and driving the effectiveness of Phase 2 policies.We aslo discussed the lessons for future Policy-making. ( see l**ines 711-748** of the revised manuscript)

Finally, we propose that future policy optimization should focus on identifying existing deficiencies in CICP through PMC surface analysis, particularly in dimensions corresponding to specific evaluation variables. This analysis should be integrated with an assessment of emerging trends in China's intelligent construction sector, as identified through research frontier analysis, to formulate comprehensive recommendations and insights for policy enhancement. ( see l**ines 749-754** of the revised manuscript)

All these expanded points are now included in the revised manuscript on **pages 18-31**, and we believe that this more detailed discussion enhances the depth of our analysis and provides a clearer understanding of the policy shifts between the phases.

**Comment 8:** The most exciting section of the paper should be the recommendation section, around line 533. The recommendations given by the authors were somewhat broad and could benefit from more actionable suggestions. Instead of general statements, provide concrete actions that policymakers can take. For example, suggest specific areas where policy improvements can focus, like integrating AI more effectively into construction planning or increasing financial incentives for small and medium enterprises in construction. I would also suggest the authors, after presenting each recommendation, please link it back to a specific finding from the study. For example, if a specific policy scored lower on consistency, suggest changes to improve this aspect in future policies.

**⦁ Response:** We greatly appreciate the reviewer’s valuable feedback regarding the recommendation section. We agree that actionable, specific recommendations will be more useful to policymakers and will strengthen the practical impact of our study. In response to the reviewer’s suggestion, we have revised the recommendations section to make the suggestions more concrete and directly linked to the findings of our study. Below are the key changes we made:

**Actionable Recommendations.**

Firstly, we selected four exemplary policies from the DPP phase as research samples. Through PMC surface generation and corresponding concavity index analysis, we demonstrated and summarized the areas where CICP needs further improvement and optimization to achieve perfect policy formulation. These areas include the expansion of policy nature, enhancement of policy effectiveness, broadening of regulatory perspectives, and diversification of incentive mechanisms. These dimensions also correspond to the model variables selected in the earlier policy consistency evaluation. ( see l**ines 780-785** of the revised manuscript)

Secondly, through bibliometric analysis of China's intelligent construction field, we further identified current research hotspots. By integrating these specific research hotspots into our recommendations, we can propose targeted measures that delve into specific fields while ensuring the sustainability and alignment of future policy formulation with the development direction of China's intelligent construction. ( see l**ines 791-832** of the revised manuscript)

Finally, by combining the existing defects in CICP policy formulation with the identification of research frontiers in China's intelligent construction, we have proposed concrete and actionable policy optimization recommendations. ( see l**ines 833-876** of the revised manuscript)

**The Relevance of Recommendations to the Research Findings.**

In formulating these policy recommendations, we comprehensively considered multiple factors, including the development and current status of CICP, existing policy defects, and future trends in China's intelligent construction. Based on the four dimensions of expansion of policy nature, enhancement of policy effectiveness, broadening of regulatory perspectives, and diversification of incentive mechanisms, we have proposed corresponding policy optimization recommendations. These recommendations address specific areas within China's intelligent construction industry, such as policy support measures for the integration of AI technology and corresponding vocational training, as well as fiscal and tax incentives for SMEs. Many of these measures also respond to the findings from the empirical data analysis conducted earlier. ( see l**ines 833-876** of the revised manuscript)

We have revised the recommendation section accordingly, and these more specific, actionable suggestions are now included in the revised manuscript on **pages 32-36**.

**Comment 9:** I would suggest the authors to highlight the unique contributions of the paper. For example, emphasize how the PMC index provides a new perspective on policy evaluation or how the findings reveal important gaps in China’s current intelligent construction policies. And then point out specific suggestions for future research. For instance, suggest conducting longitudinal studies to track the long-term impact of policies or qualitative studies that involve feedback from stakeholders in the construction industry.

**⦁ Response:** We sincerely thank the reviewer for the suggestion to emphasize the unique contributions of our study. We agree that highlighting these contributions will clarify the paper's novelty and significance. In response, we have revised the conclusion section to more explicitly underscore the paper’s unique contributions and to suggest directions for future research. Below are the key additions:

**Highlighting Unique Contributions.**

*New Perspective on Policy Evaluation through the PMC Index:* We emphasize that the use of the PMC index model in this study provides a novel and quantitative framework for policy evaluation in the context of intelligent construction. Unlike traditional approaches that may focus on descriptive or qualitative analysis, the PMC index allows for a data-driven, systematic evaluation of policy effectiveness, which contributes to a more objective understanding of policy impacts. This approach fills a gap in existing literature by introducing a metric that can quantify the success of policies and guide future decision-making.

*Revealing Gaps in CICP:* We also highlight how our findings reveal significant gaps in CICP, particularly in areas such as technological integration, policy consistency, and stakeholder engagement. Our study shows that while progress has been made, there are still critical areas where policies have not been fully effective, especially in terms of scalability and implementation across regions. This gap presents opportunities for targeted policy interventions in future policy cycles.

**Suggestions for Future Research.**

*Longitudinal Studies:* We suggest that future research could focus on longitudinal studies to track the long-term impact of intelligent construction policies. Such studies would provide valuable insights into how policies evolve over time and how their effects unfold in the long run. These studies could also explore the durability of technological adoption and its sustained impact on construction practices.

*Qualitative Studies Involving Stakeholder Feedback:* We also propose conducting qualitative research that involves direct feedback from key stakeholders in the construction industry, such as policymakers, contractors, and labor force representatives. These studies could provide deeper insights into the challenges and barriers that stakeholders face in implementing policies, as well as their perceptions of policy effectiveness. Such research could complement our quantitative findings and offer a more comprehensive understanding of the practical implications of policies.

We believe that emphasizing these unique contributions and suggesting these avenues for future research will help to position the paper as a valuable reference for both scholars and policymakers. These revisions have been incorporated into **lines 946-955** and **lines 973-986** of the revised manuscript.

In addition to the specific revisions outlined above, we have thoroughly reviewed the manuscript and made additional improvements in areas such as subheadings updates, textual expressions, figure and table adjustments, etc.We have also made corresponding modifications to the abstract and conclusion sections, which can be found on **page1 and pages 36-37** of the revised manuscript. A marked-up version of the manuscript highlighting the changes is attached for your reference.

Thank you again for your time and comments. The suggestions from you are valueble for the advancement of this paper.We are happy to provide any further clarifications or revisions as necessary.

Sincerely,

**Dr. Prof. Ming Ma**
Institution: College of Architecture and Urban Planning, Yunnan University

Email: mming@ynu.edu.cn

TEL: 0086-18964833465
