# Supplementary material for: Research on quantitative evaluation of China’s Intelligent Construction Policy (CICP) based on the integration of PMC index model and multi-dimensional analytical framework
Source: PLoS One. 2025 Jul 15;20(7):e0326505. doi: 10.1371/journal.pone.0326505 (PMC12262895; doi:10.1371/journal.pone.0326505)
Supplement: Attachment — Submitted filename: Response_to_Reviewers_auresp_2.docx [file pone.0326505.s003.docx]

**Manuscript ID: PONE-D-24-31927**
**Title:** **Research on quantitative evaluation of China's Intelligent Construction Policy (CICP) based on the Integration of PMC index model and multi-dimensional analytical framework**

Dear Reviewer,

We sincerely appreciate your recognition of our revision efforts. We have carefully examined and polished the language and grammar in the paper. Please find below the implementation of your final suggestions:

|  | **Original Sentence** | **Revision** |
| --- | --- | --- |
| Line25 | conducts a comprehensive | presents a systematic |
| Line30 | reveal | demonstrate |
| Line41 | Utilizing | The adoption of |
| Lines 45-46 | These technologies are considered core because they significantly enhance construction efficiency and precision. | These core technologies significantly enhance construction efficiency and precision. |
| Line50 | Together | Collectively |
| Lines 56-57 | policies at the national level, encouraging the development of the construction industry's informatization and intellectualization. | national policies to promote digitalization and intelligent transformation in the construction industry |
| Line66 | Currently, research | Research |
| Lines 67-69 | A significant gap is the lack of quantitative evaluations of policy texts, which hinders the ability to assess their effectiveness with precision. | A critical research gap persists in the absence of rigorous quantitative evaluations of policy texts, significantly constraining the capacity to assess policy effectiveness with scientific precision. |
| Line106 | covers | spans |
| Line107 | to propose | aims to provide |
| Lines 125-127 | Although these policy suggestions based on expert experience can reflect professionalism and forward-looking in some dimensions and perspectives, it is difficult to ensure the comprehensiveness and systematization of policy evaluation. | While expert-based policy suggestions reflect professionalism and foresight, ensuring their comprehensiveness and systematic evaluation remains a challenge. |
| Line132 | made progress | advanced |
| Line141 | we aim | this research aims |
| Line170 | merges | integrates |
| Lines 230-232 | Methods that combine text encoding, data statistics, and graphical visualization were used in this research process, which could also be divided into working procedures, including Data Acquisition, Data Processing, Computation & Analysis, Results Discussion, and Conclusion. | This study employed methods such as text encoding, data analysis, and graphical visualization. The research process consisted of five stages: data acquisition, data processing, computation and analysis, results discussion, and conclusion. |
| Lines 243-244 | the CICP evaluation using the PMC Index Model is the most critical section. | the evaluation of CICP using the PMC Index Model constitutes the most critical section. |
| Lines 267-268 | The policy text (e.g., "Policy P0") is evaluated, and a standardized framework comprising ten primary variables is typically established, including "X1 Policy Nature," "X2 Policy Effectiveness," and so forth. | the policy text (e.g., 'Policy P0') is evaluated within a standardized framework of ten primary variables, including "X1 Policy Nature," "X2 Policy Effectiveness," and so forth. |
| Line274 | Policy Nature | Policy Scope |
| Line293 | Policy Nature; policy nature | Policy Scope; policy scope |
| Line381 | **Table 3. Research sample.** | **Table 3. Research sample of national intelligent construction policy texts (2010-2022).** |
| Line400 | Policy Nature | Policy Scope |
| Line401 | Policy Nature | Policy Scope |
| Line438 | , established in Table 5 | (Table 5) |
| Line451 | "Acceptable" Class | "Acceptable" |
| Line453 | considering | addressing |
| Line457 | were deemed | achieved |
| Line459 | formulate | formulation |
| Line486 | a pivotal shift | a transformative paradigm shift |
| Line517 | evolutionary progression | evolution |
| Line527 | greater effectiveness | stronger impact |
| Line572 | Policy Nature | Policy Scope |
| Line741 | Effectiveness; goals | efficacy; objectives |
| Line754 | policy nature | policy scope |
| Line770 | Policy Nature | Policy Scope |
| Line772 | Policy Nature | Policy Scope |
| Line774 | Policy Nature | Policy Scope |
| Line776 | Policy Nature | Policy Scope |
| Line783 | policy nature | policy scope |
| Line866 | policy nature | policy scope |
| Line882 | comprehensive | systematic |
| Line900 | policy nature | policy scope |
| Line916 | Policy Nature | Policy Scope |
| Line944 | By implementing these recommendations, China can | Implementing these recommendations would enable to |

Thank you again for your time and comments. The suggestions from you are valuable for the advancement of this paper. We are happy to provide any further clarifications or revisions as necessary.

Sincerely,

**Dr. Prof. Ming Ma**
Institution: College of Architecture and Urban Planning, Yunnan University

Email: mming@ynu.edu.cn

TEL: 0086-18964833465
